# Human Prostate Cancer Hallmarks Map

**DOI:** 10.1038/srep30691

**Published:** 2016-08-01

**Authors:** Dipamoy Datta, Md. Aftabuddin, Dinesh Kumar Gupta, Sanghamitra Raha, Prosenjit Sen

**Affiliations:** 1 Department of Biotechnology, Siksha Bhavana, Visva-Bharati, Santiniketan 731235, India; 2 Maulana Abul Kalam Azad University of Technology, West Bengal, Salt Lake, Sector-I, Kolkata 700064, India; 3 School of Studies in Neuroscience, Jiwaji University, Gwalior 474011, India; 4 Biological Chemistry Division, Indian Association for the Cultivation of Science, Kolkata 700032, India

## Abstract

Human prostate cancer is a complex heterogeneous disease that mainly affects elder male population of the western world with a high rate of mortality. Acquisitions of diverse sets of hallmark capabilities along with an aberrant functioning of androgen receptor signaling are the central driving forces behind prostatic tumorigenesis and its transition into metastatic castration resistant disease. These hallmark capabilities arise due to an intense orchestration of several crucial factors, including deregulation of vital cell physiological processes, inactivation of tumor suppressive activity and disruption of prostate gland specific cellular homeostasis. The molecular complexity and redundancy of oncoproteins signaling in prostate cancer demands for concurrent inhibition of multiple hallmark associated pathways. By an extensive manual curation of the published biomedical literature, we have developed Human Prostate Cancer Hallmarks Map (HPCHM), an onco-functional atlas of human prostate cancer associated signaling and events. It explores molecular architecture of prostate cancer signaling at various levels, namely key protein components, molecular connectivity map, oncogenic signaling pathway map, pathway based functional connectivity map etc. Here, we briefly represent the systems level understanding of the molecular mechanisms associated with prostate tumorigenesis by considering each and individual molecular and cell biological events of this disease process.

It is generally believed that cancer is a complex array of heteropathological disease manifestation united by some common hallmarks^1^,^2^,^3^. Prostate cancer is not an exception to this point^4^, and it is one of the most common malignancies responsible for male cancer deaths in the western world^4^,^5^,^7^,^8^. The heterogeneous^5^, multifocal^5^,^6^ nature of prostate cancer along with the late appearance of castration resistant^7^,^8^ disease form and metastatic bone invasion^7^,^8^ features makes the scenario frustratingly complex and untreatable (Fig. 1a).

The principal challenges of current cancer therapy inclusive of prostate cancer can be fundamentally grouped into three pivotal factors that together constitute an ‘Achilles heel’ in the context of molecularly targeted therapies in cancer (Fig. 1b). Its inherent nature implies that as cancer as a systems level disease^3^,^9^, there is an urgent need for intervening the cancer network at multiple hallmark level^1^,^2^. Here in this article we propose for a systems medicine based strategy (Fig. 1c) that essentially aims for a simultaneous targeting at three level- first, attack the key oncocogenic network, second, attack at the hallmark level and attack in order to break the systems modularity or oncogenic robustness. In an analogical manner, it is quiet comparable to ‘Clausewitzian dictum’, an earlier multi targeted war strategy invented and implemented by general Carl von Clausewitz during Napoleonic wartime^10^. In his most insightful scholastic masterpiece, ‘*Vom Kriege’*(*On War*)^10^ general Clausewitz, essentially pointed out that, in the context of multi target attacking, strategy should simultaneously focus both at enemy’s forces and his resources and should most specifically try to dampen enemy’s resisting ability^10^,^11^. In a parallel logical connotation, here we envision that enemy’s forces should indicate cancer protein interactome as signaling proteins in cancer is supposed to behave as a network of multiple nested networks with modular interconnections^2^,^12^. Enemy’s resources most specifically address the multifunctional role of cancer proteins as cancer is supposed to emerge from the complex interplay of different onco-functional features^1^,^2^,^3^. In another way, it could be assumed that an intertwined relationship between various cancer hallmarks most intrinsically strengthen the enemy’s resources^1^,^2^. The classic cancer hallmark concept most crucially tries to link molecular signaling events to pathological outcomes^1^,^2^,^3^. To this line, protein-protein interactions provide the most fundamental unit in oncogenic network and essentially play a vital role in the acquisition of various cancer hallmark abilities^13^. The enemy’s resisting ability here should be understood in terms of oncogenic robustness which arises as a typical consequence of functional redundancies and due to presence of alternative compensatory signaling circuitries as cancer has been previously depicted as a disease of pathways^3^,^9^. The emergence of treatment resistance in terms of chemo/radiation resistance^14^,^15^ and castration resistance (CRPC)^7^,^8^ additionally imparts the flexibilities of enemy’s resisting ability.

In support of our hypothetical proposal, here we attempt to represent a comprehensive molecular framework for unraveling the complexities of human prostate cancer associated molecular mechanisms by structuring the existing knowledge. For concept of our work, please visit at- http://www.bioinformatics.org/canjovdb/concept.php

## Results

### Human Prostate Cancer Hallmarks Map Aims & Features

**Figure 1:**
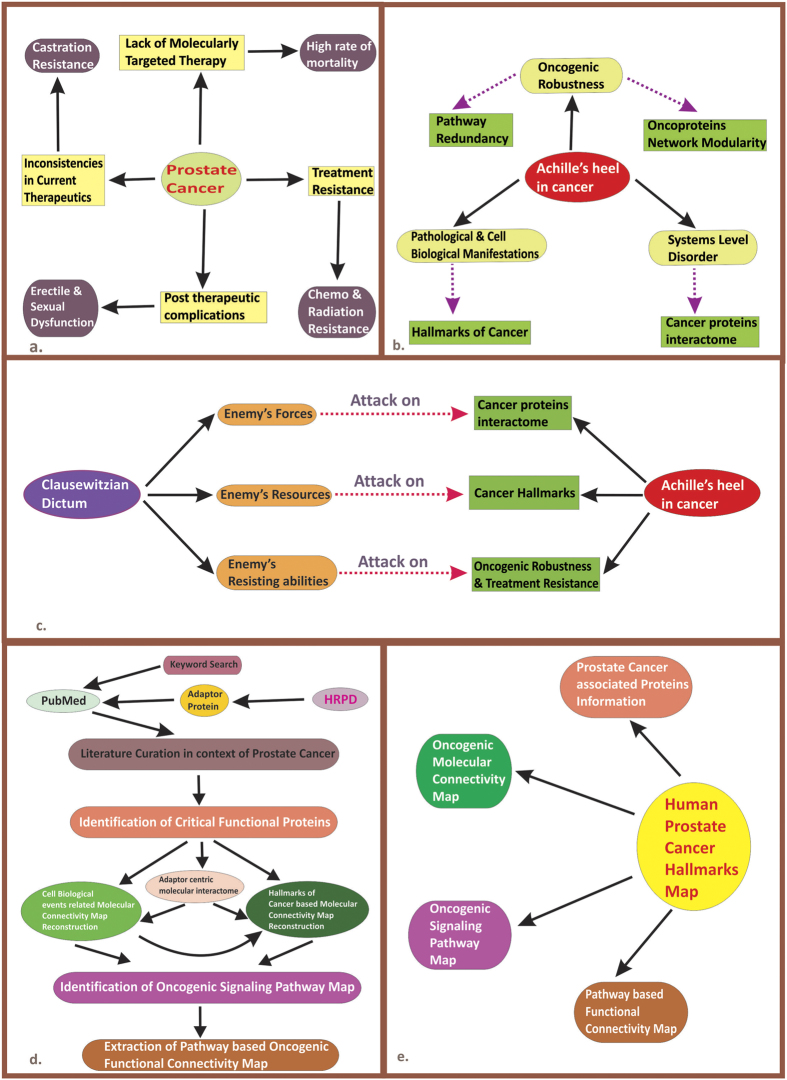
Human Prostate Cancer Hallmarks Map- A Hypothesis Driven Project. (**a**) Prostate cancer at a glance- represents some basic facts around current prostate cancer treatment approach, its limitations and its associated post therapeutic complications. (**b**) Achilles’ heel in cancer- depicts three important factors and their underlying features that most fundamentally challenges and dampens current molecularly targeted therapies in cancer. (**c**) ‘Clausewitzian Dictum’ in cancer biology- Here we propose for a multi target strategy, ‘Clausewitzian Dictum’ that essentially calls for a three level targeting- attack at the network level, attack at the cancer hallmark level, and for functional modular level. On the other hand, these three factors-cancer protein interactome network, cancer hallmark functional ability and its modular signaling network architecture together comprises the Achilles’ heel in cancer. (**d**) Our Bottom Up Approach- Adaptor protein comprises our initial seed protein. Binary protein interaction database [Human Protein Reference Database (HRPD)] provides information for adaptor protein and its physical interacting partner. For accessing the role of adaptor protein and its binary interacting partner in the context of prostate cancer, we manually curated the literature for each of adaptor protein and its corresponding interacting partner. Based on these information, we reconstruct adaptor centric molecular interactome in human prostate cancer. For gaining a functional organizational view, we then extracts various oncogenic signaling pathways maps, subnetworks and modules associated with diverse cell biological events and hallmark related phenomenon with other literature mined information’s (detailed in the material and methods section). (**e**) Human Prostate Cancer Hallmarks Map Features- It provides a comprehensive resource for revealing molecular architecture of human prostate cancer at four specific levels namely, prostate cancer hallmark & its underlying feature based phenomenon, prostate cancer hallmark based and cell biological function based molecular connectivity map, hallmark based and its underlying events related oncogenic signaling pathway map, hallmark and inter hallmark related pathway based functional connectivity map.

Human Prostate Cancer Hallmarks Map (HPCHM) is a manually constructed onco-functional atlas of human prostate tumorigenesis and is inherently based on classic cancer hallmarks concepts. It covers molecular signaling (both connectivity & pathway based) maps of most of the major cellular and pathogenic mechanisms implicated in prostate cancer progression, including 13 cancer hallmarks (10 classical & 3 prostate cancer unique hallmarks) and 11 cell biological feature based phenomenon (Table 1). It provides a comprehensive resource for revealing molecular architecture of human prostate cancer associated signaling at five specific level-first key molecular components of prostate cancer hallmarks & their underlying features (Table 2), second prostate cancer hallmark and cell biological function based molecular connectivity map (Table 1), third hallmark based and its underlying events related oncogenic signaling pathway map (Table 3), fourth hallmark and underlying cell biological feature related pathway based functional connectivity map (Tables 4 and 5), fifth inter hallmark & cell biological feature based functional connectivity map (Tables 6 and 7). Human Prostate Cancer Hallmark Map (Fig. 1e) aims to represent the systems level understanding of prostate tumorigenesis, which can be accessed at- http://www.bioinformatics.org/canjovdb.

By taking cellular signaling scaffolds or adaptor protein^16^ as seed protein, we have reconstructed the corresponding adapter centric protein-protein binary interactome map in human prostate cancer with literature mined published experimental reports (Fig. 1d). This adapter protein centric molecular interactome provides the crucial ‘scaffold’ for gaining the holistic overview of prostate tumorigenesis in the context of its various hallmarks functioning. Apart from this, we have also incorporated information by key word searching (detailed in the material and methods section). For doing so, we have manually screened over 40,000 research articles and extracted the information from 955 scientific literatures (Supplementary File 1). Our manual curation effort led to the identification of 770 molecules (Supplementary File 2) involved in various aspects of prostate cancer progression and pathogenesis. For components of adaptor centric molecular interactome map in human prostate cancer, please visit- http://www.bioinformatics.org/canjovdb/components.php.

### HPCHM content and depiction of prostate cancer allied molecular mechanisms

Here, we attempt to focus on key salient features of prostate cancer associated hallmarks in light of Human Prostate Cancer Hallmarks Map (HPCHM).

### Hallmark- Insensitivity to antigrowth signals: prostate cancer cells become unresponsive to growth restraining signals

One of the fundamental features underlying the prostate tumorigenesis involves acquisition of insensitivity to growth inhibitory signals or evasion of growth suppression. The alterations in the functional behavior of diverse sets of tumor suppressor genes by various unfavorable circumstances such as deletion, mutation, loss of expression, rearrangement, promoter methylation and haplo-insufficiency greatly contributes in this acquisition process^1^,^17^. Apart from the actions of tumor suppressor, many aberrantly working cell proliferation negative regulators, deregulated cell cycle proteins, neutralization of pro-apoptotic stimulations and extracellular ATP also plays the key leading role in the establishment of insensitivity to antigrowth signaling during the course of tumorigenesis^1^,^18^. In HPCHM, systems medicine based view of insensitivity to antigrowth signaling hallmark consists of a core network of 88 proteins with 110 physical interactions, 14 pathway based functional modules and a signaling pathway map of 90 proteins, supported by information from 24 articles. The insensitivity to antigrowth signaling hallmark pathway map depicts 3 major signaling circuitries associated with cell growth & proliferation regulation, cell cycle regulation and suppression of apoptosis^1^,^4^. For holistic map of human prostate cancer specific insensitivity to anti growth signaling, visit - http://www.bioinformatics.org/canjovdb/hallmark.php?hall=H1.

### Cell Biological Feature- Tumour Suppression

Suppression of tumor suppressive activity most critically aids in these acquisition process as progression of prostate tumorigenesis is closely associated with chromosomal rearrangement and alterations of multiple number of chromosomal regions including 1p, 6q, 7p, 7q, 8p, 10q, 13q, 16q, 17p, 17q and 18q, harboring the sites for many important tumor suppressors^19^,^20^. As a consequence of this loss of expression or functional inactivation of tumor suppressors, prostate cancer cell activates an intricate signaling circuitry that operates across diverse oncofunctional features associated signaling pathways. In HPCHM, systems medicine based view of tumor suppression contains a signaling pathway map of 93 proteins, supported by information from 45 articles. For holistic map of human prostate cancer associated tumor suppression, please visit at- http://www.bioinformatics.org/canjovdb/feature.php?feature=F1

### Cell Biological Feature- Cell Cycle

Cell cycle deregulation plays an essential leading role in the suppression of anti-proliferative signaling as majorities of anti-proliferative signals are channeled through the proteins associated with cell cycle regulatory activities and G1 to S phase transition. All of the major cell cycle regulators in prostate cancer, which also responds to anti proliferative signaling, including p21Cip1, p27Kip1, Rb, p53 are deregulated by multiple ways and are subjected to loss of function^21^. Mutational inactivation that occurs during prostate tumorigenesis have been reported for p14, p16, p27, p53 and Rb genes^21^. In HPCHM, systems medicine based view of cell cycle contains a signaling pathway map of 75 proteins, supported by information from 36 articles. For holistic map of human prostate cancer specific cell cycle, please visit at- http://www.bioinformatics.org/canjovdb/feature.php?featurety=P

### Hallmark- Self-sufficiency in growth signaling: prostate cancer cells produce their own growth signals to trigger cell growth, proliferation and survival

Three principal factors can be accounted for achieving the state of self-sufficiency in growth signaling or sustaining proliferative signaling in prostate cancer. The factors are, first, aberrant functioning of androgen receptor^22^,^23^,^24^, second, modulation of growth factor receptor activity^25^ and third, modulation of an alternative intracellular signaling circuitries associated with cell growth, survival and proliferation^6^,^8^,^26^. Most strikingly, these factors also significantly contribute in the acquisition of androgen independent phenotypes and consequent emergence of castration resistant prostate cancer^27^. Androgen receptor mediated signaling, androgen independence and castration resistance can be considered as prostate cancer unique hallmarks^7^,^8^,^22^,^23^. In HPCHM, systems medicine based view of self sufficiency in growth signaling hallmark comprises a core network of 163 proteins with 468 physical interactions, 21 pathway based functional modules and a signaling pathway map of 91 proteins, supported by information from 36 articles. The sustaining proliferative signaling hallmark pathway map represents three major signaling circuitries related to prostate cancer cell growth & tumorigenesis, cell proliferation and cell viability^1^,^4^. For holistic map, please visit- http://www.bioinformatics.org/canjovdb/hallmark.php?hall=H2

### Prostate Cancer Unique Hallmark- Androgen Receptor (AR) mediated signaling: a nuclear receptor signaling axis that plays the most pivotal role in each and every steps of prostate tumorigenesis

Numerous molecular mechanisms have been proposed for shifting of AR activation from the balanced mode to promiscuous mode in castration resistant prostate cancer (CRPC). These molecular mechanisms can be basically grouped into three major categories-First, hyper activation of androgen receptor(AR)^22^,^23^ as a result of mutation, amplification, increase in local synthesis of androgen, proteolysis, ligand independent activation and PTEN loss mediated nuclear exclusion of FOXO1 etc. Second, promiscuous activation of AR by overexpression of AR coactivators and down regulation of its corepressors^23^. Third, multiple alternative activation pathways mediated by transactivation^24^ of AR. It has been proposed that a multitude of cellular signaling pathways including PI3K/AKT, ERK, SRC etc acts in concert and converge on AR for enhancing AR mediated non-genomic response and thereby impacting self-sustenance in growth signaling^22^,^24^. For holistic view of androgen receptor mediated signaling, please visit- http://www.bioinformatics.org/canjovdb/hallmark.php?hall=H12

### Prostate Cancer Unique Hallmark- Androgen Independence: prostate cancer cells develop a variety of mechanisms to survive and flourish in an androgen deprived environment

The emergence of androgen independent phenotype is a typical characteristic of human prostate cancer development and is largely responsible for castration resistant disease progression^22^. The growth of androgen independent castration resistant prostate tumor is intensified by up regulation of both autocrine and paracrine growth factors as a consequence of an adaptive response to androgen ablation and it essentially also fuels self sufficiency in growth signaling^25^. Apart from androgen ablation therapy, various factors and a series of oncogenic signaling events contributed for the acquisition of prostate cancer androgen independence^22^. In HPCHM, systems medicine based view of androgen independence contains a signaling pathway map of 143 proteins, supported by information from 54 articles. The androgen independence map is composed of six major pathway modules devoted to the mechanisms of prostate cancer androgen independence. The modules are associated with hypersensitive pathway, outlaw pathway, bypass pathway, co-activation pathway, promiscuous pathway and lurker cell pathway^22^,^25^. For holistic map of human prostate cancer androgen independence, please visit at- http://www.bioinformatics.org/canjovdb/hallmark.php?hall=H13.

### Prostate Cancer Unique Hallmark- Castration Resistance: a late stage aggressive manifestation that results from re-growth of prostate tumor cells that has accustomed to the hormone deprived environment of the prostate

90% of prostate cancer patients finally develop castration resistant prostate cancer (CRPC), an incurable form of disease and is largely accounted for prostate cancer associated deaths with a mean survival time of only 16–18 months^7^,^8^. Modulation of an extensively rewired pathway is supposed to act for the acquisition of both self sustenance in growth signaling and the late appearance of castration resistance disease features^7^,^8^,^26^. These alternative growth and survival pathways performs normal cellular signaling processes, but become oncogenic as a consequence of two distinct means: First due to an early adaptive response to androgen ablation therapy^8^, Second and most importantly as a result of functional inactivation of many tumor suppressors, cell growth regulators and negative feed-back regulators. In HPCHM, systems medicine based view of castration resistance contains a signaling pathway map of 130 proteins, supported by information from 63 articles. The castration resistance map depicts the global view of signaling events that underlie the pathogenesis of castration resistance^4^. For holistic map of human prostate cancer castration resistance (CRPC) associated signaling pathways, please visit at- http://www.bioinformatics.org/canjovdb/hallmark.php?hall=H14

### Cell Biological Feature- Cell proliferation

Deregulated cellular proliferation is an often considered hallmark feature of prostate tumorigenesis. Although the normal prostate epithelium is generally characterized by its low rate of cell proliferation, the prostatic intraepithelial neoplasia (PIN) and early invasive prostate carcinoma exhibits seven to ten fold increase in cell proliferation rate^28^. In HPCHM, systems medicine based view of cell proliferation comprises a core network of 91 proteins with 101 physical interactions, 15 pathway based functional modules and a signaling pathway map of 90 proteins, supported by information from 58 articles. For holistic map of prostate cancer cell proliferation, please visit at- http://www.bioinformatics.org/canjovdb/feature.php?feature=F7

### Cell Biological Feature- Cell survival

A complex set of molecular mechanisms involving actions of many growth factors, intricate interplay between pro apoptotic and anti apoptotic proteins along with enhanced cell proliferation contributes together towards the enhancement of prostate cancer cell survival^8^,^26^,^27^. In HPCHM, systems medicine based view of cell proliferation comprises a core network of 91 proteins with 95 physical interactions, 9 pathway based functional modules and a signaling pathway map of 91 proteins, supported by information from 47 articles. For holistic map, visit at- http://www.bioinformatics.org/canjovdb/feature.php?feature=F3

### Cell Biological Feature- Cell growth

The normal human prostatic epithelium cell growth rate critically balances its death rate but several causes including dysregulation of AR, inactivation of tumor suppressors, evasion of apoptotic pathway and an extensively altered metabolic activity critically mediates disruption of growth regulation, resulting in enhancement of prostate tumorigenesis^8^,^26^,^27^. In HPCHM, systems medicine based view of cell growth comprises a core network of 95 proteins with 103 physical interactions, 18 pathway based functional modules and a signaling pathway map of 90 proteins, supported by information from 47 articles. For holistic map of cell growth, please visit at- http://www.bioinformatics.org/canjovdb/feature.php?feature=F4

### Hallmark- Cell death resistance: prostate cancer cells resist and modulate apoptotic program

Inhibition or evasion of apoptosis in comparison with enhanced cellular proliferation behaves as a critical pathophysiological factor for initiation and progression of majority of prostate adenocarcinomas^28^,^29^. In HPCHM, systems medicine based view of cell death resistance hallmark comprises a core network of 92 proteins with 90 physical interactions, 13 pathway based functional modules and a signaling pathway map of 89 proteins, supported by information from 24 articles. Mechanisms represented on cell death resistance map are involved in the deregulation of extrinsic & intrinsic apoptotic pathways, modulation of apoptosis resistance, inhibition of apoptotic programs, inhibition of autophagy and various androgen independent & dependent signaling events. For holistic map of prostate cancer cell death resistance, visit at- http://www.bioinformatics.org/canjovdb/hallmark.php?hall=H7

### Pathophenomic Feature- Chemoresistance & Radioresistance

A majority of the metastatic prostate cancer manifests their resistance against available cytotoxic chemotherapeutic drugs including docetaxel and paclitaxel^14^,^15^. In HPCHM, systems medicine based view of chemoresistance comprises a core network of 37 proteins with 32 physical interactions and a signaling pathway map of 37 proteins, supported by information from 19 articles. The chemoresistance map characterize the various underlying factors of prostate cancer associated chemo & radiation resistance, comprising docetaxel & paclitaxel resistance, radiation resistance, androgen receptor mediated signaling, neuroendocrine behavior and the role of transcription factors. For holistic map of human prostate cancer associated chemo-resistance & radiation resistance event, please visit at- http://www.bioinformatics.org/canjovdb/feature.php?feature=F34

### Hallmark- Angiogenesis: prostate cancer cells trigger the formation of new blood vessels

Angiogenesis is needed for each and every steps in prostate tumorigenesis, ranging from tumor outgrowth to its metastatic dissemination^30^,^31^. The microvessel density in invasive tumors of human prostatic adenoma follows a direct correlation with tumors state, grade and its clinical courses^31^. In HPCHM, systems medicine based view of angiogenesis hallmark comprises a core network of 43 proteins with 41 physical interactions, 15 pathway based functional modules and a signaling pathway map of 42 proteins, supported by information from 18 articles. The angiogenic pathway map presents the detail signaling circuitries of prostate cancer associated angiogenesis, including the activities of four distinct angiogenic switches (VEGF, HIF1A, HO-1 and CEACAM1), growth factor receptor signaling and the role of transcription factors. For holistic map of prostate cancer angiogenesis, please visit at- http://www.bioinformatics.org/canjovdb/hallmark.php?hall=H5

### Hallmark- Metastasis: the ability of prostate tumor to escape from primary organ by evading the neighboring cells and disseminate to new sites

The underlying molecular mechanism of prostate cancer metastasis is highly complex multi-step process and involves interplay of various interrelated cell biological events. Although human prostate cancer most typically follows an exceptionally slow progression pattern, but still metastatic dissemination comprises the principal cause for prostate cancer associated mortalities^7^,^8^,^32^. The development of prostate cancer metastasis mainly depends on an intense orchestration of various coordinated key cell biological events, namely cell migration, invasion, cell adhesion, cell motility and epithelial mesenchymal transition^33^. In HPCHM, systems medicine based view of metastasis hallmark comprises a core network of 157 proteins with 427 physical interactions, 14 pathway based functional modules and a signaling pathway map of 101 proteins, supported by information from 45 articles. The metastasis pathway map shows crucial cell signaling cascades underlying multifaceted metastasis hallmark, including bone metastasis^1^,^4^. For holistic map of human prostate cancer specific metastasis, visit- http://www.bioinformatics.org/canjovdb/hallmark.php?hall=H6

### Cell Biological Feature- Epithelial mesenchymal transition (EMT)

The epithelial mesenchymal transition in prostate cancer plays a leading role in the emergence of metastatic castration resistant prostate cancer (mCRPC) and functions as crucial determinants of prostate cancer metastatic dissemination^33^. In HPCHM, systems medicine based view of EMT contains a core network of 39 proteins with 34 physical interactions and a signaling pathway map of 38 proteins, supported by information from x articles. For holistic map of prostate cancer specific epithelial mesenchymal transition (EMT), please visit at- http://www.bioinformatics.org/canjovdb/feature.php?feature=F27

### Cell Biological Feature- Cell adhesion

During prostate tumorigenesis, the cell adhesion molecules^33^ provide a framework for mediating prostate cancer cell-cell interactions and an aberrant interaction between prostate glandular epithelial cells and the extracellular matrix. In HPCHM, systems medicine based view of cell adhesion comprises a core network of 41 proteins with 42 physical interactions, 4 pathway based functional modules and a signaling pathway map of 40 proteins, supported by information from 20 articles. For holistic map of human prostate cancer specific cell adhesion event, please visit at- http://www.bioinformatics.org/canjovdb/feature.php?feature=F28

### Cell Biological Feature- Cell Invasion

The earliest step of prostate cancer metastasis is the local invasion^33^ which involves entry of cancer cells from the location of primary prostate tumour into the surrounding extracellular matrix and tumor associated stromal cell layer. In HPCHM, systems medicine based view of cell invasion comprises a core network of 71 proteins with 73 physical interactions, 16 pathway based functional modules and a signaling pathway map of 71 proteins, supported by information from 50 articles. For holistic map of human prostate cancer cell invasion, please visit at- http://www.bioinformatics.org/canjovdb/feature.php?feature=F25

### Cell Biological Feature- Cell motility

Cell motility plays a very important role during the metastatic dissemination of prostate cancer^33^. In HPCHM, systems medicine based view of cell motility comprises a core network of 68 proteins with 89 physical interactions, 18 pathway based functional modules and a signaling pathway map of 66 proteins, supported by information from 28 articles. For holistic map of cell motility, please visit at- http://www.bioinformatics.org/canjovdb/feature.php?feature=F26

### Cell Biological Feature- Cell Migration

Cell migration plays a crucial role in prostate cancer metastatic progression^33^. In HPCHM, systems medicine based view of cell migration comprises a core network of 82 proteins with 93 protein-protein interactions, 18 pathway based functional modules and a signaling pathway map of 82 proteins, supported by information from 53 articles. For details, please visit at- http://www.bioinformatics.org/canjovdb/feature.php?feature=F29

### Hallmark- Inflammation: promotion of prostate tumor through enhancement of inflammatory response

Prostatic inflammation accelerates the aggressiveness of prostate tumorigenesis and its subsequent transformation into castration resistant metastatic pathological forms^34^. Prostate epithelial cells play an important role in initiating and amplifying the prostatic inflammatory processes through simultaneous activation of NF-κB pathway and by locally producing diverse pro-inflammatory cytokines that supports the recruitment and activation of additional immune cells, such as macrophage, to the prostate^35^. In HPCHM, system medicine based view of inflammation hallmark comprises a core network of 23 proteins with 63 physical interactions, 16 pathway based functional modules and a signaling pathway map of 29 proteins, supported by information from 9 articles. The inflammation signaling pathway map represents four important signaling mechanisms that critically drives prostate tumour inflammation. The signaling mechanisms include modulation of NF-kB activation, modulation of inflammatory response, inflammasome activation and immune cell activation. For details, please visit at- http://www.bioinformatics.org/canjovdb/hallmark.php?hall=H3

### Hallmark- Metabolic Reprogramming: prostate cancer cells deregulate metabolic program and energetics to support tumor formation

Although normal human prostatic epithelium cell growth rate critically balances its death rate but several factors including multivariate metabolic regulation by androgen receptor^36^, inactivation of tumor suppressors^37^ and metabolic reprogramming^38^ crucially fuels for the disruption of normal metabolic regulation, leading to an enhancement of prostate tumorigenesis. In HPCHM, systems medicine based view of metabolic reprogramming contains a signaling pathway map of 78 proteins, supported by information from 47 articles. The metabolic reprogramming oncogenic signaling map contains 12 critical metabolic switches that most significantly promotes prostate cancer cell metabolism. The metabolic switches are associated with PTEN inactivation, AMPK regulation, Warburg effect, HIF1A regulation, De novo fatty acid synthesis, De novo cholesterol synthesis, Cholesterol accumulation, Intratumoral steroidogenesis, D-Lactate metabolism, Restoration of TCA cycle, Decrease in zinc uptake and Reverse Warburg Effect. For holistic map of human prostate cancer specific metabolic reprogramming, please visit at- http://www.bioinformatics.org/canjovdb/hallmark.php?hall=H8

### Hallmark- Enabling Replicative Immortality: prostate cancer cells have unlimited replicative potential

Telomere dysfunction and reactivation of the telomerase reverse transcriptase activity, which is typically associated with telomere maintenance, plays the profound significant roles in imparting limitless replicative potential of the prostate tumor cells^39^,^40^. Telomerase activity has not been detected in both normal prostate and benign prostatic hyperplastic tissue, but over 90% of prostate cancer exhibits significantly higher level of telomerase activity^40^. Although replicative senescence is assumed a significant impediment to cellular immortalization and transformation^1^, but the deregulation of a broad spectrum of senescence regulators greatly contributes in surpassing classical senescence program that eventually enhances prostate tumorigenesis. In HPCHM, systems medicine based view of replicative immortalization contains a signaling pathway map of 38 proteins, supported by information from 27 articles. The replicative immortalization signaling map represents detail molecular mechanisms associated with expression, modulation & enhancement of telomerase (hTERT) activity, down-regulation of replicative senescence and the role of androgen deprivation therapy in modulation of prostate tumors senescence program. For holistic map of prostate cancer replicative immortalization, visit- http://www.bioinformatics.org/canjovdb/hallmark.php?hall=H10

### Hallmark- Avoidance of Immune Destruction: prostate cancer cells evade the monitoring capability of immune system

Tumor immune evasion is currently recognized as an emerging hallmark of cancer^1^ and the current research strongly suggests that evasion of immune destruction plays a crucial role in the development of prostate cancer along with its pathogenic progression^41^. A significant alteration in cell mediated immune response is particularly observed during the course of prostate tumorigenesis and it involves multiple potential immune suppressive mechanisms^41^,^42^. In HPCHM, the avoidance of immune destruction holistic map represents an integrated view of signaling events between prostate cancer cell and various immune cells, which can be accessed at- http://www.bioinformatics.org/canjovdb/hallmark.php?hall=H9

### Hallmark- Tumor Microenvironment: the surroundings of prostate tumor which critically fuels other hallmark manifestations

The tumor microenvironment in prostate cancer, which involves complex reciprocal interactions between prostate cancer epithelial cell and surrounding ‘reactive stroma’, plays the most influential role in survival, angiogenesis, inflammation and its metastatic dissemination^43^. Prostate cancer ‘reactive stroma’ is typically characterized by the presence of cancer associated fibroblastic (CAF) and myofibroblastic cell populations along with a consequential loss of smooth muscle cells and is centrally responsible for the genesis of intratumoural heterogeneity^43^,^44^. Additionally, adipocytes play a very essential role in the modulation of prostate tumour microenvironment by secreting multiple growth factors (IGF1, TGF-beta, TNF-alpha, IL-6) and inflammatory adipokines^45^. In HPCHM, the tumor microenvironmental holistic map depicts the detail paracrine signaling interactions between prostate cancer cell and its surrounding ‘reactive stroma’ components including CAF, myofibroblast and adipocytes^1^,^43^. For details of prostate cancer associated tumor microenvironment, visit at- http://www.bioinformatics.org/canjovdb/hallmark.php?hall=H11

## Discussion

Currently there is no effective molecularly targeted treatment strategy available for metastatic castration resistant prostate cancer^7^,^8^. Consequently, there is an actual need for identification of potential molecular targets and global molecular signaling map in the context of human prostate tumorigenesis, progression and treatment resistance^7^,^26^. But several challenging factors make the scenario frustratingly complex and practically infeasible. First, progression of prostate cancer involves a number of molecular and cellular processes at the same time^46^, second, prostate tumor’s heterogeneous and multifocal nature^5^,^6^, third, prostate cancer’s transition from androgen dependent to androgen unresponsive castration resistant form^7^,^8^. In addition, integrating information from multifarious sources of information across diverse systems is also a formidable challenge because of limitation of the available computational tools^2^,^47^. As a consequence, our understanding of the pathway signaling map and their modular interplay at prostate cancer hallmark level is largely lacking^4^. This is due to three principal reasons: first, although the classic cancer hallmark concept^1^ alone provides the most fundamental organizing principles of neoplastic diseases, it remains descriptive^2^ and no prostate cancer specific views are available yet^4^,^7^,^26^. Second, comprehensive information for most of cancer hallmark network in a generalized and global basis is largely unavailable there by restricting any computational based prediction^2^. Third, currently available pathway databases mainly offer the ‘canonical pathway’ based depiction, predominantly ignoring the facts that the multi stage process of tumorigenesis involves concomitantly multiple cellular processes and their signaling events^48^. For addressing all these issues, we have taken an initiative from a system medicine perspective for systems level exploration of prostate cancer associated molecular process and signaling cascades.

Although the concept of system medicine offer an unique opportunity for simplification of complex systems (by transforming medicine into a network based platform in a context specific fashion)^47^,^49^, but it is assumed that the currently available interactome maps (generated by high-throughput methods) are more than 80% incomplete^50^,^47^. From this current standpoint, compilation of already available published experimental report in the literature provides a paramount opportunity for reconstruction of interactomes thereby inferring as realistic as possible views of molecular signaling maps underlying diseases^51^. To this line, literature mined binary protein interactome maps plays a potential role in attaining hypothesis about focused biological mechanisms^13^,^51^. In the current study, we have followed a bottom up approach, which is essentially based on network medicine^47^,^51^ (Fig. 1d). Our effort reveals HPCHM, a web based contrivance, which provides the basic blue print of human prostate cancer specific ‘hallmarkosme’, an assembly of cancer hallmarks and their underlying cell biological events. This ‘hallmarkosome’ represents a set of comprehensive molecular signaling map of each and individual cellular and molecular processes related with human prostate cancer pathogenesis. For comprehensive map of human prostate cancer associated hallmarks, please visit at the following link- http://www.bioinformatics.org/canjovdb/comprehensive.php

To our knowledge, Human Prostate Cancer Hallmarks Map (HPCHM) provides the first time depiction of global molecular signaling events that are underlying human prostate tumorigenesis. Several key features make the holistic maps unique in its representation: (1) It shows most of the crucial molecular signaling cascades, their crosstalks and regulatory molecular processes and also, ‘a disease of pathway’ based view that are usually deregulated during prostate cancer development and progression; (2) It covers all major human prostate tumour specific features including androgen dependent & independent signaling views, androgen receptor (AR) mediated signaling and their regulation, the role of prostate tumour specific modulators etc; (3) It presents detailed functional view of various onco & tumour suppressor proteins, whose collaborative action functions as one of the key driver of human prostate tumorigenesis; (4) It offers an inter hallmark connectivity map, as it has been currently hypothesized that cancer hallmarks are closely intertwined^2^.

In summary, here we represent a new frame work for gaining a comprehensive understanding of cellular and molecular events underlying of human prostate cancer pathogenesis by accumulating the existing knowledge. Most importantly, Human Prostate Cancer Hallmarks Map (HPCHM) provides a major step towards systems level mechanistic representation of prostate tumorigenesis that will surely give a new space for the development of novel therapeutic approaches.

## Methods

### Initial seed gene listing

Adapter protein comprises our initial seed gene. Human Protein Reference Database (HRPD)^52^ provides information for different aspects of human proteins ranging from protein-protein interactions to molecular class information, post–translational modification to disease associations. From HRPD molecule class we got the list of 339 human adapter genes under the name of ‘Adaptor molecule’.

### Gene name normalization

A gene might appear under different synonyms. For example TICAM2 indicates toll- like receptor adaptor molecule 2 gene might shows TIRP, TRAM, TIRAP3, MyD 88-4, TICAM-2, different synonyms in biological textual article. For these reason we used the gene name provided by the HUGO Gene Nomenclature Committee GGNC database^53^.

### Literature Database searching

We manually curated the text for each of 339 adapter protein for their corresponding role in human prostate cancer. For literature search we used the NCBI PubMed database, for instance, by typing BCL2 in prostate cancer yields 159 different article containing experimentally verified information for the role of BCL2 adapter protein in prostate cancer. We manually curated each & individual of these articles for accessing the role of adapter proteins in prostate cancer.

Additionally, an extensive literature survey was followed using NCBI Pubmed for retrieving articles related to human prostate cancer associated cell biological events by key word searching, for example using search terms ‘prostate cancer cell proliferation’ etc (corresponding cell biological events). The full text articles were manually screened for cell signaling, molecular interactions, oncogenic activation & tumor suppression processes, nuclear translocation events, transcriptional gene regulation and the hyperlinked PubMed identifier for the corresponding publication. For enhancing the accuracy of this textual curation, each of the information has been simultaneously cross verified three times by different researchers.

### Binary Interaction partner selection

Adaptor proteins physical interacting partners were selected from the Human Protein Reference Database (HRPD)^52^. Each of the binary interacting partners is subjected for pubmed search in previously mentioned ways for identifying their role in human prostate cancer. In this manner, we constructed adaptor centric interactome map in prostate cancer based on proteins binary interaction along with literature curetted experimentally verified information.

### Oncogenic Signaling Map extraction

We manually constructed the comprehensive pathway maps [Molecular Connectivity Map & Oncogenic Signaling Pathway Map] for prostate cancer hallmarks, their underlying cell biological events and its associated pathophenomic features (Androgen Independence, Castration Resistance etc). These maps are intrinsically based on the molecular interactions documented in adapter centric molecular interactome. In addition, we have also incorporated information from current existing publication on molecular events associated with pathogenesis and progression of human prostate cancer.

### Pathway based Oncogenic Functional Connectivity Map reconstruction

For pathway based information, we incorporated data from various sources, ranging from literature mined information to pathway databases, including KEGG^54^, Pathway Commons^55^ and WikiPathways^56^ through both manual curation and by using WEB-based GEne SeT AnaLysis Toolkit^57^. The subnetworks were derived by using Pajek^58^ and represented in Pajek^58^ and Cytoscape^59^ based visualization platform.

## Additional Information

**How to cite this article**: Datta, D. *et al*. Human Prostate Cancer Hallmarks Map. *Sci. Rep*. **6**, 30691; doi: 10.1038/srep30691 (2016).

## Supplementary Material

Supplementary Information

## Figures and Tables

**Table 1:** Content of Human Prostate Cancer Hallmarks Map.

Cancer Hallmarks/ Cell Bilogical Features	Molecular Components	Network Statistics		Oncogenic Signaling Pathway	Pathway based Functional Connectivity Map
Classical Cancer Hallmarks	Proteins	Nodes	Edges	Proteins	No of Connectivity Maps
Evasion of growth suppression	62	88	110	90	14
Sustaning proliferative signaling	100	163	468	91	21
Tumour promoting inflammation	12	23	63	29	16
Genome instability & Mutation	56	56	125	—	19
Cell death resistance	36	92	90	89	13
Angiogenesis	23	43	41	42	15
Metastasis	106	157	427	101	14
Metabolic reprogramming	23	—	—	78	—
Avoidance of Immune Destruction	22	—	—	43	—
Replicative Immortalization	29	—	—	38	—
Tumour Microenvironment	30	—	—	74	—
Prostate Cancer Unique Hallmarks					
AR mediated signaling	17	116	115	58	—
Androgen Independence	29	—	—	143	—
Castration Resistance	49	—	—	130	—
Cell Bilogical Features					
Cell Cycle	27	—	—	75	—
Tumour Suppression	39	—	—	93	—
Cell Growth	59	95	103	90	18
Cell Survival	35	91	95	91	9
Cell Proliferation	56	91	101	90	15
Chemoresistance	7	37	32	37	—
Cell Migration	46	82	93	82	18
Cell Motility	33	68	89	66	18
Cell Adhesion	21	41	42	40	4
Cell Invasion	53	71	73	71	16
Bone Metastasis	13	13	24	—	13
Epithelial Mesenchymal Transition (EMT)	22	39	34	38	—

Node represents number of proteins and edge indicates number of binary (physical) interactions between the proteins present in network.

**Table 2:** Molecular components of Human Prostate Cancer Hallmarks Map.

Cancer Hallmarks/Cell Bilogical Features	Total Proteins(Intrinsic Components)	Adaptors	Receptors	Kinases	Enzymes	Transcription Factors & Regulators	Other Signaling Proteins	Last Update
Classical Cancer Hallmarks								
Evasion of growth suppression	62	9	8	4	4	16	21	2015
Sustaning proliferative signaling	100	23	25	18	6	14	14	2015
Tumour promoting inflammation	12	2	5	—	—	3	2	2015
Genome instability & Mutation	56	16	14	5	—	8	13	2015
Cell death resistance	36	14	8	5	2	4	3	2015
Angiogenesis	23	1	8	6	—	4	4	2015
Metastasis	106	18	20	14	5	22	27	2015
Metabolic reprogramming	23	4	2	3	3	2	9	2015
Avoidance of Immune Destruction	22	—	4	—	2	3	13	2015
Replicative Immortalization	29	1	4	3	3	9	9	2015
Tumour Microenvironment	30	1	8	1	5	3	12	2015
Prostate Cancer Unique Hallmarks								
AR mediated signaling	17	1	4	4	1	5	2	2015
Androgen Independence	29	3	7	3	3	4	9	2015
Castration Resistance	49	5	7	9	5	9	14	2015
Cell Biological Features								
Cell Cycle	27	5	2	3	1	4	12	2015
Tumour Suppression	39	5	5	1	3	12	13	2015
Cell Growth	59	10	17	14	3	7	8	2015
Cell Survival	35	9	11	7	1	3	4	2015
Cell Proliferation	56	12	11	6	3	8	16	2015
Chemoresistance	7	3	1	1	—	1	1	2015
Cell Migration	46	10	12	5	1	10	8	2015
Cell Motility	33	8	5	7	1	4	8	2015
Cell Adhesion	21	5	6	1	—	1	8	2015
Cell Invasion	53	10	11	7	2	10	13	2015
Epithelial Mesenchymal Transition (EMT)	22	—	5	2	2	7	6	2015
Bone Metastasis	13	—	7	2	—	2	2	2015

**Table 3:** Oncogenic signaling pathway map statistics

Cancer Hallmarks/Cell Bilogical Features	Total Proteins	Adaptors	Receptors	Transcription Factors & Regulators	OtherSignaling Proteins	Oncogenes	TumourSuppressor Proteins	Modulators	Gene expressions	References
Classical Cancer Hallmarks										
Evasion of growth suppression	90	10	14	—	66	14	19	6	—	24
Sustaning proliferative signaling	91	6	8	12	65	17	3	14	30	36
Tumour promoting inflammation	29	3	5	3	18	5	2	3	1	9
Cell death resistance	89	13	11	11	54	15	7	5	16	24
Angiogenesis	42	4	10	5	23	7	3	2	7	18
Metastasis	101	11	24	17	49	16	5	5	27	45
Metabolic reprogramming	78	3	16	5	54	15	8	17	12	47
Avoidance of Immune Destruction	43	1	9	3	30	6	1	2	17	21
Replicative Immortalization	38	2	3	9	24	7	7	7	3	27
Tumour Microenvironment	73	3	19	9	42	9	1	6	12	37
Prostate Cancer Unique Hallmarks										
AR mediated signaling	58	6	4	7	41	9	2	7	16	23
Androgen Independence	143	8	18	27	90	25	10	23	22	54
Castration Resistance	130	7	12	21	90	18	4	11	29	63
Cell Biological Features										
Cell Cycle	75	4	9	14	48	14	6	2	13	36
Tumour Suppression	93	4	9	0	80	14	56	3	0	45
Cell Growth	90	8	12	11	59	22	3	6	21	47
Cell Survival	91	5	20	12	54	19	4	6	23	47
Cell Proliferation	90	10	15	9	56	17	7	5	18	58
Chemoresistance	37	2	9	7	19	14	3	4	12	19
Cell Migration	82	13	18	11	40	14	7	5	12	53
Cell Motility	66	9	16	6	35	18	3	3	12	28
Cell Adhesion	40	10	10	2	18	7	2	6	6	20
Cell Invasion	71	11	17	11	32	19	7	9	15	50
Epithelial Mesenchymal Transition (EMT)	38	3	5	9	21	11	2	8	7	16

**Table 4:** Hallmarks related oncogenic functional connectivity map (pathway based) statistics.

Name of Pathway	Network statistics						
	EVGS	SPS	TPI	GIM	ANG	MET	CDR
Actin cytoskeletal regulation						N-11, E-14	
Adherens junction	N-4, E-5	N-12, E-19			N-4, E-2	N-13, E-21	
Alpha6 Beta4 mediated signaling		N-15, E-32	N-8, E-12		N-4, E-4		
Androgen receptor mediated signaling	N-4, E-5	N-16, E-35		N-11, E-19	N-6, E-9	N-13, E-30	
AP-1 transcription factor associated signaling	N-10, E-15		N-21, E-55				
ATM dependent DNA damage response	N-3, E-2						
Bladder cancer associated pathogenesis				N-10, E-9			
CDC42 mediated signaling			N-21, E-55				
Chronic myeloid leukemia pathogenesis	N-6, E-5	N-12, E-14	N-7, E-8	N-15, E-22		N-9, E-11	
Class I PI3K mediated signaling				N-48, E-110	N-13, E-20		N-20, E-27
Colorectal cancer pathogenesis	N-5, E-7	N-17, E-29	N-8, E-13	N-12, E-18	N-4, E-3	N-10, E-14	
DNA damage response		N-14, E-17		N-9, E-12			
EGFR mediated signaling		N-19, E-44				N-12, E-14	
Endometrial cancer associated pathogenesis				N-9, E-8			
ERBB mediated signaling		N-13, E-21	N-9, E-16				N-4, E-4
Focal Adhesion		N-16, E-32	N-10, E-20		N-7, E-7	N-14, E-27	N-5, E-6
Glioma associated pathogenesis		N-9, E-9	N-8, E-12	N-14, E-27	N-6, E-5	N-14, E-19	N-6, E-7
IGF1 mediated signaling						N-46, E-134	
IFN-gamma signaling		N-26, E-63					
IL-2 mediated signaling		N-15, E-24		N-9, E-11			
IL-3 mediated signaling		N-19, E-40					
IL-5 mediated signaling		N-14, E-23					
IL-6 mediated signaling		N-17, E-39	N-7, E-7				
IL-7 mediated signaling				N-7, E-7			
IL-11 mediated signaling			N-8, E-9				
Insulin mediated signaling				N-48, E-110			N-20, E-27
Integrin-linked kinase associated signaling			N-21, E-55				
JAK-STAT signaling			N-6, E-5				
Kit receptor associated signaling					N-5, E-4		N-4, E-3
Leptin signaling			N-11, E-25		N-5, E-5		
MAPK signaling					N-5, E-3	N-13, E-15	N-4, E-3
Melanoma pathogenesis		N-9, E-8		N-13, E-15		N-7, E-5	
Microtubule cytoskeletal regulation					N-5, E-6		
Neurotrophin signaling							N-4, E-3
Non small lung cancer associated pathogenesis				N-10, E-11			
Oncostatin M signaling			N-12, E-19	N-10, E-9			N-4, E-3
Pancreatic cancer pathogenesis	N-5, E-7	N-11, E-13	N-8, E-10	N-13, E-13	N-6, E-5	N-20, E-30	N-7, E-8
PAR1 mediated thrombin signaling	N-10, E-15						
PDGFR-beta associated signaling			N-23, E-63		N-13, E-20		
Prolactin signaling			N-10, E-17	N-11, E-12		N-14, E-21	
Senescence & autophagy associated signaling	N-5, E-3			N-10, E-11			N-5, E-4
TGF beta associated signaling	N-4, E-5	N-20, E-42	N-11, E-21			N-13, E-19	
TRAIL signaling							N-23, E-29
VEGF signaling					N-4, E-4		

Abbreviations: EVGS-Evasion of growth suppression, SPS-sustaining proliferative signaling, TPI-Tumor promoting inflammation, GIM-Genomic instability & mutation, ANG-Angiogenesis, MET-Metastasis, CDR-Cell death resistance, N- Node, number of protein present in the corresponding network, E-Edge, number of binary (physical) interactions between the proteins present in the corresponding network

**Table 5:** Cell biological feature related oncogenic functional connectivity map (pathway based) statistics.

Name of Pathway	Network statistics							
	CPN	CGH	CSV	CMG	CML	CIV	CAH	OVP
Actin cytoskeletal regulation				N-7, E-7				
Adherens junction	N-8, E-9	N-8, E-12		N-9, E-10	N-4, E-3	N-8, E-13		
Alpha6 Beta4 mediated signaling		N-11, E-21	N-6, E-6	N-7, E-10				
Androgen receptor mediated signaling	N-11, E-21	N-12, E-24		N-9, E-16	N-7, E-8	N-9, E-17		N-3, E-2
ARF6 trafficking event					N-16, E-31			
ATM dependent DNA damage response		N-8, E-9				N-6, E-5		
Chemokine mediated signaling		N-11, E-18		N-8, E-6	N-8, E-8			
Chronic myeloid leukemia pathogenesis		N-8, E-8				N-6, E-5		
Class I PI3K mediated signaling	N-30, E-56							N-21, E-28
Colorectal cancer pathogenesis	N-8, E-9	N-10, E-15			N-4, E-3	N-8, E-9		
EGFR mediated signaling	N-8, E-7	N-13, E-28	N-3, E-2			N-11, E-15		
Estrogen receptor mediated signaling	N-31, E-59		N-8, E-9	N-10, E-10				
Endometrial cancer associated pathogenesis	N-7, E-7							
ERBB mediated signaling	N-8, E-7	N-11, E-18				N-6, E-7		
Focal Adhesion	N-9, E-11	N-14, E-31	N-8, E-8	N-8, E-10	N-9, E-13	N-8, E-11	N-4, E-4	N-7, E-7
Glioma associated pathogenesis		N-7, E-7						
HGF mediated signaling						N-8, E-10		
IGF1 mediated signaling							N-7, E-6	
IFN-gamma signaling		N-17, E-35				N-10, E-14		
IL-2 mediated signaling				N-4, E-3				
IL-3 mediated signaling		N-11, E-21			N-6, E-5	N-9, E-11		
IL-5 mediated signaling		N-10, E-15		N-5, E-4				
IL-6 mediated signaling		N-13, E-27	N-4, E-3			N-6, E-6		
IL-11 mediated signaling					N-6, E-5			
Insulin mediated signaling	N-6, E-5		N-8, E-7					
Integrin mediated cell adhesion					N-7, E-10			
Kit receptor associated signaling		N-9, E-18			N-5, E-4			
Leptin signaling	N-8, E-6				N-9, E-11			
MAPK signaling				N-7, E-5		N-6, E-5		
Melanoma pathogenesis		N-7, E-6						
Neurotrophin signaling				N-8, E-7	N-5, E-5			
Oncostatin M signaling					N-7, E-8			
Pancreatic cancer pathogenesis	N-12, E-13			N-6, E-5		N-7, E-5		N-5, E-3
p75(NTR) mediated signaling				N-8, E-7		N-7, E-5		
Prolactin signaling	N-12, E-12				N-10, E-15		N-3, E-2	
TGF beta associated signaling	N-9, E-8	N-12, E-20	N-8, E-6	N-9, E-12		N-8, E-9		
TRAIL signaling			N-9, E-9					
VEGF signaling					N-5, E-4		N-6, E-5	
Wnt signaling		N-10, E-10		N-8, E-8				
uPAR mediated signaling								N-21, E-28

Abbreviations: CPN-Cell proliferation, CGH-Cell growth, CSV-Cell survival, CMG-Cell migration, CML-Cell motility, CIV-Cell invasion, CAH-Cell adhesion, OVP-Overexpression, N-Node, number of protein present in the corresponding network, E-Edge, number of binary [physical] interactions between the proteins present in the corresponding network.

**Table 6:** A summary of inter-hallmark functional connectivity map.

	EVGS	SPS	TPI	GIM	CDR	ANG	MET
*EVGS*		6	—	9	3	—	6
*SPS*			17	34	17	13	44
*TPI*				12	4	7	13
*GIM*					16	7	23
*CDR*						19	12
*ANG*							13
*MET*							

Abbreviations: EVGS-Evasion of growth suppression, SPS-sustaining proliferative signaling, TPI-Tumor promoting inflammation, GIM-Genomic instability & mutation, ANG-Angiogenesis, MET-Metastasis, CDR-Cell death resistance. Number indicates common protein between two corresponding hallmark.

**Table 7:** A summary of inter-cell biological feature based functional connectivity map.

	CGH	CSV	CPN	EMT	CMG	CML	CAH	CIV	CMR	BMT	OVP	TSP
*CGH*		10	17	20	17	9	19	8	16	—	10	—
*CSV*			12	16	—	29	14	15	17	6	—	—
*CPN*				18	13	32	21	12	15	6	7	5
*EMT*					19	16	9	15	19	—	—	—
*CMG*						35	24	23	26	10	9	—
*CML*							20	—	35	5	—	—
*CAH*								23	8	—	—	—
*CIV*									39	9	10	—
*CMR*										—	—	—
*BMT*											4	—
*OVP*												—
*TSP*												

Abbreviations: CGH-Cell growth, CSV-Cell survival, CPN-Cell proliferation, EMT-Epithelial mesenchymal transition, CMG-Cell migration, CML-Cell motility, CAH-Cell adhesion, CIV-Cell invasion, CMR-Chemoresistance, MBT-Bone metastasis, OVP-Overexpression, TSP-Tumour suppressor protein. Number indicates common proteins between two corresponding cell biological features.
