# Supplementary material for: Human Prostate Cancer Hallmarks Map
Source: Sci Rep. 2016 Aug 1;6:30691. doi: 10.1038/srep30691 (PMC4967902; doi:10.1038/srep30691)
Supplement: Supplementary Information [file srep30691-s1.doc]

Human Prostate Cancer Hallmarks Map

Dipamoy Datta^1^, Md Aftabuddin^2^, Dinesh Kumar Gupta^3^, Sanghamitra Raha^1^

and Prosenjit Sen^4^

^1^Department of Biotechnology, Siksha Bhavana, Visva-Bharati, Santiniketan 731235, India

^2^Maulana Abul Kalam Azad University of Technology, Salt Lake, Sector-I, Kolkata 700064, India

^3^School of Studies in Neuroscience, Jiwaji University, Gwalior 474011, India

^4^Biological Chemistry Division, Indian Association for the Cultivation of Science, Kolkata700032, India

Total References that are involved for construction of Human Prostate Cancer Hallmarks Map (HPCHM)

1. [Katsogiannou M](http://www.ncbi.nlm.nih.gov/pubmed/?term=Katsogiannou M%5BAuthor%5D&cauthor=true&cauthor_uid=25981454), [Ziouziou H](http://www.ncbi.nlm.nih.gov/pubmed/?term=Ziouziou H%5BAuthor%5D&cauthor=true&cauthor_uid=25981454), [Karaki S](http://www.ncbi.nlm.nih.gov/pubmed/?term=Karaki S%5BAuthor%5D&cauthor=true&cauthor_uid=25981454), [Andrieu C](http://www.ncbi.nlm.nih.gov/pubmed/?term=Andrieu C%5BAuthor%5D&cauthor=true&cauthor_uid=25981454), [Henry de Villeneuve M](http://www.ncbi.nlm.nih.gov/pubmed/?term=Henry de Villeneuve M%5BAuthor%5D&cauthor=true&cauthor_uid=25981454), [Rocchi P](http://www.ncbi.nlm.nih.gov/pubmed/?term=Rocchi P%5BAuthor%5D&cauthor=true&cauthor_uid=25981454). The hallmarks of castration-resistant prostate cancers. [Cancer Treat Rev.](http://www.ncbi.nlm.nih.gov/pubmed/25981454) 2015 Jul;41(7):588-97.
2. Hanahan D. & Weinberg R.A. Hallmarks of cancer: the next generation. Cell 144, 646-674 (2011).
3. Zarei N. Review of hallmarks of prostate cancer (PCa). Health Professional Student Journal 2,1 (2015).
4. Mazaris E.& Tsiotras A. Molecular pathways in prostate cancer. Nephrourol Mon 5, 792-800 (2013).
5. [Yardy GW](http://www.ncbi.nlm.nih.gov/pubmed/?term=Yardy GW%5BAuthor%5D&cauthor=true&cauthor_uid=18514389), [Bicknell DC](http://www.ncbi.nlm.nih.gov/pubmed/?term=Bicknell DC%5BAuthor%5D&cauthor=true&cauthor_uid=18514389), [Wilding JL](http://www.ncbi.nlm.nih.gov/pubmed/?term=Wilding JL%5BAuthor%5D&cauthor=true&cauthor_uid=18514389), [Bartlett S](http://www.ncbi.nlm.nih.gov/pubmed/?term=Bartlett S%5BAuthor%5D&cauthor=true&cauthor_uid=18514389). Mutations in the AXIN1 gene in advanced prostate cancer. Eur Urol. 2009 Sep;56(3):486-94.

# [**Matsumura Y**](http://www.ncbi.nlm.nih.gov/pubmed/?term=Matsumura Y%5BAuthor%5D&cauthor=true&cauthor_uid=19955841), [**Shimada K**](http://www.ncbi.nlm.nih.gov/pubmed/?term=Shimada K%5BAuthor%5D&cauthor=true&cauthor_uid=19955841), [**Tanaka N**](http://www.ncbi.nlm.nih.gov/pubmed/?term=Tanaka N%5BAuthor%5D&cauthor=true&cauthor_uid=19955841), [**Fujimoto K**](http://www.ncbi.nlm.nih.gov/pubmed/?term=Fujimoto K%5BAuthor%5D&cauthor=true&cauthor_uid=19955841). Phosphorylation status of Fas-associated death domain-containing protein regulates telomerase activity and strongly correlates with prostate cancer outcomes. [**Pathobiology**.](http://www.ncbi.nlm.nih.gov/pubmed/19955841) 2009;76(**6**):293-302.

# [**Yuan XJ**](http://www.ncbi.nlm.nih.gov/pubmed/?term=Yuan XJ%5BAuthor%5D&cauthor=true&cauthor_uid=11803475), [**Whang YE**](http://www.ncbi.nlm.nih.gov/pubmed/?term=Whang YE%5BAuthor%5D&cauthor=true&cauthor_uid=11803475). PTEN sensitizes prostate cancer cells to death receptor-mediated and drug-induced apoptosis through a FADD-dependent pathway. [**Oncogene.**](http://www.ncbi.nlm.nih.gov/pubmed/?term=Oncogene.+2002+Jan+10%3B21(2)%3A319-27.)**2002**Jan 10;**21**(**2**):**319-27**.

# Castilla C, [**Congregado B**](http://www.ncbi.nlm.nih.gov/pubmed/?term=Congregado B%5BAuthor%5D&cauthor=true&cauthor_uid=16794010), [**Chinchón D**](http://www.ncbi.nlm.nih.gov/pubmed/?term=Chinchón D%5BAuthor%5D&cauthor=true&cauthor_uid=16794010), [**Torrubia FJ**](http://www.ncbi.nlm.nih.gov/pubmed/?term=Torrubia FJ%5BAuthor%5D&cauthor=true&cauthor_uid=16794010). Bcl-xL is overexpressed in hormone-resistant prostate cancer and promotes survival of LNCaP cells via interaction with proapoptotic Bak. [**Endocrinology.**](http://www.ncbi.nlm.nih.gov/pubmed/?term=Endocrinology.+2006+Oct%3B147(10)%3A4960-7.)**2006**Oct;**147**(**10**):**4960-7**.

# [**Lamb LE**](http://www.ncbi.nlm.nih.gov/pubmed/?term=Lamb LE%5BAuthor%5D&cauthor=true&cauthor_uid=21310825), [**Zarif JC**](http://www.ncbi.nlm.nih.gov/pubmed/?term=Zarif JC%5BAuthor%5D&cauthor=true&cauthor_uid=21310825), [**Miranti CK**](http://www.ncbi.nlm.nih.gov/pubmed/?term=Miranti CK%5BAuthor%5D&cauthor=true&cauthor_uid=21310825). The androgen receptor induces integrin α6β1 to promote prostate tumor cell survival via NF-κB and Bcl-xL Independently of PI3K signaling. **Cancer Res**. **2011**Apr 1;**71**(**7**):**2739-49**.

# Kunnev D, [**Ivanov I**](http://www.ncbi.nlm.nih.gov/pubmed/?term=Ivanov I%5BAuthor%5D&cauthor=true&cauthor_uid=19737411), [**Ionov Y**](http://www.ncbi.nlm.nih.gov/pubmed/?term=Ionov Y%5BAuthor%5D&cauthor=true&cauthor_uid=19737411).Par-3 partitioning defective 3 homolog (C. elegans) and androgen-induced prostate proliferative shutoff associated protein genes are mutationally inactivated in prostate cancer cells. [**BMC Cancer.**](http://www.ncbi.nlm.nih.gov/pubmed/?term=BMC+Cancer.+2009+Sep+8%3B9%3A318.) **2009**Sep 8;**9**:**318**.

# [**Kutikhin AG**](http://www.ncbi.nlm.nih.gov/pubmed/?term=Kutikhin AG%5BAuthor%5D&cauthor=true&cauthor_uid=21745515). Role of NOD1/CARD4 and NOD2/CARD15 gene polymorphisms in cancer etiology. **Hum Immunol**.**2011**Oct;**72**(**10**):**955-68**.

# Kang MJ, [**Heo SK**](http://www.ncbi.nlm.nih.gov/pubmed/?term=Heo SK%5BAuthor%5D&cauthor=true&cauthor_uid=22228081), [**Song EJ**](http://www.ncbi.nlm.nih.gov/pubmed/?term=Song EJ%5BAuthor%5D&cauthor=true&cauthor_uid=22228081), [**Kim DJ**](http://www.ncbi.nlm.nih.gov/pubmed/?term=Kim DJ%5BAuthor%5D&cauthor=true&cauthor_uid=22228081).Activation of Nod1 and Nod2 induces innate immune responses of**prostate** epithelial cells. [**Prostate**.](http://www.ncbi.nlm.nih.gov/pubmed/?term=Prostate.+2012+Sep+1%3B72(12)%3A1351-8.) **2012**Sep 1;**72**(**12**):**1351-8**.

# [**Low CG**](http://www.ncbi.nlm.nih.gov/pubmed/?term=Low CG%5BAuthor%5D&cauthor=true&cauthor_uid=23409057), [**Luk IS**](http://www.ncbi.nlm.nih.gov/pubmed/?term=Luk IS%5BAuthor%5D&cauthor=true&cauthor_uid=23409057), [**Lin D**](http://www.ncbi.nlm.nih.gov/pubmed/?term=Lin D%5BAuthor%5D&cauthor=true&cauthor_uid=23409057), [**Fazli L**](http://www.ncbi.nlm.nih.gov/pubmed/?term=Fazli L%5BAuthor%5D&cauthor=true&cauthor_uid=23409057). BIRC6 protein, an inhibitor of apoptosis: role in survival of human prostate cancer cells. [**PLoS One.**](http://www.ncbi.nlm.nih.gov/pubmed/?term=PLoS+One.+2013%3B8(2)%3Ae55837.)2013;8(2):e55837.

# [**Viticchiè G**](http://www.ncbi.nlm.nih.gov/pubmed/?term=Viticchiè G%5BAuthor%5D&cauthor=true&cauthor_uid=21368580), [**Lena AM**](http://www.ncbi.nlm.nih.gov/pubmed/?term=Lena AM%5BAuthor%5D&cauthor=true&cauthor_uid=21368580), [**Latina A**](http://www.ncbi.nlm.nih.gov/pubmed/?term=Latina A%5BAuthor%5D&cauthor=true&cauthor_uid=21368580), [**Formosa A**](http://www.ncbi.nlm.nih.gov/pubmed/?term=Formosa A%5BAuthor%5D&cauthor=true&cauthor_uid=21368580). MiR-203 controls proliferation, migration and invasive potential of prostate cancer cell lines. [**Cell Cycle.**](http://www.ncbi.nlm.nih.gov/pubmed/?term=Cell+Cycle.+2011+Apr+1%3B10(7)%3A1121-31.)**2011**Apr 1;**10**(**7**):**1121-31**.

# [**Aita VM**](http://www.ncbi.nlm.nih.gov/pubmed/?term=Aita VM%5BAuthor%5D&cauthor=true&cauthor_uid=10395800), [**Liang XH**](http://www.ncbi.nlm.nih.gov/pubmed/?term=Liang XH%5BAuthor%5D&cauthor=true&cauthor_uid=10395800), [**Murty VV**](http://www.ncbi.nlm.nih.gov/pubmed/?term=Murty VV%5BAuthor%5D&cauthor=true&cauthor_uid=10395800). Cloning and genomic organization of beclin**1**, a candidate tumor suppressor gene on chromosome 17q21. [**Genomics.**](http://www.ncbi.nlm.nih.gov/pubmed/?term=Genomics.+1999+Jul+1%3B59(1)%3A59-65.)**1999**Jul**1**;**59**(**1**):**59-65**.

# DiPaola RS, [**Dvorzhinski D**](http://www.ncbi.nlm.nih.gov/pubmed/?term=Dvorzhinski D%5BAuthor%5D&cauthor=true&cauthor_uid=18767033), [**Thalasila A**](http://www.ncbi.nlm.nih.gov/pubmed/?term=Thalasila A%5BAuthor%5D&cauthor=true&cauthor_uid=18767033).Therapeutic starvation and autophagy in**prostate**cancer: a new paradigm for targeting metabolism in cancer therapy. [**Prostate.**](http://www.ncbi.nlm.nih.gov/pubmed/?term=Prostate.+2008+Dec+1%3B68(16)%3A1743-52.)**2008**Dec 1;**68**(**16**):**1743-52**.

# [**Shimada K**](http://www.ncbi.nlm.nih.gov/pubmed/?term=Shimada K%5BAuthor%5D&cauthor=true&cauthor_uid=16537561), [**Nakamura M**](http://www.ncbi.nlm.nih.gov/pubmed/?term=Nakamura M%5BAuthor%5D&cauthor=true&cauthor_uid=16537561), [**Matsuyoshi S**](http://www.ncbi.nlm.nih.gov/pubmed/?term=Matsuyoshi S%5BAuthor%5D&cauthor=true&cauthor_uid=16537561). Specific positive and negative effects of FLIP on cell survival in human prostate cancer. [**Carcinogenesis.**](http://www.ncbi.nlm.nih.gov/pubmed/?term=Carcinogenesis.+2006+Jul%3B27(7)%3A1349-57.)**2006**Jul;**27**(**7**):**1349-57**.

# [**Gao S**](http://www.ncbi.nlm.nih.gov/pubmed/?term=Gao S%5BAuthor%5D&cauthor=true&cauthor_uid=15731171), [**Lee P**](http://www.ncbi.nlm.nih.gov/pubmed/?term=Lee P%5BAuthor%5D&cauthor=true&cauthor_uid=15731171), [**Wang H**](http://www.ncbi.nlm.nih.gov/pubmed/?term=Wang H%5BAuthor%5D&cauthor=true&cauthor_uid=15731171). The androgen receptor directly targets the cellular Fas/FasL-associated death domain protein-like inhibitory protein gene to promote the androgen-independent growth of prostate cancer cells. [**Mol Endocrinol.**](http://www.ncbi.nlm.nih.gov/pubmed/?term=Mol+Endocrinol.+2005+Jul%3B19(7)%3A1792-802.)**2005**Jul;**19**(**7**):**1792-802**.

# [**Neuwirt H**](http://www.ncbi.nlm.nih.gov/pubmed/?term=Neuwirt H%5BAuthor%5D&cauthor=true&cauthor_uid=19342366), [**Puhr M**](http://www.ncbi.nlm.nih.gov/pubmed/?term=Puhr M%5BAuthor%5D&cauthor=true&cauthor_uid=19342366), [**Santer FR**](http://www.ncbi.nlm.nih.gov/pubmed/?term=Santer FR%5BAuthor%5D&cauthor=true&cauthor_uid=19342366).Suppressor of cytokine signaling (SOCS)-1 is expressed in human prostate cancer and exerts growth-inhibitory function through down-regulation of cyclins and cyclin-dependent kinases. [**Am J Pathol.**](http://www.ncbi.nlm.nih.gov/pubmed/?term=Am+J+Pathol.+2009+May%3B174(5)%3A1921-30.)**2009**May;**174**(**5**):**1921-30**.

# [**Suzuki M**](http://www.ncbi.nlm.nih.gov/pubmed/?term=Suzuki M%5BAuthor%5D&cauthor=true&cauthor_uid=16458425), [**Shigematsu H**](http://www.ncbi.nlm.nih.gov/pubmed/?term=Shigematsu H%5BAuthor%5D&cauthor=true&cauthor_uid=16458425), [**Shivapurkar N**](http://www.ncbi.nlm.nih.gov/pubmed/?term=Shivapurkar N%5BAuthor%5D&cauthor=true&cauthor_uid=16458425). Methylation of apoptosis related genes in the pathogenesis and prognosis of prostate cancer. [**Cancer Lett.**](http://www.ncbi.nlm.nih.gov/pubmed/?term=Cancer+Lett.+2006+Oct+28%3B242(2)%3A222-30.)**2006**Oct 28;**242**(**2**):**222-30**.

# [**Wang D**](http://www.ncbi.nlm.nih.gov/pubmed/?term=Wang D%5BAuthor%5D&cauthor=true&cauthor_uid=19934328), [**Montgomery RB**](http://www.ncbi.nlm.nih.gov/pubmed/?term=Montgomery RB%5BAuthor%5D&cauthor=true&cauthor_uid=19934328), [**Schmidt LJ**](http://www.ncbi.nlm.nih.gov/pubmed/?term=Schmidt LJ%5BAuthor%5D&cauthor=true&cauthor_uid=19934328). Reduced tumor necrosis factor receptor-associated death domain expression is associated with prostate cancer progression. [**Cancer Res.**](http://www.ncbi.nlm.nih.gov/pubmed/?term=Cancer+Res.+2009+Dec+15%3B69(24)%3A9448-56.)**2009**Dec 15;**69**(**24**):**9448-56**.

# [**Wang D**](http://www.ncbi.nlm.nih.gov/pubmed/?term=Wang D%5BAuthor%5D&cauthor=true&cauthor_uid=23402817), [**Lu J**](http://www.ncbi.nlm.nih.gov/pubmed/?term=Lu J%5BAuthor%5D&cauthor=true&cauthor_uid=23402817), [**Tindall DJ**](http://www.ncbi.nlm.nih.gov/pubmed/?term=Tindall DJ%5BAuthor%5D&cauthor=true&cauthor_uid=23402817). Androgens regulate TRAIL-induced cell death in prostate cancer cells via multiple mechanisms. [**Cancer Lett.**](http://www.ncbi.nlm.nih.gov/pubmed/?term=Cancer+Lett.+2013+Jul+10%3B335(1)%3A136-44.)**2013**Jul 10;**335**(**1**):**136-44**.

# [**Higuchi T**](http://www.ncbi.nlm.nih.gov/pubmed/?term=Higuchi T%5BAuthor%5D&cauthor=true&cauthor_uid=18008329), [**Nakamura M**](http://www.ncbi.nlm.nih.gov/pubmed/?term=Nakamura M%5BAuthor%5D&cauthor=true&cauthor_uid=18008329), [**Shimada K**](http://www.ncbi.nlm.nih.gov/pubmed/?term=Shimada K%5BAuthor%5D&cauthor=true&cauthor_uid=18008329). HRK inactivation associated with promoter methylation and LOH in **prostate**cancer. [**Prostate.**](http://www.ncbi.nlm.nih.gov/pubmed/?term=Prostate.+2008+Jan+1%3B68(1)%3A105-13.)**2008**Jan**1**;**68**(**1**):**105-13**.

# [**Kwan PS**](http://www.ncbi.nlm.nih.gov/pubmed/?term=Kwan PS%5BAuthor%5D&cauthor=true&cauthor_uid=23239745), [**Lau CC**](http://www.ncbi.nlm.nih.gov/pubmed/?term=Lau CC%5BAuthor%5D&cauthor=true&cauthor_uid=23239745), [**Chiu YT**](http://www.ncbi.nlm.nih.gov/pubmed/?term=Chiu YT%5BAuthor%5D&cauthor=true&cauthor_uid=23239745).Daxx regulates mitotic progression and prostate cancer predisposition. [**Carcinogenesis.**](http://www.ncbi.nlm.nih.gov/pubmed/?term=Carcinogenesis.+2013+Apr%3B34(4)%3A750-9.)**2013**Apr;**34**(**4**):**750-9**.

# [**Zhang XA**](http://www.ncbi.nlm.nih.gov/pubmed/?term=Zhang XA%5BAuthor%5D&cauthor=true&cauthor_uid=12738793), [**He B**](http://www.ncbi.nlm.nih.gov/pubmed/?term=He B%5BAuthor%5D&cauthor=true&cauthor_uid=12738793), [**Zhou B**](http://www.ncbi.nlm.nih.gov/pubmed/?term=Zhou B%5BAuthor%5D&cauthor=true&cauthor_uid=12738793). Requirement of the p130CAS-Crk coupling for metastasis suppressor KAI1/CD82-mediated inhibition of cell migration. [**J Biol Chem.**](http://www.ncbi.nlm.nih.gov/pubmed/?term=J+Biol+Chem.+2003+Jul+18%3B278(29)%3A27319-28.)**2003**Jul 18;**278**(**29**):**27319-28**.

# Shaikhibrahim Z, [**Langer B**](http://www.ncbi.nlm.nih.gov/pubmed/?term=Langer B%5BAuthor%5D&cauthor=true&cauthor_uid=21258770), [**Lindstrot A**](http://www.ncbi.nlm.nih.gov/pubmed/?term=Lindstrot A%5BAuthor%5D&cauthor=true&cauthor_uid=21258770). Ets-1 is implicated in the regulation of androgen co-regulator FHL2 and reveals specificity for migration, but not invasion, of PC3 prostate cancer cells. [**Oncol Rep.**](http://www.ncbi.nlm.nih.gov/pubmed/?term=Oncol+Rep.+2011+Apr%3B25(4)%3A1125-9.)**2011**Apr;**25**(**4**):**1125-9**.

# [**Heemers HV**](http://www.ncbi.nlm.nih.gov/pubmed/?term=Heemers HV%5BAuthor%5D&cauthor=true&cauthor_uid=17975004), [**Regan KM**](http://www.ncbi.nlm.nih.gov/pubmed/?term=Regan KM%5BAuthor%5D&cauthor=true&cauthor_uid=17975004), [**Dehm SM**](http://www.ncbi.nlm.nih.gov/pubmed/?term=Dehm SM%5BAuthor%5D&cauthor=true&cauthor_uid=17975004). Androgen induction of the androgen receptor coactivator four and a half LIM domain protein-2: evidence for a role for serum response factor in prostate cancer. [**Cancer Res.**](http://www.ncbi.nlm.nih.gov/pubmed/?term=Cancer+Res.+2007+Nov+1%3B67(21)%3A10592-9.)**2007**Nov 1;**67**(**21**):**10592-9**.

# [**Yang Y**](http://www.ncbi.nlm.nih.gov/pubmed/?term=Yang Y%5BAuthor%5D&cauthor=true&cauthor_uid=15692560), [**Hou H**](http://www.ncbi.nlm.nih.gov/pubmed/?term=Hou H%5BAuthor%5D&cauthor=true&cauthor_uid=15692560), [**Haller EM**](http://www.ncbi.nlm.nih.gov/pubmed/?term=Haller EM%5BAuthor%5D&cauthor=true&cauthor_uid=15692560). Suppression of FOXO1 activity by FHL2 through SIRT1-mediated deacetylation. [**EMBO J.**](http://www.ncbi.nlm.nih.gov/pubmed/?term=EMBO+J.+2005+Mar+9%3B24(5)%3A1021-32.)**2005**Mar 9;**24**(**5**):**1021-32**.

# [**Kahl P**](http://www.ncbi.nlm.nih.gov/pubmed/?term=Kahl P%5BAuthor%5D&cauthor=true&cauthor_uid=17145880), [**Gullotti L**](http://www.ncbi.nlm.nih.gov/pubmed/?term=Gullotti L%5BAuthor%5D&cauthor=true&cauthor_uid=17145880), [**Heukamp LC**](http://www.ncbi.nlm.nih.gov/pubmed/?term=Heukamp LC%5BAuthor%5D&cauthor=true&cauthor_uid=17145880). Androgen receptor coactivators lysine-specific histone demethylase 1 and four and a half LIM domain protein 2 predict risk of prostate cancer recurrence. [**Cancer Res.**](http://www.ncbi.nlm.nih.gov/pubmed/?term=Cancer+Res.+2006+Dec+1%3B66(23)%3A11341-7.)**2006**Dec 1;**66**(**23**):**11341-7**.

# [**Yang Y**](http://www.ncbi.nlm.nih.gov/pubmed/?term=Yang Y%5BAuthor%5D&cauthor=true&cauthor_uid=23652996), [**Jiao L**](http://www.ncbi.nlm.nih.gov/pubmed/?term=Jiao L%5BAuthor%5D&cauthor=true&cauthor_uid=23652996), [**Hou J**](http://www.ncbi.nlm.nih.gov/pubmed/?term=Hou J%5BAuthor%5D&cauthor=true&cauthor_uid=23652996). Dishevelled-2 silencing reduces androgen-dependent prostate tumor cell proliferation and migration and expression of Wnt-3a and matrix metalloproteinases. [**Mol Biol Rep.**](http://www.ncbi.nlm.nih.gov/pubmed/?term=Mol+Biol+Rep.+2013+Jul%3B40(7)%3A4241-50.)**2013**Jul;**40**(**7**):**4241-50**.

# [**Zheng C**](http://www.ncbi.nlm.nih.gov/pubmed/?term=Zheng C%5BAuthor%5D&cauthor=true&cauthor_uid=21487968), [**Yinghao S**](http://www.ncbi.nlm.nih.gov/pubmed/?term=Yinghao S%5BAuthor%5D&cauthor=true&cauthor_uid=21487968), [**Li J**](http://www.ncbi.nlm.nih.gov/pubmed/?term=Li J%5BAuthor%5D&cauthor=true&cauthor_uid=21487968). MiR-221 expression affects invasion potential of human prostate carcinoma cell lines by targeting DVL2. [**Med Oncol.**](http://www.ncbi.nlm.nih.gov/pubmed/?term=Med+Oncol. 2012+Jun%3B29(2)%3A815-22.)**2012**Jun;29(**2**):815-22.

# [**Reebye V**](http://www.ncbi.nlm.nih.gov/pubmed/?term=Reebye V%5BAuthor%5D&cauthor=true&cauthor_uid=20688158), [**Bevan CL**](http://www.ncbi.nlm.nih.gov/pubmed/?term=Bevan CL%5BAuthor%5D&cauthor=true&cauthor_uid=20688158), [**Nohadani M**](http://www.ncbi.nlm.nih.gov/pubmed/?term=Nohadani M%5BAuthor%5D&cauthor=true&cauthor_uid=20688158). Interaction between AR signalling and CRKL bypasses casodex inhibition in prostate cancer. Cell Signal. 2010 Dec;22(12):1874-81.

# [**Mizutani K**](http://www.ncbi.nlm.nih.gov/pubmed/?term=Mizutani K%5BAuthor%5D&cauthor=true&cauthor_uid=16457155), [**Miyamoto S**](http://www.ncbi.nlm.nih.gov/pubmed/?term=Miyamoto S%5BAuthor%5D&cauthor=true&cauthor_uid=16457155), [**Nagahata T**](http://www.ncbi.nlm.nih.gov/pubmed/?term=Nagahata T%5BAuthor%5D&cauthor=true&cauthor_uid=16457155). Upregulation and overexpression of DVL1, the human counterpart of the Drosophila dishevelled gene, in prostate cancer. Tumori. 2005 Nov-Dec;91(6):546-51.

# [**Murata T**](http://www.ncbi.nlm.nih.gov/pubmed/?term=Murata T%5BAuthor%5D&cauthor=true&cauthor_uid=22904106), [**Takayama K**](http://www.ncbi.nlm.nih.gov/pubmed/?term=Takayama K%5BAuthor%5D&cauthor=true&cauthor_uid=22904106), [**Urano T**](http://www.ncbi.nlm.nih.gov/pubmed/?term=Urano T%5BAuthor%5D&cauthor=true&cauthor_uid=22904106). 14-3-3ζ, a novel androgen-responsive gene, is upregulated in prostate cancer and promotes prostate cancer cell proliferation and survival. [**Clin Cancer Res.**](http://www.ncbi.nlm.nih.gov/pubmed/?term=Clin+Cancer+Res.+2012+Oct+15%3B18(20)%3A5617-27.)**2012**Oct 15;**18**(**20**):**5617-27**.

# [**Goc A**](http://www.ncbi.nlm.nih.gov/pubmed/?term=Goc A%5BAuthor%5D&cauthor=true&cauthor_uid=22808202), [**Abdalla M**](http://www.ncbi.nlm.nih.gov/pubmed/?term=Abdalla M%5BAuthor%5D&cauthor=true&cauthor_uid=22808202), [**Al-Azayzih A**](http://www.ncbi.nlm.nih.gov/pubmed/?term=Al-Azayzih A%5BAuthor%5D&cauthor=true&cauthor_uid=22808202)**.Rac1 activation driven**by 14-3-3ζ**dimerization promotes prostate cancercell-matrix interactions**,**motility**and**transendothelial migration**. [**PLoS One.**](http://www.ncbi.nlm.nih.gov/pubmed/?term=Rac1+Activation+Driven+by+14-3-3ζ+Dimerization+Promotes+Prostate+Cancer+Cell-Matrix+Interactions%2C+Motility+and+Transendothelial+Migration)2012;7(7):e40594.

# [**Macoska JA**](http://www.ncbi.nlm.nih.gov/pubmed/?term=Macoska JA%5BAuthor%5D&cauthor=true&cauthor_uid=7585607), [**Trybus TM**](http://www.ncbi.nlm.nih.gov/pubmed/?term=Trybus TM%5BAuthor%5D&cauthor=true&cauthor_uid=7585607), [**Benson PD**](http://www.ncbi.nlm.nih.gov/pubmed/?term=Benson PD%5BAuthor%5D&cauthor=true&cauthor_uid=7585607). Evidence for three tumor suppressor gene loci on chromosome 8p in human prostate cancer. Cancer Res. 1995 Nov 15;55(22):5390-5.

# [**Ge K**](http://www.ncbi.nlm.nih.gov/pubmed/?term=Ge K%5BAuthor%5D&cauthor=true&cauthor_uid=10738240), [**Minhas F**](http://www.ncbi.nlm.nih.gov/pubmed/?term=Minhas F%5BAuthor%5D&cauthor=true&cauthor_uid=10738240), [**Duhadaway J**](http://www.ncbi.nlm.nih.gov/pubmed/?term=Duhadaway J%5BAuthor%5D&cauthor=true&cauthor_uid=10738240). Loss of heterozygosity and tumor suppressor activity of Bin1 in prostate carcinoma. Int J Cancer. 2000 Apr 15;86(2):155-61.

# [**Kuznetsova EB**](http://www.ncbi.nlm.nih.gov/pubmed/?term=Kuznetsova EB%5BAuthor%5D&cauthor=true&cauthor_uid=17477881), [**Kekeeva TV**](http://www.ncbi.nlm.nih.gov/pubmed/?term=Kekeeva TV%5BAuthor%5D&cauthor=true&cauthor_uid=17477881), [**Larin SS**](http://www.ncbi.nlm.nih.gov/pubmed/?term=Larin SS%5BAuthor%5D&cauthor=true&cauthor_uid=17477881). Methylation of the BIN1 gene promoter CpG island associated with breast and prostate cancer. [**J Carcinog.**](http://www.ncbi.nlm.nih.gov/pubmed/?term=J+Carcinog.+2007+May+4%3B6%3A9.)**2007**May 4;**6**:**9**.

# [**Gurumurthy S**](http://www.ncbi.nlm.nih.gov/pubmed/?term=Gurumurthy S%5BAuthor%5D&cauthor=true&cauthor_uid=12085964), [**Vasudevan KM**](http://www.ncbi.nlm.nih.gov/pubmed/?term=Vasudevan KM%5BAuthor%5D&cauthor=true&cauthor_uid=12085964). Regulation of apoptosis in prostate cancer. Cancer Metastasis Rev. 2001;20(3-4):225-43.

# [**Trerotola M**](http://www.ncbi.nlm.nih.gov/pubmed/?term=Trerotola M%5BAuthor%5D&cauthor=true&cauthor_uid=22378065) [**Li J**](http://www.ncbi.nlm.nih.gov/pubmed/?term=Li J%5BAuthor%5D&cauthor=true&cauthor_uid=22378065), [**Alberti S**](http://www.ncbi.nlm.nih.gov/pubmed/?term=Alberti S%5BAuthor%5D&cauthor=true&cauthor_uid=22378065). Trop-2 inhibits prostate cancer cell adhesion to fibronectin through the β1 integrin-RACK1 axis. J Cell Physiol. 2012 Nov;227(11):3670-7.

# [**Shen F**](http://www.ncbi.nlm.nih.gov/pubmed/?term=Shen F%5BAuthor%5D&cauthor=true&cauthor_uid=23912224), [**Yan C**](http://www.ncbi.nlm.nih.gov/pubmed/?term=Yan C%5BAuthor%5D&cauthor=true&cauthor_uid=23912224), [**Liu M**](http://www.ncbi.nlm.nih.gov/pubmed/?term=Liu M%5BAuthor%5D&cauthor=true&cauthor_uid=23912224). RACK1 promotes prostate cancer cell proliferation, invasion and metastasis. Mol Med Rep. 2013 Oct;8(4):999-1004.

# [**Shi XB**](http://www.ncbi.nlm.nih.gov/pubmed/?term=Shi XB%5BAuthor%5D&cauthor=true&cauthor_uid=18056640), [**Xue L**](http://www.ncbi.nlm.nih.gov/pubmed/?term=Xue L%5BAuthor%5D&cauthor=true&cauthor_uid=18056640), [**Yang J**](http://www.ncbi.nlm.nih.gov/pubmed/?term=Yang J%5BAuthor%5D&cauthor=true&cauthor_uid=18056640). An androgen-regulated miRNA suppresses Bak1 expression and induces androgen-independent growth of prostate cancer cells. Proc Natl Acad Sci U S A. 2007 Dec 11;104(50):19983-8.

# [**Nelson JB**](http://www.ncbi.nlm.nih.gov/pubmed/?term=Nelson JB%5BAuthor%5D&cauthor=true&cauthor_uid=16026642), [**Udan MS**](http://www.ncbi.nlm.nih.gov/pubmed/?term=Udan MS%5BAuthor%5D&cauthor=true&cauthor_uid=16026642), [**Guruli G**](http://www.ncbi.nlm.nih.gov/pubmed/?term=Guruli G%5BAuthor%5D&cauthor=true&cauthor_uid=16026642). Endothelin-1 inhibits apoptosis in prostate cancer. Neoplasia. 2005 Jul;7(7):631-7.

# [**Zhoul J**](http://www.ncbi.nlm.nih.gov/pubmed/?term=Zhoul J%5BAuthor%5D&cauthor=true&cauthor_uid=16267015), [**Hernandez G**](http://www.ncbi.nlm.nih.gov/pubmed/?term=Hernandez G%5BAuthor%5D&cauthor=true&cauthor_uid=16267015), [**Tu SW**](http://www.ncbi.nlm.nih.gov/pubmed/?term=Tu SW%5BAuthor%5D&cauthor=true&cauthor_uid=16267015). The role of DOC-2/DAB2 in modulating androgen receptor-mediated cell growth via the nongenomic c-Src-mediated pathway in normal prostatic epithelium and cancer. Cancer Res. 2005 Nov 1;65(21):9906-13.

# [**Zhou J**](http://www.ncbi.nlm.nih.gov/pubmed/?term=Zhou J%5BAuthor%5D&cauthor=true&cauthor_uid=12473651), [**Scholes J**](http://www.ncbi.nlm.nih.gov/pubmed/?term=Scholes J%5BAuthor%5D&cauthor=true&cauthor_uid=12473651), [**Hsieh JT**](http://www.ncbi.nlm.nih.gov/pubmed/?term=Hsieh JT%5BAuthor%5D&cauthor=true&cauthor_uid=12473651). Characterization of a novel negative regulator (DOC-2/DAB2) of c-Src in normal prostatic epithelium and cancer. J Biol Chem. 2003 Feb 28;278(9):6936-41.

# [**Tseng CP**](http://www.ncbi.nlm.nih.gov/pubmed/?term=Tseng CP%5BAuthor%5D&cauthor=true&cauthor_uid=10542228), [**Ely BD**](http://www.ncbi.nlm.nih.gov/pubmed/?term=Ely BD%5BAuthor%5D&cauthor=true&cauthor_uid=10542228). The role of DOC-2/DAB2 protein phosphorylation in the inhibition of AP-1 activity. An underlying mechanism of its tumor-suppressive function in prostate cancer. J Biol Chem. 1999 Nov 5;274(45):31981-6.

# [**Puri C**](http://www.ncbi.nlm.nih.gov/pubmed/?term=Puri C%5BAuthor%5D&cauthor=true&cauthor_uid=19855435), [**Chibalina MV**](http://www.ncbi.nlm.nih.gov/pubmed/?term=Chibalina MV%5BAuthor%5D&cauthor=true&cauthor_uid=19855435), [**Arden SD**](http://www.ncbi.nlm.nih.gov/pubmed/?term=Arden SD%5BAuthor%5D&cauthor=true&cauthor_uid=19855435). Overexpression of myosin VI in prostate cancer cells enhances PSA and VEGF secretion, but has no effect on endocytosis. Oncogene. 2010 Jan 14;29(2):188-200.

# [**Iwasaki M**](http://www.ncbi.nlm.nih.gov/pubmed/?term=Iwasaki M%5BAuthor%5D&cauthor=true&cauthor_uid=17974966), [**Homma S**](http://www.ncbi.nlm.nih.gov/pubmed/?term=Homma S%5BAuthor%5D&cauthor=true&cauthor_uid=17974966), [**Hishiya A**](http://www.ncbi.nlm.nih.gov/pubmed/?term=Hishiya A%5BAuthor%5D&cauthor=true&cauthor_uid=17974966). BAG3 regulates motility and adhesion of epithelial cancer cells. Cancer Res. 2007 Nov 1;67(21):10252-9.

# [**Puhr M**](http://www.ncbi.nlm.nih.gov/pubmed/?term=Puhr M%5BAuthor%5D&cauthor=true&cauthor_uid=19738059), [**Santer FR**](http://www.ncbi.nlm.nih.gov/pubmed/?term=Santer FR%5BAuthor%5D&cauthor=true&cauthor_uid=19738059), [**Neuwirt H**](http://www.ncbi.nlm.nih.gov/pubmed/?term=Neuwirt H%5BAuthor%5D&cauthor=true&cauthor_uid=19738059). Down-regulation of suppressor of cytokine signaling-3 causes prostate cancer cell death through activation of the extrinsic and intrinsic apoptosis pathways. Cancer Res. 2009 Sep 15;69(18):7375-84.

# [**Bellezza I**](http://www.ncbi.nlm.nih.gov/pubmed/?term=Bellezza I%5BAuthor%5D&cauthor=true&cauthor_uid=17148681), [**Neuwirt H**](http://www.ncbi.nlm.nih.gov/pubmed/?term=Neuwirt H%5BAuthor%5D&cauthor=true&cauthor_uid=17148681), [**Nemes C**](http://www.ncbi.nlm.nih.gov/pubmed/?term=Nemes C%5BAuthor%5D&cauthor=true&cauthor_uid=17148681). Suppressor of cytokine signaling-3 antagonizes cAMP effects on proliferation and apoptosis and is expressed in human prostate cancer. Am J Pathol. 2006 Dec;169(6):2199-208.

# [**Horndasch M**](http://www.ncbi.nlm.nih.gov/pubmed/?term=Horndasch M%5BAuthor%5D&cauthor=true&cauthor_uid=21308719), [**Culig Z**](http://www.ncbi.nlm.nih.gov/pubmed/?term=Culig Z%5BAuthor%5D&cauthor=true&cauthor_uid=21308719). .SOCS-3 antagonizes pro-apoptotic effects of TRAIL and resveratrol in**prostate**cancer cells. Prostate. 2011 Sep;71(12):1357-66.

# [**Verdoodt B**](http://www.ncbi.nlm.nih.gov/pubmed/?term=Verdoodt B%5BAuthor%5D&cauthor=true&cauthor_uid=23612742), [**Neid M**](http://www.ncbi.nlm.nih.gov/pubmed/?term=Neid M%5BAuthor%5D&cauthor=true&cauthor_uid=23612742), [**Vogt M**](http://www.ncbi.nlm.nih.gov/pubmed/?term=Vogt M%5BAuthor%5D&cauthor=true&cauthor_uid=23612742). MicroRNA-205, a novel regulator of the anti-apoptotic protein Bcl2, is downregulated in prostate cancer. Int J Oncol. 2013 Jul;43(1):307-14.

# [**Bonci D**](http://www.ncbi.nlm.nih.gov/pubmed/?term=Bonci D%5BAuthor%5D&cauthor=true&cauthor_uid=18931683), [**Coppola V**](http://www.ncbi.nlm.nih.gov/pubmed/?term=Coppola V%5BAuthor%5D&cauthor=true&cauthor_uid=18931683), [**Musumeci M**](http://www.ncbi.nlm.nih.gov/pubmed/?term=Musumeci M%5BAuthor%5D&cauthor=true&cauthor_uid=18931683). The miR-15a-miR-16-1 cluster controls prostate cancer by targeting multiple oncogenic activities. Nat Med. 2008 Nov;14(11):1271-7.

1. [Scott SL](http://www.ncbi.nlm.nih.gov/pubmed/?term=Scott SL%5BAuthor%5D&cauthor=true&cauthor_uid=12537668), [Higdon R](http://www.ncbi.nlm.nih.gov/pubmed/?term=Higdon R%5BAuthor%5D&cauthor=true&cauthor_uid=12537668), [Beckett L](http://www.ncbi.nlm.nih.gov/pubmed/?term=Beckett L%5BAuthor%5D&cauthor=true&cauthor_uid=12537668). BCL2 antisense reduces prostate cancer cell survival following irradiation. Cancer Biother Radiopharm. 2002 Dec;17(6):647-56.

# [**Herrmann JL**](http://www.ncbi.nlm.nih.gov/pubmed/?term=Herrmann JL%5BAuthor%5D&cauthor=true&cauthor_uid=9260915), [**Menter DG**](http://www.ncbi.nlm.nih.gov/pubmed/?term=Menter DG%5BAuthor%5D&cauthor=true&cauthor_uid=9260915), [**Beham A**](http://www.ncbi.nlm.nih.gov/pubmed/?term=Beham A%5BAuthor%5D&cauthor=true&cauthor_uid=9260915). Regulation of lipid signaling pathways for cell survival and apoptosis by bcl-**2**in prostate carcinoma cells. Exp Cell Res. 1997 Aug 1;234(2):442-51.

# [**Guo Y**](http://www.ncbi.nlm.nih.gov/pubmed/?term=Guo Y%5BAuthor%5D&cauthor=true&cauthor_uid=9486855), [**Kyprianou N**](http://www.ncbi.nlm.nih.gov/pubmed/?term=Kyprianou N%5BAuthor%5D&cauthor=true&cauthor_uid=9486855). Overexpression of transforming growth factor (TGF) beta1 type II receptor restores TGF-beta1 sensitivity and signaling in human prostate cancer cells. Cell Growth Differ. 1998 Feb;9(2):185-93.

# [**Bello-DeOcampo D**](http://www.ncbi.nlm.nih.gov/pubmed/?term=Bello-DeOcampo D%5BAuthor%5D&cauthor=true&cauthor_uid=12643470), [**Tindall DJ**](http://www.ncbi.nlm.nih.gov/pubmed/?term=Tindall DJ%5BAuthor%5D&cauthor=true&cauthor_uid=12643470). TGF-betal/Smad signaling in prostate cancer. Curr Drug Targets. 2003 Apr;4(3):197-207.

# [**Li X**](http://www.ncbi.nlm.nih.gov/pubmed/?term=Li X%5BAuthor%5D&cauthor=true&cauthor_uid=18724388), [**Placencio V**](http://www.ncbi.nlm.nih.gov/pubmed/?term=Placencio V%5BAuthor%5D&cauthor=true&cauthor_uid=18724388), [**Iturregui JM**](http://www.ncbi.nlm.nih.gov/pubmed/?term=Iturregui JM%5BAuthor%5D&cauthor=true&cauthor_uid=18724388). Prostate tumor progression is mediated by a paracrine TGF-beta/Wnt3a signaling axis. Oncogene. 2008 Nov 27;27(56):7118-3.

# [**Tu WH**](http://www.ncbi.nlm.nih.gov/pubmed/?term=Tu WH%5BAuthor%5D&cauthor=true&cauthor_uid=12869309), [**Thomas TZ**](http://www.ncbi.nlm.nih.gov/pubmed/?term=Thomas TZ%5BAuthor%5D&cauthor=true&cauthor_uid=12869309), [**Masumori N**](http://www.ncbi.nlm.nih.gov/pubmed/?term=Masumori N%5BAuthor%5D&cauthor=true&cauthor_uid=12869309). The loss of TGF-beta signaling promotes prostate cancer metastasis. Neoplasia. 2003 May; 5(3): 267–277.

# [**Jones E**](http://www.ncbi.nlm.nih.gov/pubmed/?term=Jones E%5BAuthor%5D&cauthor=true&cauthor_uid=19236240), [**Pu H**](http://www.ncbi.nlm.nih.gov/pubmed/?term=Pu H%5BAuthor%5D&cauthor=true&cauthor_uid=19236240), [**Kyprianou N**](http://www.ncbi.nlm.nih.gov/pubmed/?term=Kyprianou N%5BAuthor%5D&cauthor=true&cauthor_uid=19236240). Targeting TGF-beta in prostate cancer: therapeutic possibilities during tumor progression. Expert Opin Ther Targets. 2009 Feb;13(2):227-34.

# [**Li X**](http://www.ncbi.nlm.nih.gov/pubmed/?term=Li X%5BAuthor%5D&cauthor=true&cauthor_uid=22290877), [**Sterling JA**](http://www.ncbi.nlm.nih.gov/pubmed/?term=Sterling JA%5BAuthor%5D&cauthor=true&cauthor_uid=22290877), [**Fan KH**](http://www.ncbi.nlm.nih.gov/pubmed/?term=Fan KH%5BAuthor%5D&cauthor=true&cauthor_uid=22290877). Loss of TGF-β responsiveness in prostate stromal cells alters chemokine levels and facilitates the development of mixed osteoblastic/osteolytic bone lesions. Mol Cancer Res. 2012 Apr;10(4):494-503.

# [**Bruckheimer EM**](http://www.ncbi.nlm.nih.gov/pubmed/?term=Bruckheimer EM%5BAuthor%5D&cauthor=true&cauthor_uid=12242728), [**Kyprianou N**](http://www.ncbi.nlm.nih.gov/pubmed/?term=Kyprianou N%5BAuthor%5D&cauthor=true&cauthor_uid=12242728).Bcl-**2**antagonizes the combined apoptotic effect of transforming growth factor-beta and dihydrotestosterone in**prostate**cancer cells. Prostate. 2002 Oct 1;53(2):133-42.

1. [Elliott RL](http://www.ncbi.nlm.nih.gov/pubmed/?term=Elliott RL%5BAuthor%5D&cauthor=true&cauthor_uid=15774796), [Blobe GC](http://www.ncbi.nlm.nih.gov/pubmed/?term=Blobe GC%5BAuthor%5D&cauthor=true&cauthor_uid=15774796). Role of transforming growth factor Beta in human cancer. J Clin Oncol. 2005 Mar 20;23(9):2078-93.

# [**Nishimori H**](http://www.ncbi.nlm.nih.gov/pubmed/?term=Nishimori H%5BAuthor%5D&cauthor=true&cauthor_uid=22532569), [**Ehata S**](http://www.ncbi.nlm.nih.gov/pubmed/?term=Ehata S%5BAuthor%5D&cauthor=true&cauthor_uid=22532569), [**Suzuki HI**](http://www.ncbi.nlm.nih.gov/pubmed/?term=Suzuki HI%5BAuthor%5D&cauthor=true&cauthor_uid=22532569). Prostate cancer cells and bone stromal cells mutually interact with each other through bone morphogenetic protein-mediated signals. J Biol Chem. 2012 Jun 8;287(24):20037-46.

# [**Breen MJ**](http://www.ncbi.nlm.nih.gov/pubmed/?term=Breen MJ%5BAuthor%5D&cauthor=true&cauthor_uid=23967299), [**Moran DM**](http://www.ncbi.nlm.nih.gov/pubmed/?term=Moran DM%5BAuthor%5D&cauthor=true&cauthor_uid=23967299), [**Liu W**](http://www.ncbi.nlm.nih.gov/pubmed/?term=Liu W%5BAuthor%5D&cauthor=true&cauthor_uid=23967299). Endoglin-mediated suppression of prostate cancer invasion is regulated by activin and bone morphogenetic protein type II receptors PLoS One. 2013 Aug 13;8(8):e72407.

# [**Ye L**](http://www.ncbi.nlm.nih.gov/pubmed/?term=Ye L%5BAuthor%5D&cauthor=true&cauthor_uid=18922975), [**Kynaston H**](http://www.ncbi.nlm.nih.gov/pubmed/?term=Kynaston H%5BAuthor%5D&cauthor=true&cauthor_uid=18922975), [**Jiang WG**](http://www.ncbi.nlm.nih.gov/pubmed/?term=Jiang WG%5BAuthor%5D&cauthor=true&cauthor_uid=18922975). Bone morphogenetic protein-9 induces apoptosis in prostate cancer cells, the role of prostate apoptosis response-4. Mol Cancer Res. 2008 Oct;6(10):1594-606.

# [**Kim IY**](http://www.ncbi.nlm.nih.gov/pubmed/?term=Kim IY%5BAuthor%5D&cauthor=true&cauthor_uid=15354178), [**Lee DH**](http://www.ncbi.nlm.nih.gov/pubmed/?term=Lee DH%5BAuthor%5D&cauthor=true&cauthor_uid=15354178), [**Lee DK**](http://www.ncbi.nlm.nih.gov/pubmed/?term=Lee DK%5BAuthor%5D&cauthor=true&cauthor_uid=15354178). Loss of expression of bone morphogenetic protein receptor type II in human prostate cancer cells. Oncogene. 2004 Oct 7;23(46):7651-9.

# [**Kobayashi A**](http://www.ncbi.nlm.nih.gov/pubmed/?term=Kobayashi A%5BAuthor%5D&cauthor=true&cauthor_uid=22124112), [**Okuda H**](http://www.ncbi.nlm.nih.gov/pubmed/?term=Okuda H%5BAuthor%5D&cauthor=true&cauthor_uid=22124112), [**Xing F**](http://www.ncbi.nlm.nih.gov/pubmed/?term=Xing F%5BAuthor%5D&cauthor=true&cauthor_uid=22124112). Bone morphogenetic protein 7 in dormancy and metastasis of prostate cancer stem-like cells in bone. J Exp Med. 2011 Dec 19;208(13):2641-55.

# [**Yang S**](http://www.ncbi.nlm.nih.gov/pubmed/?term=Yang S%5BAuthor%5D&cauthor=true&cauthor_uid=18172312), [**Pham LK**](http://www.ncbi.nlm.nih.gov/pubmed/?term=Pham LK%5BAuthor%5D&cauthor=true&cauthor_uid=18172312), [**Liao CP**](http://www.ncbi.nlm.nih.gov/pubmed/?term=Liao CP%5BAuthor%5D&cauthor=true&cauthor_uid=18172312). A novel bone morphogenetic protein signaling in heterotypic cell interactions in prostate cancer. Cancer Res. 2008 Jan 1;68(1):198-205.

# [**Patki M**](http://www.ncbi.nlm.nih.gov/pubmed/?term=Patki M%5BAuthor%5D&cauthor=true&cauthor_uid=23426362), [**Chari V**](http://www.ncbi.nlm.nih.gov/pubmed/?term=Chari V%5BAuthor%5D&cauthor=true&cauthor_uid=23426362), [**Sivakumaran S**](http://www.ncbi.nlm.nih.gov/pubmed/?term=Sivakumaran S%5BAuthor%5D&cauthor=true&cauthor_uid=23426362). The ETS domain transcription factor ELK1 directs a critical component of growth signaling by the androgen receptor in prostate cancer cells. J Biol Chem. 2013 Apr 19;288(16):11047-65.

# [**Bartek J**](http://www.ncbi.nlm.nih.gov/pubmed/?term=Bartek J%5BAuthor%5D&cauthor=true&cauthor_uid=24203954), [**Mistrik M**](http://www.ncbi.nlm.nih.gov/pubmed/?term=Mistrik M%5BAuthor%5D&cauthor=true&cauthor_uid=24203954), [**Bartkova J**](http://www.ncbi.nlm.nih.gov/pubmed/?term=Bartkova J%5BAuthor%5D&cauthor=true&cauthor_uid=24203954). Androgen receptor signaling fuels DNA repair and radio resistance in prostate cancer. Cancer Discov. 2013 Nov;3(11):1222-4.

# [**Shiota M**](http://www.ncbi.nlm.nih.gov/pubmed/?term=Shiota M%5BAuthor%5D&cauthor=true&cauthor_uid=23775496), [**Kashiwagi E**](http://www.ncbi.nlm.nih.gov/pubmed/?term=Kashiwagi E%5BAuthor%5D&cauthor=true&cauthor_uid=23775496), [**Yokomizo A**](http://www.ncbi.nlm.nih.gov/pubmed/?term=Yokomizo A%5BAuthor%5D&cauthor=true&cauthor_uid=23775496). Interaction between docetaxel resistance and castration resistance in**prostate**cancer: implications of Twist1, YB-1, and androgen receptor. Prostate. 2013 Sep;73(12):1336-44.

# [**Wen S**](http://www.ncbi.nlm.nih.gov/pubmed/?term=Wen S%5BAuthor%5D&cauthor=true&cauthor_uid=23993415), [**Niu Y**](http://www.ncbi.nlm.nih.gov/pubmed/?term=Niu Y%5BAuthor%5D&cauthor=true&cauthor_uid=23993415), [**Lee SO**](http://www.ncbi.nlm.nih.gov/pubmed/?term=Lee SO%5BAuthor%5D&cauthor=true&cauthor_uid=23993415). Androgen receptor (AR) positive vs negative roles in prostate cancer cell deaths including apoptosis, anoikis, entosis, necrosis and autophagic cell death. Cancer Treat Rev. 2014 Feb;40(1):31-40.

# [**Liu X**](http://www.ncbi.nlm.nih.gov/pubmed/?term=Liu X%5BAuthor%5D&cauthor=true&cauthor_uid=24130878), [**Busby J**](http://www.ncbi.nlm.nih.gov/pubmed/?term=Busby J%5BAuthor%5D&cauthor=true&cauthor_uid=24130878), [**John C**](http://www.ncbi.nlm.nih.gov/pubmed/?term=John C%5BAuthor%5D&cauthor=true&cauthor_uid=24130878). Direct interaction between AR and PAK6 in androgen-stimulated PAK6 activation. PLoS One. 2013 Oct 10;8(10):e77367.

# [**Ray S**](http://www.ncbi.nlm.nih.gov/pubmed/?term=Ray S%5BAuthor%5D&cauthor=true&cauthor_uid=23608168), [**Johnston R**](http://www.ncbi.nlm.nih.gov/pubmed/?term=Johnston R%5BAuthor%5D&cauthor=true&cauthor_uid=23608168), [**Campbell D**](http://www.ncbi.nlm.nih.gov/pubmed/?term=Campbell DC%5BAuthor%5D&cauthor=true&cauthor_uid=23608168). Androgens and estrogens stimulate ribosome biogenesis in prostate and breast cancer cells in receptor dependent manner. Gene. 2013 Aug 15;526(1):46-53.

# [**Wang X**](http://www.ncbi.nlm.nih.gov/pubmed/?term=Wang X%5BAuthor%5D&cauthor=true&cauthor_uid=23536722), [**Lee SO**](http://www.ncbi.nlm.nih.gov/pubmed/?term=Lee SO%5BAuthor%5D&cauthor=true&cauthor_uid=23536722), [**Xia S**](http://www.ncbi.nlm.nih.gov/pubmed/?term=Xia S%5BAuthor%5D&cauthor=true&cauthor_uid=23536722). Endothelial cells enhance prostate cancer metastasis via IL-**6**→androgen receptor→TGF-β→MMP-9 signals. Mol Cancer Ther. 2013 Jun;12(6):1026-37.

# [**Allioli N**](http://www.ncbi.nlm.nih.gov/pubmed/?term=Allioli N%5BAuthor%5D&cauthor=true&cauthor_uid=21656834), [**Vincent S**](http://www.ncbi.nlm.nih.gov/pubmed/?term=Vincent S%5BAuthor%5D&cauthor=true&cauthor_uid=21656834), [**Vlaeminck-Guillem V**](http://www.ncbi.nlm.nih.gov/pubmed/?term=Vlaeminck-Guillem V%5BAuthor%5D&cauthor=true&cauthor_uid=21656834). TM4SF1, a novel primary androgen receptor target gene over-expressed in human**prostate**cancer and involved in cell migration. Prostate. 2011 Aug 1;71(11):1239-50.

# [**Castoria G**](http://www.ncbi.nlm.nih.gov/pubmed/?term=Castoria G%5BAuthor%5D&cauthor=true&cauthor_uid=21359179), [**D'Amato L**](http://www.ncbi.nlm.nih.gov/pubmed/?term=D'Amato L%5BAuthor%5D&cauthor=true&cauthor_uid=21359179), [**Ciociola A**](http://www.ncbi.nlm.nih.gov/pubmed/?term=Ciociola A%5BAuthor%5D&cauthor=true&cauthor_uid=21359179). Androgen-induced cell migration: role of androgen receptor/filamin A association. PLoS One. 2011 Feb 16;6(2):e17218.

# [**Grant CM**](http://www.ncbi.nlm.nih.gov/pubmed/?term=Grant CM%5BAuthor%5D&cauthor=true&cauthor_uid=25346895), [**Kyprianou N**](http://www.ncbi.nlm.nih.gov/pubmed/?term=Kyprianou N%5BAuthor%5D&cauthor=true&cauthor_uid=25346895). Epithelial mesenchymal transition (EMT) in prostate growth and tumor progression. Transl Androl Urol 2013;2(3):202-211.

# [**Cano P**](http://www.ncbi.nlm.nih.gov/pubmed/?term=Cano P%5BAuthor%5D&cauthor=true&cauthor_uid=17234758), [**Godoy A**](http://www.ncbi.nlm.nih.gov/pubmed/?term=Godoy A%5BAuthor%5D&cauthor=true&cauthor_uid=17234758), [**Escamilla R**](http://www.ncbi.nlm.nih.gov/pubmed/?term=Escamilla R%5BAuthor%5D&cauthor=true&cauthor_uid=17234758). Stromal-epithelial cell interactions and androgen receptor-coregulator recruitment is altered in the tissue microenvironment of prostate cancer. Cancer Res. 2007 Jan 15;67(2):511-9.

# [**Izumi K**](http://www.ncbi.nlm.nih.gov/pubmed/?term=Izumi K%5BAuthor%5D&cauthor=true&cauthor_uid=24498558), [**Chang C**](http://www.ncbi.nlm.nih.gov/pubmed/?term=Chang C%5BAuthor%5D&cauthor=true&cauthor_uid=24498558). Targeting inflammatory cytokines-androgen receptor (AR) signaling with ASC-J9® to better battle prostate cancer progression. Oncoimmunology. 2013 Dec 1;2(12):e26853.

# [**Fang LY**](http://www.ncbi.nlm.nih.gov/pubmed/?term=Fang LY%5BAuthor%5D&cauthor=true&cauthor_uid=23878190), [**Izumi K**](http://www.ncbi.nlm.nih.gov/pubmed/?term=Izumi K%5BAuthor%5D&cauthor=true&cauthor_uid=23878190), [**Lai KP**](http://www.ncbi.nlm.nih.gov/pubmed/?term=Lai KP%5BAuthor%5D&cauthor=true&cauthor_uid=23878190). Infiltrating macrophages promote prostate tumorigenesis via modulating androgen receptor-mediated CCL4-STAT3 signaling. Cancer Res. 2013 Sep 15;73(18):5633-46

# [**Liu S**](http://www.ncbi.nlm.nih.gov/pubmed/?term=Liu S%5BAuthor%5D&cauthor=true&cauthor_uid=20571066), [**Qi Y**](http://www.ncbi.nlm.nih.gov/pubmed/?term=Qi Y%5BAuthor%5D&cauthor=true&cauthor_uid=20571066), [**Ge Y**](http://www.ncbi.nlm.nih.gov/pubmed/?term=Ge Y%5BAuthor%5D&cauthor=true&cauthor_uid=20571066). Telomerase as an important target of androgen signaling blockade for prostate cancer treatment. Mol Cancer Ther. 2010 Jul;9(7):2016-25.

# [**Heinlein CA**](http://www.ncbi.nlm.nih.gov/pubmed/?term=Heinlein CA%5BAuthor%5D&cauthor=true&cauthor_uid=15082523), [**Chang C**](http://www.ncbi.nlm.nih.gov/pubmed/?term=Chang C%5BAuthor%5D&cauthor=true&cauthor_uid=15082523). Androgen receptor in prostate cancer. Endocr Rev. 2004 Apr;25(2):276-308.

# [**Nupponen N**](http://www.ncbi.nlm.nih.gov/pubmed/?term=Nupponen N%5BAuthor%5D&cauthor=true&cauthor_uid=10325488), [**Visakorpi T**](http://www.ncbi.nlm.nih.gov/pubmed/?term=Visakorpi T%5BAuthor%5D&cauthor=true&cauthor_uid=10325488). Molecular biology of progression of prostate cancer. Eur Urol. 1999;35(5-6):351-4.

# S [**Melck D**](http://www.ncbi.nlm.nih.gov/pubmed/?term=Melck D%5BAuthor%5D&cauthor=true&cauthor_uid=10614630), [**De Petrocellis L**](http://www.ncbi.nlm.nih.gov/pubmed/?term=De Petrocellis L%5BAuthor%5D&cauthor=true&cauthor_uid=10614630), [**Orlando P**](http://www.ncbi.nlm.nih.gov/pubmed/?term=Orlando P%5BAuthor%5D&cauthor=true&cauthor_uid=10614630). Suppression of nerve growth factor Trk receptors and prolactin receptors by endocannabinoids leads to inhibition of human breast and prostate cancer cell proliferation. Endocrinology. 2000 Jan;141(1):118-26.

1. [Jacobson EM](http://www.ncbi.nlm.nih.gov/pubmed/?term=Jacobson EM%5BAuthor%5D&cauthor=true&cauthor_uid=20846877), [Hugo ER](http://www.ncbi.nlm.nih.gov/pubmed/?term=Hugo ER%5BAuthor%5D&cauthor=true&cauthor_uid=20846877). Unexploited therapies in breast and prostate cancer: blockade of the prolactin receptor. Trends Endocrinol Metab. 2010 Nov;21(11):691-8.

# [**Liao Z**](http://www.ncbi.nlm.nih.gov/pubmed/?term=Liao Z%5BAuthor%5D&cauthor=true&cauthor_uid=21416055), [**Nevalainen MT**](http://www.ncbi.nlm.nih.gov/pubmed/?term=Nevalainen MT%5BAuthor%5D&cauthor=true&cauthor_uid=21416055). Targeting transcription factor Stat5a/b as a therapeutic strategy for prostate cancer. Am J Transl Res. 2011 Feb;3(2):133-8. Epub 2010 Nov 21.

# [**Dagvadorj A**](http://www.ncbi.nlm.nih.gov/pubmed/?term=Dagvadorj A%5BAuthor%5D&cauthor=true&cauthor_uid=17412813), [**Collins S**](http://www.ncbi.nlm.nih.gov/pubmed/?term=Collins S%5BAuthor%5D&cauthor=true&cauthor_uid=17412813), [**Jomain JB**](http://www.ncbi.nlm.nih.gov/pubmed/?term=Jomain JB%5BAuthor%5D&cauthor=true&cauthor_uid=17412813). Autocrine prolactin promotes prostate cancer cell growth via Janus kinase-2-signal transducer and activator of transcription-5a/b signaling pathway. Endocrinology. 2007 Jul;148(7):3089-101.

# [**Chin AI**](http://www.ncbi.nlm.nih.gov/pubmed/?term=Chin AI%5BAuthor%5D&cauthor=true&cauthor_uid=20233880), [**Miyahira AK**](http://www.ncbi.nlm.nih.gov/pubmed/?term=Miyahira AK%5BAuthor%5D&cauthor=true&cauthor_uid=20233880), [**Covarrubias A**](http://www.ncbi.nlm.nih.gov/pubmed/?term=Covarrubias A%5BAuthor%5D&cauthor=true&cauthor_uid=20233880). Toll-like receptor 3-mediated suppression of TRAMP prostate cancer shows the critical role of type I interferons in tumor immune surveillance. Cancer Res. 2010 Apr 1;70(7):2595-603.

# [**Kwon EM**](http://www.ncbi.nlm.nih.gov/pubmed/?term=Kwon EM%5BAuthor%5D&cauthor=true&cauthor_uid=21430300), [**Salinas CA**](http://www.ncbi.nlm.nih.gov/pubmed/?term=Salinas CA%5BAuthor%5D&cauthor=true&cauthor_uid=21430300), [**Kolb S**](http://www.ncbi.nlm.nih.gov/pubmed/?term=Kolb S%5BAuthor%5D&cauthor=true&cauthor_uid=21430300). Genetic polymorphisms in inflammation pathway genes and prostate cancer risk. Cancer Epidemiol Biomarkers Prev. 2011 May;20(5):923-33.

# [**Liu XH**](http://www.ncbi.nlm.nih.gov/pubmed/?term=Liu XH%5BAuthor%5D&cauthor=true&cauthor_uid=11779161), [**Kirschenbaum A**](http://www.ncbi.nlm.nih.gov/pubmed/?term=Kirschenbaum A%5BAuthor%5D&cauthor=true&cauthor_uid=11779161), [**Lu M**](http://www.ncbi.nlm.nih.gov/pubmed/?term=Lu M%5BAuthor%5D&cauthor=true&cauthor_uid=11779161). Prostaglandin E(2) stimulates prostatic intraepithelial neoplasia cell growth through activation of the interleukin-6/GP130/STAT-3 signaling pathway. Biochem Biophys Res Commun. 2002 Jan 11;290(1):249-55.

# [**Lou W**](http://www.ncbi.nlm.nih.gov/pubmed/?term=Lou W%5BAuthor%5D&cauthor=true&cauthor_uid=10639195), [**Ni Z**](http://www.ncbi.nlm.nih.gov/pubmed/?term=Ni Z%5BAuthor%5D&cauthor=true&cauthor_uid=10639195), [**Dyer K**](http://www.ncbi.nlm.nih.gov/pubmed/?term=Dyer K%5BAuthor%5D&cauthor=true&cauthor_uid=10639195). Interleukin-6 induces**prostate**cancer cell growth accompanied by activation of stat3 signaling pathway. Prostate. 2000 Feb 15;42(3):239-42.

# [**Jones KJ**](http://www.ncbi.nlm.nih.gov/pubmed/?term=Jones KJ%5BAuthor%5D&cauthor=true&cauthor_uid=24078461), [**Chetram MA**](http://www.ncbi.nlm.nih.gov/pubmed/?term=Chetram MA%5BAuthor%5D&cauthor=true&cauthor_uid=24078461), [**Bethea DA**](http://www.ncbi.nlm.nih.gov/pubmed/?term=Bethea DA%5BAuthor%5D&cauthor=true&cauthor_uid=24078461). Cysteine (C)-X-C Receptor 4 Regulates NADPH Oxidase-2 During Oxidative Stress in Prostate**Cancer**Cells. Cancer Microenviron. 2013 Sep 28.

# [**Cho KS**](http://www.ncbi.nlm.nih.gov/pubmed/?term=Cho KS%5BAuthor%5D&cauthor=true&cauthor_uid=24137439), [**Yoon SJ**](http://www.ncbi.nlm.nih.gov/pubmed/?term=Yoon SJ%5BAuthor%5D&cauthor=true&cauthor_uid=24137439), [**Lee JY**](http://www.ncbi.nlm.nih.gov/pubmed/?term=Lee JY%5BAuthor%5D&cauthor=true&cauthor_uid=24137439). Inhibition of tumor growth and histopathological changes following treatment with a chemokine receptor CXCR4 antagonist in a prostate cancer xenograft model. Oncol Lett. 2013 Oct;6(4):933-938.

# [**Sun YX**](http://www.ncbi.nlm.nih.gov/pubmed/?term=Sun YX%5BAuthor%5D&cauthor=true&cauthor_uid=12761880), [**Wang J**](http://www.ncbi.nlm.nih.gov/pubmed/?term=Wang J%5BAuthor%5D&cauthor=true&cauthor_uid=12761880), [**Shelburne CE**](http://www.ncbi.nlm.nih.gov/pubmed/?term=Shelburne CE%5BAuthor%5D&cauthor=true&cauthor_uid=12761880). Expression of CXCR4 and CXCL12 (SDF-1) in human prostate cancers (PCa) in vivo. J Cell Biochem. 2003 Jun 1;89(3):462-73.

# [**Wang Q**](http://www.ncbi.nlm.nih.gov/pubmed/?term=Wang Q%5BAuthor%5D&cauthor=true&cauthor_uid=21306303), [**Diao X**](http://www.ncbi.nlm.nih.gov/pubmed/?term=Diao X%5BAuthor%5D&cauthor=true&cauthor_uid=21306303), [**Sun J**](http://www.ncbi.nlm.nih.gov/pubmed/?term=Sun J%5BAuthor%5D&cauthor=true&cauthor_uid=21306303). Regulation of VEGF, MMP-**9**and metastasis by CXCR4 in a prostate cancer cell line. Cell Biol Int. 2011 Sep;35(9):897-904.

# [**Uygur B**](http://www.ncbi.nlm.nih.gov/pubmed/?term=Uygur B%5BAuthor%5D&cauthor=true&cauthor_uid=22074556), [**Wu WS**](http://www.ncbi.nlm.nih.gov/pubmed/?term=Wu WS%5BAuthor%5D&cauthor=true&cauthor_uid=22074556). SLUG promotes prostate cancer cell migration and invasion via CXCR4/CXCL12 axis. Mol Cancer. 2011 Nov 10;10:139.

# [**Engl T**](http://www.ncbi.nlm.nih.gov/pubmed/?term=Engl T%5BAuthor%5D&cauthor=true&cauthor_uid=16756721), [**Relja B**](http://www.ncbi.nlm.nih.gov/pubmed/?term=Relja B%5BAuthor%5D&cauthor=true&cauthor_uid=16756721), [**Marian D**](http://www.ncbi.nlm.nih.gov/pubmed/?term=Marian D%5BAuthor%5D&cauthor=true&cauthor_uid=16756721).CXCR4 chemokine receptor mediates prostate tumor cell adhesion through alpha5 and beta3 integrins. Neoplasia. 2006 Apr;8(4):290-301.

# [**Begley L**](http://www.ncbi.nlm.nih.gov/pubmed/?term=Begley L%5BAuthor%5D&cauthor=true&cauthor_uid=16300481), [**Monteleon C**](http://www.ncbi.nlm.nih.gov/pubmed/?term=Monteleon C%5BAuthor%5D&cauthor=true&cauthor_uid=16300481), [**Shah RB**](http://www.ncbi.nlm.nih.gov/pubmed/?term=Shah RB%5BAuthor%5D&cauthor=true&cauthor_uid=16300481). CXCL12 overexpression and secretion by aging fibroblasts enhance human prostate epithelial proliferation in vitro. Aging Cell. 2005 Dec;4(6):291-8.

# [**de Muga S**](http://www.ncbi.nlm.nih.gov/pubmed/?term=de Muga S%5BAuthor%5D&cauthor=true&cauthor_uid=23321466), [**Hernández S**](http://www.ncbi.nlm.nih.gov/pubmed/?term=Hernández S%5BAuthor%5D&cauthor=true&cauthor_uid=23321466), [**Salido M**](http://www.ncbi.nlm.nih.gov/pubmed/?term=Salido M%5BAuthor%5D&cauthor=true&cauthor_uid=23321466). CXCR4 mRNA overexpression in high grade prostate tumors: lack of association with TMPRSS2-ERG rearrangement. Cancer Biomark. 2012-2013;12(1):21-30.

# [**Wang J**](http://www.ncbi.nlm.nih.gov/pubmed/?term=Wang J%5BAuthor%5D&cauthor=true&cauthor_uid=16005185), [**Wang J**](http://www.ncbi.nlm.nih.gov/pubmed/?term=Wang J%5BAuthor%5D&cauthor=true&cauthor_uid=16005185), [**Sun Y**](http://www.ncbi.nlm.nih.gov/pubmed/?term=Sun Y%5BAuthor%5D&cauthor=true&cauthor_uid=16005185). Diverse signaling pathways through the SDF-1/CXCR4 chemokine axis in prostate cancer cell lines leads to altered patterns of cytokine secretion and angiogenesis. Cell Signal. 2005 Dec;17(12):1578-92.

# [**Wang J**](http://www.ncbi.nlm.nih.gov/pubmed/?term=Wang J%5BAuthor%5D&cauthor=true&cauthor_uid=17210694), [**Wang J**](http://www.ncbi.nlm.nih.gov/pubmed/?term=Wang J%5BAuthor%5D&cauthor=true&cauthor_uid=17210694), [**Dai J**](http://www.ncbi.nlm.nih.gov/pubmed/?term=Dai J%5BAuthor%5D&cauthor=true&cauthor_uid=17210694). A glycolytic mechanism regulating an angiogenic switch in prostate cancer. Cancer Res. 2007 Jan 1;67(1):149-59.

# [**Chinni SR**](http://www.ncbi.nlm.nih.gov/pubmed/?term=Chinni SR%5BAuthor%5D&cauthor=true&cauthor_uid=16114056), [**Sivalogan S**](http://www.ncbi.nlm.nih.gov/pubmed/?term=Sivalogan S%5BAuthor%5D&cauthor=true&cauthor_uid=16114056). CXCL12/CXCR4 signaling activates Akt-**1**and MMP-9 expression in**prostate**cancer cells: the role of bone microenvironment-associated CXCL12. Prostate. 2006 Jan 1;66(1):32-48.

# [**Kaulfuss S**](http://www.ncbi.nlm.nih.gov/pubmed/?term=Kaulfuss S%5BAuthor%5D&cauthor=true&cauthor_uid=19701244), [**von Hardenberg S**](http://www.ncbi.nlm.nih.gov/pubmed/?term=von Hardenberg S%5BAuthor%5D&cauthor=true&cauthor_uid=19701244), [**Schweyer S**](http://www.ncbi.nlm.nih.gov/pubmed/?term=Schweyer S%5BAuthor%5D&cauthor=true&cauthor_uid=19701244). Leupaxin acts as a mediator in prostate carcinoma progression through deregulation of p120catenin expression. Oncogene. 2009 Nov 12;28(45):3971-82.

# [**Kaulfuss S**](http://www.ncbi.nlm.nih.gov/pubmed/?term=Kaulfuss S%5BAuthor%5D&cauthor=true&cauthor_uid=18451096), [**Grzmil M**](http://www.ncbi.nlm.nih.gov/pubmed/?term=Grzmil M%5BAuthor%5D&cauthor=true&cauthor_uid=18451096), [**Hemmerlein B**](http://www.ncbi.nlm.nih.gov/pubmed/?term=Hemmerlein B%5BAuthor%5D&cauthor=true&cauthor_uid=18451096). Leupaxin, a novel coactivator of the androgen receptor, is expressed in prostate cancer and plays a role in adhesion and invasion of prostate carcinoma cells. Mol Endocrinol. 2008 Jul;22(7):1606-21.

# [**Sahu SN**](http://www.ncbi.nlm.nih.gov/pubmed/?term=Sahu SN%5BAuthor%5D&cauthor=true&cauthor_uid=17329398), [**Nunez S**](http://www.ncbi.nlm.nih.gov/pubmed/?term=Nunez S%5BAuthor%5D&cauthor=true&cauthor_uid=17329398), [**Bai G**](http://www.ncbi.nlm.nih.gov/pubmed/?term=Bai G%5BAuthor%5D&cauthor=true&cauthor_uid=17329398). Interaction of Pyk2 and PTP-PEST with leupaxin in prostate cancer cells. Am J Physiol Cell Physiol. 2007 Jun;292(6):C2288-96.

# [**Collard RL**](http://www.ncbi.nlm.nih.gov/pubmed/?term=Collard RL%5BAuthor%5D&cauthor=true&cauthor_uid=16425203), [**Harya NS**](http://www.ncbi.nlm.nih.gov/pubmed/?term=Harya NS%5BAuthor%5D&cauthor=true&cauthor_uid=16425203), [**Monzon FA**](http://www.ncbi.nlm.nih.gov/pubmed/?term=Monzon FA%5BAuthor%5D&cauthor=true&cauthor_uid=16425203). Methylation of the ASC gene promoter is associated with aggressive**prostate**cancer. Prostate. 2006 May 15;66(7):687-95.

# [**Ruiz C**](http://www.ncbi.nlm.nih.gov/pubmed/?term=Ruiz C%5BAuthor%5D&cauthor=true&cauthor_uid=22473923), [**Oeggerli M**](http://www.ncbi.nlm.nih.gov/pubmed/?term=Oeggerli M%5BAuthor%5D&cauthor=true&cauthor_uid=22473923), [**Germann M**](http://www.ncbi.nlm.nih.gov/pubmed/?term=Germann M%5BAuthor%5D&cauthor=true&cauthor_uid=22473923). High NRBP1 expression in**prostate**cancer is linked with poor clinical outcomes and increased cancer cell growth. Prostate. 2012 Nov;72(15):1678-87.

# [**Valencia T**](http://www.ncbi.nlm.nih.gov/pubmed/?term=Valencia T%5BAuthor%5D&cauthor=true&cauthor_uid=22078327), [**Joseph A**](http://www.ncbi.nlm.nih.gov/pubmed/?term=Joseph A%5BAuthor%5D&cauthor=true&cauthor_uid=22078327), [**Kachroo N**](http://www.ncbi.nlm.nih.gov/pubmed/?term=Kachroo N%5BAuthor%5D&cauthor=true&cauthor_uid=22078327). Role and expression of FRS2 and FRS3 in prostate cancer. BMC Cancer. 2011 Nov 11;11:484.

# [**Gamble SC**](http://www.ncbi.nlm.nih.gov/pubmed/?term=Gamble SC%5BAuthor%5D&cauthor=true&cauthor_uid=14968116), [**Odontiadis M**](http://www.ncbi.nlm.nih.gov/pubmed/?term=Odontiadis M%5BAuthor%5D&cauthor=true&cauthor_uid=14968116), [**Waxman J**](http://www.ncbi.nlm.nih.gov/pubmed/?term=Waxman J%5BAuthor%5D&cauthor=true&cauthor_uid=14968116). Androgens target prohibitin to regulate proliferation of prostate cancer cells. Oncogene. 2004 Apr 15;23(17):2996-3004.

# [**Gamble SC**](http://www.ncbi.nlm.nih.gov/pubmed/?term=Gamble SC%5BAuthor%5D&cauthor=true&cauthor_uid=16964284), [**Chotai D**](http://www.ncbi.nlm.nih.gov/pubmed/?term=Chotai D%5BAuthor%5D&cauthor=true&cauthor_uid=16964284), [**Odontiadis M**](http://www.ncbi.nlm.nih.gov/pubmed/?term=Odontiadis M%5BAuthor%5D&cauthor=true&cauthor_uid=16964284). Prohibitin, a protein downregulated by androgens, represses androgen receptor activity. Oncogene. 2007 Mar 15;26(12):1757-68.

# [**Fletcher CE**](http://www.ncbi.nlm.nih.gov/pubmed/?term=Fletcher CE%5BAuthor%5D&cauthor=true&cauthor_uid=22505583), [**Dart DA**](http://www.ncbi.nlm.nih.gov/pubmed/?term=Dart DA%5BAuthor%5D&cauthor=true&cauthor_uid=22505583), [**Sita-Lumsden A**](http://www.ncbi.nlm.nih.gov/pubmed/?term=Sita-Lumsden A%5BAuthor%5D&cauthor=true&cauthor_uid=22505583). Androgen-regulated processing of the oncomir miR-27a, which targets Prohibitin in prostate cancer. Hum Mol Genet. 2012 Jul 15;21(14):3112-27.

# [**Wang S**](http://www.ncbi.nlm.nih.gov/pubmed/?term=Wang S%5BAuthor%5D&cauthor=true&cauthor_uid=21566741), [**Faller DV**](http://www.ncbi.nlm.nih.gov/pubmed/?term=Faller DV%5BAuthor%5D&cauthor=true&cauthor_uid=21566741). Roles of prohibitin in growth control and tumor suppression in human cancers. Transl Oncogenomics. 2008 Feb 10;3:23-37.

# [**Zhu B**](http://www.ncbi.nlm.nih.gov/pubmed/?term=Zhu B%5BAuthor%5D&cauthor=true&cauthor_uid=19725029), [**Zhai J**](http://www.ncbi.nlm.nih.gov/pubmed/?term=Zhai J%5BAuthor%5D&cauthor=true&cauthor_uid=19725029), [**Zhu H**](http://www.ncbi.nlm.nih.gov/pubmed/?term=Zhu H%5BAuthor%5D&cauthor=true&cauthor_uid=19725029).Prohibitin regulates TGF-beta induced apoptosis as a downstream effector of Smad-dependent and -independent signaling. Prostate. 2010 Jan 1;70(1):17-26.

# [**Dai Y**](http://www.ncbi.nlm.nih.gov/pubmed/?term=Dai Y%5BAuthor%5D&cauthor=true&cauthor_uid=22144090), [**Qi L**](http://www.ncbi.nlm.nih.gov/pubmed/?term=Qi L%5BAuthor%5D&cauthor=true&cauthor_uid=22144090), [**Zhang X**](http://www.ncbi.nlm.nih.gov/pubmed/?term=Zhang X%5BAuthor%5D&cauthor=true&cauthor_uid=22144090). CrkI and p130(Cas) complex regulates the migration and invasion of prostate cancer cells. Cell Biochem Funct. 2011 Dec;29(8):625-9.

# [**Lee MS**](http://www.ncbi.nlm.nih.gov/pubmed/?term=Lee MS%5BAuthor%5D&cauthor=true&cauthor_uid=14696093), [**Igawa T**](http://www.ncbi.nlm.nih.gov/pubmed/?term=Igawa T%5BAuthor%5D&cauthor=true&cauthor_uid=14696093), [**Chen SJ**](http://www.ncbi.nlm.nih.gov/pubmed/?term=Chen SJ%5BAuthor%5D&cauthor=true&cauthor_uid=14696093). p66Shc protein is upregulated by steroid hormones in hormone-sensitive cancer cells and in primary prostate carcinomas. Int J Cancer. 2004 Feb 20;108(5):672-8.

# [**Lee MS**](http://www.ncbi.nlm.nih.gov/pubmed/?term=Lee MS%5BAuthor%5D&cauthor=true&cauthor_uid=14990987), [**Igawa T**](http://www.ncbi.nlm.nih.gov/pubmed/?term=Igawa T%5BAuthor%5D&cauthor=true&cauthor_uid=14990987), [**Lin MF**](http://www.ncbi.nlm.nih.gov/pubmed/?term=Lin MF%5BAuthor%5D&cauthor=true&cauthor_uid=14990987). Tyrosine-317 of p52(Shc) mediates androgen-stimulated proliferation signals in human prostate cancer cells. Oncogene. 2004 Apr 15;23(17):3048-58.

# [**Reiss K**](http://www.ncbi.nlm.nih.gov/pubmed/?term=Reiss K%5BAuthor%5D&cauthor=true&cauthor_uid=11313980), [**Wang JY**](http://www.ncbi.nlm.nih.gov/pubmed/?term=Wang JY%5BAuthor%5D&cauthor=true&cauthor_uid=11313980), [**Romano G**](http://www.ncbi.nlm.nih.gov/pubmed/?term=Romano G%5BAuthor%5D&cauthor=true&cauthor_uid=11313980). Mechanisms of regulation of cell adhesion and motility by insulin receptor substrate-1 in prostate cancer cells. Oncogene. 2001 Jan 25;20(4):490-500.

# [**Tahir SA**](http://www.ncbi.nlm.nih.gov/pubmed/?term=Tahir SA%5BAuthor%5D&cauthor=true&cauthor_uid=23302227), [**Yang G**](http://www.ncbi.nlm.nih.gov/pubmed/?term=Yang G%5BAuthor%5D&cauthor=true&cauthor_uid=23302227), [**Goltsov A**](http://www.ncbi.nlm.nih.gov/pubmed/?term=Goltsov A%5BAuthor%5D&cauthor=true&cauthor_uid=23302227). Caveolin-1-LRP6 signaling module stimulates aerobic glycolysis in prostate cancer. Cancer Res. 2013 Mar 15;73(6):1900-11.

# [**Sattler HP**](http://www.ncbi.nlm.nih.gov/pubmed/?term=Sattler HP%5BAuthor%5D&cauthor=true&cauthor_uid=11074522), [**Lensch R**](http://www.ncbi.nlm.nih.gov/pubmed/?term=Lensch R%5BAuthor%5D&cauthor=true&cauthor_uid=11074522), [**Rohde V**](http://www.ncbi.nlm.nih.gov/pubmed/?term=Rohde V%5BAuthor%5D&cauthor=true&cauthor_uid=11074522). Novel amplification unit at chromosome 3q25-q27 in human**prostate** cancer. Prostate. 2000 Nov 1;45(3):207-15.

# [**Ikenberg K**](http://www.ncbi.nlm.nih.gov/pubmed/?term=Ikenberg K%5BAuthor%5D&cauthor=true&cauthor_uid=20591150), [**Fritzsche FR**](http://www.ncbi.nlm.nih.gov/pubmed/?term=Fritzsche FR%5BAuthor%5D&cauthor=true&cauthor_uid=20591150), [**Zuerrer-Haerdi U**](http://www.ncbi.nlm.nih.gov/pubmed/?term=Zuerrer-Haerdi U%5BAuthor%5D&cauthor=true&cauthor_uid=20591150). Insulin-like growth factor II mRNA binding protein 3 (IMP3) is overexpressed in prostate cancer and correlates with higher Gleason scores. BMC Cancer. 2010 Jun 30;10:341.

# [**Zhu ZH**](http://www.ncbi.nlm.nih.gov/pubmed/?term=Zhu ZH%5BAuthor%5D&cauthor=true&cauthor_uid=20651226), [**Yu YP**](http://www.ncbi.nlm.nih.gov/pubmed/?term=Yu YP%5BAuthor%5D&cauthor=true&cauthor_uid=20651226), [**Zheng ZL**](http://www.ncbi.nlm.nih.gov/pubmed/?term=Zheng ZL%5BAuthor%5D&cauthor=true&cauthor_uid=20651226). Integrin alpha 7 interacts with high temperature requirement A2 (HtrA2) to induce prostate cancer cell death. Am J Pathol. 2010 Sep;177(3):1176-86.

# [**Hu XY**](http://www.ncbi.nlm.nih.gov/pubmed/?term=Hu XY%5BAuthor%5D&cauthor=true&cauthor_uid=16764760), [**Chen XC**](http://www.ncbi.nlm.nih.gov/pubmed/?term=Chen XC%5BAuthor%5D&cauthor=true&cauthor_uid=16764760), [**Zhu ZH**](http://www.ncbi.nlm.nih.gov/pubmed/?term=Zhu ZH%5BAuthor%5D&cauthor=true&cauthor_uid=16764760). [Effects of Omi/HtrA2 on expression of anti-apoptotic protein PED/PEA-15 and apoptosis of prostate cancer cell line PC-3]. Ai Zheng. 2006 Jun;25(6):677-82.

# [**van Duin M**](http://www.ncbi.nlm.nih.gov/pubmed/?term=van Duin M%5BAuthor%5D&cauthor=true&cauthor_uid=16130124), [**van Marion R**](http://www.ncbi.nlm.nih.gov/pubmed/?term=van Marion R%5BAuthor%5D&cauthor=true&cauthor_uid=16130124), [**Vissers K**](http://www.ncbi.nlm.nih.gov/pubmed/?term=Vissers K%5BAuthor%5D&cauthor=true&cauthor_uid=16130124). High-resolution array comparative genomic hybridization of chromosome arm 8q: evaluation of genetic progression markers for prostate cancer. Genes Chromosomes Cancer. 2005 Dec;44(4):438-49.

# [**Rahrmann EP**](http://www.ncbi.nlm.nih.gov/pubmed/?term=Rahrmann EP%5BAuthor%5D&cauthor=true&cauthor_uid=19401450), [**Collier LS**](http://www.ncbi.nlm.nih.gov/pubmed/?term=Collier LS%5BAuthor%5D&cauthor=true&cauthor_uid=19401450), [**Knutson TP**](http://www.ncbi.nlm.nih.gov/pubmed/?term=Knutson TP%5BAuthor%5D&cauthor=true&cauthor_uid=19401450). Identification of PDE4D as a proliferation promoting factor in prostate cancer using a Sleeping Beauty transposon-based somatic mutagenesis screen. Cancer Res. 2009 May 15;69(10):4388-97.

# [**Miles FL**](http://www.ncbi.nlm.nih.gov/pubmed/?term=Miles FL%5BAuthor%5D&cauthor=true&cauthor_uid=22228025), [**Tung NS**](http://www.ncbi.nlm.nih.gov/pubmed/?term=Tung NS%5BAuthor%5D&cauthor=true&cauthor_uid=22228025), [**Aguiar AA**](http://www.ncbi.nlm.nih.gov/pubmed/?term=Aguiar AA%5BAuthor%5D&cauthor=true&cauthor_uid=22228025). Increased TGF-β1-mediated suppression of growth and motility in castrate-resistant**prostate**cancer cells is consistent with Smad2/3 signaling. Prostate. 2012 Sep 1;72(12):1339-50.

# [**Yang J**](http://www.ncbi.nlm.nih.gov/pubmed/?term=Yang J%5BAuthor%5D&cauthor=true&cauthor_uid=19276350), [**Wahdan-Alaswad R**](http://www.ncbi.nlm.nih.gov/pubmed/?term=Wahdan-Alaswad R%5BAuthor%5D&cauthor=true&cauthor_uid=19276350), [**Danielpour D**](http://www.ncbi.nlm.nih.gov/pubmed/?term=Danielpour D%5BAuthor%5D&cauthor=true&cauthor_uid=19276350).Critical role of Smad2 in tumor suppression and transforming growth factor-beta-induced apoptosis of prostate epithelial cells. Cancer Res. 2009 Mar 15;69(6):2185-90.

# [**Konishi N**](http://www.ncbi.nlm.nih.gov/pubmed/?term=Konishi N%5BAuthor%5D&cauthor=true&cauthor_uid=1562975), [**Enomoto T**](http://www.ncbi.nlm.nih.gov/pubmed/?term=Enomoto T%5BAuthor%5D&cauthor=true&cauthor_uid=1562975), [**Buzard G**](http://www.ncbi.nlm.nih.gov/pubmed/?term=Buzard G%5BAuthor%5D&cauthor=true&cauthor_uid=1562975).K-ras activation and ras p21 expression in latent prostatic carcinoma in Japanese men. Cancer. 1992 May 1;69(9):2293-9.

# [**Cho NY**](http://www.ncbi.nlm.nih.gov/pubmed/?term=Cho NY%5BAuthor%5D&cauthor=true&cauthor_uid=16721785), [**Choi M**](http://www.ncbi.nlm.nih.gov/pubmed/?term=Choi M%5BAuthor%5D&cauthor=true&cauthor_uid=16721785), [**Kim BH**](http://www.ncbi.nlm.nih.gov/pubmed/?term=Kim BH%5BAuthor%5D&cauthor=true&cauthor_uid=16721785). BRAF and KRAS mutations in prostatic adenocarcinoma. Int J Cancer. 2006 Oct 15;119(8):1858-62.

# [**Xu B**](http://www.ncbi.nlm.nih.gov/pubmed/?term=Xu B%5BAuthor%5D&cauthor=true&cauthor_uid=21197560), [**Niu X**](http://www.ncbi.nlm.nih.gov/pubmed/?term=Niu X%5BAuthor%5D&cauthor=true&cauthor_uid=21197560), [**Zhang X**](http://www.ncbi.nlm.nih.gov/pubmed/?term=Zhang X%5BAuthor%5D&cauthor=true&cauthor_uid=21197560). miR-143 decreases prostate cancer cells proliferation and migration and enhances their sensitivity to docetaxel through suppression of KRAS. Mol Cell Biochem. 2011 Apr;350(1-2):207-13.

# [**Zins K**](http://www.ncbi.nlm.nih.gov/pubmed/?term=Zins K%5BAuthor%5D&cauthor=true&cauthor_uid=24040362), [**Lucas T**](http://www.ncbi.nlm.nih.gov/pubmed/?term=Lucas T%5BAuthor%5D&cauthor=true&cauthor_uid=24040362), [**Reichl P**](http://www.ncbi.nlm.nih.gov/pubmed/?term=Reichl P%5BAuthor%5D&cauthor=true&cauthor_uid=24040362). A Rac1/Cdc42 GTPase-specific small molecule inhibitor suppresses growth of primary human prostate cancer xenografts and prolongs survival in mice. PLoS One. 2013 Sep 11;8(9):e74924.

# [**Trerotola M**](http://www.ncbi.nlm.nih.gov/pubmed/?term=Trerotola M%5BAuthor%5D&cauthor=true&cauthor_uid=23536555), [**Jernigan DL**](http://www.ncbi.nlm.nih.gov/pubmed/?term=Jernigan DL%5BAuthor%5D&cauthor=true&cauthor_uid=23536555), [**Liu Q**](http://www.ncbi.nlm.nih.gov/pubmed/?term=Liu Q%5BAuthor%5D&cauthor=true&cauthor_uid=23536555). Trop-2 promotes prostate cancer metastasis by modulating β(1) integrin functions. Cancer Res. 2013 May 15;73(10):3155-67.

# [**Kobayashi T**](http://www.ncbi.nlm.nih.gov/pubmed/?term=Kobayashi T%5BAuthor%5D&cauthor=true&cauthor_uid=20203103), [**Inoue T**](http://www.ncbi.nlm.nih.gov/pubmed/?term=Inoue T%5BAuthor%5D&cauthor=true&cauthor_uid=20203103), [**Shimizu Y**](http://www.ncbi.nlm.nih.gov/pubmed/?term=Shimizu Y%5BAuthor%5D&cauthor=true&cauthor_uid=20203103). Activation of Rac1 is closely related to androgen-independent cell proliferation of prostate cancer cells both in vitro and in vivo. Mol Endocrinol. 2010 Apr;24(4):722-34.

# [**Lin KT**](http://www.ncbi.nlm.nih.gov/pubmed/?term=Lin KT%5BAuthor%5D&cauthor=true&cauthor_uid=22659453), [**Gong J**](http://www.ncbi.nlm.nih.gov/pubmed/?term=Gong J%5BAuthor%5D&cauthor=true&cauthor_uid=22659453), [**Li CF**](http://www.ncbi.nlm.nih.gov/pubmed/?term=Li CF%5BAuthor%5D&cauthor=true&cauthor_uid=22659453). Vav3-rac1 signaling regulates prostate cancer metastasis with elevated Vav3 expression correlating with prostate cancer progression and posttreatment recurrence. Cancer Res. 2012 Jun 15;72(12):3000-9.

# [**Barthel SR**](http://www.ncbi.nlm.nih.gov/pubmed/?term=Barthel SR%5BAuthor%5D&cauthor=true&cauthor_uid=23149920), [**Hays DL**](http://www.ncbi.nlm.nih.gov/pubmed/?term=Hays DL%5BAuthor%5D&cauthor=true&cauthor_uid=23149920), [**Yazawa EM**](http://www.ncbi.nlm.nih.gov/pubmed/?term=Yazawa EM%5BAuthor%5D&cauthor=true&cauthor_uid=23149920). Definition of molecular determinants of prostate cancer cell bone extravasation. Cancer Res. 2013 Jan 15;73(2):942-52.

# [**Chatterjee M**](http://www.ncbi.nlm.nih.gov/pubmed/?term=Chatterjee M%5BAuthor%5D&cauthor=true&cauthor_uid=21776386), [**Sequeira L**](http://www.ncbi.nlm.nih.gov/pubmed/?term=Sequeira L%5BAuthor%5D&cauthor=true&cauthor_uid=21776386). Individual rac GTPases mediate aspects of prostate cancer cell and bone marrow endothelial cell interactions. J Signal Transduct. 2011;2011:541851.

# [**Cai Y**](http://www.ncbi.nlm.nih.gov/pubmed/?term=Cai Y%5BAuthor%5D&cauthor=true&cauthor_uid=22389719), [**Wang J**](http://www.ncbi.nlm.nih.gov/pubmed/?term=Wang J%5BAuthor%5D&cauthor=true&cauthor_uid=22389719), [**Ren C**](http://www.ncbi.nlm.nih.gov/pubmed/?term=Ren C%5BAuthor%5D&cauthor=true&cauthor_uid=22389719). Frequent heterogeneous missense mutations of GGAP2 in prostate cancer: implications for tumor biology, clonality and mutation analysis. PLoS One. 2012;7(2):e32708.

# [**Zoubeidi A**](http://www.ncbi.nlm.nih.gov/pubmed/?term=Zoubeidi A%5BAuthor%5D&cauthor=true&cauthor_uid=20068069), [**Ettinger S**](http://www.ncbi.nlm.nih.gov/pubmed/?term=Ettinger S%5BAuthor%5D&cauthor=true&cauthor_uid=20068069), [**Beraldi E**](http://www.ncbi.nlm.nih.gov/pubmed/?term=Beraldi E%5BAuthor%5D&cauthor=true&cauthor_uid=20068069). Clusterin facilitates COMMD1 and I-kappaB degradation to enhance NF-kappaB activity in prostate cancer cells. Mol Cancer Res. 2010 Jan;8(1):119-30.

# [**Wu K**](http://www.ncbi.nlm.nih.gov/pubmed/?term=Wu K%5BAuthor%5D&cauthor=true&cauthor_uid=23838317), [**Xie D**](http://www.ncbi.nlm.nih.gov/pubmed/?term=Xie D%5BAuthor%5D&cauthor=true&cauthor_uid=23838317), [**Zou Y**](http://www.ncbi.nlm.nih.gov/pubmed/?term=Zou Y%5BAuthor%5D&cauthor=true&cauthor_uid=23838317). The mechanism of DAB2IP in chemoresistance of prostate cancer cells. Clin Cancer Res. 2013 Sep 1;19(17):4740-9.

# [**Yu L**](http://www.ncbi.nlm.nih.gov/pubmed/?term=Yu L%5BAuthor%5D&cauthor=true&cauthor_uid=23308052), [**Tumati V**](http://www.ncbi.nlm.nih.gov/pubmed/?term=Tumati V%5BAuthor%5D&cauthor=true&cauthor_uid=23308052), [**Tseng SF**](http://www.ncbi.nlm.nih.gov/pubmed/?term=Tseng SF%5BAuthor%5D&cauthor=true&cauthor_uid=23308052). DAB2IP regulates autophagy in prostate cancer in response to combined treatment of radiation and a DNA-PKcs inhibitor. Neoplasia. 2012 Dec;14(12):1203-12.

# [**Kong Z**](http://www.ncbi.nlm.nih.gov/pubmed/?term=Kong Z%5BAuthor%5D&cauthor=true&cauthor_uid=20332235), [**Xie D**](http://www.ncbi.nlm.nih.gov/pubmed/?term=Xie D%5BAuthor%5D&cauthor=true&cauthor_uid=20332235), [**Boike T**](http://www.ncbi.nlm.nih.gov/pubmed/?term=Boike T%5BAuthor%5D&cauthor=true&cauthor_uid=20332235).Downregulation of human DAB2IP gene expression in prostate cancer cells results in resistance to ionizing radiation. Cancer Res. 2010 Apr 1;70(7):2829-39.

# [**Min J**](http://www.ncbi.nlm.nih.gov/pubmed/?term=Min J%5BAuthor%5D&cauthor=true&cauthor_uid=20154697), [**Zaslavsky A**](http://www.ncbi.nlm.nih.gov/pubmed/?term=Zaslavsky A%5BAuthor%5D&cauthor=true&cauthor_uid=20154697), [**Fedele G**](http://www.ncbi.nlm.nih.gov/pubmed/?term=Fedele G%5BAuthor%5D&cauthor=true&cauthor_uid=20154697). An oncogene-tumor suppressor cascade drives metastatic prostate cancer by coordinately activating Ras and nuclear factor-kappaB. Nat Med. 2010 Mar;16(3):286-94.

# [**Chen H**](http://www.ncbi.nlm.nih.gov/pubmed/?term=Chen H%5BAuthor%5D&cauthor=true&cauthor_uid=15817459), [**Tu SW**](http://www.ncbi.nlm.nih.gov/pubmed/?term=Tu SW%5BAuthor%5D&cauthor=true&cauthor_uid=15817459), [**Hsieh JT**](http://www.ncbi.nlm.nih.gov/pubmed/?term=Hsieh JT%5BAuthor%5D&cauthor=true&cauthor_uid=15817459). Down-regulation of human DAB2IP gene expression mediated by polycomb Ezh2 complex and histone deacetylase in prostate cancer. J Biol Chem. 2005 Jun 10;280(23):22437-44.

# [**Xie D**](http://www.ncbi.nlm.nih.gov/pubmed/?term=Xie D%5BAuthor%5D&cauthor=true&cauthor_uid=20080667), [**Gore C**](http://www.ncbi.nlm.nih.gov/pubmed/?term=Gore C%5BAuthor%5D&cauthor=true&cauthor_uid=20080667), [**Liu J**](http://www.ncbi.nlm.nih.gov/pubmed/?term=Liu J%5BAuthor%5D&cauthor=true&cauthor_uid=20080667). Role of DAB2IP in modulating epithelial-to-mesenchymal transition and prostate cancer metastasis. Proc Natl Acad Sci U S A. 2010 Feb 9;107(6):2485-90.

# [**Scaggiante B**](http://www.ncbi.nlm.nih.gov/pubmed/?term=Scaggiante B%5BAuthor%5D&cauthor=true&cauthor_uid=22095224), [**Dapas B**](http://www.ncbi.nlm.nih.gov/pubmed/?term=Dapas B%5BAuthor%5D&cauthor=true&cauthor_uid=22095224), [**Bonin S**](http://www.ncbi.nlm.nih.gov/pubmed/?term=Bonin S%5BAuthor%5D&cauthor=true&cauthor_uid=22095224). Dissecting the expression of EEF1A1/2 genes in human prostate cancer cells: the potential of EEF1A2 as a hallmark for prostate transformation and progression. Br J Cancer. 2012 Jan 3;106(1):166-73.

# [**Ekman M**](http://www.ncbi.nlm.nih.gov/pubmed/?term=Ekman M%5BAuthor%5D&cauthor=true&cauthor_uid=22496417), [**Mu Y**](http://www.ncbi.nlm.nih.gov/pubmed/?term=Mu Y%5BAuthor%5D&cauthor=true&cauthor_uid=22496417), [**Lee SY**](http://www.ncbi.nlm.nih.gov/pubmed/?term=Lee SY%5BAuthor%5D&cauthor=true&cauthor_uid=22496417). APC and Smad7 link TGFβ type I receptors to the microtubule system to promote cell migration. Mol Biol Cell. 2012 Jun;23(11):2109-21.

# [**Walker L**](http://www.ncbi.nlm.nih.gov/pubmed/?term=Walker L%5BAuthor%5D&cauthor=true&cauthor_uid=22678424), [**Millena AC**](http://www.ncbi.nlm.nih.gov/pubmed/?term=Millena AC%5BAuthor%5D&cauthor=true&cauthor_uid=22678424), [**Strong N**](http://www.ncbi.nlm.nih.gov/pubmed/?term=Strong N%5BAuthor%5D&cauthor=true&cauthor_uid=22678424). Expression of TGFβ3 and its effects on migratory and invasive behavior of prostate cancer cells: involvement of PI3-kinase/AKT signaling pathway. Clin Exp Metastasis. 2013 Jan;30(1):13-23.

# [**Bokobza SM**](http://www.ncbi.nlm.nih.gov/pubmed/?term=Bokobza SM%5BAuthor%5D&cauthor=true&cauthor_uid=21042764), [**Ye L**](http://www.ncbi.nlm.nih.gov/pubmed/?term=Ye L%5BAuthor%5D&cauthor=true&cauthor_uid=21042764), [**Kynaston HG**](http://www.ncbi.nlm.nih.gov/pubmed/?term=Kynaston HG%5BAuthor%5D&cauthor=true&cauthor_uid=21042764). Growth and differentiation factor-9 promotes adhesive and motile capacity of prostate cancer cells by up-regulating FAK and Paxillin via Smad dependent pathway. Oncol Rep. 2010 Dec;24(6):1653-9.

# [**Kang HY**](http://www.ncbi.nlm.nih.gov/pubmed/?term=Kang HY%5BAuthor%5D&cauthor=true&cauthor_uid=19257827), [**Huang HY**](http://www.ncbi.nlm.nih.gov/pubmed/?term=Huang HY%5BAuthor%5D&cauthor=true&cauthor_uid=19257827), [**Hsieh CY**](http://www.ncbi.nlm.nih.gov/pubmed/?term=Hsieh CY%5BAuthor%5D&cauthor=true&cauthor_uid=19257827). Activin A enhances prostate cancer cell migration through activation of androgen receptor and is overexpressed in metastatic prostate cancer. J Bone Miner Res. 2009 Jul;24(7):1180-93.

# [**Lu S**](http://www.ncbi.nlm.nih.gov/pubmed/?term=Lu S%5BAuthor%5D&cauthor=true&cauthor_uid=17908958), [**Lee J**](http://www.ncbi.nlm.nih.gov/pubmed/?term=Lee J%5BAuthor%5D&cauthor=true&cauthor_uid=17908958), [**Revelo M**](http://www.ncbi.nlm.nih.gov/pubmed/?term=Revelo M%5BAuthor%5D&cauthor=true&cauthor_uid=17908958). Smad3 is overexpressed in advanced human prostate cancer and necessary for progressive growth of prostate cancer cells in nude mice. Clin Cancer Res. 2007 Oct 1;13(19):5692-702.

1. [Friedlander TW](http://www.ncbi.nlm.nih.gov/pubmed/?term=Friedlander TW%5BAuthor%5D&cauthor=true&cauthor_uid=22158653), [Roy R](http://www.ncbi.nlm.nih.gov/pubmed/?term=Roy R%5BAuthor%5D&cauthor=true&cauthor_uid=22158653), [Tomlins SA](http://www.ncbi.nlm.nih.gov/pubmed/?term=Tomlins SA%5BAuthor%5D&cauthor=true&cauthor_uid=22158653). Common structural and epigenetic changes in the genome of castration-resistant prostate cancer. Cancer Res. 2012 Feb 1;72(3):616-25.

# [**Bettendorf O**](http://www.ncbi.nlm.nih.gov/pubmed/?term=Bettendorf O%5BAuthor%5D&cauthor=true&cauthor_uid=18383208), [**Schmidt H**](http://www.ncbi.nlm.nih.gov/pubmed/?term=Schmidt H%5BAuthor%5D&cauthor=true&cauthor_uid=18383208), [**Staebler A**](http://www.ncbi.nlm.nih.gov/pubmed/?term=Staebler A%5BAuthor%5D&cauthor=true&cauthor_uid=18383208). Chromosomal imbalances, loss of heterozygosity, and immunohistochemical expression of TP53, RB1, and PTEN in intraductal cancer, intraepithelial neoplasia, and invasive adenocarcinoma of the prostate. Genes Chromosomes Cancer. 2008 Jul;47(7):565-72.

# [**Sharma A**](http://www.ncbi.nlm.nih.gov/pubmed/?term=Sharma A%5BAuthor%5D&cauthor=true&cauthor_uid=21099110), [**Yeow WS**](http://www.ncbi.nlm.nih.gov/pubmed/?term=Yeow WS%5BAuthor%5D&cauthor=true&cauthor_uid=21099110), [**Ertel A**](http://www.ncbi.nlm.nih.gov/pubmed/?term=Ertel A%5BAuthor%5D&cauthor=true&cauthor_uid=21099110). The retinoblastoma tumor suppressor controls androgen signaling and human prostate cancer progression. J Clin Invest. 2010 Dec;120(12):4478-92.

# [**Teng Y**](http://www.ncbi.nlm.nih.gov/pubmed/?term=Teng Y%5BAuthor%5D&cauthor=true&cauthor_uid=23677069), [**Ghoshal P**](http://www.ncbi.nlm.nih.gov/pubmed/?term=Ghoshal P%5BAuthor%5D&cauthor=true&cauthor_uid=23677069), [**Ngoka L**](http://www.ncbi.nlm.nih.gov/pubmed/?term=Ngoka L%5BAuthor%5D&cauthor=true&cauthor_uid=23677069). Critical role of the WASF3 gene in JAK2/STAT3 regulation of cancer cell motility. Carcinogenesis. 2013 Sep;34(9):1994-9.

# [**Abdulghani J**](http://www.ncbi.nlm.nih.gov/pubmed/?term=Abdulghani J%5BAuthor%5D&cauthor=true&cauthor_uid=18483213), [**Gu L**](http://www.ncbi.nlm.nih.gov/pubmed/?term=Gu L%5BAuthor%5D&cauthor=true&cauthor_uid=18483213), [**Dagvadorj A**](http://www.ncbi.nlm.nih.gov/pubmed/?term=Dagvadorj A%5BAuthor%5D&cauthor=true&cauthor_uid=18483213). Stat3 promotes metastatic progression of prostate cancer. Am J Pathol. 2008 Jun;172(6):1717-28.

# [**Azare J**](http://www.ncbi.nlm.nih.gov/pubmed/?term=Azare J%5BAuthor%5D&cauthor=true&cauthor_uid=17438134), [**Leslie K**](http://www.ncbi.nlm.nih.gov/pubmed/?term=Leslie K%5BAuthor%5D&cauthor=true&cauthor_uid=17438134), [**Al-Ahmadie H**](http://www.ncbi.nlm.nih.gov/pubmed/?term=Al-Ahmadie H%5BAuthor%5D&cauthor=true&cauthor_uid=17438134). Constitutively activated Stat3 induces tumorigenesis and enhances cell motility of prostate epithelial cells through integrin beta 6. Mol Cell Biol. 2007 Jun;27(12):4444-53.

# [**Kwon GT**](http://www.ncbi.nlm.nih.gov/pubmed/?term=Kwon GT%5BAuthor%5D&cauthor=true&cauthor_uid=21497499), [**Jung JI**](http://www.ncbi.nlm.nih.gov/pubmed/?term=Jung JI%5BAuthor%5D&cauthor=true&cauthor_uid=21497499), [**Song HR**](http://www.ncbi.nlm.nih.gov/pubmed/?term=Song HR%5BAuthor%5D&cauthor=true&cauthor_uid=21497499). Piceatannol inhibits migration and invasion of prostate cancer cells: possible mediation by decreased interleukin-6 signaling. J Nutr Biochem. 2012 Mar;23(3):228-38.

# [**Sekine Y**](http://www.ncbi.nlm.nih.gov/pubmed/?term=Sekine Y%5BAuthor%5D&cauthor=true&cauthor_uid=20979115), [**Suzuki K**](http://www.ncbi.nlm.nih.gov/pubmed/?term=Suzuki K%5BAuthor%5D&cauthor=true&cauthor_uid=20979115), [**Remaley AT**](http://www.ncbi.nlm.nih.gov/pubmed/?term=Remaley AT%5BAuthor%5D&cauthor=true&cauthor_uid=20979115). HDL and sphingosine-1-phosphate activate stat3 in**prostate**cancer DU145 cells via ERK1/2 and S1P receptors, and promote cell migration and invasion. Prostate. 2011 May 15;71(7):690-9.

# [**Cho KH**](http://www.ncbi.nlm.nih.gov/pubmed/?term=Cho KH%5BAuthor%5D&cauthor=true&cauthor_uid=23623921), [**Jeong KJ**](http://www.ncbi.nlm.nih.gov/pubmed/?term=Jeong KJ%5BAuthor%5D&cauthor=true&cauthor_uid=23623921), [**Shin SC**](http://www.ncbi.nlm.nih.gov/pubmed/?term=Shin SC%5BAuthor%5D&cauthor=true&cauthor_uid=23623921). STAT3 mediates TGF-β**1**-induced TWIST1 expression and prostate cancer invasion. Cancer Lett. 2013 Aug 9;336(1):167-73.

# [**Singh N**](http://www.ncbi.nlm.nih.gov/pubmed/?term=Singh N%5BAuthor%5D&cauthor=true&cauthor_uid=23134344), [**Hussain S**](http://www.ncbi.nlm.nih.gov/pubmed/?term=Hussain S%5BAuthor%5D&cauthor=true&cauthor_uid=23134344). Overexpression of signal transducer and activator of transcription (STAT-3 and STAT-5) transcription factors and alteration of suppressor of cytokine signaling (SOCS-1) protein in prostate cancer. J Recept Signal Transduct Res. 2012 Dec;32(6):321-7.

# [**Jimin Shin**](http://www.ncbi.nlm.nih.gov/pubmed/?term=Shin J%5Bauth%5D), [**Hyo-Jeong Lee**](http://www.ncbi.nlm.nih.gov/pubmed/?term=Lee HJ%5Bauth%5D), [**Deok-Beom Jung**](http://www.ncbi.nlm.nih.gov/pubmed/?term=Jung DB%5Bauth%5D). Suppression of STAT3 and HIF-1 Alpha Mediates Anti-Angiogenic Activity of Betulinic Acid in Hypoxic PC-3 Prostate Cancer Cells. PLoS One. 2011;6(6):e21492.

# [**Reddy KR**](http://www.ncbi.nlm.nih.gov/pubmed/?term=Reddy KR%5BAuthor%5D&cauthor=true&cauthor_uid=21480310), [**Guan Y**](http://www.ncbi.nlm.nih.gov/pubmed/?term=Guan Y%5BAuthor%5D&cauthor=true&cauthor_uid=21480310), [**Qin G**](http://www.ncbi.nlm.nih.gov/pubmed/?term=Qin G%5BAuthor%5D&cauthor=true&cauthor_uid=21480310). Combined treatment targeting HIF-1α and Stat3 is a potent strategy for**prostate**cancer therapy. Prostate. 2011 Dec;71(16):1796-809.

# [**Sun M**](http://www.ncbi.nlm.nih.gov/pubmed/?term=Sun M%5BAuthor%5D&cauthor=true&cauthor_uid=21538419), [**Liu C**](http://www.ncbi.nlm.nih.gov/pubmed/?term=Liu C%5BAuthor%5D&cauthor=true&cauthor_uid=21538419), [**Nadiminty N**](http://www.ncbi.nlm.nih.gov/pubmed/?term=Nadiminty N%5BAuthor%5D&cauthor=true&cauthor_uid=21538419). Inhibition of Stat3 activation by sanguinarine suppresses**prostate**cancer cell growth and invasion. Prostate. 2012 Jan;72(1):82-9.

# [**Spiotto MT**](http://www.ncbi.nlm.nih.gov/pubmed/?term=Spiotto MT%5BAuthor%5D&cauthor=true&cauthor_uid=10617865), [**Chung TD**](http://www.ncbi.nlm.nih.gov/pubmed/?term=Chung TD%5BAuthor%5D&cauthor=true&cauthor_uid=10617865). STAT3 mediates IL-6-induced growth inhibition in the human**prostate**cancer cell line LNCaP. Prostate. 2000 Feb 1;42(2):88-98.

# [**Chau MN**](http://www.ncbi.nlm.nih.gov/pubmed/?term=Chau MN%5BAuthor%5D&cauthor=true&cauthor_uid=17615260), [**El Touny LH**](http://www.ncbi.nlm.nih.gov/pubmed/?term=El Touny LH%5BAuthor%5D&cauthor=true&cauthor_uid=17615260), [**Jagadeesh S**](http://www.ncbi.nlm.nih.gov/pubmed/?term=Jagadeesh S%5BAuthor%5D&cauthor=true&cauthor_uid=17615260). Physiologically achievable concentrations of genistein enhance telomerase activity in prostate cancer cells via the activation of STAT3. Carcinogenesis. 2007 Nov;28(11):2282-90.

# [**Verone AR**](http://www.ncbi.nlm.nih.gov/pubmed/?term=Verone AR%5BAuthor%5D&cauthor=true&cauthor_uid=23576568), [**Duncan K**](http://www.ncbi.nlm.nih.gov/pubmed/?term=Duncan K%5BAuthor%5D&cauthor=true&cauthor_uid=23576568), [**Godoy A**](http://www.ncbi.nlm.nih.gov/pubmed/?term=Godoy A%5BAuthor%5D&cauthor=true&cauthor_uid=23576568). Androgen-responsive serum response factor target genes regulate prostate cancer cell migration. Carcinogenesis. 2013 Aug;34(8):1737-46.

# [**Lu H**](http://www.ncbi.nlm.nih.gov/pubmed/?term=Lu H%5BAuthor%5D&cauthor=true&cauthor_uid=21969818), [**Liu P**](http://www.ncbi.nlm.nih.gov/pubmed/?term=Liu P%5BAuthor%5D&cauthor=true&cauthor_uid=21969818), [**Pan Y**](http://www.ncbi.nlm.nih.gov/pubmed/?term=Pan Y%5BAuthor%5D&cauthor=true&cauthor_uid=21969818). Inhibition of cyclin-dependent kinase phosphorylation of FOXO1 and prostate cancer cell growth by a peptide derived from FOXO1. Neoplasia. 2011 Sep;13(9):854-63.

# [**Zhang H**](http://www.ncbi.nlm.nih.gov/pubmed/?term=Zhang H%5BAuthor%5D&cauthor=true&cauthor_uid=21505104), [**Pan Y**](http://www.ncbi.nlm.nih.gov/pubmed/?term=Pan Y%5BAuthor%5D&cauthor=true&cauthor_uid=21505104), [**Zheng.**](http://www.ncbi.nlm.nih.gov/pubmed/?term=Zheng L%5BAuthor%5D&cauthor=true&cauthor_uid=21505104)FOXO1 inhibits Runx2 transcriptional activity and prostate cancer cell migration and invasion. Cancer Res. 2011 May 1;71(9):3257-67.

# [**Liu P**](http://www.ncbi.nlm.nih.gov/pubmed/?term=Liu P%5BAuthor%5D&cauthor=true&cauthor_uid=18408765), [**Kao TP**](http://www.ncbi.nlm.nih.gov/pubmed/?term=Kao TP%5BAuthor%5D&cauthor=true&cauthor_uid=18408765), [**Huang H**](http://www.ncbi.nlm.nih.gov/pubmed/?term=Huang H%5BAuthor%5D&cauthor=true&cauthor_uid=18408765). CDK1 promotes cell proliferation and survival via phosphorylation and inhibition of FOXO1 transcription factor. Oncogene. 2008 Aug 7;27(34):4733-44.

# [**Brett A**](http://www.ncbi.nlm.nih.gov/pubmed/?term=Brett A%5BAuthor%5D&cauthor=true&cauthor_uid=23298185), [**Pandey S**](http://www.ncbi.nlm.nih.gov/pubmed/?term=Pandey S%5BAuthor%5D&cauthor=true&cauthor_uid=23298185), [**Fraizer G**](http://www.ncbi.nlm.nih.gov/pubmed/?term=Fraizer G%5BAuthor%5D&cauthor=true&cauthor_uid=23298185). The Wilms' tumor gene (WT1) regulates E-cadherin expression and migration of prostate cancer cells. Mol Cancer. 2013 Jan 8;12:3.

# [**Hanson J**](http://www.ncbi.nlm.nih.gov/pubmed/?term=Hanson J%5BAuthor%5D&cauthor=true&cauthor_uid=17127464), [**Gorman J**](http://www.ncbi.nlm.nih.gov/pubmed/?term=Gorman J%5BAuthor%5D&cauthor=true&cauthor_uid=17127464), [**Reese J**](http://www.ncbi.nlm.nih.gov/pubmed/?term=Reese J%5BAuthor%5D&cauthor=true&cauthor_uid=17127464). Regulation of vascular endothelial growth factor, VEGF, gene promoter by the tumor suppressor, WT1. Front Biosci. 2007 Jan 1;12:2279-90.

# [**Fraizer G**](http://www.ncbi.nlm.nih.gov/pubmed/?term=Fraizer G%5BAuthor%5D&cauthor=true&cauthor_uid=14767530), [**Leahy R**](http://www.ncbi.nlm.nih.gov/pubmed/?term=Leahy R%5BAuthor%5D&cauthor=true&cauthor_uid=14767530), [**Priyadarshini S**](http://www.ncbi.nlm.nih.gov/pubmed/?term=Priyadarshini S%5BAuthor%5D&cauthor=true&cauthor_uid=14767530). Suppression of prostate tumor cell growth in vivo by WT1, the Wilms' tumor suppressor gene. Int J Oncol. 2004 Mar;24(3):461-71.

# [**Dong Q**](http://www.ncbi.nlm.nih.gov/pubmed/?term=Dong Q%5BAuthor%5D&cauthor=true&cauthor_uid=20418948), [**Meng P**](http://www.ncbi.nlm.nih.gov/pubmed/?term=Meng P%5BAuthor%5D&cauthor=true&cauthor_uid=20418948), [**Wang T**](http://www.ncbi.nlm.nih.gov/pubmed/?term=Wang T%5BAuthor%5D&cauthor=true&cauthor_uid=20418948). MicroRNA let-7a inhibits proliferation of human prostate cancer cells in vitro and in vivo by targeting E2F2 and CCND2. PLoS One. 2010 Apr 14;5(4):e10147.

# [**Browne G**](http://www.ncbi.nlm.nih.gov/pubmed/?term=Browne G%5BAuthor%5D&cauthor=true&cauthor_uid=23073173), [**Nesbitt H**](http://www.ncbi.nlm.nih.gov/pubmed/?term=Nesbitt H%5BAuthor%5D&cauthor=true&cauthor_uid=23073173), [**Ming L**](http://www.ncbi.nlm.nih.gov/pubmed/?term=Ming L%5BAuthor%5D&cauthor=true&cauthor_uid=23073173). Bicalutamide-induced hypoxia potentiates RUNX2-mediated Bcl-2 expression resulting in apoptosis resistance. Br J Cancer. 2012 Nov 6;107(10):1714-21.

# [**Gupta A**](http://www.ncbi.nlm.nih.gov/pubmed/?term=Gupta A%5BAuthor%5D&cauthor=true&cauthor_uid=22966907), [**Cao W**](http://www.ncbi.nlm.nih.gov/pubmed/?term=Cao W%5BAuthor%5D&cauthor=true&cauthor_uid=22966907), [**Chellaiah MA**](http://www.ncbi.nlm.nih.gov/pubmed/?term=Chellaiah MA%5BAuthor%5D&cauthor=true&cauthor_uid=22966907). Integrin αvβ3 and CD44 pathways in metastatic prostate cancer cells support osteoclastogenesis via a Runx2/Smad 5/receptor activator of NF-κB ligand signaling axis. Mol Cancer. 2012 Sep 11;11:66.

# [**Zhang H**](http://www.ncbi.nlm.nih.gov/pubmed/?term=Zhang H%5BAuthor%5D&cauthor=true&cauthor_uid=21505104), [**Pan Y**](http://www.ncbi.nlm.nih.gov/pubmed/?term=Pan Y%5BAuthor%5D&cauthor=true&cauthor_uid=21505104), [**Zheng L**](http://www.ncbi.nlm.nih.gov/pubmed/?term=Zheng L%5BAuthor%5D&cauthor=true&cauthor_uid=21505104).FOXO1 inhibits Runx2 transcriptional activity and prostate cancer cell migration and invasion. Cancer Res. 2011 May 1;71(9):3257-67.

# [**Pratap J**](http://www.ncbi.nlm.nih.gov/pubmed/?term=Pratap J%5BAuthor%5D&cauthor=true&cauthor_uid=16166639), [**Javed A**](http://www.ncbi.nlm.nih.gov/pubmed/?term=Javed A%5BAuthor%5D&cauthor=true&cauthor_uid=16166639), [**Languino LR**](http://www.ncbi.nlm.nih.gov/pubmed/?term=Languino LR%5BAuthor%5D&cauthor=true&cauthor_uid=16166639). The Runx2 osteogenic transcription factor regulates matrix metalloproteinase 9 in bone metastatic cancer cells and controls cell invasion. Mol Cell Biol. 2005 Oct;25(19):8581-91.

# [**van der Deen M**](http://www.ncbi.nlm.nih.gov/pubmed/?term=van der Deen M%5BAuthor%5D&cauthor=true&cauthor_uid=20082326), [**Akech J**](http://www.ncbi.nlm.nih.gov/pubmed/?term=Akech J%5BAuthor%5D&cauthor=true&cauthor_uid=20082326), [**Wang T**](http://www.ncbi.nlm.nih.gov/pubmed/?term=Wang T%5BAuthor%5D&cauthor=true&cauthor_uid=20082326). The cancer-related Runx2 protein enhances cell growth and responses to androgen and TGFbeta in prostate cancer cells. J Cell Biochem. 2010 Mar 1;109(4):828-37.

# [**Lewis H**](http://www.ncbi.nlm.nih.gov/pubmed/?term=Lewis H%5BAuthor%5D&cauthor=true&cauthor_uid=24200968), [**Lance R**](http://www.ncbi.nlm.nih.gov/pubmed/?term=Lance R%5BAuthor%5D&cauthor=true&cauthor_uid=24200968), [**Troyer D**](http://www.ncbi.nlm.nih.gov/pubmed/?term=Troyer D%5BAuthor%5D&cauthor=true&cauthor_uid=24200968). miR-888 is an expressed prostatic secretions-derived microRNA that promotes prostate cell growth and migration. Cell Cycle. 2013 Nov 7;13(2).

# [**Aitchison AA**](http://www.ncbi.nlm.nih.gov/pubmed/?term=Aitchison AA%5BAuthor%5D&cauthor=true&cauthor_uid=18213629), [**Veerakumarasivam A**](http://www.ncbi.nlm.nih.gov/pubmed/?term=Veerakumarasivam A%5BAuthor%5D&cauthor=true&cauthor_uid=18213629), [**Vias M**](http://www.ncbi.nlm.nih.gov/pubmed/?term=Vias M%5BAuthor%5D&cauthor=true&cauthor_uid=18213629). Promoter methylation correlates with reduced Smad4 expression in advanced**prostate**cancer. Prostate. 2008 May 1;68(6):661-74.

# [**Ding Z**](http://www.ncbi.nlm.nih.gov/pubmed/?term=Ding Z%5BAuthor%5D&cauthor=true&cauthor_uid=21289624), [**Wu CJ**](http://www.ncbi.nlm.nih.gov/pubmed/?term=Wu CJ%5BAuthor%5D&cauthor=true&cauthor_uid=21289624), [**Chu GC**](http://www.ncbi.nlm.nih.gov/pubmed/?term=Chu GC%5BAuthor%5D&cauthor=true&cauthor_uid=21289624). SMAD4-dependent barrier constrains prostate cancer growth and metastatic progression. Nature. 2011 Feb 10;470(7333):269-73.

# [**Wu KJ**](http://www.ncbi.nlm.nih.gov/pubmed/?term=Wu KJ%5BAuthor%5D&cauthor=true&cauthor_uid=19288748), [**Zhang D**](http://www.ncbi.nlm.nih.gov/pubmed/?term=Zhang D%5BAuthor%5D&cauthor=true&cauthor_uid=19288748), [**Zhu GF**](http://www.ncbi.nlm.nih.gov/pubmed/?term=Zhu GF%5BAuthor%5D&cauthor=true&cauthor_uid=19288748) [Expression of Smad4 in prostate cancer LNCaP and ARCaP cell lines with different metastatic potentials and its significance]. Zhonghua Nan Ke Xue. 2009 Jan;15(1):41-4.

# [**Khanna A**](http://www.ncbi.nlm.nih.gov/pubmed/?term=Khanna A%5BAuthor%5D&cauthor=true&cauthor_uid=24072747), [**Kauko O**](http://www.ncbi.nlm.nih.gov/pubmed/?term=Kauko O%5BAuthor%5D&cauthor=true&cauthor_uid=24072747), [**Böckelman C**](http://www.ncbi.nlm.nih.gov/pubmed/?term=Böckelman C%5BAuthor%5D&cauthor=true&cauthor_uid=24072747).Chk1 targeting reactivates PP2A tumor suppressor activity in**cancer**cells. Cancer Res. 2013 Nov 26.

# [**Aimola P**](http://www.ncbi.nlm.nih.gov/pubmed/?term=Aimola P%5BAuthor%5D&cauthor=true&cauthor_uid=22448262), [**Carmignani M**](http://www.ncbi.nlm.nih.gov/pubmed/?term=Carmignani M%5BAuthor%5D&cauthor=true&cauthor_uid=22448262), [**Volpe AR**](http://www.ncbi.nlm.nih.gov/pubmed/?term=Volpe AR%5BAuthor%5D&cauthor=true&cauthor_uid=22448262). Cadmium induces p53-dependent apoptosis in human prostate epithelial cells. PLoS One. 2012;7(3):e33647.

# [**Cai C**](http://www.ncbi.nlm.nih.gov/pubmed/?term=Cai C%5BAuthor%5D&cauthor=true&cauthor_uid=22174378), [**Hsieh CL**](http://www.ncbi.nlm.nih.gov/pubmed/?term=Hsieh CL%5BAuthor%5D&cauthor=true&cauthor_uid=22174378), [**Gao S**](http://www.ncbi.nlm.nih.gov/pubmed/?term=Gao S%5BAuthor%5D&cauthor=true&cauthor_uid=22174378). Soluble guanylyl cyclase α1 and p53 cytoplasmic sequestration and down-regulation in prostate cancer. Mol Endocrinol. 2012 Feb;26(2):292-307.

# [**Ecke TH**](http://www.ncbi.nlm.nih.gov/pubmed/?term=Ecke TH%5BAuthor%5D&cauthor=true&cauthor_uid=20592345), [**Schlechte HH**](http://www.ncbi.nlm.nih.gov/pubmed/?term=Schlechte HH%5BAuthor%5D&cauthor=true&cauthor_uid=20592345), [**Schiemenz K**](http://www.ncbi.nlm.nih.gov/pubmed/?term=Schiemenz K%5BAuthor%5D&cauthor=true&cauthor_uid=20592345). TP53 gene mutations in prostate cancer progression. Anticancer Res. 2010 May;30(5):1579-86.

# [**Macera MJ**](http://www.ncbi.nlm.nih.gov/pubmed/?term=Macera MJ%5BAuthor%5D&cauthor=true&cauthor_uid=9973923), [**Godec CJ**](http://www.ncbi.nlm.nih.gov/pubmed/?term=Godec CJ%5BAuthor%5D&cauthor=true&cauthor_uid=9973923), [**Sharma N**](http://www.ncbi.nlm.nih.gov/pubmed/?term=Sharma N%5BAuthor%5D&cauthor=true&cauthor_uid=9973923). Loss of heterozygosity of the TP53 tumor suppressor gene and detection of point mutations by the non-isotopic RNAse cleavage assay in prostate cancer. Cancer Genet Cytogenet. 1999 Jan 1;108(1):42-7.

# [**Martin P**](http://www.ncbi.nlm.nih.gov/pubmed/?term=Martin P%5BAuthor%5D&cauthor=true&cauthor_uid=21703421), [**Liu YN**](http://www.ncbi.nlm.nih.gov/pubmed/?term=Liu YN%5BAuthor%5D&cauthor=true&cauthor_uid=21703421), [**Pierce R**](http://www.ncbi.nlm.nih.gov/pubmed/?term=Pierce R%5BAuthor%5D&cauthor=true&cauthor_uid=21703421). Prostate epithelial Pten/TP53 loss leads to transformation of multipotential progenitors and epithelial to mesenchymal transition. Am J Pathol. 2011 Jul;179(1):422-35.

# [**Wang Y**](http://www.ncbi.nlm.nih.gov/pubmed/?term=Wang Y%5BAuthor%5D&cauthor=true&cauthor_uid=23982184), [**Zhang YX**](http://www.ncbi.nlm.nih.gov/pubmed/?term=Zhang YX%5BAuthor%5D&cauthor=true&cauthor_uid=23982184), [**Kong CZ**](http://www.ncbi.nlm.nih.gov/pubmed/?term=Kong CZ%5BAuthor%5D&cauthor=true&cauthor_uid=23982184). Loss of P53 facilitates invasion and metastasis of prostate cancer cells. Mol Cell Biochem. 2013 Dec;384(1-2):121-7.

# [**Schlechte H**](http://www.ncbi.nlm.nih.gov/pubmed/?term=Schlechte H%5BAuthor%5D&cauthor=true&cauthor_uid=9803007), [**Lenk SV**](http://www.ncbi.nlm.nih.gov/pubmed/?term=Lenk SV%5BAuthor%5D&cauthor=true&cauthor_uid=9803007), [**Löning T**](http://www.ncbi.nlm.nih.gov/pubmed/?term=Löning T%5BAuthor%5D&cauthor=true&cauthor_uid=9803007). p53 tumour suppressor gene mutations in benign prostatic hyperplasia and prostate cancer. Eur Urol. 1998;34(5):433-40.

# [**Wang YM**](http://www.ncbi.nlm.nih.gov/pubmed/?term=Wang YM%5BAuthor%5D&cauthor=true&cauthor_uid=23805288), [**Liu ZW**](http://www.ncbi.nlm.nih.gov/pubmed/?term=Liu ZW%5BAuthor%5D&cauthor=true&cauthor_uid=23805288), [**Guo JB**](http://www.ncbi.nlm.nih.gov/pubmed/?term=Guo JB%5BAuthor%5D&cauthor=true&cauthor_uid=23805288). ESR1 Gene Polymorphisms and Prostate Cancer Risk: A HuGE Review and Meta-Analysis. PLoS One. 2013 Jun 21;8(6):e66999.

# [**McIntyre MH**](http://www.ncbi.nlm.nih.gov/pubmed/?term=McIntyre MH%5BAuthor%5D&cauthor=true&cauthor_uid=18006911), [**Kantoff PW**](http://www.ncbi.nlm.nih.gov/pubmed/?term=Kantoff PW%5BAuthor%5D&cauthor=true&cauthor_uid=18006911), [**Stampfer MJ**](http://www.ncbi.nlm.nih.gov/pubmed/?term=Stampfer MJ%5BAuthor%5D&cauthor=true&cauthor_uid=18006911). Prostate cancer risk and ESR1 TA, ESR2 CA repeat polymorphisms. Cancer Epidemiol Biomarkers Prev. 2007 Nov;16(11):2233-6.

# [**Hatano K**](http://www.ncbi.nlm.nih.gov/pubmed/?term=Hatano K%5BAuthor%5D&cauthor=true&cauthor_uid=23788635), [**Yamaguchi S**](http://www.ncbi.nlm.nih.gov/pubmed/?term=Yamaguchi S%5BAuthor%5D&cauthor=true&cauthor_uid=23788635), [**Nimura K**](http://www.ncbi.nlm.nih.gov/pubmed/?term=Nimura K%5BAuthor%5D&cauthor=true&cauthor_uid=23788635). Residual prostate cancer cells after docetaxel therapy increase the tumorigenic potential via constitutive signaling of CXCR4, ERK1/2 and c-Myc. Mol Cancer Res. 2013 Sep;11(9):1088-100.

# [**Itkonen HM**](http://www.ncbi.nlm.nih.gov/pubmed/?term=Itkonen HM%5BAuthor%5D&cauthor=true&cauthor_uid=23720054), [**Minner S**](http://www.ncbi.nlm.nih.gov/pubmed/?term=Minner S%5BAuthor%5D&cauthor=true&cauthor_uid=23720054), [**Guldvik IJ**](http://www.ncbi.nlm.nih.gov/pubmed/?term=Guldvik IJ%5BAuthor%5D&cauthor=true&cauthor_uid=23720054)O-GlcNAc transferase integrates metabolic pathways to regulate the stability of c-MYC in human prostate cancer cells. [**Cancer Res.**](http://www.ncbi.nlm.nih.gov/pubmed/?term=Cancer+Res.+2013+Aug+15%3B73(16)%3A5277-87.)**2013**Aug 15;**73**(**16**):**5277-87**.

# c-Myc expression and MEK1-induced Erk2 nuclear localization are required for TGF-beta induced epithelial-mesenchymal transition and invasion in prostate cancer. Carcinogenesis. 2012 Oct;33(10):1965-75.

# [**Rohan JN**](http://www.ncbi.nlm.nih.gov/pubmed/?term=Rohan JN%5BAuthor%5D&cauthor=true&cauthor_uid=19164469), [**Weigel NL**](http://www.ncbi.nlm.nih.gov/pubmed/?term=Weigel NL%5BAuthor%5D&cauthor=true&cauthor_uid=19164469). 1Alpha,25-dihydroxyvitamin D3 reduces c-Myc expression, inhibiting proliferation and causing G1 accumulation in C4-2 prostate cancer cells. [**Endocrinology.**](http://www.ncbi.nlm.nih.gov/pubmed/?term=Endocrinology.+2009+May%3B150(5)%3A2046-54.) **2009**May;**150**(**5**):**2046-54**.

# [**Chuan YC**](http://www.ncbi.nlm.nih.gov/pubmed/?term=Chuan YC%5BAuthor%5D&cauthor=true&cauthor_uid=20010876), [**Iglesias-Gato D**](http://www.ncbi.nlm.nih.gov/pubmed/?term=Iglesias-Gato D%5BAuthor%5D&cauthor=true&cauthor_uid=20010876), [**Fernandez-Perez L**](http://www.ncbi.nlm.nih.gov/pubmed/?term=Fernandez-Perez L%5BAuthor%5D&cauthor=true&cauthor_uid=20010876).Ezrin mediates c-Myc actions in prostate cancer cell invasion. Oncogene. 2010 Mar 11;29(10):1531-42.

# Overexpression of C-MYC oncogene in prostate cancer predicts biochemical recurrence. Prostate Cancer Prostatic Dis. 2010 Dec;13(4):311-5.

# [**Gurel B**](http://www.ncbi.nlm.nih.gov/pubmed/?term=Gurel B%5BAuthor%5D&cauthor=true&cauthor_uid=18567993), [**Iwata T**](http://www.ncbi.nlm.nih.gov/pubmed/?term=Iwata T%5BAuthor%5D&cauthor=true&cauthor_uid=18567993), [**Koh CM**](http://www.ncbi.nlm.nih.gov/pubmed/?term=Koh CM%5BAuthor%5D&cauthor=true&cauthor_uid=18567993). Nuclear MYC protein overexpression is an early alteration in human prostate carcinogenesis. Mod Pathol. 2008 Sep;21(9):1156-67 Mod Pathol. 2008 Sep;21(9):1156-67.

# [**Lee S**](http://www.ncbi.nlm.nih.gov/pubmed/?term=Lee S%5BAuthor%5D&cauthor=true&cauthor_uid=24239550), [**Chun JN**](http://www.ncbi.nlm.nih.gov/pubmed/?term=Chun JN%5BAuthor%5D&cauthor=true&cauthor_uid=24239550), [**Kim SH**](http://www.ncbi.nlm.nih.gov/pubmed/?term=Kim SH%5BAuthor%5D&cauthor=true&cauthor_uid=24239550). Icilin inhibits E2F1-mediated cell cycle regulatory programs in prostate cancer. Biochem Biophys Res Commun. 2013 Nov 29;441(4):1005-10.

1. Huang H, Tindall DJ. The role of the androgen receptor in prostate cancer. Critical reviews in eukaryotic gene expression. 2002; 12:193–207.
2. Grossmann ME, Huang H, Tindall DJ. Androgen receptor signaling in androgen-refractory prostate cancer. Journal of the National Cancer Institute. 2001; 93:1687–1697.
3. Mohler JL, Gregory CW, Ford OH 3rd. The androgen axis in recurrent prostate cancer. Clin Cancer Res. 2004; 10:440–448.
4. Mani RS, Tomlins SA, Callahan K. Induced chromosomal proximity and gene fusions in prostate cancer. Science. 2009; 326:1230.
5. Carver BS, Tran J, Gopalan A, et al. Aberrant ERG expression cooperates with loss of PTEN to promote cancer progression in the prostate. Nature genetics. 2009; 41:619–624.
6. King JC, Xu J, Wongvipat J, et al. Cooperativity of TMPRSS2-ERG with PI3-kinase pathway activation in prostate oncogenesis. Nature genetics. 2009; 41:524–526.
7. Kumar-Sinha C, Tomlins SA, Chinnaiyan AM. Recurrent gene fusions in prostate cancer. Nat Rev Cancer. 2008; 8:497–511.
8. Tomlins SA, Rhodes DR, Perner S, et al. Recurrent fusion of TMPRSS2 and ETS transcription factor genes in prostate cancer. Science. 2005; 310:644–648.
9. Kuroda K, Liu H, Kim S, Guo M, Navarro V, Bander NH. Docetaxel down-regulates the expression of androgen receptor and prostate-specific antigen but not prostate-specific membrane antigen in prostate cancer cell lines: implications for PSA surrogacy. The Prostate. 2009.
10. Yang G, Ayala G, De Marzo A, et al. Elevated Skp2 protein expression in human prostate cancer: association with loss of the cyclin-dependent kinase inhibitor p27 and PTEN and with reduced recurrence-free survival. Clin Cancer Res. 2002; 8:3419–3426.
11. Deocampo ND, Huang H, Tindall DJ. The role of PTEN in the progression and survival of prostate cancer. Minerva endocrinologica. 2003; 28:145–153.
12. Huang H, Regan KM, Wang F, et al. Skp2 inhibits FOXO1 in tumor suppression through ubiquitin-mediated degradation. Proceedings of the National Academy of Sciences of the United States of America. 2005; 102:1649–1654.
13. Wu X, Senechal K, Neshat MS, Whang YE, Sawyers CL. The PTEN/MMAC1 tumor suppressor phosphatase functionsas a negative regulator of the phosphoinositide 3-kinase/Akt pathway. Proceedings of the National Academy of Sciences of the United States of America. 1998;95:15587–15591.
14. Gan L, Chen S, Wang Y, et al. Inhibition of the androgen receptor as a novel mechanism of taxol chemotherapy in prostate cancer. Cancer research. 2009; 69:8386–8394.
15. Ma Q, Fu W, Li P, et al. FoxO1 mediates PTEN suppression of androgen receptor N- and Cterminal interactions and coactivator recruitment. Molecular endocrinology.
16. Liu P, Li S, Gan L, Kao TP, Huang H. A transcription-independent function of FOXO1 in inhibition of androgen-independent activation of the androgen receptor in prostate cancer cells. Cancer research. 2008; 68:10290–10299.
17. Dong XY, Chen C, Sun X, et al. FOXO1A is a candidate for the 13q14 tumor suppressor gene inhibiting androgen receptor signaling in prostate cancer. Cancer research. 2006; 66:6998–7006.
18. Haldar S, Chintapalli J, Croce CM. Taxol induces bcl-2 phosphorylation and death of prostate cancer cells. Cancer research. 1996; 56:1253–1255.
19. Petrylak DP, Tangen CM, Hussain MH, et al. Docetaxel and estramustine compared with mitoxantrone and prednisone for advanced refractory prostate cancer. The New England journal of medicine. 2004; 351:1513–1520.
20. Tannock IF, de Wit R, Berry WR, et al. Docetaxel plus prednisone or mitoxantrone plus prednisone for advanced prostate cancer. The New England journal of medicine. 2004; 351:1502–1512.
21. Smith DC, Pienta KJ. Paclitaxel in the treatment of hormone-refractory prostate cancer. Seminars in oncology. 1999; 26(1 Suppl 2):109–111.
22. Chun JY, Nadiminty N, Dutt S, et al. Interleukin-6 regulates androgen synthesis in prostate cancer cells. Clin Cancer Res. 2009; 15:4815–4822.
23. Borre M, Offersen BV, Nerstrom B and Overgaard J. Microvessel density predicts survival in prostate cancer patients subjected to watchful waiting. Br J Cancer 1998; 78: 940-944.
24. Weidner N, Carroll PR, Flax J, Blumenfeld W and Folkman J: Tumor angiogenesis correlates with metastasis in invasive prostate carcinoma. Am J Pathol. 1993; 143: 401-409.
25. Ferrer FA, Miller LJ, Lindquist R, Kowalczyk P, Laudone VP, Albertsen PC and Kreutzer DL: Expression of vascular endothelial growth factor receptors in human prostate cancer. Urology 1999; 54: 567-572.
26. Smith MR, Saad F, Egerdie B, *et al*: Effects of denosumab on bone mineral density in men receiving androgen deprivation therapy for prostate cancer. J Urol ; 182: 2670-2675.
27. Fizazi K, Yang J, Peleg S, *et al*: Prostate cancer cells-osteoblast interaction shifts expression of growth/survival-related genes in prostate cancer and reduces expression of osteoprotegerin in osteoblasts. Clin Cancer Res 2003; 9: 2587-2597.
28. Tu SM, Millikan RE, Mengistu B, *et al*: Bone-targeted therapy for advanced androgen-independent carcinoma of the prostate: a randomised phase II trial. Lancet 2001; 357: 336-341.
29. James ND, Caty A, Borre M, *et al*: Safety and efficacy of the specific endothelin-A receptor antagonist ZD4054 in patients with hormone-resistant prostate cancer and bone metastases who were pain free or mildly symptomatic: a double-blind, placebo-controlled, randomised, phase 2 trial. Eur Urol 2009; 55: 1112-1123.
30. Mooso B, Madhav A, Johnson S, *et al*: Androgen receptor regu­lation of vitamin D receptor in response of castration-resistant prostate cancer cells to 1α-hydroxyvitamin D5 - a calcitriol analog. Genes Cancer 2010; 1: 927-940.
31. Nam S, Kim D, Cheng JQ, *et al*: Action of the Src family kinase inhibitor, dasatinib (BMS-354825), on human prostate cancer cells. Cancer Res 2005; 65: 9185-9189.
32. Asim M, Siddiqui IA, Hafeez BB, Baniahmad A and Mukhtar H: Src kinase potentiates androgen receptor transactivation function and invasion of androgen-independent prostate cancer C4-2 cells. Oncogene 2008; 27: 3596-3604.
33. Di Lorenzo G, Figg WD, Fossa SD, *et al*: Combination of bevaci­zumab and docetaxel in docetaxel-pretreated hormone-refractory prostate cancer: a phase 2 study. Eur Urol 2008; 54: 1089-1094.
34. Stavridi F, Karapanagiotou EM and Syrigos KN: Targeted therapeutic approaches for hormone-refractory prostate cancer. Cancer Treat Rev 2010; 36: 122-130.
35. Beltran H, Rickman DS, Park K, et al. Molecular characterization of neuroendocrine prostate cancer and identification of new drug targets. Cancer Discov 2011;1:487-95.
36. Terry S, Maillé P, Baaddi H, et al. Cross modulation between the androgen receptor axis and protocadherin- PC in mediating neuroendocrine transdifferentiation and therapeutic resistance of prostate cancer. Neoplasia 2013;15:761-72.
37. Gnoni A, Marech I, Silvestris N, et al. Dasatinib: an anti-tumour agent via Src inhibition. Curr Drug Targets 2011;12:563-78.
38. Yang JC, Bai L, Yap S, et al. Effect of the specific Src family kinase inhibitor saracatinib on osteolytic lesions using the PC-3 bone model. Mol Cancer Ther 2010;9:1629-37.
39. Lee LF, Louie MC, Desai SJ, et al. Interleukin-8 confers androgen-independent growth and migration of LNCaP: differential effects of tyrosine kinases Src and FAK. Oncogene 2004;23:2197-205.
40. Kung HJ, Evans CP. Oncogenic activation of androgen receptor. Urol Oncol 2009;27:48-52. Fizazi K.
41. The role of Src in prostate cancer. Ann Oncol 2007;18:1765-73.
42. Thomas C, Lamoureux F, Crafter C, et al. Synergistic targeting of PI3K/AKT-pathway and androgen-receptor axis significantly delays castration-resistant prostate cancer progression in vivo. Mol Cancer Ther 2013;12:2342-55.
43. Pourmand G, Ziaee AA, Abedi AR, et al. Role of PTEN gene in progression of prostate cancer. Urol J 2007;4:95-100. Fang J, Ding M, Yang L, et al. PI3K/PTEN/AKT signaling regulates prostate tumor angiogenesis. Cell Signal 2007;19:2487-97.
44. Reid AH, Attard G, Ambroisine L, et al. Molecular characterisation of ERG, ETV1 and PTEN gene loci identifies patients at low and high risk of death from prostate cancer. Br J Cancer 2010;102:678-84.
45. McCubrey JA, Steelman LS, Kempf CR, et al. Therapeutic resistance resulting from mutations in Raf/MEK/ERK and PI3K/PTEN/Akt/mTOR signaling pathways. J Cell Physiol 2011;226:2762-81.
46. Schayowitz A, Sabnis G, Njar VC, et al. Synergistic effect of a novel antiandrogen, VN/124-1, and signal transduction inhibitors in prostate cancer progression to hormone independence in vitro. Mol Cancer Ther 2008;7:121-32.
47. Taylor BS, Schultz N, Hieronymus H, et al. Integrative genomic profiling of human prostate cancer. Cancer Cell 2010;18:11-22.
48. Karlou M, Lu JF, Wu G, et al. Hedgehog signaling inhibition by the small molecule smoothened inhibitor GDC-0449 in the bone forming prostate cancer xenograft MDA PCa 118b. Prostate 2012;72:1638-47.
49. Bragina O, Njunkova N, Sergejeva S, et al. Sonic Hedgehog pathway activity in prostate cancer. Oncol Lett 2010;1:319-25.
50. Dayyani F, Parikh NU, Varkaris AS, et al. Combined Inhibition of IGF-1R/IR and Src family kinases enhances antitumor effects in prostate cancer by decreasing activated survival pathways. PLoS One 2012;7:e51189.
51. Kawada M, Inoue H, Masuda T, et al. Insulin-like growth factor I secreted from prostate stromal cells mediates tumor-stromal cell interactions of prostate cancer. Cancer Res 2006;66:4419-25.
52. Cano P, Godoy A, Escamilla R, et al. Stromal-epithelial cell interactions and androgen receptor-coregulator recruitment is altered in the tissue microenvironment of prostate cancer. Cancer Res 2007;67:511-9.
53. Cano P, Godoy A, Escamilla R, et al. Stromal-epithelial cell interactions and androgen receptor-coregulator recruitment is altered in the tissue microenvironment of prostate cancer. Cancer Res 2007;67:511-9.
54. Sluka P, Davis ID. Cell mates: paracrine and stromal targets for prostate cancer therapy. Nat Rev Urol 2013;10:441-51.
55. Haffner MC, Aryee MJ, Toubaji A, et al. Androgen-induced TOP2B-mediated double-strand breaks and prostate cancer gene rearrangements. Nat Genet 2010;42:668-75.
56. Mendes-Pereira AM, Martin SA, Brough R, et al. Synthetic lethal targeting of PTEN mutant cells with PARP inhibitors. EMBO Mol Med 2009;1:315-22.
57. Gallagher DJ, Gaudet MM, Pal P, et al. Germline BRCA mutations denote a clinicopathologic subset of prostate cancer. Clin Cancer Res 2010;16:2115-21.
58. Tolcher AW, Kuhn J, Schwartz G, et al. A Phase I pharmacokinetic and biological correlative study of oblimersen sodium (genasense, g3139), an antisense oligonucleotide to the bcl-2 mRNA, and of docetaxel in patients with hormone-refractory prostate cancer. Clin Cancer Res 2004;10:5048-57.
59. Sternberg CN, Dumez H, Van Poppel H, et al. Docetaxel plus oblimersen sodium (Bcl-2 antisense oligonucleotide): an EORTC multicenter, randomized phase II study in patients with castration-resistant prostate cancer. Ann Oncol 2009;20:1264-9.
60. McDonnell TJ, Troncoso P, Brisbay SM, et al. Expression of the protooncogene bcl-2 in the prostate and its association with emergence of androgen-independent prostate cancer. Cancer Res 1992;52:6940-4.
61. Zoubeidi A, Zardan A, Beraldi E, et al. Cooperative interactions between androgen receptor (AR) and heat-shock protein 27 facilitate AR transcriptional activity. Cancer Res 2007;67:10455-65.
62. Zoubeidi A, Zardan A, Wiedmann RM, et al. Hsp27 promotes insulin-like growth factor-I survival signaling in prostate cancer via p90Rsk-dependent phosphorylation and inactivation of BAD. Cancer Res 2010;70:2307-17.
63. Matsumoto H, Yamamoto Y, Shiota M, et al. Cotargeting androgen receptor and clusterin delays castrate-resistant prostate cancer progression by inhibiting adaptive stress response and AR stability. Cancer Res 2013;73:5206-17.
64. Spratt DE, Zhang C, Zumsteg ZS, et al. Metformin and prostate cancer: reduced development of castration-resistant disease and prostate cancer mortality. Eur Urol 2013;63:709-16.
65. Kantoff PW, Higano CS, Shore ND, et al. Sipuleucel-T immunotherapy for castration-resistant prostate cancer. N Engl J Med 2010;363:411-22.
66. Caffo O, Pappagallo G, Brugnara S, et al. Multiple rechallenges for castration-resistant prostate cancer patients responding to first-line docetaxel: assessment of clinical outcomes and predictive factors. Urology 2012;79:644-9.
67. Petrylak DP, Tangen CM, Hussain MH, et al. Docetaxel and estramustine compared with mitoxantrone and prednisone for advanced refractory prostate cancer. N Engl J Med 2004;351:1513-20.
68. Zhu ML, Horbinski CM, Garzotto M, et al. Tubulin-targeting chemotherapy impairs androgen receptor activity in prostate cancer. Cancer Res 2010;70:7992-8002.
69. Carver BS, Chapinski C, Wongvipat J, et al. Reciprocal feedback regulation of PI3K and androgen receptor signaling in PTEN-deficient prostate cancer. Cancer Cell 2011;19:575-86.
70. Wang Y, Kreisberg JI, Ghosh PM. Cross-talk between the androgen receptor and the phosphatidylinositol 3-kinase/ Akt pathway in prostate cancer. Curr Cancer Drug Targets 2007;7:591-604.
71. Green SM, Mostaghel EA, Nelson PS. Androgen action and metabolism in prostate cancer. Mol Cell Endocrinol. 2012. Epub Mar 20.
72. Shiota M, Yokomizo A, Naito S. Increased androgen receptor tran­scription: a cause of castration-resistant prostate cancer and a possible therapeutic target. J Mol Endocrinol. 2011;47(1):R25–R41.
73. Golias Ch, Iliadis I, Peschos D, Charalabopoulos K. Amplification and co-regulators of androgen receptor gene in prostate cancer. Exp Oncol. 2009;31(1):3–8.
74. Ward JE, McNeel DG. GVAX: an allogeneic, whole-cell, GM-CSF-secreting cellular immunotherapy for the treatment of prostate cancer. Expert Opin Biol Ther. 2007;7(12):1893–1902.
75. Fishman M. A changing world for DCvax: a PSMA loaded autologous dendritic cell vaccine for prostate cancer. Expert Opin Biol Ther. 2009;9(12):1565–1575.
76. Kim SH, Sehrawat A, Sakao K, Hahm ER, Singh SV. Notch activation by phenethyl isothiocyanate attenuates its inhibitory effect on prostate cancer cell migration. PLoS One. 2011;6(10):e26615.
77. Kong D, Banerjee S, Ahmad A, et al. Epithelial to mesenchymal tran­sition is mechanistically linked with stem cell signatures in prostate cancer cells. PLoS One. 2010;5(8):e12445.
78. Bin Hafeez B, Adhami VM, Asim M, et al. Targeted knockdown of Notch1 inhibits invasion of human prostate cancer cells concomitant with inhibition of matrix metalloproteinase-9 and urokinase plasmino­gen activator. Clin Cancer Res. 2009;15(2):452–459.
79. Bin Hafeez B, Adhami VM, Asim M, et al. Targeted knockdown of Notch1 inhibits invasion of human prostate cancer cells concomitant with inhibition of matrix metalloproteinase-9 and urokinase plasmino­gen activator. Clin Cancer Res. 2009;15(2):452–459.
80. Bin Hafeez B, Adhami VM, Asim M, et al. Targeted knockdown of Notch1 inhibits invasion of human prostate cancer cells concomitant with inhibition of matrix metalloproteinase-9 and urokinase plasmino­gen activator. Clin Cancer Res. 2009;15(2):452–459.
81. Whelan JT, Kellogg A, Shewchuk BM, Hewan-Lowe K, Bertrand FE. Notch-1 signaling is lost in prostate adenocarcinoma and promotes PTEN gene expression. J Cell Biochem. 2009;107(5):992–1001.
82. Martin DB, Gifford DR, Wright ME, et al. Quantitative proteomic analysis of proteins released by neoplastic prostate epithelium. Cancer Res. 2004;64(1):347–355.
83. Belandia B, Powell SM, García-Pedrero JM, Walker MM, Bevan CL, Parker MG. Hey1, a mediator of notch signaling, is an androgen receptor corepressor. Mol Cell Biol. 2005;25(4):1425–1436.
84. Wang XD, Leow CC, Zha J, et al. Notch signaling is required for normal prostatic epithelial cell proliferation and differentiation. Dev Biol. 2006;290(1):66–80.
85. Kwon GT, Jung JI, Song HR, et al. Piceatannol inhibits migration and invasion of prostate cancer cells: possible mediation by decreased interleukin-6 signaling. J Nutr Biochem. 2012;23(3):228–238.
86. Kim C, Kim MC, Kim SM, et al. Chrysanthemum indicum L. Extract Induces Apoptosis through Suppression of Constitutive STAT3Activation in Human Prostate Cancer DU145 Cells. Phytother Res. 2012. Epub Mar 22.
87. Shanmugam MK, Rajendran P, Li F, et al. Ursolic acid inhibits multiple cell survival pathways leading to suppression of growth of prostate can­cer xenograft in nude mice. J Mol Med (Berl). 2011;89(7):713–727.
88. Sun M, Liu C, Nadiminty N, Lou W, et al. Inhibition of Stat3 activation by sanguinarine suppresses prostate cancer cell growth and invasion. Prostate. 2012;72(1):82–89.
89. He M, Young CY. New approaches to target the androgen receptor and STAT3 for prostate cancer treatments. Mini Rev Med Chem. 2009;9(3):395–400.
90. Drachenberg DE, Elgamal AA, Rowbotham R, Peterson M, Murphy GP. Circulating levels of interleukin-6 in patients with hormone refractory prostate cancer. Prostate. 1999;41(2):127–133.
91. Liu X, He Z, Li CH, Huang G, Ding C, Liu H. Correlation analysis of JAK-STAT pathway components on prognosis of patients with prostate cancer. Pathol Oncol Res. 2012;18(1):17–23.
92. Lou W, Ni ZY, Dyer K, Tweardy DJ, Gao AC. Interleukin-6 induces prostate cancer cell growth accompanied by activation of stat3 signaling pathway. Prostate. 2000;42(3):239–242.
93. Han G, Yu JY, Chen YD, et al. The usefulness of phosphorylated-signal transduction and activators of transcription 3 in detecting prostate cancer from negative biopsies. Eur J Surg Oncol. 2012;38(4):367–373.
94. Majumder PK, Febbo PG, Bikoff R, et al. mTOR inhibition reverses Akt-dependent prostate intraepithelial neoplasia through regulation of apop­totic and HIF-1-dependent pathways. *Nat Med*. 2004;10(6):594–601.
95. Chee KG, Longmate J, Quinn DI, et al. The AKT inhibitor perifosine in biochemically recurrent prostate cancer: a phase II California/Pittsburgh cancer consortium trial. Clin Genitourin Cancer. 2007;5(7):433–437.
96. Seol JW, Lee YJ, Kang HS, et al. Wortmannin elevates tumor necrosis factor-related apoptosis-inducing ligand sensitivity in LNCaP cells through down-regulation of IAP-2 protein. Exp Oncol. 2005;27(2): 120–124.
97. Diehl JA, Cheng M, Roussel MF, Sherr CJ. Glycogen synthase kinase-3beta regulates cyclin D1 proteolysis and subcellular localization. Genes Dev. 1998;12(22):3499–3511.
98. Shin I, Yakes FM, Rojo F, et al. PKB/Akt mediates cell-cycle progres­sion by phosphorylation of p27(Kip1) at threonine 157 and modulation of its cellular localization. Nat Med. 2002;8(10):1145–1152.
99. Kharaziha P, Rodriguez P, Li Q, et al. Targeting of distinct signaling cas­cades and cancer-associated fibroblasts define the efficacy of Sorafenib against prostate cancer cells. Cell Death Dis. 2012;3:e262.42.
100. Carrión-Salip D, Panosa C, Menendez JA, et al. Androgen-independent prostate cancer cells circumvent EGFR inhibition by overexpression of alternative HER receptors and ligands. Int J Oncol. 2012. Epub Jun 6.
101. Koreckij TD, Trauger RJ, Montgomery RB, et al. HE3235 inhibits growth of castration-resistant prostate cancer. Neoplasia. 2009;11(11): 1216–1225.
102. Snoek R, Cheng H, Margiotti K, et al. In vivo knockdown of the androgen receptor results in growth inhibition and regression of well-established, castration-resistant prostate tumors. Clin Cancer Res. 2009;15(1):39–47.
103. Chi KN, Bjartell A, Dearnaley D, et al. Castration resistant prostate cancer: from new pathophysiology to new treatment targets. Eur Urol. 2009;56(4):594–605.
104. Sun S, Sprenger CC, Vessella RL, et al. Castration resistance in human prostate cancer is conferred by a frequently occurring androgen receptor splice variant. J Clin Invest. 2010;120(8):2715–2730.
105. Watson PA, Chen YF, Balbas MD, et al. Constitutively active andro­gen receptor splice variants expressed in castration-resistant prostate cancer require full-length androgen receptor. Proc Natl Acad Sci U S A. 2010;107(39):16759–16765.
106. Dehm SM, Schmidt LJ, Heemers HV, Vessella RL, Tindall DJ. Splicing of a novel androgen receptor exon generates a constitutively active androgen receptor that mediates prostate cancer therapy resistance. Cancer Res. 2008;68(13):5469–5477.
107. Dehm SM, Tindall DJ. Alternatively spliced androgen receptor variants. Endocr Relat Cancer. 2011;18(5):R183–R196.
108. Cai C, Chen S, Ng P, et al. Intratumoral de novo steroid synthesis activates androgen receptor in castration-resistant prostate cancer and is upregulated by treatment with CYP17A1 inhibitors. Cancer Res. 2011;71(20):6503–6513.
109. Golias Ch, Iliadis I, Peschos D, Charalabopoulos K. Amplification and co-regulators of androgen receptor gene in prostate cancer. Exp Oncol. 2009;31(1):3–8.
110. Jain G, Voogdt C, Tobias A et al. IκB kinases modulate the activity of the androgen receptor in prostate carcinoma cell lines. Neoplasia 2012; 14:178–89.
111. Ammirante M, Kuraishy AI, Shalapour S et al. An IKKα-E2F1-BMI1 cascade activated by infiltrating B cells controls prostate regeneration and tumor recurrence. Genes Dev 2013; 27: 1435–40.
112. Ammirante M, Luo J, Grivennikov S, Nedospasov S, Karin M. B-cell derived lymphotoxin promotes castration-resistant prostate cancer. Nature*;* 2010; 464: 302–5.
113. Luo JL, Tan W, Ricono JM et al. Nuclear cytokine-activated IKKalpha controls prostate cancer metastasis by repressing Maspin. Nature 2007;446: 690–4.
114. Suh J, Payvandi F, Edelstein LC et al. Mechanisms of constitutive NF-kappaB activation in human prostate cancer cells. Prostate 2002; 52:183–200.
115. Nadiminty N, Lou W, Sun M et al. Aberrant activation of the androgen receptor by NF-kappaB2/p52 in prostate cancer cells. Cancer Res 2010; 70:3309–19.
116. Zhang L, Altuwaijri S, Deng F et al. NF-kappaB regulates androgen receptor expression and prostate cancer growth. Am J Pathol 2009; 175:489–99.
117. Jin RJ, Lho Y, Connelly L et al. The nuclear factor-kappaB pathways controls the progression of prostate cancer to androgen-independent growth. Cancer Res 2008; 68: 6762–9.
118. Ko S, Shi L, Kim S, Song CS, Chatterjee B. Interplay of nuclear factor-kappaB and B-myb in the negative regulation of androgen receptor expression by tumor necrosis factor alpha. Mol Endocrinol 2008; 22:273–86.
119. Nelius T, Filleur S, Yemelyanov A et al. Androgen receptor targets NFkappaB and TSP1 to suppress prostate tumor growth in vivo. Int J Cancer 2007; 121: 999–1008.
120. Graham TR, Odero-Marah VA, Chung LW, Agrawal KC, Davis R, Abdel-Mageed AB. PI3K/Akt-dependent transcriptional regulation and activation of BMP-2-Smad signaling by NF-kappaB in metastatic prostate

cancer cells. Prostate 2009; 69: 168–80.

1. Andela VB, Gordon AH, Zotalis G et al. NFkappaB: a pivotal transcription factor in prostate cancer metastasis to bone. Clin Orthop Relat Res 2003; 415: S75–85.
2. Longoni N, Sarti M, Albino D et al. ETS transcription factor ESE1/ELF orchestrates a positive feedback loop that constitutively activates NF-κB and drives prostate cancer progression. Cancer Res 2013; 73: 4533–47.
3. Killian PH, Kronski E, Michalik KM et al. Curcumin inhibits prostate cancer metastasis in vivo by targeting the inflammatory cytokines CXCL1 and -2. Carcinogenesis 2012; 33: 2507–19.
4. De Angulo A, Faris R, Cavazos D, Jolly C, Daniel B, DeGraffenried L. Age-related alterations in T-lymphocytes modulate key pathways in prostate tumorigenesis. Prostate 2013; 73: 855–64.
5. Ismail HA, Lessard L, Mes-Masson AM, Saad F. Expression of NF-kappaB in prostate cancer lymph node metastases. Prostate 2004; 58:308–13.
6. Wong CP, Bray TM, Ho E. Induction of proinflammatory response in prostate cancer epithelial cells by activated macrophages. Cancer Lett 2009; 276: 38–46.
7. Xu Y, Josson S, Fang F et al. RelB enhances prostate cancer growth: implications for the role of the nuclear factor-kappaB alternative pathway in tumorigenicity. Cancer Res 2009; 69: 3267–71.
8. Chendil D, Das A, Dey S, Mohiuddin M, Ahmed MM. Par-4, a proapoptotic gene, inhibits radiation-induced NF kappa B activity and Bcl-2 expression leading to induction of radiosensitivity in human

prostate cancer cells PC-3. Cancer Biol Ther 2002; 1: 152–60.

1. Taichman RS, Cooper C, Keller ET, Pienta KJ, Taichman NS, McCauley LK. Use of the stromal cell-derived factor-1/CXCR4 pathway in prostate cancer metastasis to bone. Cancer Res 2002; 62: 1832–7.
2. Gueron G, De Siervi A, Vazquez E. Advanced prostate cancer: reinforcing the strings between inflammation and the metastatic behavior.Prostate Cancer Prostatic Dis 2012; 15: 213–21.
3. Hao J, Madigan MC, Khatri A, Power CA, Hung TT, Beretov J et al. In vitro and in vivo prostate cancer metastasis and chemoresistance can be modulated by expression of either CD44 or CD147. PLoS One 2012; 7: e40716.
4. Della Donna L, Lagadec C, Pajonk F. Radioresistance of prostate cancer cells with low proteasome activity. Prostate 2012; 72: 868–874.
5. Chang L, Graham PH, Hao JL, Bucci J, Cozzi PJ, Kearsley JK et al. Emerging roles of radioresistance in prostate cancer metastasis and radiation therapy. Cancer Metast Rev 2013.
6. Wu CT, Hsieh CC, Lin CC, Chen WC, Hong JH, Chen MF. Significance of IL-6 in the transition of hormone-resistant prostate cancer and the induction of myeloid-derived suppressor cells. J Mol Med 2012; 90:

1343–55.

1. Hao J, Madigan MC, Khatri A, Power CA, Hung TT, Beretov J et al. In vitro and in vivo prostate cancer metastasis and chemoresistance can be modulated by expression of either CD44 or CD147. PLoS One 2012; 7: e40716.
2. Hao JLNJ, Graham P, Cozzi PJ, Bucci J, Kearsley J et al. The CD44 isoforms in prostate cancer metastasis and progression. World J.Cancer Res 2013; 1: 3–14.
3. Xie D, Gore C, Liu J, Pong RC et al. Role of DAB2IP in modulating epithelial-to-mesenchymal transition and prostate cancer metastasis. Proc Natl Acad Sci U S A 2010; 107:2485–2490.
4. Mak P, Leav I, Pursell B, Bae D et al. ERbeta impedes prostate cancer EMT by destabilizing HIF-1alpha and inhibiting VEGF-mediated snail nuclear localization:implications for Gleason grading. Cancer Cell 2010;17:319–332.
5. Chang HH, Chen BY, Wu CY, Tsao ZJ et al. Hedgehog overexpression leads to the formation of prostate cancer stem cells with metastatic property irrespective of androgen receptor expression in the mouse model. J Biomed Sci 2011; 18:6.
6. Kong D, Wang Z, Sarkar SH, Li Y et al. Platelet-derived growth factor-D overexpression contributes to epithelial-mesenchymal transition of PC3 prostate cancer cells. Stem Cells 2008; 26:1425–1435.
7. Odero-Marah VA, Wang R, Chu G et al. Receptor activator of NF-kappaB Ligand (RANKL)expression is associated with epithelial to mesenchymal transition in human prostate cancer cells. Cell Res 2008; 18:858–870.
8. Odero-Marah VA, Wang R, Chu G et al. Receptor activator of NF-kappaB Ligand (RANKL) expression is associated with epithelial to mesenchymal transition in human prostate cancer cells. Cell Res 2008; 18:858–870.
9. Shimada K, Nakamura M, De Velasco MA et al. Syndecan-1, a new target molecule involved in progression of androgen-independent prostate cancer. Cancer Sci 2009; 100:1248–1254.
10. Drake JM, Barnes JM, Madsen JM et al. ZEB1 coordinately regulates laminin-332 and {beta}4 integrin expression altering the invasive phenotype of prostate cancer cells. J Biol Chem 2010; 285:33940–33948.
11. Neal CL, Mckeithen D, Odero-Marah VA. Snail negatively regulates cell adhesion to extracellular matrix and integrin expression via the MAPK pathway in prostate cancer cells. Cell Adh Migr 2011; 5:249–257.
12. Smith BN, Odero-Marah VA. The role of Snail in prostate cancer. Cell Adh Migr 2012; 6:433–441.
13. Kosaka T, Miyajima A, Shirotake S, Kikuchi E et al. Ets-1 and hypoxia inducible factor-1alpha inhibition by angiotensin II type-1 receptor blockade in hormone-refractory prostate cancer. Prostate 2010; 70:162–169.
14. Palapattu GS, Wu C, Silvers CR, Martin HB et al. Selective expression of CD44, a putative prostate cancer stem cell marker, in neuroendocrine tumor cells of human prostate cancer. Prostate 2009; 69:787–798.
15. Singh S, Sadacharan S, Su S et al. Overexpression of vimentin: role in the invasive phenotype in an androgen-independent model of prostate cancer. Cancer Res 2003; 63:2306–2311.
16. Fornaro M, Plescia J, Chheang S et al. Fibronectin protects prostate cancer cells from tumor necrosis factor-alpha-induced apoptosis via the AKT/survivin pathway. J Biol Chem 2003; 278:50402–50411.
17. Docheva D, Padula D, Schieker M, Clausen-Schaumann H. Effect of collagen I and fibronectin on the adhesion, elasticity and cytoskeletal organization of prostate cancer cells. Biochem Biophys Res Commun 2010; 402:361–366.
18. Trikha M, Raso E, Cai Y, Fazakas Z, Paku S, Porter AT, Timar J, Honn KV. Role of alphaII(b)beta3 integrin in prostate cancer metastasis. Prostate 1998; 35:185–192.
19. Gan Y, Shi C, Inge L, Hibner M, Balducci J, Huang Y. Differential roles of ERK and Akt pathways in regulation of EGFR-mediated signaling and motility in prostate cancer cells. Oncogene 2010; 29:4947–4958.
20. Pu H, Collazo J, Jones E, Gayheart D, Sakamoto S et al. Dysfunctional transforming growth factor-beta receptor II accelerates prostate tumorigenesis in the TRAMP mouse model. Cancer Res 2009; 69:7366–7374.
21. Ayala GE, Dai H, Tahir SA, et al. Stromal antiapototic paracrine loop in perineural invasion of prostatic carcinoma. Cancer Res. 2006; 66:5159–5164.
22. Morton DM, Barrack ER. Modulation of transforming growth factor-β effects on prostate cancer cell proliferation by growth factors and extracellular matrix. Cancer Res. 1995; 55:2596–2602.
23. Prins GS, Putz O. Molecular signaling pathways that regulate prostate gland development. Differentiation. 2008; 76:641–659.
24. Dehm SM, Tindall DJ. Androgen receptor structural and functional elements: role and regulation in prostate cancer. Mol. Endocrinol. 2007; 21(12):2855–2863.
25. Mellado B, Codony J, Ribal MJ, Visa L, Gascón P. Molecular biology of androgen-independent prostate cancer: the role of the androgen receptor pathway. Clin. Transl. Oncol. 2009; 11:5–10.
26. Zhu ML, Kyprianou N. Role of androgens and the androgen receptor in epithelial–mesenchymal transition and invasion of prostate cancer cells. FASEB J. 2010; 24:769–777.
27. Hara T, Miyazaki H, Lee A, Tran CP, Reiter RE. Androgen receptor and invasion in prostate cancer. Cancer Res. 2008; 68:1128–1135.
28. Jennbacken K, Tešan T, Wang W, Gustavsson H et al. N-cadherin increases after androgen deprivation and is associated with metastasis in prostate cancer. Endocr. Relat. Cancer. 2010; 17(2):469–479.
29. Li H, Price DK, William D, Figg WD. ADH1, an N-cadherin inhibitor, evaluated in preclinical models of angiogenesis and androgen-independent prostate cancer. Anticancer Drugs. 2007; 18(5): 563–568.
30. Chu K, Cheng CJ, Ye X, et al. Cadherin-11 promotes the metastasis of prostate cancer cells to bone. Mol. Cancer Res. 2008; 6:1259–1267.
31. Lee YC, Cheng CJ, Huang M, et al. Androgen depletion up-regulated cadherin-11 expression in prostate cancer. J. Pathol. 2010; 221:68–76.
32. Niu YN, Xia SJ. Stroma–epithelium crosstalk in prostate cancer. Asian. J. Androl. 2009; 11:28–35.
33. Robinson DR, Zylstra CR, Williams BO. Wnt signaling and prostate cancer. Curr. Drug Targets. 2008; 9:571–780.
34. Wang G, Wang J, Sadar MD. Crosstalk between the androgen receptor and β-catenin in castrate-resistant

prostate cancer. Cancer Res. 2008; 68:9918–9927.

1. Whitaker HC, Girling J, Warren AY, Leung H, Mills IG, Neal DE. Alterations in β-catenin expression and localization in prostate cancer. Prostate. 2008; 68:1196–1205.
2. Saha B, Arase A, Imam SS, et al. Overexpression of E-cadherin and β-catenin proteins in metastatic prostate cancer cells in bone. Prostate. 2008; 68:78–84.
3. Heebøll S, Borre M, Ottosen PD, Dyrskjøt L, Torben FØ, Tørring N. Snail1 is over-expressed in prostate cancer. APMIS. 2009; 117:196–204.
4. Baygi EM, Soheili ZS, Schmitz I, Sameie S, Schulz WA. Snail regulated cell survival and inhibits senescence in human metastatic prostate cancer cell lines. Cell Biol. Toxicol. 2010; 26:553–567.
5. Yuen HF, Chua CW, Chan YP, Wong YC, Wang X, Chan KW. Significance of TWIST and Ecadherin expression in the metastatic progression of prostatic cancer. Histopathology. 2007; 50:648–658.
6. Shiota M, Yokomizo A, Tada Y, et al. Castration resistance of prostate cancer cells caused by castration-induced oxidative stress through Twist1 and androgen receptor over expression. Oncogene. 2010; 29:237–250.
7. Wang H, McKnight NC, Zhang T, Lu ML, Balk SP, Yuan X. SOX9 is expressed in normal prostate basal cells and regulates androgen receptor expression in prostate cancer cells. Cancer Res. 2007; 67(2):528–536.
8. Graham T, Zhau H, Odero-Marah V, et al. Insulin-like growth factor-1-dependent up-regulation of ZEB1 drives epithelial-to-mesenchymal transition in human prostate cancer cells. Cancer Res. 2008; 68 :2479–2488.
9. Anose BM, LaGoo L, Schwendinger J. Characterization of androgen regulation of ZEB-1 and PSA in 22RV1 prostate cancer cells. Adv. Exp. Med. Biol. 2008; 617:541–546.
10. Drake JM, Strohbehn G, Moreland JG, Henry MD. ZEB1 enhances trans endothelial migration and represses the epithelial phenotype of prostate cancer cells. Mol. Biol. Cell. 2009; 20:2207–2217.
11. Yang F, Tuxhorn JA, Ressler SJ, McAlhany SJ et al.. Stromal expression of connective tissue growth factor promotes angiogenesis and prostate cancer tumorigenesis. Cancer Res. 2005; 65(19):8887–8895.
12. Escaff S, Fernández JM, González LO, et al. Study of matrix metallo proteinases and their inhibitors in prostate cancer. Br. J. Cancer. 2010; 102:922–929.
13. Ostling P, Leivonen SK, Aakula A, et al. Systematic analysis of micrornas targeting the androgen receptor in prostate cancer cells . Cancer Res. 2011; 71:1956–1967.
14. Hughes C, Murphy A, Martin C, Sheils O and O'Leary J. Molecular pathology of prostate cancer*.* J Clin Pathol 2005; 58: 673-84.
15. Qin HR, Iliopoulos D, Semba S et al. A role for the WWOX gene in prostate cancer*.* Cancer Res 2006; 66: 6477-81.
16. Lane TM, Strefford JC, Yanez-Munoz RJ et al. Identification of a recurrent t(4;6) chromosomal translocation in prostate cancer*.* J Urol 2007; 177: 1907-12.
17. Berger MF, Lawrence MS, Demichelis F, Drier Y et al. The genomic complexity of primary human prostate cancer*.* Nature 2011; 470: 214-20.
18. van Bokhoven A, Caires A, Maria MD, SchulteAP. Spectral karyotype (SKY) analysis of human prostate carcinoma cell lines*.* Prostate 2003; 57: 226-44.
19. Pflueger D, Rickman DS, Sboner A, Perner S et al. N-myc downstream regulated gene 1 (NDRG1) is fused to ERG in prostate cancer*.* Neoplasia 2009; 11: 804-11.
20. Tomlins SA, Rhodes DR, Perner S, Dhanasekaran SM. Recurrent fusion of TMPRSS2 and ETS transcription factor genes in prostate cancer*.* Science 2005; 310: 644-8.
21. Tomlins SA, Laxman B, Dhanasekaran SM. Distinct classes of chromosomal rearrangements create oncogenic ETS gene fusions in prostate cancer*.* Nature 2007; 448: 595-9.
22. Tomlins SA, Mehra R, Rhodes DR, Smith LR et al. TMPRSS2:ETV4 gene fusions define a third molecular subtype of prostate cancer*.* Cancer Res 2006; 66: 3396-400.
23. Gronberg H. Prostate cancer epidemiology*.* Lancet 2003; 361: 859-64.
24. Epis MR, Giles KM, Barker A, Kendrick TS, Leedman PJ. miR-331-3p regulates ERBB-2 expression and androgen receptor signaling in prostate cancer. J Biol Chem. 2009;284:24696–704.
25. Li T, Li D, Sha J, Sun P, Huang Y. MicroRNA-21 directly targets MARCKS and promotes apoptosis resistance and invasion in prostate cancer cells. Biochem Biophys Res Commun.2009;383:280–5.
26. Li T, Li D, Sha J, Sun P, Huang Y. MicroRNA-21 directly targets MARCKS and promotes apoptosis resistance and invasion in prostate cancer cells. Biochem Biophys Res Commun. 2009;383:280–5.
27. Spahn M, Kneitz S, Scholz CJ, Nico S, Rudiger T, Strobel P et al. Expression of microRNA-221 is progressively reduced in aggressive prostate cancer and metastasis and predicts clinical recurrence. Int J Cancer. 2009;7(4):30–1.
28. Noonan EJ, Place RF, Pookot D, Basak S, Whitson JM, Hirata H, et al. miR-449a targets HDAC-1 and induces growth arrest in prostate cancer. Oncogene. 2009;28:1714–24.
29. Bonci D, Coppola V, Musumeci M, Addario A, Giuffrida R, Memeo L, et al. The miR-15a-miR-16-1 cluster controls prostate cancer by targeting multiple oncogenic activities. Nat Med.2008; 14:1271–7.
30. Ozen M, Creighton CJ, Ozdemir M, Ittmann M. Widespread deregulation of microRNA expression in human prostate cancer. Oncogene. 2008;27:1788–93.
31. Gandellini P, Folini M, Zaffaroni N. Towards the definition of prostate cancer-related microRNAs: where are we now? Trends Mol Med. 2009;15:381–90.
32. Shaw G, Prowse DM. Inhibition of androgen-independent prostate cancer cell growth is enhanced by combination therapy targeting Hedgehog and ErbB signalling. Cancer Cell Int. 2008*;* 8:3.
33. Marian CO, Shay JW. Prostate tumor-initiating cells: a new target for telomerase inhibition therapy? Biochim. Biophys. Acta*.* 2009;1792:289–96.
34. Fu Y, *et al.* Pten null prostate tumorigenesis and AKT activation are blocked by targeted knockout of ER chaperone GRP78/BiP in prostate epithelium. Proc. Natl. Acad. Sci. U. S. A*.* 2008;105:19444–9.
35. Zhang ZX, Xu QQ, Huang XB, Zhu JC, Wang XF. Early and delayed castrations confer a similar survival advantage in TRAMP mice. Asian J. Androl. 2009; 11:291–7.
36. Sarkar FH, Li Y, Wang Z, Kong D. Novel targets for prostate cancer chemoprevention. Endocr. Relat. Cancer. 2010; 17:R195–212.
37. Di Lorenzo G, *et al.* Expression of epidermal growth factor receptor correlates with disease relapse and progression to androgen-independence in human prostate cancer. Clin. Cancer Res*.* 2002; 8:3438–44.
38. Hammarsten P, *et al.* Low levels of phosphorylated epidermal growth factor receptor in nonmalignant and malignant prostate tissue predict favorable outcome in prostate cancer patients. Clin. Cancer Res*.* 2010; 16:1245–55.
39. Koumakpayi IH, Le Page C, Mes-Masson AM, Saad F. Hierarchical clustering of immunohistochemical analysis of the activated ErbB/PI3K/Akt/NF-kappaB signalling pathway and prognostic significance in prostate cancer. *Br.J. Cancer.* 2010; 102:1163–73.
40. King JC, *et al.* Cooperativity of TMPRSS2-ERG with PI3-kinase pathway activation in prostate oncogenesis. Nat. Genet 2009*;* 41:524–6.
41. Bismar TA, *et al.* PTEN genomic deletion is an early event associated with ERG gene rearrangements in prostate cancer. BJU Int*.* 2011; 107:477–85.
42. Ao M, *et al.* Cross-talk between paracrine acting cytokine and chemokine pathways promotes malignancy in benign human prostatic epithelium. *Cancer Res.* 2007; 67:4244–53.
43. Giannoni E, *et al.* Reciprocal activation of prostate cancer cells and cancer-associated fibroblast stimulates epithelial-mesenchymaltransition and cancer stemness. *Cancer Res.* 2010; 70:6945–56.
44. van den Hoogen C, *et al.* High aldehyde dehydrogenase activity identifies tumor-initiating and metastasis-initiating cells in human prostate cancer. *Cancer Res.* 2010; 70:5163–73.
45. Tokar EJ, Ancrile BB, Cunha GR, Webber MM. Stem/progenitor and intermediate cell types and origin of human prostate cancer. Differentiation. 2005; 73:463–73.
46. Zhou Z, et al*.* Synergy of p53 and Rb deficiency in a conditional mouse model for metastatic

prostate cancer. Cancer Res*.* 2006; 66:7889–98.

1. Bisson I, Prowse DM. WNT signaling regulates self-renewal and differentiation o prostate cancer cells with stem cell characteristics. Cell Res*.* 2009; 19:683–97.
2. Song H, et al*.* Loss of Nkx3.1 leads to the activation of discrete downstream target genes during prostate tumorigenesis. Oncogene*.* 2009; 28:3307–19.
3. Couto SS, et al*.* Simultaneous haploinsufficiency of Pten and Trp53 tumor suppressor genes accelerates tumorigenesis in a mouse model of prostate cancer. Differentiation*.* 2009; 77:103–11.
4. Zhang S, et al*.* Chemokine CXCL12 and its receptor CXCR4 expression are associated with perineural invasion of prostate cancer. J. Exp.Clin. Cancer Res. 2008*;* 27:62.
5. Skvortsova I, *et al.* Intracellular signaling pathways regulating radioresistance of human prostate carcinoma cells. Proteomics*.* 2008; 8:4521–33.
6. Narita S, *et al.* GLI2 knockdown using an antisense oligonucleotide induces apoptosis and chemosensitizes cells to paclitaxel in androgen- independent prostate cancer. *Clin. Cancer Res.* 2008; 14:5769–77.
7. Zhao H, Peehl DM. Tumor-promoting phenotype of CD90hi prostate cancer-associated fibroblasts. *Prostate.* 2009; 69:991–1000.
8. Chen M, *et al.* Hedgehog/Gli support androgen signaling in androgen deprived and androgen independent prostate cancer cells. *Mol. Cancer.* 2010; 9:89.
9. Giannoni E, Fiaschi T, Ramponi G, Chiarugi P. Redox regulation of anoikis resistance of metastatic prostate cancer cells: key role for Src and EGFR-mediated pro-survival signals. Oncogene*.* 2009; 28:2074–86.
10. Higgins LH, *et al.* Hypoxia and the metabolic phenotype of prostate cancer cells. *Biochim. Biophys. Acta* 2009; 1787:1433–43.
11. Ben Sahra I, *et al.* Targeting cancer cell metabolism: the combination of metformin and 2-deoxyglucose induces p53-dependent apoptosis in prostate cancer cells. Cancer Res*. 2010;* 70:2465–75.
12. DeHaan AM, Wolters NM, Keller ET, Ignatoski KM. EGFR ligand switch in late stage prostate cancer contributes to changes in cell signaling and bone remodeling. *Prostate.* 2009; 69:528–37.
13. Bednarz N, *et al.* BRCA1 loss preexisting in small subpopulations of prostate cancer is associated with advanced disease and metastatic spread to lymph nodes and peripheral blood. Clin. Cancer Res. 2010; 16:3340–8.
14. Morrissey C, Vessella RL. The role of tumor microenvironment in prostate cancer bone metastasis. J. Cell. Biochem*.* 2007; 101:873–86.
15. Chen SJ, *et al.* Prostate-derived factor as a paracrine and autocrine factor for the proliferation of androgen receptor-positive human prostate cancer cells. Prostate*.* 2007; 67:557–71.
16. Senapati S, *et al.* Overexpression of macrophage inhibitory cytokine-1 induces metastasis of human prostate cancer cells through the FAK-RhoA signaling pathway. Oncogene*.* 2010; 9:1293–302.
17. Engl T, *et al.* CXCR4 chemokine receptor mediates prostate tumor cell adhesion through alpha5 and beta3 integrins. Neoplasia*.* 2006; 8:290–301.
18. Jung Y, *et al.* (2009) Expression of PGK1 by prostate cancer cells induces bone formation. *Mol. Cancer Res.* 7:1595–604.
19. Wakchoure S, *et al.* Expression of macrophage inhibitory cytokine-1 in prostate cancer bone metastases induces osteoclast activation and weight loss. Prostate*.* 2009; 69:652–61.
20. Coulson-Thomas VJ, *et al.* Fibroblast and prostate tumor cell cross-talk: fibroblast differentiation, TGF-beta, and extracellular matrix down-regulation. Exp. Cell Res*.* 2010; 316:3207–26.
21. Josson S, Matsuoka Y, Chung LW, Zhau HE, Wang R Tumor-stroma co-evolution in prostate cancer progression and metastasis. Semin. Cell. Dev. Biol*,* 2010; 21:26–32.
22. Altieri DC, *et al.* Prostate cancer regulatory networks. J. Cell. Biochem*.* 2009; 107:845–52.
23. Tang Y, *et al.* The relationship of neuroendocrine carcinomas to anti-tumor therapies in TRAMP mice. Prostate. 2009, 69:1763–73.
24. Tang Y, *et al.* Divergent effects of castration on prostate cancer in TRAMP mice: possible implications for therapy. Clin. Cancer Res*.* 2008; 14:2936–43.
25. Banach-Petrosky W, *et al.* Prolonged exposure to reduced levels of androgen accelerates prostate cancer progression in Nkx3.1; Pten mutant mice. Cancer Res*.* 2007; 67:9089–96.
26. Basu HS, *et al.* A small molecule polyamine oxidase inhibitor blocks and androgen induced oxidative stress and delays prostate cancer progression in the transgenic adenocarcinoma of the mouse prostate model. Cancer Res*.* 2009; 69:7689–95.
27. Volate SR, *et al.* Gossypol induces apoptosis by activating p53 in prostate cancer cells and prostate tumor-initiating cells. Mol. Cancer 2010; *Ther.* 9:461–70.
28. Marian CO, Wright WE, Shay JW. The effects of telomerase inhibition on prostate tumor-initiating cells. Int. J. Cancer*.* 2010; 127:321–31.
29. Sharma A, et al. The retinoblastoma tumor suppressor controls androgen signaling and human prostate cancer progression. J Clin Invest*.* 2010; 120:4478–4492.
30. Shen MM, Abate-Shen C. Molecular genetics of prostate cancer: new prospects for old challenges.

Genes Dev. 2010; 24:1967–2000.

1. Taylor BS, et al. Integrative genomic profiling of human prostate cancer. Cancer Cell. 2010;18:11–22.
2. Foster CS, et al. Transcription factor E2F3 over expressed in prostate cancer independently predicts clinical outcome. Oncogene. 2004; 23:5871–5879.
3. Inaba Y, Ohyama C, Kato T, Satoh M, Saito H, Hagisawa S, et al. Gene transfer of alpha1,3-fucosyltransferase increases tumor growth of the PC-3 human prostate cancer cell line through enhanced adhesion to prostatic stromal cells. Int J Cancer. 2003; 107:949–957.
4. Sun YX, Wang J, Shelburne CE, Lopatin DE, Chinnaiyan AM, Rubin MA, et al. Expression of CXCR4 and CXCL12 (SDF-1) in human prostate cancers (PCa) in vivo. J Cell Biochem. 2003; 89:462–473.
5. Wang X, Ferreira AM, Shao Q, Laird DW, Sandig M. Beta3 integrins facilitate matrix interactions during transendothelial migration of PC3 prostate tumor cells. Prostate. 2005; 63:65–80.
6. Fornaro M, Manes T, Languino LR. Integrins and prostate cancer metastases. Cancer Metastasis Rev. 2001; 20:321–331.
7. Feng P, Li TL, Guan ZX, Franklin RB, Costello LC. Direct effect of zinc on mitochondrial apopto genesis in prostate cells. Prostate 2002, 52:311–318.
8. Chua CW, Chiu YT, Yuen HF, Chan KW, Man K, Wang X, et al. Suppression of androgen-independent prostate cancer cell aggressiveness by FTY720: validating Runx2 as a potential antimetastatic drug screening platform. Clin Cancer Res 2009, 15:4322–4335.
9. van der Deen M, Akech J, Wang T, FitzGerald TJ, Altieri DC, Languino LR, et al. The cancer-related Runx2 protein enhances cell growth and responses to androgen and TGFbeta in prostate cancer cells. J Cell Biochem 2010, 109:828–837.
10. Robertson BW, Chellaiah MA. Osteopontin induces beta-catenin signaling through activation of Akt in prostate cancer cells. Exp Cell Res 2010, 316:1–11.
11. Robertson BW, Bonsal L, Chellaiah MA. Regulation of Erk1/2 activation by osteopontin in PC3 human prostate cancer cells. Mol Cancer 2010, 9:260.
12. Desai B, Rogers MJ, Chellaiah MA. Mechanisms of osteopontin and CD44 as metastatic principles in prostate cancer cells. Mol Cancer 2007, 6:18.
13. Akech J, Wixted JJ, Bedard K, van der Deen M, Hussain S, Guise TA, et al. Runx2 association with progression of prostate cancer in patients: mechanisms mediating bone osteolysis and osteoblastic metastatic lesions. Oncogene 2010, 29:811–821.
14. S. Srinivasan, R. Kumar, S. Koduru, A. Chandramouli, and C. Damodaran, Inhibiting TNF-mediated signaling: a novel therapeutic paradigm for androgen independent prostate cancer. *Apoptosis*, 2010; 15: 153–161.
15. Mengmeng Liang and David J Mulholland. Lipogenic metabolism: a viable target for prostate cancer treatment? Asian J Androl. 2014 Sep-Oct; 16(5): 661–663.
16. Huang WC, Li X, Liu J, Lin J, Chung LW. Activation of androgen receptor, lipogenesis, and oxidative stressconverged by SREBP1 is responsible for regulating growth andprogression of prostate cancer cells. Mol Cancer Res. 2012 Jan;10(1):133-42.
17. Shiota M, Yokomizo A, Naito S. [Oxidative stress and androgen receptor signaling in the development and progression of castration-resistant prostate cancer.](http://www.ncbi.nlm.nih.gov/pubmed/21820046) Free Radic Biol Med. 2011 Oct 1;51(7):1320-8.
18. Krycer JR, Brown AJ. Cholesterol accumulation in prostate cancer: a classic observation from a modern perspective. Biochim Biophys Acta. 2013 Apr;1835(2):219-29.
19. Lewis SR, Hedman CJ, Ziegler T, Ricke WA. Steroidogenic factor 1 promotes aggressive growth of castration-resistantprostate cancer cells by stimulating steroid synthesis and cell proliferation. Endocrinology. 2014 Feb;155(2):358-69.
20. Chang KH, Li R, Papari-Zareei M.Dihydrotestosterone synthesis bypasses testosterone to drive castration-resistant prostate cancer. Proc Natl Acad Sci U S A. 2011 Aug 16;108(33):13728-33.
21. Lamont KR, Tindall DJ. Minireview: Alternative activation pathways for the androgen receptor in prostate cancer. Mol Endocrinol. 2011 Jun;25(6):897-907.
22. Feldman B.J. & Feldman D. The development of androgen-independent prostate cancer. Nat Rev Cancer 1, 34-45 (2001).
23. Vinall RL, Mahaffey CM, Davis RR.  Dual blockade of PKA and NF-κB inhibits H2 relaxin-mediated castrate resistant growth of prostate cancer sublines and induces apoptosis.Horm Cancer. 2011 Aug;2(4):224-38.
24. Parray A., Siddique H.R., Nanda S., Konety B.R. & Saleem M. Castration-resistant prostate cancer: potential targets and therapies. Biologics 6, 267-276 (2012).
25. Patel JC, Maughan BL. Emerging molecularly targeted therapies in castration refractory prostate cancer. Prostate Cancer. 2013;2013:981684.
26. Inoue T. & Ogawa O. Role of signaling transduction pathways in development of castration-resistant prostate cancer. Prostate Cancer 2011, 647987 (2011).
27. Karantanos T., Corn P.G. &Thompson T.C. Prostate cancer progression after androgen deprivation therapy: mechanisms of castrate resistance and novel therapeutic approaches. Oncogene 32, 5501-5511 (2013).
28. Lorente D. & De Bono J.S. Molecular alterations and emerging targets in castration resistant prostate cancer. Eur J Cancer 50, 753-764 (2014).
29. Liao RS, Ma S. Androgen receptor-mediated non-genomic regulation of prostate cancercell proliferation. Transl Androl Urol. 2013 Sep;2(3):187-96.
30. Schweizer MT, Yu EY. Persistent androgen receptor addiction in castration-resistant prostate cancer. J Hematol Oncol. 2015 Nov 13;8:128.
31. Devlin H.L. & Mudryj M. Progression of prostate cancer: multiple pathways to androgen independence. Cancer Lett 274, 177-186 (2009).
32. Lamont KR, Tindall DJ. Minireview: Alternative activation pathways for the androgen receptor in prostate cancer. Mol Endocrinol. 2011 Jun;25(6):897-907.
33. Heemers HV, Tindall DJ. Androgen receptor (AR): a diversity of functions converging on and regulating the AR transcriptional complex. Endocr Rev. 2007 Dec;28(7):778-808.
34. Fiaschi T, Giannoni E, Taddei ML, Cirri P, Marini A. Pintus G, Nativi C. Carbonic anhydrase IX from cancer-associated fibroblasts drives epithelial mesenchymal transition in prostate carcinoma cells. Cell Cycle. 2013 Jun 1;12(11):1791-801.
35. Yu S, Xia S, Yang D, Wang K, Yeh S, Gao Z, Chang C. Androgen receptor in human prostate cancer-associated fibroblastspromotes prostate cancer epithelial cell growth and invasion.MedOncol. 2013)674.
36. Taddei ML, [Cavallini L](http://www.ncbi.nlm.nih.gov/pubmed/?term=Cavallini L%5BAuthor%5D&cauthor=true&cauthor_uid=25091736). Senescent stroma promotes prostate cancer progression: the role of miR-210. Mol Oncol. 2014 Dec;8(8):1729-46.
37. Leach DA, Need EF, Trotta AP, Grubisha MJ. Hic-5 influences genomic and non-genomic actions of the androgenreceptor in prostate myofibroblasts. Mol Cell Endocrinol. 2014 Mar 25;384(1-2):185-99.
38. Ammirante M, Shalapour S, Kang Y, Jamieson CA, Karin M. Tissue injury and hypoxia promote malignant progression of prostatecancer by inducing CXCL13 expression in tumor myofibroblasts. Proc Natl Acad Sci U S A. 2014 Oct 14;111(41):14776-81.
39. Webber JP, Spary LK, Sanders AJ, Webber JP, Spary LK, Sanders AJ, Steadman R. Differentiation of tumour-promoting stromal myofibroblasts by cancerexosomes. Oncogene. 2015 Jan 15;34(3):290-302.
40. [Sampson N](http://www.ncbi.nlm.nih.gov/pubmed/?term=Sampson N%5BAuthor%5D&cauthor=true&cauthor_uid=23720424), [Zenzmaier C](http://www.ncbi.nlm.nih.gov/pubmed/?term=Zenzmaier C%5BAuthor%5D&cauthor=true&cauthor_uid=23720424), [Heitz M](http://www.ncbi.nlm.nih.gov/pubmed/?term=Heitz M%5BAuthor%5D&cauthor=true&cauthor_uid=23720424), [Hermann M](http://www.ncbi.nlm.nih.gov/pubmed/?term=Hermann M%5BAuthor%5D&cauthor=true&cauthor_uid=23720424).  Stromal insulin-like growth factor binding protein 3 (IGFBP3) is elevated in the diseased human prostate and promotes ex vivo fibroblastmyofibroblast differentiation. [Endocrinology.](http://www.ncbi.nlm.nih.gov/pubmed/?term=Stromal+insulin-like+growth+factor+binding+protein+3+(IGFBP3)+is+elevated+in+the+diseased+human+prostate+and+promotes+ex+vivo+fibroblast-to-myofibroblast+differentiation.) 2013 Aug;154(8):2586-99.
41. Toren P, Venkateswaran V. Periprostatic adipose tissue and prostate cancer progression: new insights into the tumor microenvironment. [Clin Genitourin Cancer.](http://www.ncbi.nlm.nih.gov/pubmed/24269373) 2014 Feb;12(1):21-6.

# Ribeiro R, Lopes C, Medeiros R. The**link**between**obesity**and**prostate cancer**:the**leptin pathway**and **therapeutic perspectives**. **Prostate Cancer**Prostatic Dis. 2006;9(1):19-24.

1. Barron DA, Rowley DR.The reactive stroma microenvironment and prostate cancer progression. [Endocr Relat Cancer.](http://www.ncbi.nlm.nih.gov/pubmed/22930558) 2012 Oct 30;19(6):R187-204.

# [**Condon MS**](http://www.ncbi.nlm.nih.gov/pubmed/?term=Condon MS%5BAuthor%5D&cauthor=true&cauthor_uid=15652458). The role of the stromal microenvironment in prostate cancer. [**Semin Cancer Biol.**](http://www.ncbi.nlm.nih.gov/pubmed/15652458)2005 Apr;15(2):132-7.

1. Lundholm M, Schröder M, Nagaeva O, Baranov V, Widmark A, Mincheva-Nilsson L. Prostate tumor derived exosomes downregulate NKG2D expression onnatural killer cells and CD8+ T cells: mechanim of immune evasion. PLoS One. 2014 Sep 30;9(9):e108925.
2. Fujita K, Ewing CM, Isaacs WB. Immunomodulatory IL-18 binding protein is produced by prostate cancer cells and its levels in urine and serum correlate with tumor status. Int J Cancer. 2011 Jul 15;129(2):424-32.
3. Spary LK, Salimu J, Webber JP. Tumor stroma-derived factors skew monocyte to dendritic cell differentiation toward a suppressive CD14+ PD-L1+ phenotype in prostate cancer. Oncoimmunology. 2014 Dec 13;3(9):e955331.
4. Barach YS, Lee JS, Zang X. T cell coinhibition in prostate cancer: new immune evasion pathways and emerging therapeutics. Trends Mol Med. 2011 Jan;17(1):47-55.
5. Choi SY, Gout PW, Collins CC. Epithelial immune cell-like transition (EIT): a proposed transdifferentiation process underlying immune-suppressive activity of epithelial cancers. Differentiation. 2012 Jun;83(5):293-8.
6. Rajarubendra N, Lawrentschuk N, Bolton DM. Prostate cancer immunology - an update for Urologists. BJU Int. 2011 Apr;107(7):1046-51.
7. Silvestri I, Cattarino S, Aglianò AM. Beyond the Immune Suppression: The Immunotherapy in Prostate Cancer. Biomed Res Int. 2015;2015:794968.
8. Thakur A, Vaishampayan U, Lum LG. Immunotherapy and immune evasion in prostate cancer. Cancers (Basel). 2013 May 24;5(2):569-90.
9. Angèle S, Falconer A, Foster CS. ATM protein overexpression in prostate tumors: possible role in telomere maintenance. Am J Clin Pathol. 2004 Feb;121(2):231-6.
10. Wetterau LA, Francis MJ, Ma L, Cohen P. Insulin-like growth factor I stimulates telomerase activity in prostate cancer cells. J Clin Endocrinol Metab. 2003 Jul;88(7):3354-9.
11. Matsumura Y, Shimada K, Tanaka N, Fujimoto K, Hirao Y, Konishi N. Phosphorylation status of Fas-associated death domain-containing protein regulates telomerase activity and strongly correlates with prostate cancer outcomes. Pathobiology. 2009;76(6):293-302.
12. Ding Z, Wu CJ, Jaskelioff M, Ivanova E, Kost-Alimova M, Protopopov A. Telomerase reactivation following telomere dysfunction yields murine prostate tumors with bone metastases. Cell. 2012 Mar 2;148(5):896-907.
13. Biroccio A, Leonetti C. Telomerase as a new target as a new target for the treatment of hormone-refractory prostate cancer. Endocr Relat Cancer. 2004 Sep;11(3):407-21.
14. Wullich B, Rohde V, Oehlenschläger B, Bonkhoff H, Ketter R, Zwergel T, Sattler HP. Focal intratumoral heterogeneity for telomerase activity in human prostate cancer. J Urol. 1999 Jun;161(6):1997-2001.
15. Xu T, He K, Wang L, Goldkorn A. Prostate tumour cells with cancer progenitor properties have high telomerase activity and are rapidly killed by telomerase interference. Prostate. 2011 Sep 15;71(13):1390-400.
16. Elmore LW, Forsythe R, Forsythe H, Bright AT, Nasim S, Endo K, Holt SE. Overexpression of telomerase-associated chaperone proteins in prostatic intraepithelial neoplasia and carcinomas. Oncol Rep. 2008 Sep;20(3):613-7.
17. Moehren U, Papaioannou M, Reeb CA, Grasselli A, Nanni S, Asim M, Roell D, Prade I, Farsetti A, Baniahmad A. Wild-type but not mutant androgen receptor inhibits expression of the hTERT telomerase subunit : a novel role for AR mutation for prostate cancer development. FASEB J. 2008 Apr;22(4):1258-67.
18. Matuszak EA, Kyprianou N. Androgen regulation of epithelial-mesenchymal transition in prostate tumorigenesis. Expert Rev Endocrinol Metab. 2011 May;6(3):469-482.
19. Shiota M, Bishop JL, Nip KM, Zardan A, Takeuchi A, Cordonnier T, Beraldi E, Bazov J, Fazli L, Chi K, Gleave M, Zoubeidi A. Hsp27 regulates epithelial mesenchymal transition, metastasis and circulating tumour cells in prostate cancer. Cancer Res. 2013 May 15;73(10):3109-19.
20. Li P, Yang R, Gao WQ. Contributions of epithelial-mesenchymal transition and cancer stem cells to the development of castration resistance of prostate cancer. Mol Cancer. 2014 Mar 12;13:55.
21. Zhang S, Wang X, Osunkoya AO, Iqbal S, Wang Y, Chen Z. EPLIN downregulation promotes epithelial-mesenchymal transition in prostate cancer cells and correlates with clinical lymph node metastasis. Oncogene. 2011 Dec 15;30(50):4941-52.
22. Campbell M. Grant, Natasha Kyprianou. Epithelial mesenchymal transition (EMT) in prostate growth and tumour progression. Transl Androl Urol 2013;2(3):202-211.
23. Xie D, Gore C, Liu J, Pong RC, Mason R, Hao G, Long M, Kabbani W, Yu L, Zhang H, Chen H, Sun X, Boothman DA, Min W, Hsieh JT. Role of DAB2IP in modulating epithelial- to-mesenchymal transition and prostate cancer metastasis. Proc Natl Acad Sci U S A. 2010 Feb 9;107(6):2485-90.
24. Martin P^1^, Liu YN, Pierce R, Abou-Kheir W, Casey O, Seng V, Camacho D, Simpson RM, Kelly K. Prostate epithelial Pten/TP53 loss leads to transformation multipotential progenitors and epithelial to mesenchymal transition. Am J Pathol. 2011 Jul;179(1):422-35.
25. Ren D^1^, Wang M, Guo W, Zhao X, Tu X, Huang S, Zou X, Peng X. Wild- type P53 suppresses the epithelial-mesenchymal transition and stemness in PC-3 prostate cancer cells by modulating miR-145. [Int J Oncol.](http://www.ncbi.nlm.nih.gov/pubmed/?term=Int+J+Oncol.+2013+Apr%3B42(4)%3A1473-81.) 2013 Apr;42(4):1473-81.
26. Zaman MS^1^, Chen Y, Deng G, Shahryari V, Suh SO, Saini S, Majid S, Liu J, Khatri G, Tanaka Y, Dahiya R.The functional significance of microRNA-145 in prostate cancer. Br J Cancer. 2010 Jul 13;103(2):256-64.
27. Smit MA^1^, Peeper DS. Deregulating EMT and senescence: double impact by a single twist. Cancer Cell. 2008 Jul 8;14(1):5-7.
28. Mulholland DJ^1^, Kobayashi N, Ruscetti M, Zhi A, Tran LM, Huang J, Gleave M, Wu H. Pten loss and RAS/MAPK activation cooperate to promote EMT and metastasis initiated from prostate cancer stem/progenitor cells. Cancer Res. 2012 Apr 1;72(7):1878-89.
29. Lue HW^1^, Yang X, Wang R, Qian W, Xu RZ, Lyles R, Osunkoya AO, Zhou BP, Vessella RL, Zayzafoon M, Liu ZR, Zhau HE,Chung LW. LIV-1 promotes prostate cancer epithelial-to-mesenchymal transition and metastasis through HB-EGF shedding and EGFR-mediated ERK signaling. PLoS One. 2011;6(11):e27720.
30. Wang C^1^, Wang L, Su B, Lu N, Song J, Yang X, Fu W, Tan W, Han B. Serine protease inhibitor Kazal type 1 promotes epithelial-mesenchymal transition throygh EGFR signaling pathway in prostate cancer. Prostate. 2014 May;74(7):689-701.
31. Acevedo VD^1^, Gangula RD, Freeman KW, Li R, Zhang Y, Wang F, Ayala GE, Peterson LE, Ittmann M, Spencer DM. Inducible FGFR-1 activation leads to irreversible prostate adenocarcinoma and an epithelial-to-mesenchymal transition. Cancer Cell. 2007 Dec;12(6):559-71.
32. Sun Y^1^, Wang BE, Leong KG, Yue P, Li L, Jhunjhunwala S, Chen D, Seo K, Modrusan Z, Gao WQ, Settleman J, Johnson L. Androgen deprivation causes epithelial-mesenchymal transition in the prostate: implications for androgen deprivation therapy. Cancer Res. 2012 Jan 15;72(2):527-36.
33. El-Amm J^1^, Freeman A, Patel N, Aragon-Ching JB. Bone-Targeted Therapies in Metastatic Castration-Resistant Prostate Cancer: Evolving Paradigms. Prostate Cancer. 2013;2013:210686.
34. Dolloff NG^1^, Shulby SS, Nelson AV, Stearns ME, Johannes GJ, Thomas JD, Meucci O, Fatatis A. Bone-metastatic potential of human prostate cancer cells correlates with Akt/PKB activation by alpha platelet-derived growth factor receptor. Oncogene. 2005 Oct 13;24(45):6848-54.
35. Rubens RD. Bone metastases—the clinical problem. Eur J Cancer. 1998 Feb;34(2):210-3.
36. Yan Z^1^, Jin S^2^, Wei Z^3^, Huilian H^4^, Zhanhai Y^5^, Yue T^1^, Juan L^1^, Jing L^1^, Libo Y^2^, Xu L^1^. Discoidin domain receptor 2 facilitates prostate cancer bone metastasis via regulating parathyroid hormone-related protein. Biochim Biophys Acta. 2014 Apr 27.
37. Jin JK^1^, Dayyani F, Gallick GE. Steps in prostate cancer progression that lead to bone metastasis. Int J Cancer. 2011 Jun 1;128(11):2545-61.
38. Keller ET^1^, Brown J. Prostate cancer bone metastases promote both osteolytic and osteoblastic activity. J Cell Biochem. 2004 Mar 1;91(4):718-29.
39. Roberts E^1^, Cossigny DA^1^, Quan GM^2^. The Role of Vascular Endothelial Growth Factor in Metastatic Prostate Cancer to The Skeleton. Prostate Cancer. 2013;2013:418340.
40. Morrissey C^1^, Vessella RL. The role of tumour microenvironment in prostate cancer bone metastasis. J Cell Biochem. 2007 Jul 1;101(4):873-86.
41. Kang HY^1^, Huang HY, Hsieh CY, Li CF, Shyr CR, Tsai MY, Chang C, Chuang YC, Huang KE. Activin A enhances prostate cancer cell migration through activation of androgen receptor and is overexpressed in metastatic prostate cancer. J Bone Miner Res. 2009 Jul;24(7):1180-93.
42. Alessandro Sciarra, Gianna Mariotti, Stefano Salciccia Ana Autran Gomez. Prostate growth and inflammation. Journal of Steroid Biochemistry & Molecular Biology 108 (2008)254–260.
43. Sfanos KS,  De Marzo AM. Prostate cancer and inflammation: the evidence. Histopathology. 2012 Jan;60(1):199-215.
44. De Marzo AM^1^, Platz EA, Sutcliffe S, Xu J, Grönberg H, Drake CG, Nakai Y, Isaacs WB, Nelson WG. Inflammation in in prostate carcinogenesis. Nat Rev Cancer. 2007 Apr;7(4):256-69.
45. Veeranki S. Role of inflammasomes and their regulators in prostate cancer initiation, progression and metastasis. Cell Mol Biol Lett. 2013 Sep;18(3):355-67.
46. Kwon EM^1^, Salinas CA, Kolb S, Fu R, Feng Z, Stanford JL, Ostrander EA. Genetic polymorphisms in inflammation pathway genes and prostate cancer risk. Cancer Epidemiol Biomarkers Prev. 2011 May;20(5):923-33.
47. Nguyen DP^1^, Li J, Yadav SS, Tewari AK. Recent insights into NF-κB signaling pathways and the link between inflammation and prostate cancer. BJU Int. 2013 Oct 8.
48. Garg R^1^, Blando J, Perez CJ, Wang H, Benavides FJ, Kazanietz MG. Activation of nuclear factor κB (NF-κB ) in prostate cancer is mediated by protein kinase C epsilon (PKC epsilon) . J Biol Chem. 2012 Oct 26;287(44):37570-82.
49. Sweeney C^1^, Li L, Shanmugam R, Bhat-Nakshatri P, Jayaprakasan V, Baldridge LA,  [Gardner T](http://www.ncbi.nlm.nih.gov/pubmed?term=Gardner T%5BAuthor%5D&cauthor=true&cauthor_uid=15328189), [Smith M](http://www.ncbi.nlm.nih.gov/pubmed?term=Smith M%5BAuthor%5D&cauthor=true&cauthor_uid=15328189), Nakshatri H, Cheng L. Nuclear factor-kappaB is constitutively activated in prostate cancer in vitro and is overexpressed in prostatic intraepithelial neoplasia and adenocarcinoma of the prostate. Clin Cancer Res. 2004 Aug 15;10(16):5501-7.
50. Wong CP^1^, Bray TM, Ho E. Induction of proinflammatory response in prostate cancer epithelial cells by activated macrophages. Cancer Lett. 2009 Apr 8;276(1):38-46.
51. Bohonowych JE^1^, Hance MW, Nolan KD, Defee M, Parsons CH, Isaacs JS. Extracellular Hsp90 mediates an NF-κB dependent inflammatory stromal program: implications for the prostate tumour microenvironment. Prostate. 2014 Apr;74(4):395-407.
52. Mimeault M^1^, Batra SK. Development of animal models underlining mechanistic connections between prostate inflammation and cancer. World J Clin Oncol. 2013 Feb 10;4(1):4-13.
53. Kwon OJ^1^, Zhang L, Ittmann MM, Xin L. Prostatic inflammation enhances basal-to-luminal differentiation and accelerates initiation of prostate cancer with a basal cell origin. Proc Natl Acad Sci U S A. 2014 Feb 4;111(5):E592-600.
54. Fang LY^1^, Izumi K,  Lai KP,  Liang L,  Li L,  Miyamoto H,  Lin WJ,  Chang C. Infiltrating macrophages promotes prostate tumorigenesis via modulating androgen receptor- mediated CCL4-STAT3 signaling. Cancer Res. 2013 Sep 15;73(18):5633-46.
55. Khandrika L^1^, Kumar B, Koul S, Maroni P, Koul HK. Oxidative stress in prostate cancer. [Cancer Lett.](http://www.ncbi.nlm.nih.gov/pubmed/?term=Role+of+Oxidative+Stress+in+Prostate+Cancer%2C+Cancer+Lett.+2009) 2009 Sep 18;282(2):125-36.
56. Debelec-Butuner B^1^, Alapinar C, Varisli L, Erbaykent-Tepedelen B, Hamid SM, Gonen-Korkmaz C, Korkmaz KS. Inflammation mediated abrogation of androgen signaling: an invitro model of prostate cell inflammation. Mol Carcinog. 2014 Feb;53(2):85-97.
57. Folkman J. Endogeneous angiogenesis inhibitors. APMIS. 2004 Jul-Aug;112(7-8):496-507.
58. Russo G, Mischi M, Scheepens W, De la Rosette JJ, Wijkstra H. Angiogenesis in prostate cancer: onset, progression and imaging. BJU Int. 2012 Dec;110(11 Pt C):E794-808.
59. Wang J^1^, Wang J, Dai J, Jung Y, Wei CL, Wang Y, Havens AM, Hogg PJ, Keller ET, Pienta KJ, Nor JE, Wang CY, Taichman RS. A glycolytic mechanism regulating an angiogenic switch in prostate cancer. Cancer Res. 2007 Jan 1;67(1):149-59.
60. Lu S^1^, Lee J, Revelo M, Wang X, Lu S, Dong Z. Smad3 is overexpressed in advanced human prostate cancer and necessary for progressive growth in prostate cancer cells in nude mice. Clin Cancer Res. 2007 Oct 1;13(19):5692-702.
61. Strohmeyer D^1^, Rössing C, Strauss F, Bauerfeind A, Kaufmann O, Loening S. Tumour angiogenesis is associated with progression after radial prostatectomy in pT2/ pT3 prostate cancer. Prostate. 2000 Jan;42(1):26-33.
62. Volpert O^1^, Luo W, Liu TJ, Estrera VT, Logothetis C, Lin SH. Inhibition of prostate tumour angiogenesis by the tumour suppressor CEACAM1. J Biol Chem. 2002 Sep 20;277(38):35696-702.
63. Tilki D^1^, Irmak S, Oliveira-Ferrer L, Hauschild J, Miethe K, Atakaya H, Hammerer P, Friedrich MG, Schuch G, Galalae R, Stief CG, Kilic E, Huland H, Ergun S. Oncogene. 2006 Aug 17;25(36):4965-74.
64. Wang J^1^, Wang J, Dai J, Jung Y, Wei CL, Wang Y, Havens AM, Hogg PJ, Keller ET, Pienta KJ, Nor JE, Wang CY, Taichman RS. A glycolytic enzyme regulating an angiogenic switch in prostate cancer. Cancer Res. 2007 Jan 1;67(1):149-59.
65. Lu S^1^, Lee J, Revelo M, Wang X, Lu S, Dong Z. Smad3 is overexpressed in advanced human prostate cancer and necessary for progressive growth of prostate cancer cells in nude mice. [Clin Cancer Res.](http://www.ncbi.nlm.nih.gov/pubmed/?term=Clin+Cancer+Res.+2007+Oct+1%3B13(19)%3A5692-702.) 2007 Oct 1;13(19):5692-702.
66. Ferrando M^1^, Gueron G, Elguero B, Giudice J, Salles A, Leskow FC, Jares-Erijman EA, Colombo L, Meiss R, Navone N, De Siervi A, Vazquez E. Heme oxygenase 1(HO-1) challenges the angiogenic switch in prostate cancer. Angiogenesis. 2011 Dec;14(4):467-79.
67. Elguero B^1^, Gueron G, Giudice J, Toscani MA, De Luca P, Zalazar F, Coluccio-Leskow F, Meiss R, Navone N, De Siervi A, Vazquez E. Unveiling the association of STAT3 and HO-1 in prostate cancer: role beyond heme degradation. [Neoplasia.](http://www.ncbi.nlm.nih.gov/pubmed/?term=Unveiling+the+Association+of+STAT3+and+HO-1+in+Prostate+Cancer%3A+Role+beyond+Heme+Degradation) 2012 Nov;14(11):1043-56.

# Liu LZ^1^, Li C, Chen Q, Jing Y, Carpenter R, Jiang Y, Kung HF, Lai L, Jiang BH. MiR-21 induced angiogenesis through AKT and ERK activation and HIF-1α**expression**. [**PLoS One.**](http://www.ncbi.nlm.nih.gov/pubmed/?term=MiR-21+induced+angiogenesis+through+AKT+and+ERK+activation+and+HIF-1α+expression.)2011 Apr 22;6(4):e19139.

1. Gonzalez-Moreno O^1^, Lecanda J, Green JE, Segura V, [Catena R](http://www.ncbi.nlm.nih.gov/pubmed?term=Catena R%5BAuthor%5D&cauthor=true&cauthor_uid=20006606), Serrano D, Calvo A. VEGF elicits epithelial-mesenchymal transition(EMT) in prostate intraepithelial neoplasia (PIN)-like cells via an autocrine loop. Exp Cell Res. 2010 Feb 15;316(4):554-67.
2. Hanson J^1^, Gorman J, Reese J, Fraizer G. Regulation of vascular endothelial growth factor, VEGF, gene promoter by the tumour suppressor, WT1. Front Biosci. 2007 Jan 1;12:2279-90.
3. Li J^1^, Al-Azzawi F. Mechanism of androgen receptor action. [Maturitas.](http://www.ncbi.nlm.nih.gov/pubmed/19372015) 2009 Jun 20;63(2):142-8.
4. Jiang J^1^, Huang H. Targeting the Androgen Receptor by Taxol in Castration-Resistant Prostate Cancer. [Mol Cell Pharmacol.](http://www.ncbi.nlm.nih.gov/pubmed/20419056) 2010 Jan 1;2(1):1-5.
5. Arnold JT^1^, Isaacs JT. Mechanisms involved in the progression of androgen-independent prostate cancers: it is not only the cancer cell’s fault. Endocr Relat Cancer. 2002 Mar;9(1):61-73.
6. Russell PJ^1^, Bennett S, Stricker P. Growth factor involvement in the progression of prostate cancer. Clin Chem. 1998 Apr;44(4):705-23.
7. Montano X, Djamgoz MB. Epidermal growth factors, neurotrophins and the metastatic cascade in prostate cancer. FEBS Lett. 2004 Jul 30;571(1-3):1-8.
8. Paul G Corn. The tumour microenvironment in prostate cancer. Cancer Manag Res. 2012; 4: 183–193.
9. Natasha Kyprianou. TGF-beta signaling dictates therapeutic targeting in prostate cancer. Therapy (2008) 5(1), 7-11.
10. Min J, Zaslavsky A, Fedele G. An oncogene-tumor suppressor cascade drives metastatic prostate cancer by coordinately activating Ras and nuclear factor-kappaB. Nat Med. 2010 Mar;16(3):286-94.
11. Ge K^1^, Minhas F, Duhadaway J, Mao NC, Wilson D, Buccafusca R, Sakamuro D, Nelson P, Malkowicz SB, Tomaszewski J, [Prendergast GC](http://www.ncbi.nlm.nih.gov/pubmed?term=Prendergast GC%5BAuthor%5D&cauthor=true&cauthor_uid=10738240). Loss of heterozygosity and tumour suppressor activity Bin1 in prostate carcinoma. Int J Cancer. 2000 Apr 15;86(2):155-61.
12. Kreeger PK, Lauffenburger DA. Cancer systems biology: a network modeling perspective. Carcinogenesis. 2010 Jan;31(1):2-8.
13. Luo J, Solimini NL, Elledge SJ. Principles of cancer therapy: oncogene and non oncogene addiction. Cell. 2009 Mar 6;136(5):823-37.
14. Mazaris E, Tsiotras A. Molecular pathways in prostate cancer. Nephrourol Mon. 2013 Jul 1;5(3):792-800.
15. Laubenbacher R, Hower V, Jarrah A, Torti SV, Shulaev V, Mendes P, Torti FM, Akman S. A systems biology view of cancer. Biochim Biophys Acta. 2009 Dec;1796(2):129-39.
16. von Clausewitz, C. On War. (Ed./trans. M. Howard and P. Paret.) (Alfred A. Knopf, "Everyman's Library" edition, New York, NY, 1993)
17. Csermely P, Agoston V, Pongor S. The efficiency of multi-target drugs: the network approach might help drug design. Trends Pharmacol Sci. 2005 Apr;26(4):178-82.
18. Azmi AS. Network pharmacology for future drug discovery: are we there yet? Future Med Chem. 2012 May;4(8):939-41.
19. Haura EB. From modules to medicine: How modular domains and their associated networks can enable personalized medicine. FEBS Lett. 2012 Aug 14;586(17):2580-5.
20. Pujol A, Mosca R, Farrés J, Aloy P. Unveiling the role of network and systems biology in drug discovery. Trends Pharmacol Sci. 2010 Mar;31(3):115-23.
21. Ivanov AA^1^, Khuri FR, Fu H. Targeting protein-protein interactions as an anti cancer strategy. Trends Pharmacol Sci. 2013 Jul;34(7):393-400.
22. Schadt EE. Molecular networks as sensors and drivers of common human diseases. Nature. 2009 Sep 10;461(7261):218-23.
23. Vidal M, Cusick ME, Barabási AL. Interactome networks and human disease. Cell. 2011 Mar 18;144(6):986-98.
24. Barabási AL, Gulbahce N, Loscalzo J. Network medicine: a network based approach to human disease. Nat Rev Genet. 2011 Jan;12(1):56-68.
25. Mark A Rubin and Angelo M De Marzo. Molecular genetics of human prostate cancer. [Mod Pathol.](http://www.ncbi.nlm.nih.gov/pubmed/14752525) 2004 Mar;17(3):380-8.
26. Isaacs W^1^, Kainu T. Oncogenes and tumour suppressor genes in prostate cancer. Epidemiol Rev. 2001;23(1):36-41.
27. Karan D^1^, Lin MF, Johansson SL, Batra SK. Current status of the molecular genetics of human prostatic adenocarcinomas. Int J Cancer. 2003 Jan 20;103(3):285-93.
28. Prevarskaya N^1^, Skryma R, Shuba Y. Ion channels and the hallmarks of cancer. Trends Mol Med. 2010 Mar;16(3):107-21.
29. Bello-DeOcampo D^1^, Tindall DJ. TGF-beta I/Smad signaling in prostate cancer. Curr Drug Targets. 2003 Apr;4(3):197-207.
30. Baron V^1^, Adamson ED, Calogero A, Ragona G. The transcription factor Egr1 is a direct regulators of multiple tumour suppressors including TGFbeta1, PTEN, p53, and fibronectin. Cancer Gene Ther. 2006 Feb;13(2):115-24.
31. Zheng C^1^, Ren Z, Wang H, Zhang W, Kalvakolanu DV, Tian Z, Xiao W. E2F1 induces tumour cell survival via nuclear factor-kappaB-dependent induction of EGR1 dependent transcription in prostate cancer cells. Cancer Res. 2009 Mar 15;69(6):2324-31.
32. Reynolds AR^1^, Kyprianou N. Growth factor signaling in prostatic growth: significance in tumour development and therapeutic targeting. Br J Pharmacol. 2006 Feb;147.
33. Shen MM^1^, Abate-Shen C. Pten inactivation and the emergence of androgen-independent prostate cancer. Cancer Res. 2007 Jul 15;67(14):6535-8.
34. van Duijn PW^1^, Ziel-van der Made AC, van der Korput JA, Trapman J. PTEN mediated G1 cell-cycle arrest in LNCaP prostate cancer cells is associated with altered expressions of cell-cycle regulators. Prostate. 2010 Feb 1;70(2):135-46.
35. Phin S^1^, Moore MW, Cotter PD. Genomic Rearrangements of PTEN in Prostate Cancer. Front Oncol. 2013 Sep 17;3:240.
36. Inoue K^1^, Fry EA, Taneja P. Recent progress in mouse models for tumour suppressor genes and its implications in human cancer. Clin Med Insights Oncol. 2013 Jun 3;7:103-22.
37. Jiang J^1^, Huang H. Targeting the Androgen Receptor by Taxol in Castration-Resistant Prostate Cancer. Mol Cell Pharmacol. 2010 Jan 1;2(1):1-5.
38. Song H^1^, Zhang B, Watson MA, Humphrey PA. Loss of NKx3.1 leads to the activation of discrete downstream target genes during prostate tumorigenesis. Oncogene. 2009 Sep 17;28(37):3307-19.
39. Mathew R, Karantza-Wadsworth V, White E. Role of autophagy in cancer. Nat Rev Cancer. 2007 Dec; 7(12):961-7.
40. Yu L^1^, Tumati V, Tseng SF, Hsu FM, Kim DN, Hong D, Hsieh JT, Jacobs C, Kapur P, Saha D. DAB2IP regulates autophagy in prostate cancer in response to combined treatment of radiation and a DNA-PKcs inhibitor. [Neoplasia.](http://www.ncbi.nlm.nih.gov/pubmed/?term=Neoplasia.+2012+Dec%3B14(12)%3A1203-12.) 2012 Dec;14(12):1203-12.
41. Ziparo E^1^, Petrungaro S, Marini ES, Starace D, Conti S. Autophagy in prostate cancer and androgen suppression therapy. Int J Mol Sci. 2013 Jun 6;14(6):12090-106.
42. Sui X^1^, Chen R, Wang Z, Huang Z, Kong N. Autophagy and chemotherapeutic resistance: a promising therapeutic target for cancer treatment. Cell Death Dis. 2013 Oct 10;4:e838.
43. Chang PC^1^, Wang TY^2^, Chang YT^1^, Chu CY^3^, Lee CL. Autophagy Pathway Is Required for IL-6 Induced Neuroendocrine Differentiation and Chemoresistance of Prostate Cancer LNCaP Cells. PLoS One. 2014 Feb 14;9(2):e88556.
44. Chhipa RR^1^, Wu Y, Ip C. AMPK-mediated autophagy is a survival mechanism in androgen-dependent prostate cancer cells subjected to androgen deprivation and hypoxia. Cell Signal. 2011 Sep;23(9):1466-72.
45. Pennati M^1^, Lopergolo A^1^, Profumo V^1^, De Cesare M^1^, Sbarra S. miR-205 impairs the autophagic flux and enhances cisplatin cytotoxicity in castration-resistant prostate cancer cells. Biochem Pharmacol. 2014 Feb 15;87(4):579-97.
46. Low CG^1^, Luk IS, Lin D, Fazli L, Yang K, Xu Y. BIRC6 protein, an inhibitor of apoptosis: role in survival of human prostate cancer. PLoS One. 2013;8(2):e55837.
47. Qu X^1^, Yu J, Bhagat G, Furuya N, Hibshoosh H, Troxel A. Promotion of tumorigenesis by heterpzygous disruption of the beclin1 autophagy gene. J Clin Invest. 2003 Dec;112(12):1809-20.
48. Djeu JY^1^, Wei S. Clusterin and chemoresistance. Adv Cancer Res. 2009;105:77-92.
49. Zhu B^1^, Block NL, Lokeshwar BL. Interaction between stromal cells and tumour cells induces chemoresistance and matrix metalloproteinase secretion. Ann N Y Acad Sci. 1999 Jun 30;878:642-6.
50. Shiota M^1^, Kashiwagi E, Yokomizo A, Takeuchi A, Dejima T. Interaction between docitaxel resistance and castration resistance in prostate cancer: implications of Twist1, YB-1, and androgen receptor . Prostate. 2013 Sep;73(12):1336-44.
51. O'Neill AJ^1^, Prencipe M, Dowling C, Fan Y, Mulrane L. Characeterisation and manipulation of docetaxel resistant prostate cancer cell lines. Mol Cancer. 2011 Oct 7;10:126.
52. Tantivejkul K^1^, Loberg RD, Mawocha SC, Day LL, John LS, Pienta BA, Rubin MA, Pienta KJ. PAR1-mediated NF-kappaB activation promotes survival of prostate cancer cells through a Bcl-xL-dependent mechanism. [J Cell Biochem.](http://www.ncbi.nlm.nih.gov/pubmed/?term=PAR1-Mediated+NFkB+Activation+Promotes+Survival+of+Prostate+Cancer+Cells+Through+a+Bcl-xL-Dependent+Mechanism) 2005 Oct 15;96(3):641-52.
53. Codony-Servat J^1^, Marín-Aguilera M, Visa L, García-Albéniz X. Nuclear factor-kappa B and interleukin-6 related docetaxel resistance in castration-resistant prostate cancer. Prostate. 2013 Apr;73(5):512-21.
54. Zheng C^1^, Ren Z, Wang H, Zhang W, Kalvakolanu DV, Tian Z, Xiao W. E2F1 induces tumour cell survival via nuclear factor-kappaB-dependent induction of EGR1 dependent transcription in prostate cancer cells. Cancer Res. 2009 Mar 15;69(6):2324-31.
55. Ni J^1^, Cozzi P, Hao J, Beretov J, Chang L, Duan W. Epithelial cell adhesion molecule (EpCAM) is associated with prostate cancer metastasis and chemo/radioresistance via the PI3K/Akt/mTOR signaling pathway. Int J Biochem Cell Biol. 2013 Dec;45(12):2736-48.
56. Lee JT Jr^1^, Steelman LS, McCubrey JA. Phosphatidykinositol 3’-kinase activation leads to multidrug resistance protein-1 expression and subsequent chemoresistance in advanced prostate cancer cells. Cancer Res. 2004 Nov 15;64(22):8397-404.
57. Zhong B^1^, Sallman DA, Gilvary DL, Pernazza D, Sahakian. Induction of clusterin by AKT-role in cytoprotection against docetaxel in prostate tumour cells. Mol Cancer Ther. 2010 Jun;9(6):1831-41.
58. Djeu JY^1^, Wei S. Clusterin and chemoresistance. Adv Cancer Res. 2009;105:77-92.
59. Patterson SG^1^, Wei S, Chen X, Sallman DA, Gilvary DL. Novel role of Stat1 in the development of docetaxel resistance in prostate tumour cells. Oncogene. 2006 Oct 5;25(45):6113-22.
60. Wu K^1^, Xie D, Zou Y, Zhang T, Pong RC, Xiao G. The mechanism of DAB2IP in chemoresistance of prostate cancer cells. Clin Cancer Res. 2013 Sep 1;19(17):4740-9.
61. Jiang J^1^, Huang H. Targeting the Androgen Receptor by Taxol in Castration-Resistant Prostate Cancer. Mol Cell Pharmacol. 2010 Jan 1;2(1):1-5.
62. Shiota M^1^, Kashiwagi E, Yokomizo A, Takeuchi A. Interaction between docetaxel resistance and castration resistance in prostate cancer: implications of Twist-1, YB-1 and androgen receptor. Prostate. 2013 Sep;73(12):1336-44.
63. Shiota M^1^, Takeuchi A, Song Y, Yokomizo A, Kashiwagi E. Y-box binding protein-1 promotes castration-resistant prostate cancer growth via androgen receptor expression. Endocr Relat Cancer. 2011 Jul 11;18(4):505-17.
64. Shiota M^1^, Zoubeidi A, Kumano M, Beraldi E, Naito S, Nelson CC. Clusterin is a critical downstream mediator stress-induced YB-1 transactivation in prostate cancer. Mol Cancer Res. 2011 Dec;9(12):1755-66.
65. Shiota M^1^, Izumi H, Tanimoto A, Takahashi M, Miyamoto N. Programmed cell death protein 4 down regulates Y-box binding protein-1 expression via a direct interaction with Twist1 to suppress cancer cell growth. Cancer Res. 2009 Apr 1;69(7):3148-56.
66. Shi GH^1^, Ye DW, Yao XD, Zhang SL, Dai B. Involvement of microRNA-21 in mediating chemo-resistance to docetaxel in androgen-independent prostate cancer PC-3 cells. Acta Pharmacol Sin. 2010 Jul;31(7):867-73.
67. Terry S^1^, Maillé P, Baaddi H, Kheuang L, Soyeux P. Cross modulation between the androgen receptor axis and protocadherin-PC in mediating neuroendocrine transdifferentiation and therapeutic resistance to prostate cancer. Neoplasia. 2013 Jul;15(7):761-72.
68. Li Y^1^, Chen HQ, Chen MF, Liu HZ, Dai YQ. Neuroendocrine differentiation is involved in chemoresistance induced by EGF in prostate cancer cells. Life Sci. 2009 Jun 19;84(25-26):882-7.
69. Chang PC^1^, Wang TY^2^, Chang YT^1^, Chu CY^3^, Lee CL. Autophagy Pathway Is Required for IL-6 Induced Neuroendocrine Differentiation and Chemoresistance of Prostate Cancer. LNCaP Cells. PLoS One. 2014 Feb 14;9(2):e88556.
70. Sui X^1^, Chen R, Wang Z, Huang Z, Kong N. Autophagy and chemotherapeutic resistance: a promising therapeutic target for cancer treatment. Cell Death Dis. 2013 Oct 10;4:e838.
71. Tu H^1^, Jacobs SC, Borkowski A, Kyprianou N. Incidence of apoptosis and cell proliferation in prostate cancer: relationship with TGF-beta1 and bcl-2 expression. Int J Cancer. 1996 Oct 21;69(5):357-63.
72. Lorenzo PI^1^, Arnoldussen YJ, Saatcioglu F. Molecular mechanisms of apoptosis in prostate cancer. Crit Rev Oncog. 2007 Aug;13(1):1-38.
73. Denmeade SR^1^, Lin XS, Isaacs JT. Role of programmed (apoptotic) cell death during the progression and therapy for prostate cancer. Prostate. 1996 Apr;28(4):251-65.
74. Guseva NV^1^, Taghiyev AF, Rokhlin OW, Cohen MB. Death receptor-induced cell death in prostate cancer. J Cell Biochem. 2004 Jan 1;91(1):70-99.
75. McKenzie S^1^, Kyprianou N. Apoptosis evasion: the role of survival pathways in prostate cancer progression and therapeutic resistance. J Cell Biochem. 2006 Jan 1;97(1):18-32.
76. Gleave M^1^, Miyake H, Chi K. Beyond simple castration: targeting the molecular basis of treatment resistance in advanced prostate cancer. Cancer Chemother Pharmacol. 2005 Nov;56 Suppl 1:47-57.
77. Wang D^1^, Lu J, Tindall DJ. Androgens regulate TRAIL-induced cell death in prostate cancer cells via multiple mechanisms. Cancer Lett. 2013 Jul 10;335(1):136-44.
78. Xu J^1^, Zhou JY, Wei WZ, Wu GS. Activation of Akt survival pathway contributes to TRAIL resistance in cancer cells. PLoS One. 2010 Apr 19;5(4):e10226.
79. Tong X^1^, Li H. eNOS protects prostate cancer cells from TRAIL-induced apoptosis. Cancer Lett. 2004 Jul 8;210(1):63-71.
80. McCourt C^1^, Maxwell P, Mazzucchelli R, Montironi R. Elevation of c-FLIP in castrate-resistant prostate cancer antagonizes therapeutic response to androgen receptor-targeted therapy. Clin Cancer Res. 2012 Jul 15;18(14):3822-33.
81. Kim MH^1^, Minton AZ, Agrawal V. C/EBP beta regulates metastatic gene expression and confers TNF-alpha resistance to prostate cancer cells. Prostate. 2009 Sep 15;69(13):1435-47.
82. Benavides F^1^, Blando J, Perez CJ, Garg R, Conti CJ, DiGiovanni J, Kazanietz MG. Transgenic overexpression of PKCε PKCε in the mouse prostate induces pre-neoplastic lesions. Cell Cycle. 2011 Jan 15;10(2):268-77.
83. Ricote M^1^, García-Tuñón I, Fraile B, Fernández C, Aller P, Paniagua R, Royuela M. P38 MAPK protects against TNF-alpha-provoked apoptosis in LNCaP prostatic cancer cells. Apoptosis. 2006 Nov;11(11):1969-75.
84. Wang D^1^, Montgomery RB, Schmidt LJ, Mostaghel EA, Huang H, Nelson PS, Tindall DJ. Reduced tumour necrosis factor receptor-associated death domain expression is associated with prostate cancer progression. Cancer Res. 2009 Dec 15;69(24):9448-56.
85. Zhu LB^1^, Zhao ST^2^, Xu TZ^3^, Wang H^4^. Tumour necrosis factor- α-induced a disintegrin and metalloprotease 10 increases apoptosis resistance in prostate cancer cells. [Oncol Lett.](http://www.ncbi.nlm.nih.gov/pubmed/?term=Tumor+necrosis+factor-α-induced+a+disintegrin+and+metalloprotease+10+increases+apoptosis+resistance+in+prostate+cancer+cells) 2014 Mar;7(3):897-901.
86. Winter RN^1^, Kramer A, Borkowski A, Kyprianou N. Loss of caspase-1 and caspase-3 in human prostate cancer. [Cancer Res.](http://www.ncbi.nlm.nih.gov/pubmed/11221855) 2001 Feb 1;61(3):1227-32.
87. Jane-Dar Lee, Lieng-Yi Lu, Wen-Hsuan Cheng, Ya-Tang Yang, Shaw-Yeu Jeng. Dysregulation of apoptosis involves the extrinsic pathway in human prostate cancer. JTUA 20:120-6, 2009.
88. Rodríguez-Berriguete G^1^, Galvis L, Fraile B, de Bethencourt FR, Martínez-Onsurbe P, Olmedilla G, Paniagua R, Royuela M. Immunoreactivity to caspase-3, caspase-7, caspase-8 and caspase-9 form is frequently lost in human prostate tumours.
89. Bruckheimer EM^1^, Kyprianou N. Bcl-2 antagonizes the combined apoptotic effect of transforming growth factor-beta and dihydrotestosterone in prostate cancer cells. Prostate. 2002 Oct 1;53(2):133-42.
90. Verdoodt B^1^, Neid M, Vogt M, Kuhn V, Liffers ST, Palisaar RJ, Noldus J, Tannapfel A, Mirmohammadsadegh A. MicroRNA-205, a novel regulator of the anti-apoptotic protein Bcl2, is downregulated in prostate cancer. Int J Oncol. 2013 Jul;43(1):307-14.
91. Browne G^1^, Nesbitt H, Ming L, Stein GS. Bicalutamide-induced hypoxia potentiates RUNX2-mediated Bcl-2 expression resulting in apoptosis resistance. Br J Cancer. 2012 Nov 6;107(10):1714-21.
92. Castilla C^1^, Congregado B, Chinchón D, Torrubia FJ. BCL-xL is overexpressed in hormone-resistant prostate cancer and promotes survival of LNCaP cells via interaction with proapoptotic Bak. Endocrinology. 2006 Oct;147(10):4960-7.
93. Sun A^1^, Tang J, Hong Y, Song J, Terranova PF, Thrasher JB. Androgen receptor-dependent regulation of BCL-xL expression: Implication in prostate cancer progression. Prostate. 2008 Mar 1;68(4):453-61.
94. McJilton MA^1^, Van Sikes C, Wescott GG, Wu D, Foreman TL, Gregory CW, Weidner DA, Harris Ford O, Morgan Lasater A, Mohler JL, Terrian DM. Protein kinaseCepsilon interacts with Bax and promotes survival of human prostate cancer cells. Oncogene. 2003 Sep 11;22(39):7958-68.
95. Benavides F^1^, Blando J, Perez CJ, Garg R, Conti CJ, DiGiovanni J. Transgenic overexpression of PKCε in the mouse prostate induces preneoplastic lesions. Cell Cycle. 2011 Jan 15;10(2):268-77.
96. Yang Y^1^, Hou H, Haller EM, Nicosia SV. Suppression of FOXO1 activity by FHL2 through SIRT1-mediated deacetylation. EMBO J. 2005 Mar 9;24(5):1021-32.
97. Schmukler E^1^, Shai B, Ehrlich M, Pinkas-Kramarski R. Neuregulin promotes incomplete autophagy of prostate cancer cells that is independent of mTOR pathway inhibition. PLoS One. 2012;7(5):e36828.
98. Bennett HL^1^, Fleming JT, O'Prey J, Ryan KM, Leung HY. Androgens modulates autophagy and cell death via regulation of the endoplasmic reticulum chaperone glucose-regulated protein 78/BiP in prostate cancer cells. Cell Death Dis. 2010 Sep 9;1:e72.
99. Jin RJ^1^, Lho Y, Connelly L, Wang Y, Yu X, Saint Jean L, Case TC. The nuclear factor-kappaB controls the progression of prostate cancer to androgen independent growth. Cancer Res. 2008 Aug 15;68(16):6762-9.
100. Tantivejkul K^1^, Loberg RD, Mawocha SC, Day LL. PAR1-mediated NFkappaB activation promotes survival of prostate cancer cells through a BCL-xL-dependent mechanism. J Cell Biochem. 2005 Oct 15;96(3):641-52.
101. Lamb LE^1^, Zarif JC, Miranti CK. The androgen receptor induces integrin α6β1 to promote prostate cancer cell survival via NF-κB and Bcl-xL Independently of PI3K signaling. Cancer Res. 2011 Apr 1;71(7):2739-49.
102. Zoubeidi A^1^, Ettinger S, Beraldi E, Hadaschik B, Zardan A. Clusterin facilitates COMMD1 and I-kappa B degradation to enhance NF-kappa B activity in prostate cancer cells. Mol Cancer Res. 2010 Jan;8(1):119-30.
103. Shanmugam MK^1^, Rajendran P, Li F, Nema T, Vali S, Abbasi T, Kapoor S, Sharma A, Kumar AP, Ho PC, Hui KM, Sethi G. Ursolic acid inhibits multiple cell survival pathways leading to suppression of growth of prostate cancer xenograft in nude mice. J Mol Med (Berl). 2011 Jul;89(7):713-27.
104. Murata T^1^, Takayama K, Urano T, Fujimura T. 14-3-3ζ, a novel androgen-responsive gene, is up regulated in prostate cancer and promotes prostate cancer cell proliferation and survival. Clin Cancer Res. 2012 Oct 15;18(20):5617-27.
105. Chesire DR^1^, Ewing CM, Gage WR, Isaacs WB. In vitro evidence for complex modes of nuclear beta-catenine signaling during prostate cancer growth and tumorigenesis. Oncogene. 2002 Apr 18;21(17):2679-94.
106. Sun P^1^, Xiong H, Kim TH, Ren B, Zhang Z. Positive inter-regulation between beta-catenine/T cell factor-4 signaling and endothelin-1 signaling potentiates proliferation and survival of prostate cancer cells. Mol Pharmacol. 2006 Feb;69(2):520-31.
107. Peacock SO^1^, Fahrenholtz CD, Burnstein KL. Vav3 enhances androgen receptor splice variants activity and is critical for castration-resistant prostate cancer growth and survival. Mol Endocrinol. 2012 Dec;26(12):1967-79.
108. Jariwala U^1^, Prescott J, Jia L, Barski A, Pregizer S. Identification of novel androgen receptor target genes in prostate cancer. Mol Cancer. 2007 Jun 6;6:39.
109. Mo W^1^, Zhang J, Li X, Meng D, Gao Y, Yang S. Identification of novel AR-targeted microRNAs mediating androgen signaling through critical pathways to regulate cell viability in prostate cancer. PLoS One. 2013;8(2):e56592.
110. Gao L^1^, Schwartzman J, Gibbs A, Lisac R. Androgen receptor promotes ligand-independent prostate cancer progression through c-Myc upregulation. PLoS One. 2013 May 21;8(5):e63563.
111. Watson DG^1^, Tonelli F, Alossaimi M, Williamson L, Chan E. The role of spigosine kinases 1 and 2 in regulating the Warburg effect in prostate cancer cells. Cell Signal. 2013 Apr;25(4):1011-7.
112. Bitting RL^1^, Armstrong AJ. Targeting the PI3K/Akt/mTOR pathway in castration-resistant prostate cancer. Endocr Relat Cancer. 2013 May 20;20(3):R83-99.
113. Shukla S^1^, Maclennan GT, Hartman DJ, Fu P, Resnick MI, Gupta S. Activation of PI3K-Akt pathway promotes prostate cancer cell invasion. Int J Cancer. 2007 Oct 1;121(7):1424-32.
114. Zins K^1^, Lucas T, Reichl P, Abraham D, Aharinejad S. A Rac1/Cdc42 GTPase-specific specific small molecule inhibitors suppresses primary human prostate cancer xenografts and prolongs survival in mice. PLoS One. 2013 Sep 11;8(9):e74924.
115. Carlton R Cooper, Christopher H Chay, and Kenneth J Pienta. The role of α_v_β_3_ in prostate cancer progression. Neoplasia. May 2002; 4(3): 191–194.
116. Majumder PK^1^, Sellers WR. Akt-regulated pathways in prostate cancer. [Oncogene.](http://www.ncbi.nlm.nih.gov/pubmed/?term=7.%09Akt-regulated+pathways+in+prostate+cancer) 2005 Nov 14;24(50):7465-74.
117. Zhuang L^1^, Kim J, Adam RM, Solomon KR, Freeman MR. Cholesterol targeting alters alters lipid raft composition and cell survival in prostate cancer cell and xenograft. J Clin Invest. 2005 Apr;115(4):959-68.
118. Wu D^1^, Thakore CU, Wescott GG, McCubrey JA, Terrian DM. Integrin signaling links protein kinase C epsilon to the protein kinaseB/Akt survival pathway in recurrent prostate cancer cells. [Oncogene.](http://www.ncbi.nlm.nih.gov/pubmed/?term=Integrin+signaling+links+protein+kinase+Calt+epsilon+to+the+protein+kinase+B%2FAkt+survival+pathway+in+recurrent+prostate+cancer+cells) 2004 Nov 11;23(53):8659-72.
119. Benavides F^1^, Blando J, Perez CJ, Garg R, Conti CJ. Transgenic overexpression of PKCε in the mouse prostate induces preneoplastic lesions. Cell Cycle. 2011 Jan 15;10(2):268-77.
120. Lu S^1^, Tsai SY, Tsai MJ. Regulation of androgen-dependent prostatic cancer cell growth: androgen regulation of CDK2, CDK4 and CKI p16 genes. Cancer Res. 1997 Oct 15;57(20):4511-6.
121. Jariwala U^1^, Prescott J, Jia L, Barski A, Pregizer S, Cogan JP, Arasheben A, Tilley WD, Scher HI, Gerald WL, Buchanan G, Coetzee GA, Frenkel B. Identification of novel androgen receptor target genes in prostate cancer. Mol Cancer. 2007 Jun 6;6:39.
122. Hsu FN^1^, Chen MC, Chiang MC, Lin E, Lee YT, Huang PH, Lee GS, Lin H. Regulation of androgen receptor and prostate cancer growth by cyclin dependent kinase 5. J Biol Chem. 2011 Sep 23;286(38):33141-9.
123. DaSilva J^1^, Gioeli D, Weber MJ, Parsons SJ. The neuroendocrine-derived peptide parathyroid hormone related protein promotes prostate cancer cell growth by stabilizing the androgen receptor. Cancer Res. 2009 Sep 15;69(18):7402-11.
124. Coffey K^1^, Robson CN. Regulation of androgen receptor by post- translational modifications. J Endocrinol. 2012 Nov;215(2):221-37.
125. Kahl P^1^, Gullotti L, Heukamp LC, Wolf S, Friedrichs N, Vorreuther R, Solleder G, Bastian PJ, Ellinger J, Metzger E, Schüle R, Buettner R. Androgen receptor coactivators lysine-specific demethylase 1 and four and a half LIM domain protein 2 predict risk of prostate cancer recurrence. Cancer Res. 2006 Dec 1;66(23):11341-7.
126. Schmidt LJ^1^, Duncan K, Yadav N, Regan KM, Verone AR, Lohse CM, Pop EA, Attwood K, Wilding G, Mohler JL, Sebo TJ, Tindall DJ, Heemers HV. Rho A as a mediator of clinically relevant androgen actions in prostate cancer. Mol Endocrinol. 2012 May;26(5):716-35.
127. Patki M^1^, Chari V, Sivakumaran S, Gonit M, Trumbly R, Ratnam M. The ETS domain transcription factor ELK1 directs a critical component of growth signaling. J Biol Chem. 2013 Apr 19;288(16):11047-65.
128. Gamble SC^1^, Chotai D, Odontiadis M, Dart DA, Brooke GN, Powell SM, Reebye V, Varela-Carver A, Kawano Y, Waxman J, Bevan CL. Prohibitin, a protein down-regulated by androgens, repress androgen receptor activity. [Oncogene.](http://www.ncbi.nlm.nih.gov/pubmed/?term=Oncogene.+2007+Mar+15%3B26(12)%3A1757-68.) 2007 Mar 15;26(12):1757-68.
129. Veeramani S^1^, Chou YW, Lin FC, Muniyan S, Lin FF, Kumar S, Xie Y, Lele SM, Tu Y, Lin MF. Reactive oxygen species induced by p66Shc longevity protein mediate nongenomic androgen action via tyrosine phosphorylation signaling to enhance tumorigenicity of prostate cancer cells. [Free Radic Biol Med.](http://www.ncbi.nlm.nih.gov/pubmed/?term=Free+Radic+Biol+Med.+2012+Jul+1%3B53(1)%3A95-108.) 2012 Jul 1;53(1):95-108.
130. Liu X^1^, Busby J, John C, Wei J, Yuan X, Lu ML. Direct interaction between AR and PAK6 activation. [PLoS One.](http://www.ncbi.nlm.nih.gov/pubmed/?term=PLoS+One.+2013+Oct+10%3B8(10)%3Ae77367.) 2013 Oct 10;8(10):e77367.
131. Massie CE^1^, Lynch A, Ramos-Montoya A, Boren J. The androgen receptor fuels prostate cancer by regulating central metabolism and biosynthesis. EMBO J. 2011 May 20;30(13):2719-33.
132. Tennakoon JB^1^, Shi Y, Han JJ, Tsouko E, White MA, Burns AR, Zhang A, Xia X, Ilkayeva OR, Xin L, Ittmann MM, Rick FG, Schally AV, Frigo DE. Androgen regulate prostate cancer cell growth via an AMPK-PGC-1α-mediated metabolic switch. Oncogene. 2013 Nov 4.
133. Sung SY^1^, Hsieh CL, Wu D, Chung LW, Johnstone PA. Tumour microenvironment promotes cancer progression, metastasis and therapeutic resistance. Curr Probl Cancer. 2007 Mar-Apr;31(2):36-100.
134. Barron DA^1^, Rowley DR. The reactive stroma microenvironment and prostate cancer progression. Endocr Relat Cancer. 2012 Oct 30;19(6):R187-204.
135. Reebye V^1^, Frilling A, Habib NA, Mintz PJ. Intracellular adaptor molecules and AR signaling in the tumour microenvironment. Cell Signal. 2011 Jun;23(6):1017-21.
136. Di Lorenzo G^1^, Tortora G, D'Armiento FP, De Rosa G. Expression of epidermal growth factor correlates with disease relapse and progression to androgen-independence in human prostate cancer. Clin Cancer Res. 2002 Nov;8(11):3438-44.
137. Jeong JY^1^, Hoxhaj G, Socha AL, Sytkowski AJ, Feldman L. An erythropoietin autocrine/paracrine axis modulates the growth and survival of human prostate cancer cells. Mol Cancer Res. 2009 Jul;7(7):1150-7.
138. Karantanos T^1^, Corn PG, Thompson TC. Prostate cancer progression after androgen deprivation therapy: mechanisms of castrate resistance and novel therapeutic approaches. Oncogene. 2013 Dec 5;32(49):5501-11.
139. Russell PJ^1^, Bennett S, Stricker P. Growth factor involvement in progression of prostate cancer. Clin Chem. 1998 Apr;44(4):705-23.
140. Arnold JT^1^, Isaacs JT. Mechanisms involved in the progression of androgen independent prostate cancers: it is not only the cancer cells fault.
141. Nakashiro K^1^, Hara S, Shinohara Y, Oyasu M. Phenotypic switch from paracrine to autocrine role of hepatocyte growth factor in an androgen independent human prostatatic carcinoma cell line. Am J Pathol. 2004 Aug;165(2):533-40.
142. Veeramani S^1^, Yuan TC, Lin FF, Lin MF. Mitochondrial redox signaling by p66Shc is involved in regulating androgenic growth stimulation of human prostate cancer cells. [Oncogene.](http://www.ncbi.nlm.nih.gov/pubmed/?term=Mitochondrial+redox+signaling+by+p66Shc+is+involved+in+regulating+androgenicgrowth+stimulation+of+human+prostate+cancer+cells.) 2008 Aug 28;27(37):5057-68.
143. Ray S^1^, Johnston R, Campbell DC, Nugent S, McDade SS, Waugh D, Panov KI. Androgens and estrogens stimulate ribosome biogenesis in prostate and breast cancer cells in receptor dependent manner. Gene. 2013 Aug 15;526(1):46-53.
144. Yang Y^1^, Jiao L, Hou J, Xu C, Wang L, Yu Y, Li Y, Yang C, Wang X, Sun Y. Dishevelled-2 silencing reduces androgen dependent prostate tumour cell proliferation and migration and expression of Wnt-3a and matrix metalloproteinases. Mol Biol Rep. 2013 Jul;40(7):4241-50.
145. Murata T^1^, Takayama K, Urano T, Fujimura T, Ashikari D, Obinata D, Horie-Inoue K, Takahashi S, Ouchi Y, Homma Y, Inoue S. 14-3-3ζ, a novel androgen responsive gene, is upregulated in prostate cancer and promotes prostate cancer cell proliferation and survival. Clin Cancer Res. 2012 Oct 15;18(20):5617-27.
146. Palamakumbura AH^1^, Vora SR, Nugent MA, Kirsch KH, Sonenshein GE, Trackman PC. Lysyl oxydase propeptide inhibits prostate cancer cell growth by mechanisms that target FGF-2 cell binding and signaling. Oncogene. 2009 Sep 24;28(38):3390-400.
147. Dayyani F^1^, Parikh NU, Varkaris AS, Song JH, Moorthy S, Chatterji T, Maity SN, Wolfe AR, Carboni JM, [Gottardis MM](http://www.ncbi.nlm.nih.gov/pubmed?term=Gottardis MM%5BAuthor%5D&cauthor=true&cauthor_uid=23300537),Logothetis CJ, Gallick GE. Combined inhibition of IGF-1R/IR and Src family kinases enhances antitumour effects in prostate cancer by decreasing activated survival pathways. PLoS One. 2012;7(12):e51189.
148. Tao J^1^, Wu D, Xu B, Qian W, Li P, Lu Q, Yin C, Zhang W. microRNA-133 inhibits cell proliferation, migration and invasion in prostate cancer cells by targeting the epidermal growth factor receptor. Oncol Rep. 2012 Jun;27(6):1967-75.
149. Sen A^1^, De Castro I, Defranco DB, Deng FM, Melamed J, Kapur P, Raj GV, Rossi R, Hammes SR. Paxillin mediates extranuclear and intranuclear signaling in prostate cancer proliferation. J Clin Invest. 2012 Jul 2;122(7):2469-81.
150. Syed V^1^, Mak P, Du C, Balaji KC. Beta-catenine mediates alteration in cell proliferation, motility and invasion of prostate cancer cells by differential expression of E-cadherin and protein kinase D1. [J Cell Biochem.](http://www.ncbi.nlm.nih.gov/pubmed/?term=Betacatenin+mediates+alteration+in+cell+proliferation%2C+motility+and+invasion+ofprostate+cancer+cells+by+differential+expression+of+E-cadherin+and+protein+kinaseD1.) 2008 May 1;104(1):82-95.
151. Liu Z^1^, Rebowe RE, Wang Z, Li Y, Wang Z, Depaolo JS, Guo J, Qian C, Liu W. KIF3a Promotes Proliferation and Invasion via Wnt Signaling in Advanced Prostate Cancer. Mol Cancer Res. 2014 Jan 10.
152. Mol AJ, Geldof AA, Meijer GA, van der Poel HG, van Moorselaar RJ. New experimental markers for early detection of high risk prostate cancer: role of cell-cell adhesion and cell migration. J Cancer Res Clin Oncol. 2007 Oct;133(10):687-95.
153. Cohen MB, Griebling TL, Ahaghotu CA, Rokhlin OW, Ross JS. Cellular adhesion molecules in urologic malignancy. Am J Clin Pathol. 1997 Jan;107(1):56-63.
154. Mol AJ, Geldof AA, Meijer GA, van der Poel HG, van Moorselaar RJ. New experimental markers for early detection of high risk prostate cancer: role of cell-cell adhesion and cell migration. J Cancer Res Clin Oncol. 2007 Oct;133(10):687-95.
155. Trerotola M, Li J, Alberti S, Languino LR. Trop-2 inhibits prostate cancer cell adhesion to fibronectin through the β1 integrin-RACK1 axis. J Cell Physiol. 2012 Nov;227(11):3670-7.
156. Van Slambrouck S, Jenkins AR, Romero AE, Steelant WF. Reorganizations of the integrin alpha2 subunit controls cell adhesion and cancer cell invasion in prostate cancer. [Int J Oncol.](http://www.ncbi.nlm.nih.gov/pubmed/?term=Int+J+Oncol.+2009+Jun%3B34(6)%3A1717-26.) 2009 Jun;34(6):1717-26.
157. Iwasaki M, Homma S, Hishiya A, Dolezal SJ, Reed JC, Takayama S. BAG3 rgulates motility and adhesion of epithelial cancer cells. Cancer Res. 2007 Nov 1;67(21):10252-9.
158. Johnson TR, Khandrika L, Kumar B, Venezia S, Koul S, Chandhoke R, Maroni P, Donohue R, Meacham RB, Koul HK. Focal adhesion kinase controls aggressive phenotype of androgen independent prostate cancer. Mol Cancer Res. 2008 Oct;6(10):1639-48.
159. Bokobza SM, Ye L, Kynaston HG, Jiang WG. Growth and differentiation factor-9 promotes adhesive and motile capacity of prostate cancer cells by up-regulating FAK and paxillin via Smad dependent pathway. Oncol Rep. 2010 Dec;24(6):1653-9.
160. Zheng Y, Tyner AL. Context-specific protein tyrosine kinase 6(PTK6) signaling in prostate cancer. [Eur J Clin Invest.](http://www.ncbi.nlm.nih.gov/pubmed/23398121) 2013 Apr;43(4):397-404.
161. Zheng Y, Asara JM, Tyner AL. Protein-tyrosine kinase 6 promotes peripheral adhesion complex formation and cell migration by phosphorylating p130-CRK associated substrate. J Biol Chem. 2012 Jan 2;287(1):148-58.
162. Kaulfuss S, Grzmil M, Hemmerlein B, Thelen P, Schweyer S, Neesen J, Bubendorf L, Glass AG, Jarry H, Auber B, Burfeind P. Leupaxin, a novel coactivator of the androgen receptor, is expressed in prostate cancer and plays a role in adhesion and invasion of prostate carcinoma cells. [Mol Endocrinol.](http://www.ncbi.nlm.nih.gov/pubmed/?term=Mol+Endocrinol.+2008+Jul%3B22(7)%3A1606-21.) 2008 Jul;22(7):1606-21.
163. Kaulfuss S, von Hardenberg S, Schweyer S, Herr AM, Laccone F, Wolf S, Burfeind P. Leupaxin acts as a mediator in prostate carcinoma progression through deregulation of p120catenine expression. Oncogene. 2009 Nov 12;28(45):3971-82.
164. Zhu GH, Dai HP, Shen Q. [Molecular mechanisms of leupaxin involved in prostate carcinoma metastasis].[Article in chinese]. Zhonghua Nan Ke Xue. 2013 Jun;19(6):555-8.
165. Kaulfuss S, Grzmil M, Hemmerlein B, Thelen P, Schweyer S, Neesen J, Bubendorf L, Glass AG, Jarry H, Auber B, Burfeind P. Leupaxin a novel coactivator of androgen receptor, is expressed in prostate cancer and plays a role in adhesion and invasion of prostate carcinoma cells. Mol Endocrinol. 2008 Jul;22(7):1606-21.
166. Hara T, Miyazaki H, Lee A, Tran CP, Reiter RE. Androgen receptor and invasion in prostate cancer. Cancer Res. 2008 Feb 15;68(4):1128-35.
167. Yang Y, Jiao L, Hou J, Xu C, Wang L, Yu Y, Li Y, Yang C, Wang X, Sun Y. Dishevelled-2 silencing reduces androgen dependent prostate tumour proliferation and migration and expression of Wnt-3a and matrix metalloproteinases. Mol Biol Rep. 2013 Jul;40(7):4241-50.
168. Trudel D, Fradet Y, Meyer F, Harel F, Tetu B. Significance of MMP-2 expression in prostate cancer: an immunohistochemical study. *Cancer Res* 2003; 63: 8511–5.
169. Matsumura Y, Shimada K, Tanaka N, Fujimoto K, Hirao Y, Konishi N. Phosphorylation status of Fas associated death domain-containing protein regulates telomerase activity and strongly correlates with prostate cancer outcomes. [Pathobiology.](http://www.ncbi.nlm.nih.gov/pubmed/19955841) 2009;76(6):293-302.
170. Ducrest AL, Szutorisz H, Lingner J, Nabholz M. Regulation of the human telomerase reverse transcriptase gene. Oncogene 2002; 21: 541–552.
171. Dai Y, Qi L, Zhang X, Li Y, Chen M, Zu X. CrkI and p130(Cas) regulates the migration and invasion of prostate cancer cells. Cell Biochem Funct. 2011 Dec;29(8):625-9.
172. Sarker D, Reid AH, Yap TA, de Bono JS. Targeting the PI3K/AKT pathway for the treatment of prostate cancer. Clin Cancer Res. 2009 Aug 1;15(15):4799-805.
173. Bitting RL, Armstrong AJ. Targeting the PI3K/Akt/mTOR pathway in castration resistant prostate cancer. Endocr Relat Cancer. 2013 May 20;20(3):R83-99.
174. Dasgupta S, Srinidhi S, Vishwanatha JK. Oncogenic activation in prostate cancer progression and metastasis: Molecular insights and future challenges. [J Carcinog.](http://www.ncbi.nlm.nih.gov/pubmed/?term=Oncogenic+activation+in+prostate+cancer+progression+and+metastasis%3A+Molecular+insights+and+future+challenges) 2012;11:4.
175. Wu SR, Cheng TS, Chen WC, Shyu HY, Ko CJ, Huang HP, Teng CH, Lin CH, Johnson MD, Lin CY, Lee MS. Matriptase is involved in ErbB-2-induced prostate cancer cell invasion. Am J Pathol. 2010 Dec;177(6):3145-58.
176. Walker L, Millena AC, Strong N, Khan SA. Expression of TGFβ3 and its effects on migratory and invasive behavior of prostate cancer cells: involvement of PI3-kinase/AKT signaling pathway. [Clin Exp Metastasis.](http://www.ncbi.nlm.nih.gov/pubmed/?term=Clin+Exp+Metastasis.+2013+Jan%3B30(1)%3A13-23.) 2013 Jan;30(1):13-23.
177. El-Haibi CP, Singh R, Sharma PK, Singh S, Lillard JW Jr. CXCL13 mediates prostate cancer cell proliferation through JNK signaling and invasion through ERK activation. Cell Prolif. 2011 Aug;44(4):311-9.
178. Franzen CA, Amargo E, Todorović V, Desai BV, Huda S, Mirzoeva S, Chiu K, Grzybowski BA, Chew TL, Green KJ, Pelling JC. The chemopreventive bioflavonoid apigenin inhibits prostate cancer cell motility through the focal adhesion kinase/Src signaling mechanism. [Cancer Prev Res (Phila).](http://www.ncbi.nlm.nih.gov/pubmed/?term=Cancer+Prev+Res+(Phila).+2009+Sep%3B2(9)%3A830-41) 2009 Sep;2(9):830-41
179. Vo BT, Morton D Jr, Komaragiri S, [Millena AC](http://www.ncbi.nlm.nih.gov/pubmed?term=Millena AC%5BAuthor%5D&cauthor=true&cauthor_uid=23515290), Leath C, Khan SA. TGF-β effects on prostate cancer cell migration and invasion are mediated by PGE2 through activation of PI3K/AKT/mTOR pathway. Endocrinology. 2013 May;154(5):1768-79.
180. Taylor BS, Schultz N, Hieronymus H, Gopalan A, Xiao Y. Integrative genomic profiling of human prostate cancer. Cancer Cell. 2010 Jul 13;18(1):11-22.
181. Goc A, Al-Husein B, Kochuparambil ST, Liu J, Heston WW, Somanath PR. PI3 kinase integrates Akt and MAP kinase signaling pathways in the regulation of prostate cancer. [Int J Oncol.](http://www.ncbi.nlm.nih.gov/pubmed/21109949) 2011 Jan;38(1):267-77.
182. El-Haibi CP, Singh R, Sharma PK, Singh S, Lillard JW Jr. CXCL13 mediates prostate cancer cell proliferation through JNK signaling and invasion through ERK activation. Cell Prolif. 2011 Aug;44(4):311-9.
183. Ayala G, Thompson T, Yang G, Frolov A, Li R, Scardino P, Ohori M, Wheeler T, Harper W. High levels of phosphorylated form of Akt-1 in prostate cancer and non-neoplastic prostate tissues are strong predictors of biochemical recurrence. Clin Cancer Res. 2004 Oct 1;10(19):6572-8.
184. Van Slambrouck S, Jenkins AR, Romero AE, Steelant WF. Reorganization of the integrin alpha 2 subunit controls cell adhesion and cancer cell invasion in prostate cancer. Int J Oncol. 2009 Jun;34(6):1717-26.
185. Lin KT, Gong J, Li CF, Jang TH, Chen WL, Chen HJ, Wang LH. Vav3-rac1 signaling regulates prostate cancer metastasis with elevated Vav3 expression correlating with prostate cancer progression and posttreatment recurrence. Cancer Res. 2012 Jun 15;72(12):3000-9.
186. Hao F, Tan M, Xu X, Han J, Miller DD, Tigyi G, Cui MZ. Lysophosphatidic acid induces prostate cancer PC3 cell migration via activation of LPA(1), p42 and p38alpha. Biochim Biophys Acta. 2007 Jul;1771(7):883-92.
187. Wang Y, Zhang YX, Kong CZ, Zhang Z, Zhu YY. Loss of P53 facilitates invasion and metastasis of prostate cancer cells. Mol Cell Biochem. 2013 Dec;384(1-2):121-7.
188. Burger KL, Learman BS, Boucherle AK, Sirintrapun SJ, Isom S, Díaz B, Courtneidge SA, Seals DF. Src-dependent Tks5 phosphorylation regulates invadopodia-associated invasions in prostate cancer cells. Prostate. 2014 Feb;74(2):134-48.

# Mohler JL, Partin AW, Coffey DS. Prediction of metastatic potential by a new grading system of cell motility: validation in the Dunning R-3327 prostatic adenocarcinoma model. [**J Urol.**](http://www.ncbi.nlm.nih.gov/pubmed/?term=JUrol+138%3A168–170%2C1987) **1987**Jul;**138**(1):168-70.

1. Mohler JL, Partin AW, Isaacs JT, Coffey DS. Metastatic potential prediction by a visual grading system of cell motility: prospective validation in the Dunning R-3327 prostatic adenocarcinoma model. [Cancer Res.](http://www.ncbi.nlm.nih.gov/pubmed/?term=Metastatic+potential+prediction+by+a+visual+grading+system+of+cell+motility%3A+prospective+validation+in+the+Dunning+R-3327+prostatic+adenocarcinoma+model.) 1988 Aug 1;48(15):4312-7.

# Abdulghani J, Gu L, Dagvadorj A, Lutz J, Leiby B, Bonuccelli G, Lisanti MP, Zellweger T, Alanen K, Mirtti T, Visakorpi T,Bubendorf L, Nevalainen MT. **Stat3 promotes metastatic progression**of**prostate cancer**. [**Am J Pathol.**](http://www.ncbi.nlm.nih.gov/pubmed/18483213)2008 Jun;172(6):1717-28.

# Teng Y, Ghoshal P, Ngoka L, Mei Y, Cowell JK. **Critical role**of the**WASF3 gene**in**JAK2**/**STAT3 regulation**of**cancer cell motility**. [**Carcinogenesis**.](http://www.ncbi.nlm.nih.gov/pubmed/?term=Critical+role+of+the+WASF3+gene+in+JAK2%2FSTAT3+regulation+of+cancer+cell+motility) 2013 Sep;34(9):1994-9.

1. Takenawa T, Suetsugu S. The WASF-WAVE protein network : connecting membrane to the cytoskeleton.

Nat Rev Mol Cell Biol. 2007 Jan;8(1):37-48.

1. Iwasaki M, Homma S, Hishiya A, Dolezal SJ, Reed JC, Takayama S. BAG3 regulates motility and adhesion of epithelial cancer cells. Cancer Res. 2007 Nov 1;67(21):10252-9.
2. Goc A, Abdalla M, Al-Azayzih A, Somanath PR. Rac1 activation driven by 14-3-3ζ dimerization promotes prostate cancer cell-matrix interactions, motility and transendothelial migration. [PLoS One.](http://www.ncbi.nlm.nih.gov/pubmed/?term=Rac1+activation+driven+by+14-3-3ζ+dimerization+promotes+prostate+cancer+cell-matrix+interactions%2C+motility+and+transendothelial+migration.) 2012;7(7).
3. Lin KT, Gong J, Li CF, Jang TH, Chen WL, Chen HJ, Wang LH. Vav3-rac1 signaling regulates prostate cancer metastasis with elevated Vav3 expression correlating with prostate cancer progression and post treatment recurrence. Cancer Res. 2012 Jun 15;72(12):3000-9.
4. Taddei ML, Parri M, Angelucci A, Bianchini F, Marconi C, Giannoni E, Raugei G, Bologna M, Calorini L, Chiarugi P. EphA2 induces metastatic growth regulating amoeboid motility and clonogenic potentials in prostate carcinoma cells. Mol Cancer Res. 2011 Feb;9(2):149-60.
5. Posadas EM, Al-Ahmadie H, Robinson VL, Jagadeeswaran R, Otto K, Kasza KE, Tretiakov M, Siddiqui J, Pienta KJ, Stadler WM, Rinker-Schaeffer C, Salgia R. FYN is overexpressed in human prostate cancer.

[BJU Int.](http://www.ncbi.nlm.nih.gov/pubmed/?term=BJU+Int.+2009+Jan%3B103(2)%3A171-7.) 2009 Jan;103(2):171-7.

1. Ribeiro R, Vasconcelos A, Costa S, Pinto D,Morais A, Oliveira J, Lobo F, Lopes C, andMedeiros R. Overexpressing leptin genetic polymorphism (-2548 G/A) is associated with susceptibility to prostate cancer and risk of advanced disease. Prostate 2004; 59(3): 268-274.
2. Aronson WJ, Barnard RJ, Freedland SJ, Henning S, Elashoff D, Jardack PM, Cohen P, Heber D, and Kobayashi N. Growth Inhibitory Effect of Low Fat Diet on Prostate Cancer Cells: Results of a Prospective, Randomized Dietary Intervention Trial in Men With Prostate Cancer. The Journal of Urology 2010; 183(1): 345-350.
3. Murad A, Down L, Davey Smith G, Donovan J, Lane A, Hamdy F, Neal D, and Martin RM. Associations of asprins, non-steroidal anti-inflammatory drug and paracetomol use with PSA-detected prostate cancer: findings from a large, population-based, case-control study (the ProtecT study). International Journal of

Cancer 2010.

1. Cai Y, Lee YF, Li G, Liu S, Bao BY, Huang J, Hsu CL, and Chang C. A new prostate cancer therapeutic

approach: combination of androgen ablation with COX-2 inhibitor. Int J Cancer 2008; 123(1): 195-201.

1. James N. Cyclooxygenase-2 inhibitors and prostate cancer. Lancet Oncol 2007; 8(10): 859-860.
2. De Marzo AM, Nakai Y, and Nelson WG. Inflammation, atrophy, and prostate carcinogenesis.

Urol Oncol 2007; 25(5): 398-400.

1. Wagenlehner FM, Elkahwaji JE, Algaba F, Bjerklund-Johansen T, Naber KG, Hartung R, and Weidner W. The role of inflammation and infection in the pathogenesis of prostate carcinoma. BJU Int 2007; 100(4): 733-737.
2. Inman BA, Harel F, Audet JF, Meyer F, Douville P, Fradet Y, and Lacombe L. Insulin-like growth factor binding protein 2: an androgendependent predictor of prostate cancer survival. Eur Urol 2005; 47(5): 695-702.
3. Moore MG, Wetterau LA, Francis MJ, Peehl DM, and Cohen P. Novel stimulatory role for insulin-like growth factor binding protein-2 in prostate cancer cells. Int J Cancer 2003; 105 (1): 14-19.
4. van der Poel HG. Molecular markers in the diagnosis of prostate cancer. Crit Rev Oncol Hematol 2007; 61(2): 104-139.
5. Kojima S, Inahara M, Suzuki H, Ichikawa T, and Furuya Y. Implications of insulin-like growth factor-I for prostate cancer therapies. Int J Urol 2009; 16(2): 161-167.
6. Baradaran N, Ahmadi H, Salem S, Lotfi M, Jahani Y, Baradaran N, Mehrsai AR, and Pourmand G. The protective effect of diabetes mellitus against prostate cancer: Role of sex hormones. Prostate 2009; 69(16): 1744-1750.
7. Michalaki V, Syrigos K, Charles P, and Waxman J. Serum levels of IL-6 and TNF-alpha correlate with clinicopathological features and patient survival in patients with prostate cancer.

Br J Cancer 2004; 90(12): 2312-2316.

1. Ricote M, Garcia-Tunon I, Bethencourt FR, Fraile B, Paniagua R, and Royuela M. Interleukin- 1 (IL-1alpha and IL-1beta) and its receptors (IL-1RI, IL-1RII, and IL-1Ra) in prostate carcinoma. Cancer 2004; 100(7): 1388-1396.
2. Tokuda Y, Satoh Y, Fujiyama C, Toda S, Sugihara H, and Masaki Z. Prostate cancer cell growth is modulated by adipocyte-cancer cell interaction. BJU Int 2003; 91(7): 716-720.
3. Wegiel B, Bjartell A, Culig Z, and Persson JL. Interleukin-6 activates PI3K/Akt pathway and regulates cyclin A1 to promote prostate cancer cell survival. Int J Cancer 2008; 122(7): 1521-1529.
4. Mistry T, Digby JE, Desai KM, and Randeva HS. Leptin and adiponectin interact in the regulation of prostate cancer cell growth via modulation of p53 and bcl-2 expression. BJU Int 2008; 101(10): 1317-1322.
5. Chuu CP, Kokontis JM, Hiipakka RA and Liao S. Modulation of liver X receptor signaling as novel therapy for prostate cancer. J Biomed Sci 2007; 14: 543-53.
6. Aaltoma SH, Lipponen PK and Kosma VM. Inducible nitric oxide synthase (iNOS) expression and its prognostic value in prostate cancer. Anticancer Res 2001; 21: 3101-6.
7. Kuroda K, Nakashima J, Kanao K, Kikuchi E, Miyajima A, Horiguchi Y, Nakagawa K, Oya M,Ohigashi T and Murai M. Interleukin 6 is associated with cachexia in patients with prostate cancer. Urology 2007; 69: 113-7.
8. Steiner H, Godoy-Tundidor S, Rogatsch H, Berger AP, Fuchs D, Comuzzi B, Bartsch G, Hobisch A and Culig Z. Accelerated in vivo growth of prostate tumors that up-regulate interleukin-6 is associated with reduced retinoblastoma protein expression and activation of the mitogenactivated protein kinase pathway. Am J Pathol 2003; 162: 655-63.
9. Baltaci S, Orhan D, Gogus C, Turkolmez K, Tulunay O and Gogus O. Inducible nitric oxide synthase expression in benign prostatic hyperplasia, low- and high-grade prostatic intraepithelial neoplasia and prostatic carcinoma. BJU In 2001; 88: 100-3.
10. Dufour J, Viennois E, De Boussac H, Baron S and Lobaccaro JM. Oxysterol receptors, AKT and prostate cancer. Curr Opin Pharmacol 2012; 12: 724-8.
11. Segawa T, Nau ME, Xu LL, Chilukuri RN, Makarem M, Zhang W, Petrovics G, Sesterhenn IA, McLeod DG, Moul JW, Vahey M and Srivastava S. Androgen-induced expression of endoplasmic reticulum (ER) stress response genes in prostate cancer cells. Oncogene 2002; 21:8749-58.
12. Krycer JR and Brown AJ. Cross-talk between the androgen receptor and the liver X receptor: implications for cholesterol homeostasis. J Biol Chem 2011; 286: 20637-47.
13. Tahir SA, Yang G, Ebara S, Timme TL, Satoh T, Li L, Goltsov A, Ittmann M, Morrisett JD and Thompson TC. Secreted caveolin-1 stimulates cell survival/clonal growth and contributes to metastasis in androgen insensitive prostate cancer. Cancer Res 2001; 61: 3882-5.
14. Zhuang L, Kim J, Adam RM, Solomon KR and Freeman MR. Cholesterol targeting alters lipid raft composition and cell survival in prostate cancer cells and xenografts. J Clin Invest 2005; 115: 959-68.
15. Zhuang L, Lin J, Lu ML, Solomon KR and Freeman MR. Cholesterol-rich lipid rafts mediate akt-regulated survival in prostate cancer cells. Cancer Res 2002; 62: 2227-31.
16. Marcelli M, Cunningham GR, Haidacher SJ, Padayatty SJ, Sturgis L, Kagan C and Denner L. Caspase-7 is activated during lovastatin-induced apoptosis of the prostate cancer cell line LNCaP. Cancer Res 1998; 58: 76-83.
17. Ahn J, Lim U, Weinstein SJ, Schatzkin A, Hayes RB, Virtamo J and Albanes D. Prediagnostic total and high-density lipoprotein cholesterol and risk of cancer. Cancer Epidemiol Biomarkers Prev 2009; 18: 2814-21.
18. Ahn J, Lim U, Weinstein SJ, Schatzkin A, Hayes RB, Virtamo J and Albanes D. Prediagnostic total and high-density lipoprotein cholesterol an risk of cancer. Cancer Epidemiol Biomarkers Prev 2009; 18: 2814-21.
19. Mondul AM, Weinstein SJ, Virtamo J and Albanes D. Serum total and HDL cholesterol and risk of prostate cancer. Cancer Causes Control 2011; 22: 1545-52.
20. Mostaghel EA, Solomon KR, Pelton K, Freeman MR and Montgomery RB. Impact of circulating cholesterol levels on growth and intratumoral androgen concentration of prostate tumors. PLoS One 2012; 7: e30062.
21. Marcelli M, Cunningham GR, Haidacher SJ, Padayatty SJ, Sturgis L, Kagan C and Denner L. Caspase-7 is activated during lovastatin-induced apoptosis of the prostate cancer cell line LNCaP. Cancer Res 1998; 58: 76-83.
22. Fukuchi J, Hiipakka RA, Kokontis JM, Hsu S, Ko AL, Fitzgerald ML and Liao S. Androgenic suppression of ATP-binding cassette transporter A1 expression in LNCaP human prostate cancer cells. Cancer Res 2004; 64: 7682-5.
23. Zhang Z, Rosen DG, Yao JL, Huang J, Liu J. Expression of p14ARF, p15INK4b, p16INK4a, and DCR2 increases during prostate cancer progression. Mod Pathol. 2006; 19:1339–1343.
24. Zeng L, Rowland RG, Lele SM, Kyprianou N. Apoptosis incidence and protein expression of p53, TGF-beta receptor II, p27Kip1, and Smad4 in benign, premalignant, and malignant human prostate. Hum Pathol. 2004; 35:290–297.
25. Yuan X, Balk SP. Mechanisms mediating androgen receptor reactivation after castration. Urol Oncol.

2009; 27:36–41.

1. Yeh S, Miyamoto H, Nishimura K, Kang H, Ludlow J, Hsiao P, et al. Retinoblastoma, a tumor suppressor, is a coactivator for the androgen receptor in human prostate cancer DU145 cells. Biochem Biophys Res Commun. 1998; 248:361–367.
2. Xu Y, Chen SY, Ross KN, Balk SP. Androgens induce prostate cancer cell proliferation through mammalian target of rapamycin activation and post-transcriptional increases in cyclin D proteins. Cancer Res. 2006; 66:7783–7792.
3. Wu TT, Wang JS, Jiaan BP, Yu CC, Tsai JY, Lin JT, et al. Role of p21(WAF1) and p27(KIP1) in predicting biochemical recurrence for organ-confined prostate adenocarcinoma. J Chin Med Assoc. 2007; 70:11–15.
4. Wolters T, Vissers KJ, Bangma CH, Schroder FH, van Leenders GJ. The value of EZH2, p27(kip1), BMI-1 and MIB-1 on biopsy specimens with low-risk prostate cancer in selecting men with significant prostate cancer at prostatectomy. BJU Int. 2010; 106:280–286.
5. Vis AN, van Rhijn BW, Noordzij MA, Schroder FH, van der Kwast TH. Value of tissue markers p27(kip1), MIB-1, and CD44s for the pre-operative prediction of tumour features in screen detected prostate cancer. J Pathol. 2002; 197:148–154.
6. Tran C, Ouk S, Clegg NJ, Chen Y, Watson PA, Arora V, et al. Development of a second-generation antiandrogen for treatment of advanced prostate cancer. Science. 2009; 324:787–790.
7. Sun H, Wang Y, Chinnam M, Zhang X, Hayward SW, Foster BA, et al. E2f binding-deficient Rb1 protein suppresses prostate tumor progression in vivo. Proc Natl Acad Sci USA. 2011; 108:704–709.
8. Stanbrough M, Bubley GJ, Ross K, Golub TR, Rubin MA, Penning TM, et al. Increased expression of genes converting adrenal androgens to testosterone in androgen-independent prostate cancer. Cancer Res. 2006; 66:2815–2825.
9. Shiraishi T, Watanabe M, Muneyuki T, Nakayama T, Morita J, Ito H, et al. A clinicopathological study of p53, p21 (WAF1/CIP1) and cyclin D1 expression in human prostate cancers. Urol Int. 1998; 61:90–94.
10. Sharma A, Yeow WS, Ertel A, Coleman I, Clegg N, Thangavel C, et al. The retinoblastoma tumor suppressor controls androgen signaling and human prostate cancer progression. J Clin Invest. 2010; 120:4478–4492.
11. Schiewer MJ, Morey LM, Burd CJ, Liu Y, Merry DE, Ho SM, et al. Cyclin D1 repressor domain mediates proliferation and survival in prostate cancer. Oncogene. 2009; 28:1016–1027.
12. Rosenbaum E, Hoque MO, Cohen Y, Zahurak M, Eisenberger MA, Epstein JI, et al. Promoter hypermethylation as an independent prognostic factor for relapse in patients with prostate cancerfollowing radical prostatectomy. Clin Cancer Res. 2005; 11:8321–8325.
13. Romics I, Banfi G, Szekely E, Krenacs T, Szende B. Expression of p21(waf1/cip1), p27 (kip1), p63 and androgen receptor in low and high Gleason score prostate cancer. Pathol Oncol Res. 2008; 14:307–311.
14. Reutens AT, Fu M, Wang C, Albanese C, McPhaul MJ, Sun Z, et al. Cyclin D1 binds the androgen receptor and regulates hormone-dependent signaling in a p300/CBP-associated factor (P/CAF)- dependent manner. Mol Endocrinol. 2001; 15:797–811.
15. Peng Y, Chen F, Melamed J, Chiriboga L, Wei J, Kong X, et al. Distinct nuclear and cytoplasmic functions of androgen receptor cofactor p44 and association with androgen-independent prostate cancer. Proc Natl Acad Sci USA. 2008; 105:5236–5241.
16. Ozen M, Ittmann M. Increased expression and activity of CDC25C phosphatase and an alternatively spliced variant in prostate cancer. Clin Cancer Res. 2005; 11:4701–4706.
17. Olshavsky NA, Groh EM, Comstock CE, Morey LM, Wang Y, Revelo MP, et al. Cyclin D3 action in androgen receptor regulation and prostate cancer. Oncogene. 2008; 27:3111–3121.
18. Nikoleishvili D, Pertia A, Trsintsadze O, Gogokhia N, Managadze L, Chkhotua A. Expression of p27((Kip1)), cyclin D3 and Ki67 in BPH, prostate cancer and hormone-treated prostate cancer cells. Int Urol Nephrol. 2008; 40:953–959.
19. Nguyen PL, Lin DI, Lei J, Fiorentino M, Mueller E, Weinstein MH, et al. The impact of Skp2 overexpression on recurrence-free survival following radical prostatectomy. Urol Oncol. 2009.
20. Mallik I, Davila M, Tapia T, Schanen B, Chakrabarti R. Androgen regulates Cdc6 transcription through interactions between androgen receptor and E2F transcription factor in prostate cancer cells. Biochim Biophys Acta. 2008; 1783:1737–1744.
21. Maddison LA, Huss WJ, Barrios RM, Greenberg NM. Differential expression of cell cycle regulatory molecules and evidence for a “cyclin switch” during progression of prostate cancer. Prostate. 2004; 58:335–344.
22. Lu L, Schulz H, Wolf DA. The F-box protein SKP2 mediates androgen control of p27 stability in LNCaP human prostate cancer cells. BMC Cell Biol. 2002; 3:22.
23. Locke JA, Guns ES, Lubik AA, Adomat HH, Hendy SC, Wood CA, et al. Androgen levels increase by intratumoral de novo steroidogenesis during progression of castration-resistant prostate cancer. Cancer Res. 2008; 68:6407–6415.
24. Li R, Wheeler TM, Dai H, Sayeeduddin M, Scardino PT, Frolov A, et al. Biological correlates of p27 compartmental expression in prostate cancer. J Urol. 2006; 175:528–532.
25. Knudsen KE, Scher HI. Starving the addiction: new opportunities for durable suppression of AR signaling in prostate cancer. Clin Cancer Res. 2009; 15:4792–4798.
26. Knudsen KE, Cavenee WK, Arden KC. D-type cyclins complex with the androgen receptor and inhibit its transcriptional transactivation ability. Cancer Res. 1999; 59:2297–2301.
27. Knudsen KE, Arden KC, Cavenee WK. Multiple G1 regulatory elements control the androgendependent proliferation of prostatic carcinoma cells. J Biol Chem. 1998; 273:20213–20222.
28. Jin F, Fondell JD. A novel androgen receptor-binding element modulates Cdc6 transcription in prostate cancer cells during cell-cycle progression. Nucleic Acids Res. 2009; 37:4826–4838.
29. Henrique R, Costa VL, Cerveira N, Carvalho AL, Hoque MO, Ribeiro FR, et al. Hypermethylation of Cyclin D2 is associated with loss of mRNA expression and tumor development in prostate cancer. J Mol Med. 2006; 84:911–918.
30. Heemers HV, Regan KM, Schmidt LJ, Anderson SK, Ballman KV, Tindall DJ. Androgen modulation of coregulator expression in prostate cancer cells. Mol Endocrinol. 2009; 23:572–583.
31. Guo Z, Yang X, Sun F, Jiang R, Linn DE, Chen H, et al. A novel androgen receptor splice variant is up-regulated during prostate cancer progression and promotes androgen depletion-resistant growth. Cancer Res. 2009; 69:2305–2313.
32. Fernandez PL, Arce Y, Farre X, Martinez A, Nadal A, Rey MJ, et al. Expression of p27/Kip1 is downregulated in human prostate carcinoma progression. J Pathol. 1999; 187:563–566.
33. Doganavsargil B, Simsir A, Boyacioglu H, Cal C, Hekimgil M. A comparison of p21 and p27 immunoexpression in benign glands, prostatic intraepithelial neoplasia and prostate adenocarcinoma. BJU Int. 2006; 97:644–648.
34. Ding Z, Wu CJ, Chu GC, Xiao Y, Ho D, Zhang J, et al. SMAD4-dependent barrier constrains prostate cancer growth and metastatic progression. Nature. 2011; 470:269–273.
35. Claudio PP, Zamparelli A, Garcia FU, Claudio L, Ammirati G, Farina A, et al. Expression of cellcycle- regulated proteins pRb2/p130, p107, p27(kip1), p53, mdm-2, and Ki-67 (MIB-1) in prostatic gland adenocarcinoma. Clin Cancer Res. 2002; 8:1808–1815.
36. Chiu YT, Han HY, Leung SC, Yuen HF, Chau CW, Guo Z, et al. CDC25A functions as a novel Ar corepressor in prostate cancer cells. J Mol Biol. 2009; 385:446–456.
37. Chen S, Xu Y, Yuan X, Bubley GJ, Balk SP. Androgen receptor phosphorylation and stabilization in prostate cancer by cyclin-dependent kinase 1. Proc Natl Acad Sci USA. 2006; 103:15969–15974.
38. Burd CJ, Petre CE, Morey LM, Wang Y, Revelo MP, Haiman CA, et al. Cyclin D1b variant influences prostate cancer growth through aberrant androgen receptor regulation. Proc Natl Acad Sci USA.

2006; 103:2190–2195.

1. Bohrer LR, Chen S, Hallstrom TC, Huang H. Androgens suppress EZH2 expression via retinoblastoma (RB) and p130-dependent pathways: a potential mechanism of androgen-refractory progression of prostate cancer. Endocrinology. 2010; 151:5136–5145.
2. Ben-Izhak O, Lahav-Baratz S, Meretyk S, Ben-Eliezer S, Sabo E, Dirnfeld M, et al. Inverse relationship between Skp2 ubiquitin ligase and the cyclin dependent kinase inhibitor p27Kip1 in prostate cancer. J Urol. 2003; 170:241–245.
3. Sun C, Dobi A, Mohamed A*, et al*. TMPRSS2-ERG fusion, a common genomic alteration in prostate cancer activates C-MYC and abrogates prostate epithelial differentiation. Oncogene. 2008;27(40):5348-53.
4. Meeker AK, Hicks JL, Platz EA, March GE, Bennett CJ, De Marzo AM. Telomere shortening is an early somatic DNA alteration in human prostate tumorigenesis. Cancer Res.2002;62:6405-9.
5. Zong Y, Xin L, Goldstein AS, Lawson DA, Teitell MA, Witte ON. ETS family transcription factors collaborate with alternative signaling pathways to induce carcinoma from adult murine prostate cells. Proc Natl Acad Sci U S A. 2009;106(30):12465-70.
6. Lamb LE, Knudsen BS, Miranti CK. E-cadherin- mediated survival of androgen-receptorexpressing secretory prostate epithelial cells derived from a stratified in vitro differentiationmodel. J Cell Sci. 2010;123(pt 2):266-76.
7. Yu J, Yu J, Mani RS*, et al*. An integrated network of androgen receptor, polycomb, and TMPRSS2-ERG gene fusions in prostate cancer progression. Cancer Cell. 2010;17(5):443-54.
8. Latil A, Vidaud D, Valeri A*, et al*. htert expression correlates with MYC over-expression in human prostate cancer. Int J Cancer. 2000;89(2):172-6.
9. Kunderfranco P, Mello-Grand M, Cangemi R*, et al*. ETS transcription factors control transcription of EZH2 and epigenetic silencing of the tumor suppressor gene Nkx3.1 in prostate cancer. PloS One. 2010;5(5):e10547.
10. Wang J, Kim J, Roh M*, et al*. Pim1 kinase synergizes with c-MYC to induce advanced prostate carcinoma. Oncogene. 2010;29(17):2477-87.
11. van der Poel HG, Zevenhoven J, Bergman AM. Pim1 regulates androgen-dependent survival signaling in prostate cancer cells. Urol Int. 2010;84(2):212-20.
12. McConkey DJ, Lin Y, Nutt LK, Ozel HZ, Newman RA. Cardiac glycosides stimulate Ca2+ increases and apoptosis in androgen-independent, metastatic human prostate adenocarcinoma cells. Cancer Res. 2000;60(14):3807-12.
13. Lin H, Juang JL, Wang PS. Involvement of Cdk5/ p25 in digoxin-triggered prostate cancer cell

apoptosis. J Biol Chem. 2004;279(28):29302-7.

1. Miller GJ. Vitamin D and prostate cancer: biologic interactions and clinical potentials. Cancer Metastasis Rev. 1998;17(4):353-60.
2. Rohan JN, Weigel NL. 1Alpha,25-dihydroxyvitamin D3 reduces c-Myc expression, inhibiting proliferation and causing G1 accumulation in C4-2 prostate cancer cells. Endocrinology. 2009;150(5):2046-54.
3. Gil J, Kerai P, Lleonart M*, et al*. Immortalization of primary human prostate epithelial cells by c-Myc. Cancer Res. 2005;65(6):2179-85.
4. Yeager M, Orr N, Hayes RB*, et al*. Genomewide association study of prostate cancer identifies a second risk locus at 8q24. Nat Genet. 2007;39(5):645-9.
5. Al Olama AA, Kote-Jarai Z, Giles GG*, et al*. Multiple loci on 8q24 associated with prostate cancer susceptibility. Nat Genet. 2009;41(10):1058-60.
6. Whitaker HC, Girling J, Warren AY, Leung H, Mills IG, Neal DE. Alterations in beta-catenin expression and localization in prostate cancer. Prostate. 2008;68(11):1196-205.
7. Fiorentino M, Zadra G, Palescandolo E*, et al*. Overexpression of fatty acid synthase is associated with palmitoylation of Wnt1 and cytoplasmic stabilization of beta-catenin in prostate cancer. Lab Invest. 2008;88(12):1340-8.
8. Wang L, Liu R, Li W*, et al*. Somatic single hits inactivate the X-linked tumor suppressor FOXP3 in the prostate. Cancer Cell. 2009;16(4):336-46.
9. Bruxvoort KJ, Charbonneau HM, Giambernardi TA*, et al*. Inactivation of Apc in the mouse prostate causes prostate carcinoma. Cancer Res. 2007;67(6):2490-6.
10. Robinson DR, Zylstra CR, Williams BO. Wnt signaling and prostate cancer. Curr Drug Targets. 2008;9(7):571-80.
11. Saramaki O, Willi N, Bratt O*, et al*. Amplification of EIF3S3 gene is associated with advanced stage in prostate cancer. Am J Pathol. 2001;159(6):2089-94.
12. Bova GS, Isaacs WB. Review of allelic loss and gain in prostate cancer. World J Urol. 1996;14(5):338-46.
13. Tomlins SA, Mehra R, Rhodes DR*, et al*. Integrative molecular concept modeling of prostate cancer progression. Nat Genet. 2007;39(1):41-51.
14. Kumar-Sinha C, Tomlins SA, Chinnaiyan AM. Recurrent gene fusions in prostate cancer. Nat Rev Cancer. 2008;8(7):497-511.
15. Recchia I, Rucci N, Festuccia C et al. Pyrrolopyrimidine c-Src inhibitors reduce growth, adhesion, motility and invasion of prostate cancer cells in vitro. Eur J Cancer 2003; 39: 1927–1935.
16. Nam S, Kim D, Cheng JQ et al. Action of the Src family kinase inhibitor, dasatinib (BMS-354825), on human prostate cancer cells. Cancer Res 2005; 65: 9185–9189.
17. Uehara H, Kim SJ, Karashima T et al. Effects of blocking platelet-derived growth factor-receptor signaling in a mouse model of experimental prostate cancer bone metastases. J Natl Cancer Inst 2003; 95: 458–470.
18. [Kuperstein I](http://www.ncbi.nlm.nih.gov/pubmed/?term=Kuperstein I%5BAuthor%5D&cauthor=true&cauthor_uid=26192618). *et al*. Atlas of Cancer Signalling Network: a systems biology resource for integrative analysis of cancer data with google maps. Oncogenesis  4, e160 (2015).
19. Pawson T. & Linding R. Network Medicine. FEBS Lett 582, 1266-1270 (2008).
20. Menche J. *et al*. Disease networks. Uncovering disease-disease relationships through the incomplete interactome. Science 347, 1257601 (2015).
21. Keshava Prasad T.S. *et al*. Human Protein Reference Database—2009 update. Nucleic Acids Res 37, D767-72 (2009).
22. Wain H.M., Lush M.J., Ducluzeau F., Khodiyar V.K. & Povey S. Genew: the Human Gene Nomenclature Database, 2004 updates. Nucleic Acids Res 32, D255-7 (2004).
23. Kanehisa M. The KEGG database. Novartis Found Symp 247, 91-101 (2002).
24. [Cerami E.G](http://www.ncbi.nlm.nih.gov/pubmed/?term=Cerami EG%5BAuthor%5D&cauthor=true&cauthor_uid=21071392). *et al*. Pathway Commons, a web resource for biological pathway data. Nucleic Acids Res 39, D685-90 (2011).
25. [Pico A.R](http://www.ncbi.nlm.nih.gov/pubmed/?term=Pico AR%5BAuthor%5D&cauthor=true&cauthor_uid=18651794). *et al*. WikiPathways: pathway editing for the people. [PLoS Biol](http://www.ncbi.nlm.nih.gov/pubmed/18651794) 6, e184 (2008).
26. Zhang B., Kirov S. & Snoddy J. WebGestalt: an integrated system for exploring gene sets in various biological contexts. Nucleic Acids Res 33, W741–W748 (2005).
27. Batagelj V. & Mrvar A. Pajek: Program for large network analysis. Connections 21, 47-57 (1998).
28. Shannon P. *et al*. Cytoscape: a software environment for integrated models of biomolecular interaction networks. Genome Res 11, 2498-504 (2003).
29. P [Chen J](http://www.ncbi.nlm.nih.gov/pubmed/?term=Chen J%5BAuthor%5D&cauthor=true&cauthor_uid=18483269)^1^, [Deng F](http://www.ncbi.nlm.nih.gov/pubmed/?term=Deng F%5BAuthor%5D&cauthor=true&cauthor_uid=18483269), [Singh SV](http://www.ncbi.nlm.nih.gov/pubmed/?term=Singh SV%5BAuthor%5D&cauthor=true&cauthor_uid=18483269), [Wang QJ](http://www.ncbi.nlm.nih.gov/pubmed/?term=Wang QJ%5BAuthor%5D&cauthor=true&cauthor_uid=18483269). Protein kinase D3 (PKD3) contributes to prostate cancer cell growth and survival through a PKCepsilon/PKD3 pathway downstream of Akt and ERK 1/2. [Cancer Res.](http://www.ncbi.nlm.nih.gov/pubmed/?term=Cancer+Res.+2008+May+15%3B68(10)%3A3844-53.) 2008 May 15;68(10):3844-53.
30. [Zoubeidi A](http://www.ncbi.nlm.nih.gov/pubmed/?term=Zoubeidi A%5BAuthor%5D&cauthor=true&cauthor_uid=19147545)^1^, [Rocha J](http://www.ncbi.nlm.nih.gov/pubmed/?term=Rocha J%5BAuthor%5D&cauthor=true&cauthor_uid=19147545), [Zouanat FZ](http://www.ncbi.nlm.nih.gov/pubmed/?term=Zouanat FZ%5BAuthor%5D&cauthor=true&cauthor_uid=19147545), [Hamel L](http://www.ncbi.nlm.nih.gov/pubmed/?term=Hamel L%5BAuthor%5D&cauthor=true&cauthor_uid=19147545). The Fer tyrosine kinase cooperates with interleukin-6 to activate signal transducer and activator of transcription 3 and promote human prostate cancer cell growth.
31. [Takeuchi A](http://www.ncbi.nlm.nih.gov/pubmed/?term=Takeuchi A%5BAuthor%5D&cauthor=true&cauthor_uid=24491388)^1^, [Shiota M](http://www.ncbi.nlm.nih.gov/pubmed/?term=Shiota M%5BAuthor%5D&cauthor=true&cauthor_uid=24491388)^1^, [Beraldi E](http://www.ncbi.nlm.nih.gov/pubmed/?term=Beraldi E%5BAuthor%5D&cauthor=true&cauthor_uid=24491388)^1^, [Thaper D](http://www.ncbi.nlm.nih.gov/pubmed/?term=Thaper D%5BAuthor%5D&cauthor=true&cauthor_uid=24491388). Insulin-like growth factor-I induces CLU expression through Twist1 to promote prostate cancer growth. Mol Cell Endocrinol. 2014 Mar 25;384(1-2):117-25.

# [**Zhuang L**](http://www.ncbi.nlm.nih.gov/pubmed/?term=Zhuang L%5BAuthor%5D&cauthor=true&cauthor_uid=15776112)^1^, [**Kim J**](http://www.ncbi.nlm.nih.gov/pubmed/?term=Kim J%5BAuthor%5D&cauthor=true&cauthor_uid=15776112), [**Adam RM**](http://www.ncbi.nlm.nih.gov/pubmed/?term=Adam RM%5BAuthor%5D&cauthor=true&cauthor_uid=15776112), [**Solomon KR**](http://www.ncbi.nlm.nih.gov/pubmed/?term=Solomon KR%5BAuthor%5D&cauthor=true&cauthor_uid=15776112). Cholesterol targeting alters lipid raft composition and cell survival in prostate cancer cells and xenografts. J Clin Invest. 2005 Apr;115(4):959-68.

1. [Figel S](http://www.ncbi.nlm.nih.gov/pubmed/?term=Figel S%5BAuthor%5D&cauthor=true&cauthor_uid=21355844)^1^, [Gelman IH](http://www.ncbi.nlm.nih.gov/pubmed/?term=Gelman IH%5BAuthor%5D&cauthor=true&cauthor_uid=21355844).Focal adhesion kinase controls prostate cancer progression via intrinsic kinase and scaffolding functions. Anticancer Agents Med Chem. 2011 Sep;11(7):607-16.

# [**Conley-LaComb MK**](http://www.ncbi.nlm.nih.gov/pubmed/?term=Conley-LaComb MK%5BAuthor%5D&cauthor=true&cauthor_uid=23902739)**^1^**, [**Saliganan A**](http://www.ncbi.nlm.nih.gov/pubmed/?term=Saliganan A%5BAuthor%5D&cauthor=true&cauthor_uid=23902739), [**Kandagatla P**](http://www.ncbi.nlm.nih.gov/pubmed/?term=Kandagatla P%5BAuthor%5D&cauthor=true&cauthor_uid=23902739). PTEN loss mediated Akt activation promotes prostate tumor growth and metastasis via CXCL12/CXCR4 signaling. Mol Cancer. 2013 Jul 31;12(1):85.

1. [Xu B](http://www.ncbi.nlm.nih.gov/pubmed/?term=Xu B%5BAuthor%5D&cauthor=true&cauthor_uid=22161865)^1^, [Wang N](http://www.ncbi.nlm.nih.gov/pubmed/?term=Wang N%5BAuthor%5D&cauthor=true&cauthor_uid=22161865), [Wang X](http://www.ncbi.nlm.nih.gov/pubmed/?term=Wang X%5BAuthor%5D&cauthor=true&cauthor_uid=22161865), [Tong N](http://www.ncbi.nlm.nih.gov/pubmed/?term=Tong N%5BAuthor%5D&cauthor=true&cauthor_uid=22161865). MiR-146a suppresses tumor growth and progression by targeting EGFR pathway and in a p-ERK-dependent manner in castration-resistant prostatecancer. Prostate. 2012 Aug 1;72(11):1171-8.
2. [Barfeld SJ](http://www.ncbi.nlm.nih.gov/pubmed/?term=Barfeld SJ%5BAuthor%5D&cauthor=true&cauthor_uid=24497572)^1^, [Itkonen HM](http://www.ncbi.nlm.nih.gov/pubmed/?term=Itkonen HM%5BAuthor%5D&cauthor=true&cauthor_uid=24497572)^1^, [Urbanucci A](http://www.ncbi.nlm.nih.gov/pubmed/?term=Urbanucci A%5BAuthor%5D&cauthor=true&cauthor_uid=24497572). Androgen-regulated metabolism and biosynthesis in prostate cancer. Endocr Relat Cancer. 2014 Aug;21(4):T57-66.

# [**Chen J**](http://www.ncbi.nlm.nih.gov/pubmed/?term=Chen J%5BAuthor%5D&cauthor=true&cauthor_uid=23231703)^1^, [**Jiao L**](http://www.ncbi.nlm.nih.gov/pubmed/?term=Jiao L%5BAuthor%5D&cauthor=true&cauthor_uid=23231703), [**Xu C**](http://www.ncbi.nlm.nih.gov/pubmed/?term=Xu C%5BAuthor%5D&cauthor=true&cauthor_uid=23231703), [**Yu Y**](http://www.ncbi.nlm.nih.gov/pubmed/?term=Yu Y%5BAuthor%5D&cauthor=true&cauthor_uid=23231703), [**Zhang Z**](http://www.ncbi.nlm.nih.gov/pubmed/?term=Zhang Z%5BAuthor%5D&cauthor=true&cauthor_uid=23231703). Neural protein gamma-synuclein interacting with androgen receptor promotes human prostate cancer progression. BMC Cancer. 2012 Dec 11;12:593.

# [**Zheng Y**](http://www.ncbi.nlm.nih.gov/pubmed/?term=Zheng Y%5BAuthor%5D&cauthor=true&cauthor_uid=23398121)^1^, [**Tyner AL**](http://www.ncbi.nlm.nih.gov/pubmed/?term=Tyner AL%5BAuthor%5D&cauthor=true&cauthor_uid=23398121). Context-specific protein tyrosine kinase 6 (PTK6) signalling in prostate cancer. Eur J Clin Invest. 2013 Apr;43(4):397-404.

1. [Bonci D](http://www.ncbi.nlm.nih.gov/pubmed/?term=Bonci D%5BAuthor%5D&cauthor=true&cauthor_uid=18931683), [Coppola V](http://www.ncbi.nlm.nih.gov/pubmed/?term=Coppola V%5BAuthor%5D&cauthor=true&cauthor_uid=18931683), [Musumeci M](http://www.ncbi.nlm.nih.gov/pubmed/?term=Musumeci M%5BAuthor%5D&cauthor=true&cauthor_uid=18931683), [Addario A](http://www.ncbi.nlm.nih.gov/pubmed/?term=Addario A%5BAuthor%5D&cauthor=true&cauthor_uid=18931683), [Giuffrida R](http://www.ncbi.nlm.nih.gov/pubmed/?term=Giuffrida R%5BAuthor%5D&cauthor=true&cauthor_uid=18931683).The miR-15a-miR-16-1 cluster controls prostate cancer by targeting multiple oncogenic activities. Nat Med. 2008 Nov;14(11):1271-7.
2. [Kahl P](http://www.ncbi.nlm.nih.gov/pubmed/?term=Kahl P%5BAuthor%5D&cauthor=true&cauthor_uid=17145880)^1^, [Gullotti L](http://www.ncbi.nlm.nih.gov/pubmed/?term=Gullotti L%5BAuthor%5D&cauthor=true&cauthor_uid=17145880), [Heukamp LC](http://www.ncbi.nlm.nih.gov/pubmed/?term=Heukamp LC%5BAuthor%5D&cauthor=true&cauthor_uid=17145880), [Wolf S](http://www.ncbi.nlm.nih.gov/pubmed/?term=Wolf S%5BAuthor%5D&cauthor=true&cauthor_uid=17145880). Androgen receptor coactivators lysine-specific histone demethylase 1 and four and a half LIM domain protein 2 predict risk of prostate cancer recurrence. Cancer Res. 2006 Dec 1;66(23):11341-7.

# [**Haflidadóttir BS**](http://www.ncbi.nlm.nih.gov/pubmed/?term=Haflidadóttir BS%5BAuthor%5D&cauthor=true&cauthor_uid=23951320)^1^, [**Larne O**](http://www.ncbi.nlm.nih.gov/pubmed/?term=Larne O%5BAuthor%5D&cauthor=true&cauthor_uid=23951320), [**Martin M**](http://www.ncbi.nlm.nih.gov/pubmed/?term=Martin M%5BAuthor%5D&cauthor=true&cauthor_uid=23951320), [**Persson M**](http://www.ncbi.nlm.nih.gov/pubmed/?term=Persson M%5BAuthor%5D&cauthor=true&cauthor_uid=23951320). Upregulation of miR-96 enhances cellular proliferation of prostate cancer cells through FOXO1. PLoS One. 2013 Aug 12;8(8):e72400.

# [**Wang Y**](http://www.ncbi.nlm.nih.gov/pubmed/?term=Wang Y%5BAuthor%5D&cauthor=true&cauthor_uid=18776922)^1^, [**Mikhailova M**](http://www.ncbi.nlm.nih.gov/pubmed/?term=Mikhailova M%5BAuthor%5D&cauthor=true&cauthor_uid=18776922), [**Bose S**](http://www.ncbi.nlm.nih.gov/pubmed/?term=Bose S%5BAuthor%5D&cauthor=true&cauthor_uid=18776922), [**Pan CX**](http://www.ncbi.nlm.nih.gov/pubmed/?term=Pan CX%5BAuthor%5D&cauthor=true&cauthor_uid=18776922). Regulation of androgen receptor transcriptional activity by rapamycin in prostate cancer cell proliferation and survival. Oncogene. 2008 Nov 27;27(56):7106-17.

# [**Obinata D**](http://www.ncbi.nlm.nih.gov/pubmed/?term=Obinata D%5BAuthor%5D&cauthor=true&cauthor_uid=21647875)^1^, [**Takayama K**](http://www.ncbi.nlm.nih.gov/pubmed/?term=Takayama K%5BAuthor%5D&cauthor=true&cauthor_uid=21647875), [**Urano T**](http://www.ncbi.nlm.nih.gov/pubmed/?term=Urano T%5BAuthor%5D&cauthor=true&cauthor_uid=21647875), [**Murata T**](http://www.ncbi.nlm.nih.gov/pubmed/?term=Murata T%5BAuthor%5D&cauthor=true&cauthor_uid=21647875). ARFGAP3, an androgen target gene, promotes prostate cancer cell proliferation and migration. Int J Cancer. 2012 May 15;130(10):2240-8.

# [**Kim JY**](http://www.ncbi.nlm.nih.gov/pubmed/?term=Kim JY%5BAuthor%5D&cauthor=true&cauthor_uid=23550155)^1^, [**Valencia T**](http://www.ncbi.nlm.nih.gov/pubmed/?term=Valencia T%5BAuthor%5D&cauthor=true&cauthor_uid=23550155), [**Abu-Baker S**](http://www.ncbi.nlm.nih.gov/pubmed/?term=Abu-Baker S%5BAuthor%5D&cauthor=true&cauthor_uid=23550155), [**Linares J**](http://www.ncbi.nlm.nih.gov/pubmed/?term=Linares J%5BAuthor%5D&cauthor=true&cauthor_uid=23550155). c-Myc phosphorylation by PKCζ represses prostate tumorigenesis. Proc Natl Acad Sci U S A. 2013 Apr 16;110(16):6418-23.

1. [Lu S](http://www.ncbi.nlm.nih.gov/pubmed/?term=Lu S%5BAuthor%5D&cauthor=true&cauthor_uid=19621387)^1^, [Archer MC](http://www.ncbi.nlm.nih.gov/pubmed/?term=Archer MC%5BAuthor%5D&cauthor=true&cauthor_uid=19621387). Sp1 coordinately regulates de novo lipogenesis and proliferation in cancer cells. Int J Cancer. 2010 Jan 15;126(2):416-25.
2. [Mo W](http://www.ncbi.nlm.nih.gov/pubmed/?term=Mo W%5BAuthor%5D&cauthor=true&cauthor_uid=23451058)^1^, [Zhang J](http://www.ncbi.nlm.nih.gov/pubmed/?term=Zhang J%5BAuthor%5D&cauthor=true&cauthor_uid=23451058), [Li X](http://www.ncbi.nlm.nih.gov/pubmed/?term=Li X%5BAuthor%5D&cauthor=true&cauthor_uid=23451058), [Meng D](http://www.ncbi.nlm.nih.gov/pubmed/?term=Meng D%5BAuthor%5D&cauthor=true&cauthor_uid=23451058), [Gao Y](http://www.ncbi.nlm.nih.gov/pubmed/?term=Gao Y%5BAuthor%5D&cauthor=true&cauthor_uid=23451058). Identification of novel AR-targeted microRNAs mediating androgen signalling through critical pathways to regulate cell viability in prostate cancer. PLoS One. 2013;8(2):e56592.

# [**Muniyan S**](http://www.ncbi.nlm.nih.gov/pubmed/?term=Muniyan S%5BAuthor%5D&cauthor=true&cauthor_uid=23698773)^1^, [**Chaturvedi NK**](http://www.ncbi.nlm.nih.gov/pubmed/?term=Chaturvedi NK%5BAuthor%5D&cauthor=true&cauthor_uid=23698773), [**Dwyer JG**](http://www.ncbi.nlm.nih.gov/pubmed/?term=Dwyer JG%5BAuthor%5D&cauthor=true&cauthor_uid=23698773). Human prostatic acid phosphatase: structure, function and regulation. Int J Mol Sci. 2013 May 21;14(5):10438-64.

1. [Qu F](http://www.ncbi.nlm.nih.gov/pubmed/?term=Qu F%5BAuthor%5D&cauthor=true&cauthor_uid=23417242)^1^, [Cui X](http://www.ncbi.nlm.nih.gov/pubmed/?term=Cui X%5BAuthor%5D&cauthor=true&cauthor_uid=23417242), [Hong Y](http://www.ncbi.nlm.nih.gov/pubmed/?term=Hong Y%5BAuthor%5D&cauthor=true&cauthor_uid=23417242), [Wang J](http://www.ncbi.nlm.nih.gov/pubmed/?term=Wang J%5BAuthor%5D&cauthor=true&cauthor_uid=23417242). MicroRNA-185 suppresses proliferation, invasion, migration, and tumorigenicity of human prostate cancer cells through targeting androgen receptor. Mol Cell Biochem. 2013 May;377(1-2):121-30.

# [**Senapati S**](http://www.ncbi.nlm.nih.gov/pubmed/?term=Senapati S%5BAuthor%5D&cauthor=true&cauthor_uid=19946339)^1^, [**Rachagani S**](http://www.ncbi.nlm.nih.gov/pubmed/?term=Rachagani S%5BAuthor%5D&cauthor=true&cauthor_uid=19946339), [**Chaudhary K**](http://www.ncbi.nlm.nih.gov/pubmed/?term=Chaudhary K%5BAuthor%5D&cauthor=true&cauthor_uid=19946339), [**Johansson SL**](http://www.ncbi.nlm.nih.gov/pubmed/?term=Johansson SL%5BAuthor%5D&cauthor=true&cauthor_uid=19946339).Overexpression of macrophage inhibitory cytokine-1 induces metastasis of human prostate cancer cells through the FAK-RhoA signaling pathway. Oncogene. 2010 Mar 4;29(9):1293-302.

1. [Gao L](http://www.ncbi.nlm.nih.gov/pubmed/?term=Gao L%5BAuthor%5D&cauthor=true&cauthor_uid=20482314)^1^, [Smith RS](http://www.ncbi.nlm.nih.gov/pubmed/?term=Smith RS%5BAuthor%5D&cauthor=true&cauthor_uid=20482314), [Chen LM](http://www.ncbi.nlm.nih.gov/pubmed/?term=Chen LM%5BAuthor%5D&cauthor=true&cauthor_uid=20482314). Tissue kallikrein promotes prostate cancer cell migration and invasion via a protease-activated receptor-1-dependent signaling pathway. Biol Chem. 2010 Jul;391(7):803-12.
2. [Trerotola M](http://www.ncbi.nlm.nih.gov/pubmed/?term=Trerotola M%5BAuthor%5D&cauthor=true&cauthor_uid=23536555)^1^, [Jernigan DL](http://www.ncbi.nlm.nih.gov/pubmed/?term=Jernigan DL%5BAuthor%5D&cauthor=true&cauthor_uid=23536555), [Liu Q](http://www.ncbi.nlm.nih.gov/pubmed/?term=Liu Q%5BAuthor%5D&cauthor=true&cauthor_uid=23536555), [Siddiqui J](http://www.ncbi.nlm.nih.gov/pubmed/?term=Siddiqui J%5BAuthor%5D&cauthor=true&cauthor_uid=23536555). Trop-2 promotes prostate cancer metastasis by modulating β(1) integrin functions. Cancer Res. 2013 May 15;73(10):3155-67.
3. [Kojima S](http://www.ncbi.nlm.nih.gov/pubmed/?term=Kojima S%5BAuthor%5D&cauthor=true&cauthor_uid=24284362)^1^, [Enokida H](http://www.ncbi.nlm.nih.gov/pubmed/?term=Enokida H%5BAuthor%5D&cauthor=true&cauthor_uid=24284362)^2^, [Yoshino H](http://www.ncbi.nlm.nih.gov/pubmed/?term=Yoshino H%5BAuthor%5D&cauthor=true&cauthor_uid=24284362)^2^, [Itesako T](http://www.ncbi.nlm.nih.gov/pubmed/?term=Itesako T%5BAuthor%5D&cauthor=true&cauthor_uid=24284362).The tumor-suppressive microRNA-143/145 cluster inhibits cell migration and invasion by targeting GOLM1 in prostate cancer. J Hum Genet. 2014 Feb;59(2):78-87.
4. [Vo BT](http://www.ncbi.nlm.nih.gov/pubmed/?term=Vo BT%5BAuthor%5D&cauthor=true&cauthor_uid=23515290)^1^, [Morton D Jr](http://www.ncbi.nlm.nih.gov/pubmed/?term=Morton D Jr%5BAuthor%5D&cauthor=true&cauthor_uid=23515290), [Komaragiri S](http://www.ncbi.nlm.nih.gov/pubmed/?term=Komaragiri S%5BAuthor%5D&cauthor=true&cauthor_uid=23515290). TGF-β effects on prostate cancer cell migration and invasion are mediated by PGE2 through activation of PI3K/AKT/mTOR pathway. Endocrinology. 2013 May;154(5):1768-79.
5. [Lin TH](http://www.ncbi.nlm.nih.gov/pubmed/?term=Lin TH%5BAuthor%5D&cauthor=true&cauthor_uid=23845726)^1^, [Liu HH](http://www.ncbi.nlm.nih.gov/pubmed/?term=Liu HH%5BAuthor%5D&cauthor=true&cauthor_uid=23845726), [Tsai TH](http://www.ncbi.nlm.nih.gov/pubmed/?term=Tsai TH%5BAuthor%5D&cauthor=true&cauthor_uid=23845726), [Chen C](http://www.ncbi.nlm.nih.gov/pubmed/?term=Chen CC%5BAuthor%5D&cauthor=true&cauthor_uid=23845726). CCL2 increases αvβ3 integrin expression and subsequently promotes prostate cancer migration. Biochim Biophys Acta. 2013 Oct;1830(10):4917-27.
6. [Sakamoto S](http://www.ncbi.nlm.nih.gov/pubmed/?term=Sakamoto S%5BAuthor%5D&cauthor=true&cauthor_uid=20160039)^1^, [McCann RO](http://www.ncbi.nlm.nih.gov/pubmed/?term=McCann RO%5BAuthor%5D&cauthor=true&cauthor_uid=20160039), [Dhir R](http://www.ncbi.nlm.nih.gov/pubmed/?term=Dhir R%5BAuthor%5D&cauthor=true&cauthor_uid=20160039). Talin1 promotes tumor invasion and metastasis via focal adhesion signaling and anoikis resistance. Cancer Res. 2010 Mar 1;70(5):1885-95.
7. [Sawhney RS](http://www.ncbi.nlm.nih.gov/pubmed/?term=Sawhney RS%5BAuthor%5D&cauthor=true&cauthor_uid=19089993)^1^, [Liu W](http://www.ncbi.nlm.nih.gov/pubmed/?term=Liu W%5BAuthor%5D&cauthor=true&cauthor_uid=19089993), [Brattain MG](http://www.ncbi.nlm.nih.gov/pubmed/?term=Brattain MG%5BAuthor%5D&cauthor=true&cauthor_uid=19089993).A novel role of ERK5 in integrin-mediated cell adhesion and motility in cancer cells via Fak signaling. J Cell Physiol. 2009 Apr;219(1):152-61.
8. [Liu X](http://www.ncbi.nlm.nih.gov/pubmed/?term=Liu X%5BAuthor%5D&cauthor=true&cauthor_uid=21369706)^1^, [Yan Z](http://www.ncbi.nlm.nih.gov/pubmed/?term=Yan Z%5BAuthor%5D&cauthor=true&cauthor_uid=21369706), [Huang L](http://www.ncbi.nlm.nih.gov/pubmed/?term=Huang L%5BAuthor%5D&cauthor=true&cauthor_uid=21369706), [Guo M](http://www.ncbi.nlm.nih.gov/pubmed/?term=Guo M%5BAuthor%5D&cauthor=true&cauthor_uid=21369706). Cell surface heat shock protein 90 modulates prostate cancer cell adhesion and invasion through the integrin-β1/focal adhesion kinase/c-Src signaling pathway. Oncol Rep. 2011 May;25(5):1343-51.
9. [Hyder CL](http://www.ncbi.nlm.nih.gov/pubmed/?term=Hyder CL%5BAuthor%5D&cauthor=true&cauthor_uid=24610946)^1^, [Lazaro G](http://www.ncbi.nlm.nih.gov/pubmed/?term=Lazaro G%5BAuthor%5D&cauthor=true&cauthor_uid=24610946)^1^, [Pylvänäinen JW](http://www.ncbi.nlm.nih.gov/pubmed/?term=Pylvänäinen JW%5BAuthor%5D&cauthor=true&cauthor_uid=24610946)^1^, [Roberts MW](http://www.ncbi.nlm.nih.gov/pubmed/?term=Roberts MW%5BAuthor%5D&cauthor=true&cauthor_uid=24610946). Nestin regulates prostate cancer cell invasion by influencing the localisation and functions of FAK and integrins. J Cell Sci. 2014 May 15;127(Pt 10):2161-73.
10. [Burger KL](http://www.ncbi.nlm.nih.gov/pubmed/?term=Burger KL%5BAuthor%5D&cauthor=true&cauthor_uid=24174371)^1^, [Learman BS](http://www.ncbi.nlm.nih.gov/pubmed/?term=Learman BS%5BAuthor%5D&cauthor=true&cauthor_uid=24174371), [Boucherle AK](http://www.ncbi.nlm.nih.gov/pubmed/?term=Boucherle AK%5BAuthor%5D&cauthor=true&cauthor_uid=24174371). Src-dependent Tks5 phosphorylation regulates invadopodia-associated invasion in prostate cancer cells. Prostate. 2014 Feb;74(2):134-48.
11. [Zoubeidi A](http://www.ncbi.nlm.nih.gov/pubmed/?term=Zoubeidi A%5BAuthor%5D&cauthor=true&cauthor_uid=20068069)^1^, [Ettinger S](http://www.ncbi.nlm.nih.gov/pubmed/?term=Ettinger S%5BAuthor%5D&cauthor=true&cauthor_uid=20068069), [Beraldi E](http://www.ncbi.nlm.nih.gov/pubmed/?term=Beraldi E%5BAuthor%5D&cauthor=true&cauthor_uid=20068069), [Hadaschik B](http://www.ncbi.nlm.nih.gov/pubmed/?term=Hadaschik B%5BAuthor%5D&cauthor=true&cauthor_uid=20068069). Clusterin facilitates COMMD1 and I-kappaB degradation to enhance NF-kappaB activity in prostate cancer cells. Mol Cancer Res. 2010 Jan;8(1):119-30.
12. [Gu L](http://www.ncbi.nlm.nih.gov/pubmed/?term=Gu L%5BAuthor%5D&cauthor=true&cauthor_uid=20167868)^1^, [Dagvadorj A](http://www.ncbi.nlm.nih.gov/pubmed/?term=Dagvadorj A%5BAuthor%5D&cauthor=true&cauthor_uid=20167868), [Lutz J](http://www.ncbi.nlm.nih.gov/pubmed/?term=Lutz J%5BAuthor%5D&cauthor=true&cauthor_uid=20167868), [Leiby B](http://www.ncbi.nlm.nih.gov/pubmed/?term=Leiby B%5BAuthor%5D&cauthor=true&cauthor_uid=20167868). Transcription factor Stat3 stimulates metastatic behavior of human prostate cancer cells in vivo, whereas Stat5b has a preferential role in the promotion of prostate cancer cell viability and tumor growth. Am J Pathol. 2010 Apr;176(4):1959-72.
13. [Wu D](http://www.ncbi.nlm.nih.gov/pubmed/?term=Wu D%5BAuthor%5D&cauthor=true&cauthor_uid=15467757)^1^, [Thakore CU](http://www.ncbi.nlm.nih.gov/pubmed/?term=Thakore CU%5BAuthor%5D&cauthor=true&cauthor_uid=15467757), [Wescott GG](http://www.ncbi.nlm.nih.gov/pubmed/?term=Wescott GG%5BAuthor%5D&cauthor=true&cauthor_uid=15467757). Integrin signaling links protein kinase Cepsilon to the protein kinase B/Akt survival pathway in recurrent prostate cancer cells. Oncogene. 2004 Nov 11;23(53):8659-72.
14. [Casar B](http://www.ncbi.nlm.nih.gov/pubmed/?term=Casar B%5BAuthor%5D&cauthor=true&cauthor_uid=23208492)^1^, [Rimann I](http://www.ncbi.nlm.nih.gov/pubmed/?term=Rimann I%5BAuthor%5D&cauthor=true&cauthor_uid=23208492)^1^, [Kato H](http://www.ncbi.nlm.nih.gov/pubmed/?term=Kato H%5BAuthor%5D&cauthor=true&cauthor_uid=23208492)^2^, [Shattil SJ](http://www.ncbi.nlm.nih.gov/pubmed/?term=Shattil SJ%5BAuthor%5D&cauthor=true&cauthor_uid=23208492). In vivo cleaved CDCP1 promotes early tumor dissemination via complexing with activated β1 integrin and induction of FAK/PI3K/Akt motility signaling. Oncogene. 2014 Jan 9;33(2):255-68.
15. [Zhang Y](http://www.ncbi.nlm.nih.gov/pubmed/?term=Zhang Y%5BAuthor%5D&cauthor=true&cauthor_uid=22648782)^1^, [Wang Y](http://www.ncbi.nlm.nih.gov/pubmed/?term=Wang Y%5BAuthor%5D&cauthor=true&cauthor_uid=22648782), [Yuan J](http://www.ncbi.nlm.nih.gov/pubmed/?term=Yuan J%5BAuthor%5D&cauthor=true&cauthor_uid=22648782), [Qin W](http://www.ncbi.nlm.nih.gov/pubmed/?term=Qin W%5BAuthor%5D&cauthor=true&cauthor_uid=22648782). Toll-like receptor 4 ligation confers chemoresistance to docetaxel on PC-3 human prostate cancer cells. Cell Biol Toxicol. 2012 Aug;28(4):269-77.
16. [Gan L](http://www.ncbi.nlm.nih.gov/pubmed/?term=Gan L%5BAuthor%5D&cauthor=true&cauthor_uid=21656826)^1^, [Wang J](http://www.ncbi.nlm.nih.gov/pubmed/?term=Wang J%5BAuthor%5D&cauthor=true&cauthor_uid=21656826), [Xu H](http://www.ncbi.nlm.nih.gov/pubmed/?term=Xu H%5BAuthor%5D&cauthor=true&cauthor_uid=21656826). Resistance to docetaxel-induced apoptosis in prostate cancer cells by p38/p53/p21 signaling. Prostate. 2011 Aug 1;71(11):1158-66.
17. [Zhu Y](http://www.ncbi.nlm.nih.gov/pubmed/?term=Zhu Y%5BAuthor%5D&cauthor=true&cauthor_uid=23861346)^1^, [Liu C](http://www.ncbi.nlm.nih.gov/pubmed/?term=Liu C%5BAuthor%5D&cauthor=true&cauthor_uid=23861346), [Nadiminty N](http://www.ncbi.nlm.nih.gov/pubmed/?term=Nadiminty N%5BAuthor%5D&cauthor=true&cauthor_uid=23861346). Inhibition of ABCB1 expression overcomes acquired docetaxel resistance in prostate cancer. Mol Cancer Ther. 2013 Sep;12(9):1829-36.
18. [Lee BY](http://www.ncbi.nlm.nih.gov/pubmed/?term=Lee BY%5BAuthor%5D&cauthor=true&cauthor_uid=24194567), [Hochgräfe F](http://www.ncbi.nlm.nih.gov/pubmed/?term=Hochgräfe F%5BAuthor%5D&cauthor=true&cauthor_uid=24194567), [Lin HM](http://www.ncbi.nlm.nih.gov/pubmed/?term=Lin HM%5BAuthor%5D&cauthor=true&cauthor_uid=24194567). Phosphoproteomic profiling identifies focal adhesion kinase as a mediator of docetaxel resistance in castrate-resistant prostate cancer. Mol Cancer Ther. 2014 Jan;13(1):190-201
19. [Criswell T](http://www.ncbi.nlm.nih.gov/pubmed/?term=Criswell T%5BAuthor%5D&cauthor=true&cauthor_uid=15689620)^1^, [Beman M](http://www.ncbi.nlm.nih.gov/pubmed/?term=Beman M%5BAuthor%5D&cauthor=true&cauthor_uid=15689620), [Araki S](http://www.ncbi.nlm.nih.gov/pubmed/?term=Araki S%5BAuthor%5D&cauthor=true&cauthor_uid=15689620). Delayed activation of insulin-like growth factor-1 receptor/Src/MAPK/Egr-1 signaling regulates clusterin expression, a pro-survival factor. J Biol Chem. 2005 Apr 8;280(14):14212-21.

# [**Shiota M**](http://www.ncbi.nlm.nih.gov/pubmed/?term=Shiota M%5BAuthor%5D&cauthor=true&cauthor_uid=21987172)^1^, [**Zoubeidi A**](http://www.ncbi.nlm.nih.gov/pubmed/?term=Zoubeidi A%5BAuthor%5D&cauthor=true&cauthor_uid=21987172), [**Kumano M**](http://www.ncbi.nlm.nih.gov/pubmed/?term=Kumano M%5BAuthor%5D&cauthor=true&cauthor_uid=21987172). Clusterin is a critical downstream mediator of stress-induced YB-1 transactivation in prostate cancer. Mol Cancer Res. 2011 Dec;9(12):1755-66.

# [**Ni J**](http://www.ncbi.nlm.nih.gov/pubmed/?term=Ni J%5BAuthor%5D&cauthor=true&cauthor_uid=24076216)^1^, [**Cozzi P**](http://www.ncbi.nlm.nih.gov/pubmed/?term=Cozzi P%5BAuthor%5D&cauthor=true&cauthor_uid=24076216), [**Hao J**](http://www.ncbi.nlm.nih.gov/pubmed/?term=Hao J%5BAuthor%5D&cauthor=true&cauthor_uid=24076216), [**Beretov J**](http://www.ncbi.nlm.nih.gov/pubmed/?term=Beretov J%5BAuthor%5D&cauthor=true&cauthor_uid=24076216). Epithelial cell adhesion molecule (EpCAM) is associated with prostate cancer metastasis and chemo/radioresistance via the PI3K/Akt/mTOR signaling pathway. Int J Biochem Cell Biol. 2013 Dec;45(12):2736-48.

1. [Zhong B](http://www.ncbi.nlm.nih.gov/pubmed/?term=Zhong B%5BAuthor%5D&cauthor=true&cauthor_uid=20501799)^1^, [Sallman DA](http://www.ncbi.nlm.nih.gov/pubmed/?term=Sallman DA%5BAuthor%5D&cauthor=true&cauthor_uid=20501799), [Gilvary DL](http://www.ncbi.nlm.nih.gov/pubmed/?term=Gilvary DL%5BAuthor%5D&cauthor=true&cauthor_uid=20501799). Induction of clusterin by AKT--role in cytoprotection against docetaxel in prostate tumor cells. Mol Cancer Ther. 2010 Jun;9(6):1831-41.

# [**Lee JT Jr**](http://www.ncbi.nlm.nih.gov/pubmed/?term=Lee JT Jr%5BAuthor%5D&cauthor=true&cauthor_uid=15548710)^1^, [**Steelman LS**](http://www.ncbi.nlm.nih.gov/pubmed/?term=Steelman LS%5BAuthor%5D&cauthor=true&cauthor_uid=15548710), [**McCubrey JA**](http://www.ncbi.nlm.nih.gov/pubmed/?term=McCubrey JA%5BAuthor%5D&cauthor=true&cauthor_uid=15548710). Phosphatidylinositol 3'-kinase activation leads to multidrug resistance protein-1 expression and subsequent chemoresistance in advanced prostate cancer cells. Cancer Res. 2004 Nov 15;64(22):8397-404.

1. [Wu K](http://www.ncbi.nlm.nih.gov/pubmed/?term=Wu K%5BAuthor%5D&cauthor=true&cauthor_uid=23604126)^1^, [Liu J](http://www.ncbi.nlm.nih.gov/pubmed/?term=Liu J%5BAuthor%5D&cauthor=true&cauthor_uid=23604126)^2^, [Tseng SF](http://www.ncbi.nlm.nih.gov/pubmed/?term=Tseng SF%5BAuthor%5D&cauthor=true&cauthor_uid=23604126)^3^, [Gore C](http://www.ncbi.nlm.nih.gov/pubmed/?term=Gore C%5BAuthor%5D&cauthor=true&cauthor_uid=23604126). The role of DAB2IP in androgen receptor activation during prostate cancer progression. Oncogene. 2014 Apr 10;33(15):1954-63.
2. [Lee KH](http://www.ncbi.nlm.nih.gov/pubmed/?term=Lee KH%5BAuthor%5D&cauthor=true&cauthor_uid=24915000)^1^, [Lin FC](http://www.ncbi.nlm.nih.gov/pubmed/?term=Lin FC%5BAuthor%5D&cauthor=true&cauthor_uid=24915000)^2^, [Hsu TI](http://www.ncbi.nlm.nih.gov/pubmed/?term=Hsu TI%5BAuthor%5D&cauthor=true&cauthor_uid=24915000)^3^, [Lin JT](http://www.ncbi.nlm.nih.gov/pubmed/?term=Lin JT%5BAuthor%5D&cauthor=true&cauthor_uid=24915000). MicroRNA-296-5p (miR-296-5p) functions as a tumor suppressor in prostate cancer by directly targeting Pin1. Biochim Biophys Acta. 2014 Sep;1843(9):2055-66.
3. [Kao CJ](http://www.ncbi.nlm.nih.gov/pubmed/?term=Kao CJ%5BAuthor%5D&cauthor=true&cauthor_uid=23728339)^1^, [Martiniez A](http://www.ncbi.nlm.nih.gov/pubmed/?term=Martiniez A%5BAuthor%5D&cauthor=true&cauthor_uid=23728339)^1^, [Shi XB](http://www.ncbi.nlm.nih.gov/pubmed/?term=Shi XB%5BAuthor%5D&cauthor=true&cauthor_uid=23728339)^2^, [Yang J](http://www.ncbi.nlm.nih.gov/pubmed/?term=Yang J%5BAuthor%5D&cauthor=true&cauthor_uid=23728339). miR-30 as a tumor suppressor connects EGF/Src signal to ERG and EMT. Oncogene. 2014 May 8;33(19):2495-503.

# [**Wang G**](http://www.ncbi.nlm.nih.gov/pubmed/?term=Wang G%5BAuthor%5D&cauthor=true&cauthor_uid=23727861)^1^, [**Lunardi A**](http://www.ncbi.nlm.nih.gov/pubmed/?term=Lunardi A%5BAuthor%5D&cauthor=true&cauthor_uid=23727861), [**Zhang J**](http://www.ncbi.nlm.nih.gov/pubmed/?term=Zhang J%5BAuthor%5D&cauthor=true&cauthor_uid=23727861), [**Chen Z**](http://www.ncbi.nlm.nih.gov/pubmed/?term=Chen Z%5BAuthor%5D&cauthor=true&cauthor_uid=23727861). Zbtb7a suppresses prostate cancer through repression of a Sox9-dependent pathway for cellular senescence bypass and tumor invasion. Nat Genet. 2013 Jul;45(7):739-46.

1. [Burkhardt L](http://www.ncbi.nlm.nih.gov/pubmed/?term=Burkhardt L%5BAuthor%5D&cauthor=true&cauthor_uid=23492366)^1^, [Fuchs S](http://www.ncbi.nlm.nih.gov/pubmed/?term=Fuchs S%5BAuthor%5D&cauthor=true&cauthor_uid=23492366), [Krohn A](http://www.ncbi.nlm.nih.gov/pubmed/?term=Krohn A%5BAuthor%5D&cauthor=true&cauthor_uid=23492366). CHD1 is a 5q21 tumor suppressor required for ERG rearrangement in prostate cancer. Cancer Res. 2013 May 1;73(9):2795-805.
2. [Han YC](http://www.ncbi.nlm.nih.gov/pubmed/?term=Han YC%5BAuthor%5D&cauthor=true&cauthor_uid=23355073)^1^, [Zheng ZL](http://www.ncbi.nlm.nih.gov/pubmed/?term=Zheng ZL%5BAuthor%5D&cauthor=true&cauthor_uid=23355073), [Zuo ZH](http://www.ncbi.nlm.nih.gov/pubmed/?term=Zuo ZH%5BAuthor%5D&cauthor=true&cauthor_uid=23355073). Metallothionein 1 h tumour suppressor activity in prostate cancer is mediated by euchromatin methyltransferase 1. J Pathol. 2013 Jun;230(2):184-93.
3. [Kojima S](http://www.ncbi.nlm.nih.gov/pubmed/?term=Kojima S%5BAuthor%5D&cauthor=true&cauthor_uid=22068816)^1^, [Chiyomaru T](http://www.ncbi.nlm.nih.gov/pubmed/?term=Chiyomaru T%5BAuthor%5D&cauthor=true&cauthor_uid=22068816), [Kawakami K](http://www.ncbi.nlm.nih.gov/pubmed/?term=Kawakami K%5BAuthor%5D&cauthor=true&cauthor_uid=22068816). Tumour suppressors miR-1 and miR-133a target the oncogenic function of purine nucleoside phosphorylase (PNP) in prostate cancer. Br J Cancer. 2012 Jan 17;106(2):405-13.

# [**Vitari AC**](http://www.ncbi.nlm.nih.gov/pubmed/?term=Vitari AC%5BAuthor%5D&cauthor=true&cauthor_uid=21572435)^1^, [**Leong KG**](http://www.ncbi.nlm.nih.gov/pubmed/?term=Leong KG%5BAuthor%5D&cauthor=true&cauthor_uid=21572435), [**Newton K**](http://www.ncbi.nlm.nih.gov/pubmed/?term=Newton K%5BAuthor%5D&cauthor=true&cauthor_uid=21572435). COP1 is a tumour suppressor that causes degradation of ETS transcription factors. Nature. 2011 May 15;474(7351):403-6.

# [**Brett A**](http://www.ncbi.nlm.nih.gov/pubmed/?term=Brett A%5BAuthor%5D&cauthor=true&cauthor_uid=23298185)^1^, [**Pandey S**](http://www.ncbi.nlm.nih.gov/pubmed/?term=Pandey S%5BAuthor%5D&cauthor=true&cauthor_uid=23298185), [**Fraizer G**](http://www.ncbi.nlm.nih.gov/pubmed/?term=Fraizer G%5BAuthor%5D&cauthor=true&cauthor_uid=23298185).The Wilms' tumor gene (WT1) regulates E-cadherin expression and migration of prostate cancer cells. Mol Cancer. 2013 Jan 8;12:3.

1. [Qu F](http://www.ncbi.nlm.nih.gov/pubmed/?term=Qu F%5BAuthor%5D&cauthor=true&cauthor_uid=23417242)^1^, [Cui X](http://www.ncbi.nlm.nih.gov/pubmed/?term=Cui X%5BAuthor%5D&cauthor=true&cauthor_uid=23417242), [Hong Y](http://www.ncbi.nlm.nih.gov/pubmed/?term=Hong Y%5BAuthor%5D&cauthor=true&cauthor_uid=23417242), [Wang J](http://www.ncbi.nlm.nih.gov/pubmed/?term=Wang J%5BAuthor%5D&cauthor=true&cauthor_uid=23417242). MicroRNA-185 suppresses proliferation, invasion, migration, and tumorigenicity of human prostate cancer cells through targeting androgen receptor. Mol Cell Biochem. 2013 May;377(1-2):121-30.

# [**Hagman Z**](http://www.ncbi.nlm.nih.gov/pubmed/?term=Hagman Z%5BAuthor%5D&cauthor=true&cauthor_uid=23922103)^1^, [**Haflidadottir BS**](http://www.ncbi.nlm.nih.gov/pubmed/?term=Haflidadottir BS%5BAuthor%5D&cauthor=true&cauthor_uid=23922103), [**Ansari M**](http://www.ncbi.nlm.nih.gov/pubmed/?term=Ansari M%5BAuthor%5D&cauthor=true&cauthor_uid=23922103). The tumour suppressor miR-34c targets MET in prostate cancer cells. Br J Cancer. 2013 Sep 3;109(5):1271-8.

1. [Shaikhibrahim Z](http://www.ncbi.nlm.nih.gov/pubmed/?term=Shaikhibrahim Z%5BAuthor%5D&cauthor=true&cauthor_uid=24938407)^1^, [Offermann A](http://www.ncbi.nlm.nih.gov/pubmed/?term=Offermann A%5BAuthor%5D&cauthor=true&cauthor_uid=24938407)^1^, [Braun M](http://www.ncbi.nlm.nih.gov/pubmed/?term=Braun M%5BAuthor%5D&cauthor=true&cauthor_uid=24938407). MED12 overexpression is a frequent event in castration-resistant prostate cancer. Endocr Relat Cancer. 2014 Aug;21(4):663-75.
2. [Lin P](http://www.ncbi.nlm.nih.gov/pubmed/?term=Lin P%5BAuthor%5D&cauthor=true&cauthor_uid=21837402), [Sun X](http://www.ncbi.nlm.nih.gov/pubmed/?term=Sun X%5BAuthor%5D&cauthor=true&cauthor_uid=21837402), [Feng T](http://www.ncbi.nlm.nih.gov/pubmed/?term=Feng T%5BAuthor%5D&cauthor=true&cauthor_uid=21837402). ADAM17 regulates prostate cancer cell proliferation through mediating cell cycle progression by EGFR/PI3K/AKT pathway. Mol Cell Biochem. 2012 Jan;359(1-2):235-43.
3. [Vander Griend DJ](http://www.ncbi.nlm.nih.gov/pubmed/?term=Vander Griend DJ%5BAuthor%5D&cauthor=true&cauthor_uid=24948876)^1^, [Litvinov IV](http://www.ncbi.nlm.nih.gov/pubmed/?term=Litvinov IV%5BAuthor%5D&cauthor=true&cauthor_uid=24948876)^2^, [Isaacs JT](http://www.ncbi.nlm.nih.gov/pubmed/?term=Isaacs JT%5BAuthor%5D&cauthor=true&cauthor_uid=24948876).Conversion of androgen receptor signaling from a growth suppressor in normal prostate epithelial cells to an oncogene in prostate cancer cells involves a gain of function in c-Myc regulation. Int J Biol Sci. 2014 Jun 10;10(6):627-42.
4. [Lin PC](http://www.ncbi.nlm.nih.gov/pubmed/?term=Lin PC%5BAuthor%5D&cauthor=true&cauthor_uid=23233736), [Chiu YL](http://www.ncbi.nlm.nih.gov/pubmed/?term=Chiu YL%5BAuthor%5D&cauthor=true&cauthor_uid=23233736), [Banerjee S](http://www.ncbi.nlm.nih.gov/pubmed/?term=Banerjee S%5BAuthor%5D&cauthor=true&cauthor_uid=23233736).Epigenetic repression of miR-31 disrupts androgen receptor homeostasis and contributes to prostate cancer progression. Cancer Res. 2013 Feb 1;73(3):1232-44.
5. [Shi Y](http://www.ncbi.nlm.nih.gov/pubmed/?term=Shi Y%5BAuthor%5D&cauthor=true&cauthor_uid=23250485), [Han JJ](http://www.ncbi.nlm.nih.gov/pubmed/?term=Han JJ%5BAuthor%5D&cauthor=true&cauthor_uid=23250485), [Tennakoon JB](http://www.ncbi.nlm.nih.gov/pubmed/?term=Tennakoon JB%5BAuthor%5D&cauthor=true&cauthor_uid=23250485). Androgens promote prostate cancer cell growth through induction of autophagy. Mol Endocrinol. 2013 Feb;27(2):280-95.
6. [Nadiminty N](http://www.ncbi.nlm.nih.gov/pubmed/?term=Nadiminty N%5BAuthor%5D&cauthor=true&cauthor_uid=23699654), [Tummala R](http://www.ncbi.nlm.nih.gov/pubmed/?term=Tummala R%5BAuthor%5D&cauthor=true&cauthor_uid=23699654), [Liu](http://www.ncbi.nlm.nih.gov/pubmed/?term=Liu C%5BAuthor%5D&cauthor=true&cauthor_uid=23699654). NF-κB2/p52 induces resistance to enzalutamide in prostate cancer: role of androgen receptor and its variants. Mol Cancer Ther. 2013 Aug;12(8):1629-37.
7. [Jiang WG](http://www.ncbi.nlm.nih.gov/pubmed/?term=Jiang WG%5BAuthor%5D&cauthor=true&cauthor_uid=24161123), [Ye L](http://www.ncbi.nlm.nih.gov/pubmed/?term=Ye L%5BAuthor%5D&cauthor=true&cauthor_uid=24161123), [Sanders AJ](http://www.ncbi.nlm.nih.gov/pubmed/?term=Sanders AJ%5BAuthor%5D&cauthor=true&cauthor_uid=24161123).Prostate transglutaminase (TGase-4, TGaseP) enhances the adhesion of prostate cancer cells to extracellular matrix, the potential role of TGase-core domain. J Transl Med. 2013 Oct 25;11:269.

# [**Zhang XA**](http://www.ncbi.nlm.nih.gov/pubmed/?term=Zhang XA%5BAuthor%5D&cauthor=true&cauthor_uid=12738793), [**He B**](http://www.ncbi.nlm.nih.gov/pubmed/?term=He B%5BAuthor%5D&cauthor=true&cauthor_uid=12738793), [**Zhou B**](http://www.ncbi.nlm.nih.gov/pubmed/?term=Zhou B%5BAuthor%5D&cauthor=true&cauthor_uid=12738793), [**Liu L**](http://www.ncbi.nlm.nih.gov/pubmed/?term=Liu L%5BAuthor%5D&cauthor=true&cauthor_uid=12738793).Requirement of the p130CAS-Crk coupling for metastasis suppressor KAI1/CD82-mediated inhibition of cell migration. **J Biol Chem**. **2003**Jul 18;**278**(**29**):**27319-28.**

# [**Chen X**](http://www.ncbi.nlm.nih.gov/pubmed/?term=Chen X%5BAuthor%5D&cauthor=true&cauthor_uid=24632071), [**Corbin JM**](http://www.ncbi.nlm.nih.gov/pubmed/?term=Corbin JM%5BAuthor%5D&cauthor=true&cauthor_uid=24632071)^2^, [**Tipton GJ**](http://www.ncbi.nlm.nih.gov/pubmed/?term=Tipton GJ%5BAuthor%5D&cauthor=true&cauthor_uid=24632071). The TMEFF2 tumor suppressor modulates integrin expression, RhoA activation and migration of prostate cancer cells. Biochem Biophys Res Commun. 2006 May 12;343(3):848-56.

# [**Valderrama F**](http://www.ncbi.nlm.nih.gov/pubmed/?term=Valderrama F%5BAuthor%5D&cauthor=true&cauthor_uid=22467863), [**Thevapala S**](http://www.ncbi.nlm.nih.gov/pubmed/?term=Thevapala S%5BAuthor%5D&cauthor=true&cauthor_uid=22467863), [**Ridley AJ**](http://www.ncbi.nlm.nih.gov/pubmed/?term=Ridley AJ%5BAuthor%5D&cauthor=true&cauthor_uid=22467863). Radixin regulates cell migration and cell-cell adhesion through Rac1. J Cell Sci. 2012 Jul 15;125(Pt 14):3310-9.

1. [Gururajan M](http://www.ncbi.nlm.nih.gov/pubmed/?term=Gururajan M%5BAuthor%5D&cauthor=true&cauthor_uid=26624980), [Cavassani KA](http://www.ncbi.nlm.nih.gov/pubmed/?term=Cavassani KA%5BAuthor%5D&cauthor=true&cauthor_uid=26624980), [Sievert M](http://www.ncbi.nlm.nih.gov/pubmed/?term=Sievert M%5BAuthor%5D&cauthor=true&cauthor_uid=26624980). SRC family kinase FYN promotes the neuroendocrine phenotype and visceral metastasis in advanced prostate cancer. Oncotarget. 2015 Dec 29;6(42):44072-83.
2. [Moritz T](http://www.ncbi.nlm.nih.gov/pubmed/?term=Moritz T%5BAuthor%5D&cauthor=true&cauthor_uid=26846108), [Venz S](http://www.ncbi.nlm.nih.gov/pubmed/?term=Venz S%5BAuthor%5D&cauthor=true&cauthor_uid=26846108), [Junker H](http://www.ncbi.nlm.nih.gov/pubmed/?term=Junker H%5BAuthor%5D&cauthor=true&cauthor_uid=26846108), [Kreuz S](http://www.ncbi.nlm.nih.gov/pubmed/?term=Kreuz S%5BAuthor%5D&cauthor=true&cauthor_uid=26846108). Isoform 1 of TPD52 (PC-1) promotes neuroendocrine transdifferentiation in prostate cancer cells. Tumour Biol. 2016 Feb 5.
3. [Dang Q](http://www.ncbi.nlm.nih.gov/pubmed/?term=Dang Q%5BAuthor%5D&cauthor=true&cauthor_uid=25817444), [Li L](http://www.ncbi.nlm.nih.gov/pubmed/?term=Li L%5BAuthor%5D&cauthor=true&cauthor_uid=25817444), [Xie H](http://www.ncbi.nlm.nih.gov/pubmed/?term=Xie H%5BAuthor%5D&cauthor=true&cauthor_uid=25817444), [He D](http://www.ncbi.nlm.nih.gov/pubmed/?term=He D%5BAuthor%5D&cauthor=true&cauthor_uid=25817444)^3^, [Chen J](http://www.ncbi.nlm.nih.gov/pubmed/?term=Chen J%5BAuthor%5D&cauthor=true&cauthor_uid=25817444). Anti-androgen enzalutamide enhances prostate cancer neuroendocrine (NE) differentiation via altering the infiltrated mast cells → androgen receptor (AR) → miRNA32 signals. Mol Oncol. 2015 Aug;9(7):1241-51.
4. [Gong Y](http://www.ncbi.nlm.nih.gov/pubmed/?term=Gong Y%5BAuthor%5D&cauthor=true&cauthor_uid=25560638), [Chippada-Venkata UD](http://www.ncbi.nlm.nih.gov/pubmed/?term=Chippada-Venkata UD%5BAuthor%5D&cauthor=true&cauthor_uid=25560638), [Galsky MD](http://www.ncbi.nlm.nih.gov/pubmed/?term=Galsky MD%5BAuthor%5D&cauthor=true&cauthor_uid=25560638), [Huang J](http://www.ncbi.nlm.nih.gov/pubmed/?term=Huang J%5BAuthor%5D&cauthor=true&cauthor_uid=25560638). Elevated circulating tissue inhibitor of metalloproteinase 1 (TIMP-1) levels are associated with neuroendocrine differentiation in castration resistantprostate cancer. Prostate. 2015 May;75(6):616-27.
5. [Li Q](http://www.ncbi.nlm.nih.gov/pubmed/?term=Li Q%5BAuthor%5D&cauthor=true&cauthor_uid=27041934), [Zhang CS](http://www.ncbi.nlm.nih.gov/pubmed/?term=Zhang CS%5BAuthor%5D&cauthor=true&cauthor_uid=27041934), [Zhang Y](http://www.ncbi.nlm.nih.gov/pubmed/?term=Zhang Y%5BAuthor%5D&cauthor=true&cauthor_uid=27041934). Molecular aspects of prostate cancer with neuroendocrine differentiation. Chin J Cancer Res. 2016 Feb;28(1):122-9.
6. [Ojo D](http://www.ncbi.nlm.nih.gov/pubmed/?term=Ojo D%5BAuthor%5D&cauthor=true&cauthor_uid=26593949), [Lin X](http://www.ncbi.nlm.nih.gov/pubmed/?term=Lin X%5BAuthor%5D&cauthor=true&cauthor_uid=26593949)^4^, [Wong N](http://www.ncbi.nlm.nih.gov/pubmed/?term=Wong N%5BAuthor%5D&cauthor=true&cauthor_uid=26593949)^7^, [Gu Y](http://www.ncbi.nlm.nih.gov/pubmed/?term=Gu Y%5BAuthor%5D&cauthor=true&cauthor_uid=26593949). Prostate Cancer Stem-like Cells Contribute to the Development of Castration-Resistant Prostate Cancer. Cancers (Basel). 2015 Nov 18;7(4):2290-308.
7. [Wang N](http://www.ncbi.nlm.nih.gov/pubmed/?term=Wang N%5BAuthor%5D&cauthor=true&cauthor_uid=26071486), [Yao M](http://www.ncbi.nlm.nih.gov/pubmed/?term=Yao M%5BAuthor%5D&cauthor=true&cauthor_uid=26071486), [Xu J](http://www.ncbi.nlm.nih.gov/pubmed/?term=Xu J%5BAuthor%5D&cauthor=true&cauthor_uid=26071486), [Quan Y](http://www.ncbi.nlm.nih.gov/pubmed/?term=Quan Y%5BAuthor%5D&cauthor=true&cauthor_uid=26071486), [Zhang K](http://www.ncbi.nlm.nih.gov/pubmed/?term=Zhang K%5BAuthor%5D&cauthor=true&cauthor_uid=26071486). Autocrine Activation of CHRM3 Promotes Prostate Cancer Growth and Castration Resistance via CaM/CaMKK-Mediated Phosphorylation of Akt. Clin Cancer Res. 2015 Oct 15;21(20):4676-85.
8. [Wang J](http://www.ncbi.nlm.nih.gov/pubmed/?term=Wang J%5BAuthor%5D&cauthor=true&cauthor_uid=27019329), [Zou JX](http://www.ncbi.nlm.nih.gov/pubmed/?term=Zou JX%5BAuthor%5D&cauthor=true&cauthor_uid=27019329), [Xue X](http://www.ncbi.nlm.nih.gov/pubmed/?term=Xue X%5BAuthor%5D&cauthor=true&cauthor_uid=27019329), [Cai D](http://www.ncbi.nlm.nih.gov/pubmed/?term=Cai D%5BAuthor%5D&cauthor=true&cauthor_uid=27019329), [Zhang Y](http://www.ncbi.nlm.nih.gov/pubmed/?term=Zhang Y%5BAuthor%5D&cauthor=true&cauthor_uid=27019329). ROR-γ drives androgen receptor expression and represents a therapeutic target in castration-resistant prostate cancer. Nat Med. 2016 May;22(5):488-96.
9. [Wu X](http://www.ncbi.nlm.nih.gov/pubmed/?term=Wu X%5BAuthor%5D&cauthor=true&cauthor_uid=26636648), [Deng F](http://www.ncbi.nlm.nih.gov/pubmed/?term=Deng F%5BAuthor%5D&cauthor=true&cauthor_uid=26636648), [Li Y](http://www.ncbi.nlm.nih.gov/pubmed/?term=Li Y%5BAuthor%5D&cauthor=true&cauthor_uid=26636648), [Daniels G](http://www.ncbi.nlm.nih.gov/pubmed/?term=Daniels G%5BAuthor%5D&cauthor=true&cauthor_uid=26636648). ACSL4 promotes prostate cancer growth, invasion and hormonal resistance. Oncotarget. 2015 Dec 29;6(42):44849-63.
10. [Zhang L](http://www.ncbi.nlm.nih.gov/pubmed/?term=Zhang L%5BAuthor%5D&cauthor=true&cauthor_uid=25645929), [Yang S](http://www.ncbi.nlm.nih.gov/pubmed/?term=Yang S%5BAuthor%5D&cauthor=true&cauthor_uid=25645929), [Chen X](http://www.ncbi.nlm.nih.gov/pubmed/?term=Chen X%5BAuthor%5D&cauthor=true&cauthor_uid=25645929), [Stauffer S](http://www.ncbi.nlm.nih.gov/pubmed/?term=Stauffer S%5BAuthor%5D&cauthor=true&cauthor_uid=25645929). The hippo pathway effector YAP regulates motility, invasion, and castration-resistant growth of prostate cancer cells. Mol Cell Biol. 2015 Apr;35(8):1350-62.
11. [Komura K](http://www.ncbi.nlm.nih.gov/pubmed/?term=Komura K%5BAuthor%5D&cauthor=true&cauthor_uid=27185910), [Jeong SH](http://www.ncbi.nlm.nih.gov/pubmed/?term=Jeong SH%5BAuthor%5D&cauthor=true&cauthor_uid=27185910), [Hinohara K](http://www.ncbi.nlm.nih.gov/pubmed/?term=Hinohara K%5BAuthor%5D&cauthor=true&cauthor_uid=27185910), [Qu F](http://www.ncbi.nlm.nih.gov/pubmed/?term=Qu F%5BAuthor%5D&cauthor=true&cauthor_uid=27185910), [Wang X](http://www.ncbi.nlm.nih.gov/pubmed/?term=Wang X%5BAuthor%5D&cauthor=true&cauthor_uid=27185910). Resistance to docetaxel in prostate cancer is associated with androgen receptor activation and loss of KDM5D expression. Proc Natl Acad Sci U S A. 2016 May 31;113(22):6259-64.
12. [Shiota M](http://www.ncbi.nlm.nih.gov/pubmed/?term=Shiota M%5BAuthor%5D&cauthor=true&cauthor_uid=26857528), [Fujimoto N](http://www.ncbi.nlm.nih.gov/pubmed/?term=Fujimoto N%5BAuthor%5D&cauthor=true&cauthor_uid=26857528), [Imada K](http://www.ncbi.nlm.nih.gov/pubmed/?term=Imada K%5BAuthor%5D&cauthor=true&cauthor_uid=26857528), [Yokomizo A](http://www.ncbi.nlm.nih.gov/pubmed/?term=Yokomizo A%5BAuthor%5D&cauthor=true&cauthor_uid=26857528). Potential Role for YB-1 in Castration-Resistant Prostate Cancer and Resistance to Enzalutamide Through the Androgen Receptor V7. J Natl Cancer Inst. 2016 Feb 8;108(7).
13. [Ding G](http://www.ncbi.nlm.nih.gov/pubmed/?term=Ding G%5BAuthor%5D&cauthor=true&cauthor_uid=25728945), [Fang J](http://www.ncbi.nlm.nih.gov/pubmed/?term=Fang J%5BAuthor%5D&cauthor=true&cauthor_uid=25728945), [Tong S](http://www.ncbi.nlm.nih.gov/pubmed/?term=Tong S%5BAuthor%5D&cauthor=true&cauthor_uid=25728945), [Qu L](http://www.ncbi.nlm.nih.gov/pubmed/?term=Qu L%5BAuthor%5D&cauthor=true&cauthor_uid=25728945).Over-expression of lipocalin 2 promotes cell migration and invasion through activating ERK signaling to increase SLUG expression in prostate cancer. Prostate. 2015 Jun 15;75(9):957-68.
14. [Byrne NM](http://www.ncbi.nlm.nih.gov/pubmed/?term=Byrne NM%5BAuthor%5D&cauthor=true&cauthor_uid=26954717), [Nesbitt H](http://www.ncbi.nlm.nih.gov/pubmed/?term=Nesbitt H%5BAuthor%5D&cauthor=true&cauthor_uid=26954717), [Ming L](http://www.ncbi.nlm.nih.gov/pubmed/?term=Ming L%5BAuthor%5D&cauthor=true&cauthor_uid=26954717), [McKeown SR](http://www.ncbi.nlm.nih.gov/pubmed/?term=McKeown SR%5BAuthor%5D&cauthor=true&cauthor_uid=26954717). Androgen deprivation in LNCaP prostate tumour xenografts induces vascular changes and hypoxic stress, resulting in promotion of epithelial-to-mesenchymal transition. Br J Cancer. 2016 Mar 15;114(6):659-68.
15. [Buczek ME](http://www.ncbi.nlm.nih.gov/pubmed/?term=Buczek ME%5BAuthor%5D&cauthor=true&cauthor_uid=26549027), [Miles AK](http://www.ncbi.nlm.nih.gov/pubmed/?term=Miles AK%5BAuthor%5D&cauthor=true&cauthor_uid=26549027), [Green W](http://www.ncbi.nlm.nih.gov/pubmed/?term=Green W%5BAuthor%5D&cauthor=true&cauthor_uid=26549027), [Johnson C](http://www.ncbi.nlm.nih.gov/pubmed/?term=Johnson C%5BAuthor%5D&cauthor=true&cauthor_uid=26549027). Cytoplasmic PML promotes TGF-β-associated epithelial-mesenchymal transition and invasion in prostate cancer. Oncogene. 2015 Nov 9.
16. [Russo MV](http://www.ncbi.nlm.nih.gov/pubmed/?term=Russo MV%5BAuthor%5D&cauthor=true&cauthor_uid=26540632), [Esposito S](http://www.ncbi.nlm.nih.gov/pubmed/?term=Esposito S%5BAuthor%5D&cauthor=true&cauthor_uid=26540632), [Tupone MG](http://www.ncbi.nlm.nih.gov/pubmed/?term=Tupone MG%5BAuthor%5D&cauthor=true&cauthor_uid=26540632), [Manzoli L](http://www.ncbi.nlm.nih.gov/pubmed/?term=Manzoli L%5BAuthor%5D&cauthor=true&cauthor_uid=26540632). SOX2 boosts major tumor progression genes in prostate cancer and is a functional biomarker of lymph node metastasis. Oncotarget. 2016 Mar 15;7(11):12372-85.
17. [Zhang K](http://www.ncbi.nlm.nih.gov/pubmed/?term=Zhang K%5BAuthor%5D&cauthor=true&cauthor_uid=26050620), [Zhao H](http://www.ncbi.nlm.nih.gov/pubmed/?term=Zhao H%5BAuthor%5D&cauthor=true&cauthor_uid=26050620), [Ji Z](http://www.ncbi.nlm.nih.gov/pubmed/?term=Ji Z%5BAuthor%5D&cauthor=true&cauthor_uid=26050620), [Zhang C](http://www.ncbi.nlm.nih.gov/pubmed/?term=Zhang C%5BAuthor%5D&cauthor=true&cauthor_uid=26050620). Shp2 promotes metastasis of prostate cancer by attenuating the PAR3/PAR6/aPKC polarity protein complex and enhancing epithelial-to-mesenchymal transition. Oncogene. 2016 Mar 10;35(10):1271-82.
18. [Baylot V](http://www.ncbi.nlm.nih.gov/pubmed/?term=Baylot V%5BAuthor%5D&cauthor=true&cauthor_uid=22893039), [Katsogiannou M](http://www.ncbi.nlm.nih.gov/pubmed/?term=Katsogiannou M%5BAuthor%5D&cauthor=true&cauthor_uid=22893039), [Andrieu C](http://www.ncbi.nlm.nih.gov/pubmed/?term=Andrieu C%5BAuthor%5D&cauthor=true&cauthor_uid=22893039), [Taieb D](http://www.ncbi.nlm.nih.gov/pubmed/?term=Taieb D%5BAuthor%5D&cauthor=true&cauthor_uid=22893039). Targeting TCTP as a new therapeutic strategy in castration-resistant prostate cancer. Mol Ther. 2012 Dec;20(12):2244-56.

| Total Proteins in Human Prostate Cancer Hallmarks Map (HPCHM) |
| --- |
| IL6 |
| INS |
| STIM1 |
| AR |
| MCP1 |
| FABP4 |
| ADIPOQ |
| PKM2 |
| CXCL13 |
| PIM1 |
| RUNX1 |
| TGFB1 |
| IGFBP3 |
| ANXA1 |
| TIMP1 |
| GDF15 |
| COX2 |
| DNMT1 |
| CAIX |
| PAR1 |
| ARG2 |
| HIC5 |
| TRPM8 |
| ESRRA |
| PINX1 |
| TERT |
| HIF1A |
| ESR2 |
| eNOS |
| HIF2A |
| BMI1 |
| HSP90 |
| P23 |
| CDKN1C |
| AKT1 |
| PRKCA |
| MYC |
| ATM |
| IGF1R |
| PARM1 |
| IGFBP2 |
| SSB |
| TLX |
| ID4 |
| JUNB |
| AGR2 |
| LGALS3BP |
| NKG2D |
| HLA1 |
| STAT3 |
| IL1A |
| IL18BP |
| B7H1 |
| ARG1 |
| NOS2 |
| LMP2 |
| LMP7 |
| TAP1 |
| TAP2 |
| IRF8 |
| B7H3 |
| TNC |
| DIAPH3 |
| SKP2 |
| FUS |
| SOX2 |
| UBE2C |
| MED12 |
| PXN |
| FGFR1 |
| LAT1 |
| LAT3 |
| FADD |
| ADAM17 |
| HOXB13 |
| CDK1 |
| ADAM9 |
| CDK6 |
| BCL2L1 |
| BIRC7 |
| DVL2 |
| ARA70 |
| NGFR |
| IQGAP2 |
| NKX3-1 |
| ALDH1A2 |
| FOXO1 |
| TGFBR3 |
| SERPINB5 |
| TSC1 |
| CHD1 |
| CADM2 |
| MT1H |
| RFWD2 |
| PAX6 |
| SLC2A1 |
| KLF5 |
| TRPM7 |
| KLHL20 |
| ELOVL7 |
| HK2 |
| SPHK1 |
| ABCA1 |
| SLC16A4 |
| NR1H3 |
| ACO2 |
| FASN |
| CTNND2 |
| TIMP3 |
| EGR3 |
| PML |
| JAK2 |
| TWIST1 |
| POSTN |
| CFD |
| PAK1 |
| PARP1 |
| TP53 |
| RB1 |
| 15-LOX2 |
| INSR |
| FZD4 |
| CTNNB1 |
| PIK3R1 |
| SIAH2 |
| BMX |
| FOXA1 |
| FHL2 |
| NCOA3 |
| STYK1 |
| AKR1C3 |
| HES6 |
| YB1 |
| NR5A1 |
| RLN2 |
| ACK1 |
| SREBP1 |
| WNT7B |
| ANG |
| MID1 |
| CXCR4 |
| FER |
| HDAC6 |
| PGC1A |
| VAV3 |
| TACC2 |
| MET |
| PKIB |
| ARR2 |
| LYN |
| CAMK2N1 |
| BIRC6 |
| EGFR |
| CAMKK2 |
| PDE4B |
| PLK1 |
| RSK1 |
| ROCK1 |
| PRL1 |
| YAP1 |
| PTP1B |
| YWHAZ |
| ZEB1 |
| DAB2IP |
| CDK5 |
| ELK1 |
| PYCR1 |
| ERBB2 |
| SMAD4 |
| FOXO3 |
| HEC1 |
| PSGR |
| PIN1 |
| CX3CL1 |
| ACSL4 |
| CHEK2 |
| TOP2A |
| MAPK1 |
| BTK |
| GSTP1 |
| JMJD1A |
| CUL1 |
| DAXX |
| HAMP |
| FGF19 |
| TLR4 |
| EFEMP1 |
| MCL1 |
| RUNX2 |
| PIAS1 |
| ADAM10 |
| BLT2 |
| PRDX3 |
| PIWIL2 |
| ZAP70 |
| CDH11 |
| FRA-1 |
| TCTN1 |
| SNAIL |
| PTTG1 |
| EPLIN |
| UBE2T |
| EIF3D |
| TLN1 |
| LCN2 |
| AQP3 |
| P2X7 |
| IL17 |
| RAP1A |
| CXCL12 |
| ESL1 |
| SRPK1 |
| FRS2 |
| PIM3 |
| EPHA6 |
| CACNA2D2 |
| SPAG9 |
| KLK2 |
| EZH2 |
| ADRB2 |
| LSD1 |
| CSL |
| CAV1 |
| IQGAP1 |
| ANGPTL2 |
| LPAR6 |
| PAX2 |
| AKT2 |
| BCL2 |
| TIP60 |
| CX3CR1 |
| ERBB3 |
| MUC1 |
| HMGA1 |
| FN14 |
| RAC1 |
| ITGB1 |
| EGF |
| EPHA1 |
| EPHA2 |
| FAK |
| P35 |
| WNT5A |
| IL6 |
| IL6ST |
| LIMK |
| ITGB3 |
| MIC1 |
| RHOA |
| SRC |
| FYN |
| MAPK3 |
| TGFBR2 |
| MAP2K1 |
| ID1 |
| ENG |
| ALK2 |
| HGF |
| EC |
| CB1 |
| BAG3 |
| CDCP1 |
| CDC42 |
| VNR |
| ACK |
| LEP |
| LEPR |
| IRS1 |
| IGF1 |
| BDKRB2 |
| KISS1 |
| WASF3 |
| SP1 |
| BCAR1 |
| CRK |
| SMAD3 |
| PKD1 |
| CDH1 |
| CAMK2 |
| CFL1 |
| PRKCD |
| AP1 |
| HSP27 |
| SMAD1 |
| NFKB3 |
| ITGB4 |
| CXCR5 |
| SDF1 |
| CK1 |
| RHOC |
| PTK2B |
| RAS1 |
| RAF1 |
| CGB |
| ITGB5 |
| S1PR |
| GNB2L1 |
| VNRA |
| ITGA2 |
| NES |
| DOCK2 |
| JNK |
| CD168 |
| MTOR |
| EP4 |
| TNFA |
| FRS3 |
| NTRK1 |
| WASP |
| SMAD2 |
| ERK5 |
| TKS5 |
| PAK6 |
| EIF4E |
| ST14 |
| BMPR2 |
| TGFBR1 |
| TMEFF2 |
| SDC3 |
| TM4SF1 |
| TROP2 |
| CXCL2 |
| CCR2 |
| PTK6 |
| KLK1 |
| TGFB3 |
| UPA |
| UPAR |
| ARFGAP3 |
| S1PR1 |
| LPXN |
| GSK3B |
| APC |
| PDE4D |
| WT1 |
| WASF1 |
| RANKL |
| PAK4 |
| PTPN12 |
| SMAD7 |
| SRF |
| TGM4 |
| GEF3 |
| GEFH1 |
| MAPK14 |
| SHC1 |
| LIVI |
| SPINK1 |
| CR1 |
| SNAI1 |
| PAR6 |
| CXCL5 |
| CXCR2 |
| FGF2 |
| NEDD9 |
| PAR4 |
| PT |
| EPCAM |
| MDH2 |
| AMPK |
| TRAP6 |
| MAPK2 |
| P53 |
| PDGF |
| PDGFR |
| CLIP170 |
| STAT1 |
| P21 |
| RIN1 |
| RHEB |
| 4EBP1 |
| IGF2 |
| BAX |
| CDK2 |
| RAS1 |
| CXCL5 |
| PRKCE |
| PKD3 |
| PRL |
| PRLR |
| EPO |
| EPOR |
| E2F1 |
| STAT5B |
| PDGFRB |
| P70S6K |
| GLUT1 |
| HK1 |
| INSR |
| GLUT3 |
| GLS1 |
| CASP3 |
| CCND1 |
| PHB |
| DAB2 |
| GHR |
| KRAS |
| VEGF |
| VEGFR2 |
| CTBP2 |
| FGF |
| NGF |
| TRKA |
| KIF3A |
| HSP90 |
| PDK1 |
| PRKCZ |
| E2F2 |
| SOCS3 |
| MED1 |
| SGK1 |
| EEF1A1 |
| CXCL13 |
| LPAR1 |
| PCA1 |
| DDR1 |
| MAP3K1 |
| JNKK |
| PDGFB |
| ABL |
| p68 |
| CD44 |
| CLU |
| COMMD1 |
| PIK3CB |
| PDGFRA |
| BAK1 |
| ATF2 |
| BAD |
| STAT5 |
| TSC2 |
| CFLAR |
| ROBO1 |
| FSCN1 |
| (RPTP)β/ζ |
| ZEB2 |
| SLUG |
| PDGFD |
| CCND2 |
| HDAC1 |
| P27 |
| RB |
| EGR1 |
| ETV1 |
| ETV4 |
| ETV5 |
| PNP |
| COP1 |
| PIP3 |
| PIP2 |
| PTEN |
| PI3K |
| cPAcP |
| HER2 |
| MEK1 |
| MEK2 |
| cMET |
| MIZ1 |
| ARA70A |
| GF |
| LRF |
| SOX9 |
| BTG2 |
| PIK3IP1 |
| MARCKS |
| BIN1 |
| BECN1 |
| p75(NTR) |
| CCNB |
| CDC25B |
| CDC20 |
| FGF1 |
| CREB |
| MYBL2 |
| p27Kip1 |
| CCNA |
| P21Cip1 |
| CCNE |
| BCLXL |
| REG4 |
| IL6 R |
| ERK1 |
| ERK2 |
| CDK4 |
| p107 |
| DP1 |
| CREB1 |
| TMPRSS2 |
| BRCA2 |
| ATF4 |
| HO1 |
| PGE2 |
| RAP1 |
| CYBA |
| CYBB |
| NCF2 |
| NCF1 |
| P2Y2 |
| PIM2 |
| NOTCH1 |
| TFDP3 |
| p202 |
| TRADD |
| TNFR |
| TRAF2 |
| P14ARF |
| MDM2 |
| PUMA |
| APAF1 |
| CASP9 |
| FASL |
| FAS |
| CASP8 |
| CASP7 |
| ILK |
| TGFA |
| BRCA1 |
| HTRA2 |
| BCL2L2 |
| EIF4B |
| FOXM1 |
| TRAIL |
| SOCS1 |
| HRK |
| FKHR |
| CCNA2 |
| PCA |
| BID |
| XIAP |
| CIAP1 |
| CFLIP |
| SPRED2 |
| cSRC |
| ROCK |
| PDCD4 |
| MTSS1 |
| NDN |
| SCFbeta-TRCP |
| COL1 |
| FOXO3a |
| CD133 |
| OCT4 |
| KLF4 |
| WOX1 |
| LRP6 |
| MEKK |
| cJUN |
| cFOS |
| MMP2 |
| MMP9 |
| WNT3A |
| PCA3 |
| PSA |
| TRPV6 |
| SRC1 |
| TCF/LFF |
| HEF1 |
| IL6 |
| IL8 |
| OPN |
| OBR1 |
| p65 |
| ICAM-1 |
| TCF/LEF |
| HDL |
| ADT |
| SNAI2 |
| DDR2 |
| PTHrP |
| MKK4 |
| BMP2 |
| CD166 |
| TGFB |
| WISP1 |
| PDGFA |
| CXCL16 |
| CXCR6 |
| RAS |
| IL6 ST |
| EP2 |
| IFNG |
| IL1B |
| TNFR1 |
| RIP1 |
| p50 |
| CASP1 |
| ASC |
| ETK |
| S1P |
| RAF |
| p300 |
| SRD5A1 |
| cABL |
| PDGF-B |
| NFKB |
| ID-1 |
| LPA |
| p38 |
| PKA |
| SIRT1 |
| aPKC |
| FKBP51 |
| cMYC |
| BCL6 |
| CEBPA |
| TRMT12 |
| SMYD3 |
| ICAM1 |
| KU80 |
| HSPA4 |
| MEP50 |
| SENP1 |
| ANT1 |
| PIAS3 |
| CDC37 |
| DNAJB1 |
| APPL |
| PELP1 |
| RANBPM |
| EBP1 |
| TCF4 |
| RBAK |
| PYDC1 |
| PNRC |
| HBX |
| AES |
| ZAC1 |
| PATZ |
| ART27 |
| TIP110 |
| ANPK |
| ARR19 |
| KPM |
| ERK8 |
| SCP2 |
| COCOA |
| PARK7 |
| DJBP |
| PP2A |
| MAK |
| RSK |
| DYRK1A |
| TZF |
| ARA160 |
| TGIF |
| RIP140 |
| ALIEN |
| GT198 |
| MRF1 |
| NRIP |
| TRAP |
| ASC1 |
| E2 |
| E7 |
| E6 |
| RNASEL |
| SMAD8 |
| RACK1 |
| ARA24 |
| PRK1 |
| ARA55 |
| GAK |
| FKBP52 |
| BAG1L |
| HSP90A |
| RAD9 |
| DNAJA1 |
| KU70 |
| HMRNPA1 |
| ZIMP10 |
| ARIP3 |
| SUMO3 |
| ARA54 |
| RNF4 |
| JMJD2C |
| G9A |
| PRMT5 |
| CAF |
| HDAC7 |
| HBO1 |
| SRC2 |
| ARIP4 |
| BAF57 |
| SRC3 |
| CBP |
| HDAC |
| PRMT1 |
| NSD1 |
| E6-AP |
| JHDM2A |
| PIRH2 |
| CHIP |
| USP10 |
| PIASY |
| UBC9 |
| GPCR |
| HA |
| AR3 |
| GAB1 |
| IKK |
| cAMP |
| RXFP1 |
| STAT5A |
| NOX5 |
| SF1 |
| BAK |
| HKII |
| MAPK |
| MEK |
| ERK |
| ABCB1 |
| ARV |
| PDPK-1 |
| JUN |
| PHLPP |
| FKBP5 |
| USP12 |
| ASCT1 |
| ASCT2 |
| SNAT2 |
| TRAF1 |
| CCNE2 |
| SREBF1 |
| NANOG   | BTG1 | | --- | | MST1 | | LATS2 | | KDM5D | | AURKA | | HSL | | STAR | | TCTP | | SLPI | | GR | | ARRB2 | | CK-18 | | MYCN | | FBXW7 | | MDK | | HEY-1 | | HES-1 | | HSF1 | | HAX1 | | SERPINE2 | | GABARAPL1 | | P62 | | GRP78 | | STK11 | | LC3B | | ABHD4 | | GUCY1A3 | | CRAF | | GATA3 | | FRA1 | | CRM1 | | PAR-4 | | AGO2 | | SATB1 | | LEF1 | | ARF1 | | P63 | | MAOA | | SCRIB | | SHP2 | | PAR3 | | CEACAM1 | | ZO-1 | | HSP70 | | CTR | | CT | |
